# Measuring the completeness of death registration in 2844 Chinese counties in 2018

**DOI:** 10.1186/s12916-020-01632-8

**Published:** 2020-07-03

**Authors:** Xinying Zeng, Tim Adair, Lijun Wang, Peng Yin, Jinlei Qi, Yunning Liu, Jiangmei Liu, Alan D. Lopez, Maigeng Zhou

**Affiliations:** 1 Chinese Center for Disease Control and Prevention, Beijing, China; 2 Melbourne School of Population and Global Health, The University of Melbourne, Carlton, Victoria Australia; 3 National Center for Chronic and Noncommunicable Disease Control and Prevention, Chinese Center for Disease Control and Prevention, 27# Nanwei Road, Xicheng District, Beijing, 100050 China

**Keywords:** Completeness of death registration, County level, China

## Abstract

**Background:**

Death registration completeness has never been assessed at the county level in China. Such analyses would provide critical intelligence to monitor the performance of the vital registration system and yield adjustment factors to correct death registration data, thereby increasing their policy utility.

**Methods:**

We estimated the completeness of death registration for 31 provinces and 2844 counties of China in 2018 based on death data from the China Cause of Death Reporting System (CDRS) by using the empirical completeness method. We computed the root mean square difference (RMSD) of county-level completeness compared with provincial-level completeness to study intra-provincial variations. A two-level (province and county) logistic regression model was fitted to explore the association between county-level registration completeness and a set of covariates reflecting socioeconomic status, healthcare quality, and specific strategies and regulations designed to improve registration.

**Results:**

In 2018, the overall death registration completeness for the CDRS in China was 74.2% (95% uncertainty interval [UI] 66.2–80.4), with very little difference for males and females. Geographical differences in completeness were higher across counties than across provinces. The county-level completeness ranged from 2.4% (95% UI 1.0–5.0%) in Burang County, Tibet, to 100.0% (95% UI 99.9–100.0%) in Guandu District, Yunnan. The coastal provinces of Jiangsu, Guangdong, and Fujian, with higher overall completeness, contained counties with low completeness; conversely, the underdeveloped provinces of Guangxi and Guizhou, with lower overall completeness, included some counties with high completeness. GDP, education, population density, minority population, healthcare access, and registration strategies were important drivers of the geographical differences in registration completeness.

**Conclusions:**

There are marked inequalities in registration completeness at the county level and within provinces in China. The socioeconomic condition, the implementation of specific registration-enhancing initiatives, and the availability and quality of medical care were the primary drivers of the observed geographical variation. A more strategic approach, with more research, is required to identify the main reasons for death under-reporting, especially in the poorer performing counties, to guide remedial action.

## Background

Reliable and timely information on mortality patterns and levels for national and subnational populations is fundamental for informing the development, implementation, and evaluation of health policy [1]. In China, such intelligence is particularly critical given the large population spread over a vast area with rapid epidemiological transition bringing numerous challenges, including an aging population and a significant burden of non-communicable diseases.

Prior to 2013, the essential data on the causes of death in China were provided by two sample-based mortality surveillance systems, the nationally representative Disease Surveillance Points (DSP) system of the Chinese Center for Disease Control and Prevention (CDC), and the vital registration (VR) system of the Chinese Ministry of Health (MOH) [2]. The DSP system was initially established in 1978 in Dongcheng and Tongxian counties of Beijing and expanded to 161 points (each point corresponding to a county or district) in 31 provinces covering around 73 million population in 2004 [2]. The VR system was built in the 1950s in 13 cities and progressively enlarged to 319 counties/districts in 22 provinces covering about 230 million people in 2012 [3]. These two systems overlapped in 42 counties [3]. The VR system, while not representative, was able to provide more accurate estimates of the proportion of deaths due to specific causes because of the larger sample of deaths than the DSP system. Conversely, the DSP system reflected the total mortality, the broad cause-of-death distribution, and the geographic distribution of deaths more accurately, because of the nationally representative sampling strategy. Differences between the two systems and their development are described in detail elsewhere [3].

In 2013, the VR and DSP systems were integrated and expanded to 605 points to become the new DSP system under the responsibility of China CDC, covering approximately one quarter of the total population of China and providing representative death data at both national and provincial levels [3]. Since the late 1990s, many provincial CDCs expanded their own death surveillance and some counties also collected death data [4]. Meanwhile, starting in 2004, the vast majority of hospitals in China also reported deaths to the China CDC [5]. Collectively, the new DSP system, the expanded provincial and county (non-DSP) registration system, and the in-hospital death reports form the China Cause of Death Reporting System (CDRS) report over 7.5 million deaths annually in real time using an internet-based reporting system established and operated by China CDC. Given the procedures and mandates of the three individual sources of mortality data, there is almost no duplication. Further, the death data from the CDRS is regularly verified and compared with data from the Ministry of Public Security and the Ministry of Civil Affairs of China to create a single, comprehensive mortality surveillance system [6]. The China CDRS was established to capture every death in every county as mandated by the National Health and Family Planning Commission in 2014 [7]. In 2018, the number of deaths registered by the CDRS was 7.7 million or about three quarters of the 10.5 million deaths estimated to have occurred in 2017 [8].

Although China has made tremendous progress in capturing deaths, approximately one in four deaths still go uncounted, with implications for reliably measuring the impact of programs designed to reduce mortality. Given the regional heterogeneity of key factors likely to affect death registration, including economy, health resource allocation, data collection policy enforcement, and coordination among multiple institutions [1, 9], death registration completeness can be expected to vary substantially across counties. However, the extent of this variation in completeness has never been assessed in China. Such analyses would not only provide the critical intelligence to monitor the performance of the death registration system, and to target interventions accordingly, but also provide the adjustment factors to correct death registration data to produce more reliable mortality statistics that serve the current needs of policymakers.

In this paper, we provide comprehensive estimates of death registration completeness for each of the 2844 Chinese counties in 2018 based on the CDRS. It is unlikely that targeted support to improve mortality surveillance systems in China can be effectively garnered from provincial-level analyses, given the heterogeneous performance of counties within provinces. We also assess how completeness is related predicted to likely key determinants, including economic status, availability and quality of healthcare, and population density.

## Methods

### Data

We used China CDRS as our source of death registration data. The CDRS in principle covers the entire population in each county or district. Death occurring in any county should be reported to the CDRS by that county, whether for local residents (i.e., persons living in the county for more than 6 months) or non-local residents. For each decedent, information is collected on basic demographic characteristics, residential address, place of death, and cause of death. Decedents are counted as local residents. Where they had lived in the county reporting the death less than 6 months, they are counted in their previous counties of residence. Resident population counts by county, age, and sex were obtained from the National Bureau of Statistics (NBS). The population at the end of 2017 was used as the mid-year population of 2018. Summary statistics on registered deaths and population size for each county (according to quartiles) are provided in Additional file 1: Table S1.

We also collated county-level data in 2017 on the gross domestic product (GDP) per capita, mean years of education per capita, and proportion of minority population from the national census and provincial-, city-, and county-level statistical reports. We evaluated the quality of these original data. Some data were missing for about 30% in some counties; further, we found that the values of GDP and mean education years in some counties for some years were very low or very high. To improve the reliability of these estimates, for GDP, we performed a spatiotemporal analysis to impute missing values and smooth the trend. For mean years of education, we used a proportional change model to project the annualized rate of change between 2000 and 2010 in order to obtain the value for 2017. We computed lag-distributed income (LDI) as the weighted average of GDP over a 5-year period. The average population density at the county level was estimated by dividing the resident population by county area (square kilometer) obtained from the National Ministry of Civil Affairs [10]. Additionally, scores on healthcare access and quality (HAQ) were obtained at the provincial level from the Global Burden of Disease 2017 (GBD2017) Study, reflecting health system capacity for timely and efficient detection, management, and treatment of disease [11]. The proportion of ethnic minority population was categorized into quartiles since only when the proportion of ethnic minority population in a region reaches a certain level will it be expected to clearly demonstrate an effect on death registration completeness.

### Estimation of completeness of death registration

We estimated the death registration completeness of the CDRS by using the empirical completeness method developed by Adair and Lopez [12]. This method predicts completeness of death registration based on relatively limited data: registered crude death rate, under-5 mortality rate (5q0), population age structure, and under-5 death registration completeness. The method has significant advantages over traditional demographic methods to estimate completeness, particularly death distribution methods, in that it does not depend on stringent and often unrealistic assumptions about population stability and migration, can be readily applied at subnational levels, and produces results for recent years. More details about the model and coefficients used in this study are provided in Additional file 1: Table S2.

The GBD Study has estimated Chinese county-level 5q0 for both sexes for 2012, the only study to our knowledge to do so [13]. The county-level 5q0 (and 95% uncertainty interval [UI]) for 2018 was estimated by assuming that the ratio of county to province 5q0 for both sexes did not vary between 2012 and 2018 and the 2018 county-level ratio of sex-specific to both sexes 5q0 did not vary within each province (Additional file 1: p 2). Given the relatively brief time interval, and the already comparatively low levels of 5q0 in China in 2012, this assumption would appear reasonable.

At the national level, we also estimated completeness of death registration using the 5q0 from the United Nations Inter-Agency Group for Child Mortality Estimation (IGME) [14]. However, it should be noted that the model itself was developed using GBD 5q0 and all-age mortality data, including from China, and so to ensure consistency of the relationship between these variables and hence produce reliable estimates it is preferred to use the GBD rather than IGME 5q0.

We calculated 95% UI for the completeness estimates by accounting for uncertainty arising from (1) the empirical completeness model, (2) 5q0 (95% uncertainty intervals were provided by the GBD; we incorporated these assuming a normal distribution of the natural log of 5q0), and (3) stochasticity in the number of registered deaths (assuming a Poisson distribution). For each province and county, we produced 1000 simulations of completeness incorporating the three types of uncertainty simultaneously in each simulation. That is, in each simulation, the 5q0 and number of registered deaths were randomly generated based on their mean and assumed distribution; these were input into the model, which produced a completeness estimate that also incorporated the variance of each model coefficient. The 95% UI for completeness is then defined by the 2.5 and 97.5 percentiles of 1000 completeness estimates generated by the simulation results.

We investigated intra-provincial variation in completeness of death registration by computing the root mean square difference (RMSD) of county-level completeness compared with provincial-level completeness (Additional file 1: Table S4).

We undertook an independent sensitivity analysis to examine the robustness of the completeness estimation using 5q0 and population data from the separate death registration system for Anji, Yunhe, and Longyou Counties of Zhejiang province. These data cover the entire Hukou population (a population that is registered in the province) and is a different system to the CDRS system run by the China CDC. We used this data source to estimate completeness of death registration in these counties and compared them to the estimates in the primary analysis of this study. The Hukou death registration system of Zhejiang, a very developed province, is subject to strong data quality controls, and some counties (e.g., Anji, Yunhe, and Longyou Counties) have a very high proportion of the resident population that are registered as Hukou. Hence, the 5q0 and population data for these counties are likely to be valid and independent alternative inputs to estimate death registration completeness.

### Socio-economic determinants of completeness of death registration

Using a two-level (provincial and county levels) logistic regression model (Additional file 1: p 3-p4), we explored the association between county-level completeness and a set of covariates reflecting socioeconomic status and healthcare quality, including income, education, minority population, population density, and healthcare access. We also considered whether the county was included in the DSP system, because this would likely led to greater awareness of the need to register deaths and more intensive surveillance procedures to do so. In particular, registration-enhancing policies and strategies were more likely to be implemented in the DSP counties, which can be expected to favorably influence the timeliness and completeness of death information reporting to the CDRS. A step-wise approach to model fitting was used; firstly, the covariates relating to the population were included in the model, followed by system-level covariates.

The median rate ratio (MRR) [15] was used to estimate geographic variation in completeness between two randomly selected provinces/counties. MRR is defined as the median value of the odds ratios (OR) between two random areas and calculated by translating area-level variance into an OR scale (Additional file 1: p 4). An MRR equal to 1 suggests no geographical variation in completeness, while MRR above 1 indicates that geographical variation is present. The contributions of these socioeconomic determinants can be assessed through the OR and its 95% confidence interval (95% CI) and the proportional change in variance (PCV) [16] (Additional file 1: p4) at the provincial and county levels.

We also used the variance inflation factor (VIF) function to assess whether there was multicollinearity among these socioeconomic determinants in the model. The VIF for each determinant (Additional file 1: Table S5) was much lower than 10, implying that these determinants were not correlated and could be applied in the model. SAS9.4 was used for all regression analyses.

## Results

In 2018, the all-age completeness of death registration of the China CDRS was estimated using 5q0 from the GBD to be 74.2% (95% UI 66.2–80.4) for both sexes, with very little difference for males and females. Using the alternative, lower 5q0 from the IGME, implying much higher under-5 completeness, the all-age estimated completeness was much higher, at 83.0%, 95% UI 77.1–87.1 (see Additional file 1: Table S3). There was a wide geographical variation in death registration completeness among provinces, ranging from 15.6% (Tibet) to 94.3% (Liaoning). Provinces with higher under-5 completeness had higher all-age completeness, as expected. Apart from Liaoning, the highest estimated levels of completeness for all ages were reported for Jiangsu (93.0%) and Fujian (89.8%), while the lowest completeness occurs in the southwest and northwest provinces, including, in addition to Tibet, Xinjiang (30.4%) and Guizhou (37.1%).

Our findings show that death registration completeness for children under-5 was uniformly lower than that for all ages in all provinces except for Tibet, where both measures were very low. The ratio of all-age to under-5 completeness ranged from 1.3 in Shanghai to 4.5 in Hebei, with under-5 completeness between 22 and 76% across provinces (Table 1). This was similar to the age pattern of the incompleteness of death registration reported elsewhere [1, 17–20], no doubt reflecting the tendency for poorer populations in particular to fail to register both the birth and the death when a child dies in the first few weeks or months of life.

**Table 1:** Completeness of death registration at all ages and age under 5 years, by province, China, 2018

Province	Death registration completeness (%), all ages			Death registration completeness (%), under 5 years		
	Both sexes	Male	Female	Both sexes	Male	Female
China	74.2 (66.2, 80.4)	73.6 (65.9, 80.4)	75.1 (67.5, 81.2)	30.6	30.7	30.6
Liaoning	94.3 (91.4, 96.5)	93.9 (90.7, 95.9)	94.0 (90.8, 96.2)	59.6	58.9	61.0
Jiangsu	93.0 (88.7, 95.6)	91.5 (87.4, 94.4)	93.5 (89.9, 96.0)	60.6	59.3	62.4
Fujian	89.8 (84.4, 93.3)	89.5 (84.9, 93.4)	89.8 (84.6, 93.1)	51.6	53.3	49.0
Shanghai	89.5 (84.4, 93.8)	88.2 (82.8, 92.2)	89.6 (84.6, 93.7)	69.2	68.8	70.0
Guangdong	86.7 (80.2, 91.3)	85.7 (79.1, 90.1)	87.6 (81.6, 92.3)	60.7	61.3	59.5
Yunnan	84.2 (76.1, 90.3)	84.4 (75.7, 90.7)	82.8 (75.3, 88.6)	52.8	55.7	49.1
Zhejiang	83.4 (76.6, 88.4)	82.3 (75.9, 87.5)	84.4 (78.2, 89.6)	40.7	40.4	41.0
Shandong	83.1 (76.7, 88.6)	82.4 (75.6, 87.9)	83.3 (76.5, 88.6)	34.9	34.1	35.9
Hunan	82.9 (76.4, 88.3)	82.0 (75.2, 87.1)	84.0 (77.5, 89.1)	32.6	32.2	33.1
Tianjin	82.8 (76.2, 87.9)	78.5 (71.2, 84.6)	87.4 (82.1, 91.2)	39.4	37.5	42.1
Beijing	80.7 (73.0, 87.7)	79.5 (72.2, 85.8)	82.9 (75.5, 89.0)	49.4	49.1	50.5
Henan	76.8 (67.1, 84.2)	75.7 (66.5, 83.3)	79.7 (71.7, 86.4)	23.5	22.2	25.4
Chongqing	76.7 (68.9, 83.7)	75.9 (67.6, 82.2)	76.7 (68.4, 83.5)	24.4	24.0	25.1
Jilin	76.3 (68.1, 82.6)	77.6 (69.6, 84.0)	75.1 (66.4, 82.2)	33.3	34.7	31.5
Hubei	75.0 (65.6, 82.3)	75.2 (67.3, 81.6)	74.3 (65.1, 81.2)	33.0	33.8	31.7
Ningxia	74.6 (64.5, 82.4)	72.5 (61.5, 81.5)	75.8 (67.8, 83.8)	48.4	46.8	50.7
Guangxi	73.6 (65.8, 80.9)	77.1 (69.5, 82.8)	70.0 (61.5, 76.8)	30.2	32.7	26.8
Heilongjiang	72.4 (63.7, 79.2)	72.9 (64.8, 79.6)	71.9 (63.0, 79.4)	24.7	23.2	27.0
Anhui	70.3 (61.6, 77.5)	71.3 (63.0, 78.4)	69.5 (59.8, 77.4)	23.1	25.1	20.2
Sichuan	69.5 (59.6, 77.6)	68.9 (59.0, 77.9)	67.8 (58.1, 75.9)	27.4	28.3	26.3
Hebei	68.0 (59.1, 75.8)	67.9 (59.1, 76.1)	70.6 (61.2, 78.3)	15.1	14.8	15.5
Inner Mongolia	66.0 (56.0, 74.4)	65.8 (56.1, 74.0)	66.5 (57.1, 75.5)	28.6	27.0	31.2
Shanxi	61.6 (52.3, 69.7)	59.9 (50.6, 68.7)	66.5 (57.6, 74.5)	21.4	20.6	23.0
Jiangxi	60.4 (50.3, 68.4)	59.7 (50.3, 67.9)	61.2 (52.3, 69.9)	24.9	25.2	24.4
Qinghai	59.7 (48.6, 71.1)	58.8 (46.4, 70.2)	60.9 (49.8, 70.0)	41.8	43.0	40.6
Shaanxi	57.3 (45.4, 65.4)	56.2 (45.6, 67.5)	57.4 (47.7, 67.8)	15.4	15.8	14.8
Gansu	43.3 (34.8, 52.3)	42.4 (33.3, 51.1)	46.7 (37.5, 55.7)	16.0	15.5	16.8
Hainan	41.0 (32.8, 50.2)	47.6 (38.2, 57.1)	38.1 (29.8, 48.4)	17.3	18.4	16.2
Guizhou	37.1 (27.8, 47.5)	39.8 (29.4, 51.6)	35.3 (26.6, 44.2)	23.4	24.1	22.3
Xinjiang	30.4 (22.5, 39.9)	30.0 (20.8, 39.5)	34.8 (25.6, 43.9)	19.2	19.6	19.1
Tibet	15.6 (10.7, 22.8)	15.1 (10.5, 21.6)	18.8 (13.3, 25.9)	15.7	17.5	13.6

**Fig. 1:**
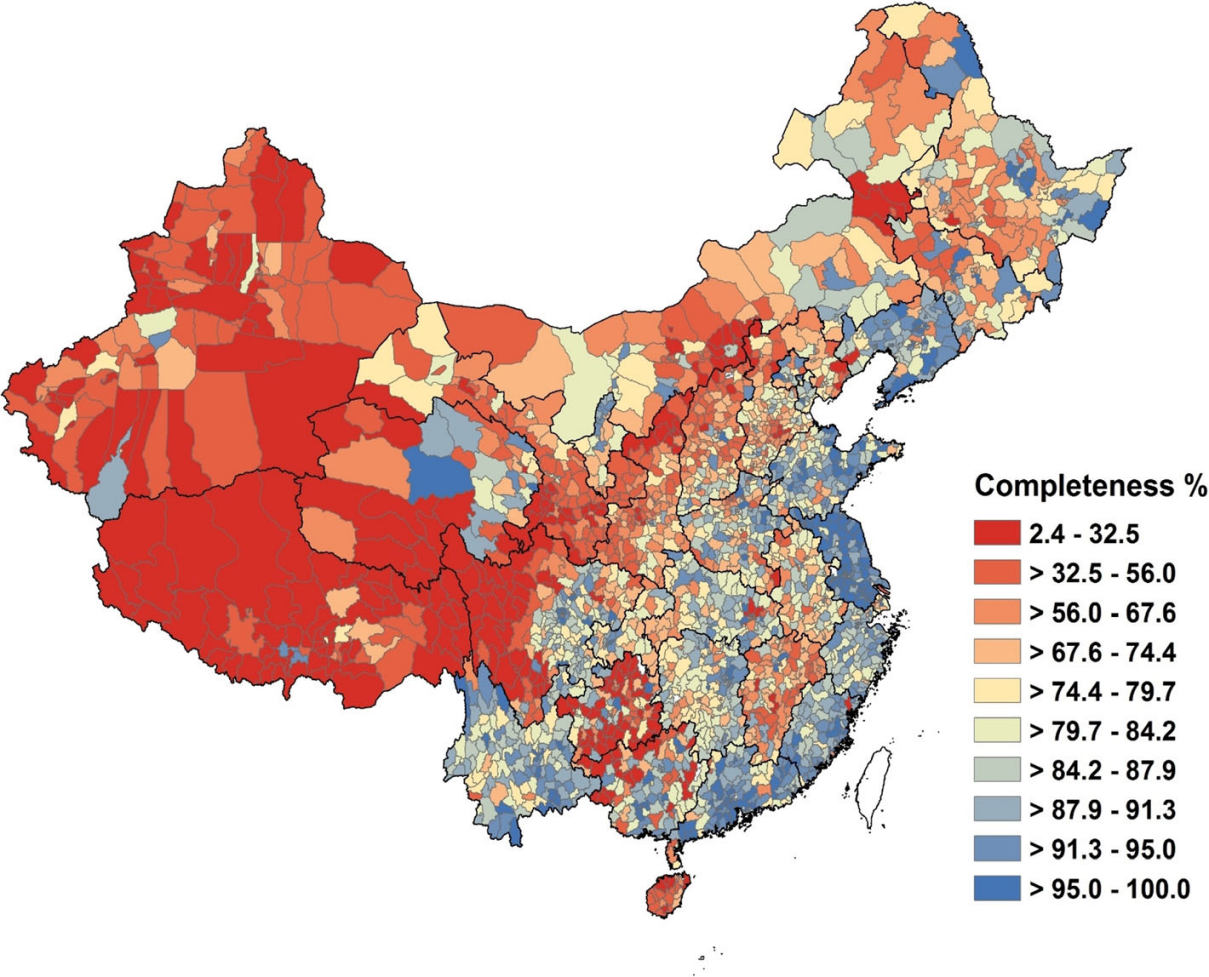
Death registration completeness (%) for both sexes in 2844 Chinese counties in 2018

As might be expected, the variation in death registration completeness was higher across counties than across provinces, reinforcing the policy value of assessing completeness at the county level. At the county level, the completeness ranged from 2.4% (95% UI 1.0–5.0%) in Burang County, Tibet, to 100.0% (95% UI 99.9–100.0%) in Guandu District, Yunnan. More details on the county-level completeness are provided in Additional file 1: Table S6 which shows, for each of the 2844 Chinese counties, the registered death rate and the estimated death registration completeness in 2018. Six hundred eighty (23.9%) counties in China had estimated levels of completeness higher than 90%, including 88 of 96 counties in Jiangsu (91.7%), 65 of 100 counties in Liaoning (65.0%), 78 of 122 counties in Guangdong (64.5%), 44 of 83 counties in Fujian (53.0%), and 8 of 16 counties in Shandong (50.0%). These counties were concentrated mostly in the east and southeast coastal provinces with more developed economies (Fig. 1). By contrast, 197 (7.0%) counties had estimated completeness lower than 20%, mostly located in Tibet (52 of 74 counties), Guizhou (37 of 87 counties), Xinjiang (25 of 98 counties), and Gansu (14 of 87 counties).

Perhaps more surprisingly, several underdeveloped provinces, including Guangxi, Guizhou, Sichuan, Qinghai, and Inner Mongolia, with lower levels of overall completeness (Table 1), contained several counties where completeness was relatively high (Fig. 1). For example, in Guizhou, although death registration completeness was less than 30% in nearly half the counties, 11 of 87 counties had completeness higher than 85%, including Kaiyang County (97.0%, 95% UI 93.5–99.0), Nanming District (95.3%, 89.1–98.4), and Jiangkou County (95.2%, 88.2–98.7), among others. In the coastal provinces of Jiangsu, Guangdong, and Fujian with high levels of overall completeness, there were nonetheless still a few counties with low completeness, such as Qinghe District (44.9%, 29.0–63.8) in Jiangsu, Suixi County (47.6%, 35.9–61.5) in Guangdong, and Fu’an City (27.3%, 19.9–35.9) in Fujian. These observations are perhaps counterintuitive but point to the influence of strong local factors which appear to affect levels of under-registration in unpredictable ways but which need to be understood if overall death registration in China is to improve significantly on current levels. The findings also confirmed the policy utility of assessing completeness levels for as local a population as possible in order to more effectively target intervention strategies.

Table 2 presents the results of the sensitivity analysis. The population counts and age structure (the fraction of the population aged 65 years old and over) of Anji, Yunhe, and Longyou Counties were taken from the NBS, and the counties were very close. Due to the slightly higher 5q0 from the counties than the GBD (and therefore lower under-5 completeness), the all-age estimated completeness of death registration was slightly lower (less than two percentage points difference) when we used the 5q0 and population data from the county as input data. However, these differences were small. The consistent findings from the application of alternative data sources provide some support for the robustness of the empirical completeness model outputs.

**Table 2:** Completeness of death registration in Anji, Yunhe, and Longyou Counties of Zhejiang province: sensitivity analysis based on different data sources of 5q0 and population structure

	Anji County	Yunhe County	Longyou County
Registered deaths from the CDRS	3213	837	2776
5q0 from the GBD study (1/1000)	5.5	6.7	7.5
5q0 from the county (1/1000)	5.9	7.5	8.2
Population from the National Bureau of Statistics (NBS)	484,902	114,700	370,799
% 65 years old and over from the NBS (%)	13.1	12.9	15.4
Population from the county	477,337	113,954	385,879
% 65 years old and over from the county (%)	14.3	13.3	15.9
Death registration completeness (%)^a^, under 5 years	42.1	12.2	70.1
Death registration completeness (%)^a^, all ages	89.9	82.8	91.8
Death registration completeness (%)^b^, under 5 years	40.6	11.4	67.9
Death registration completeness (%)^b^, all ages	88.2	81.7	88.9

^a^Death registration completeness was estimated using the 5q0 of the GBD and population from the NBS

^b^Death registration completeness was estimated using the 5q0 and population from the counties

**Fig. 2:**
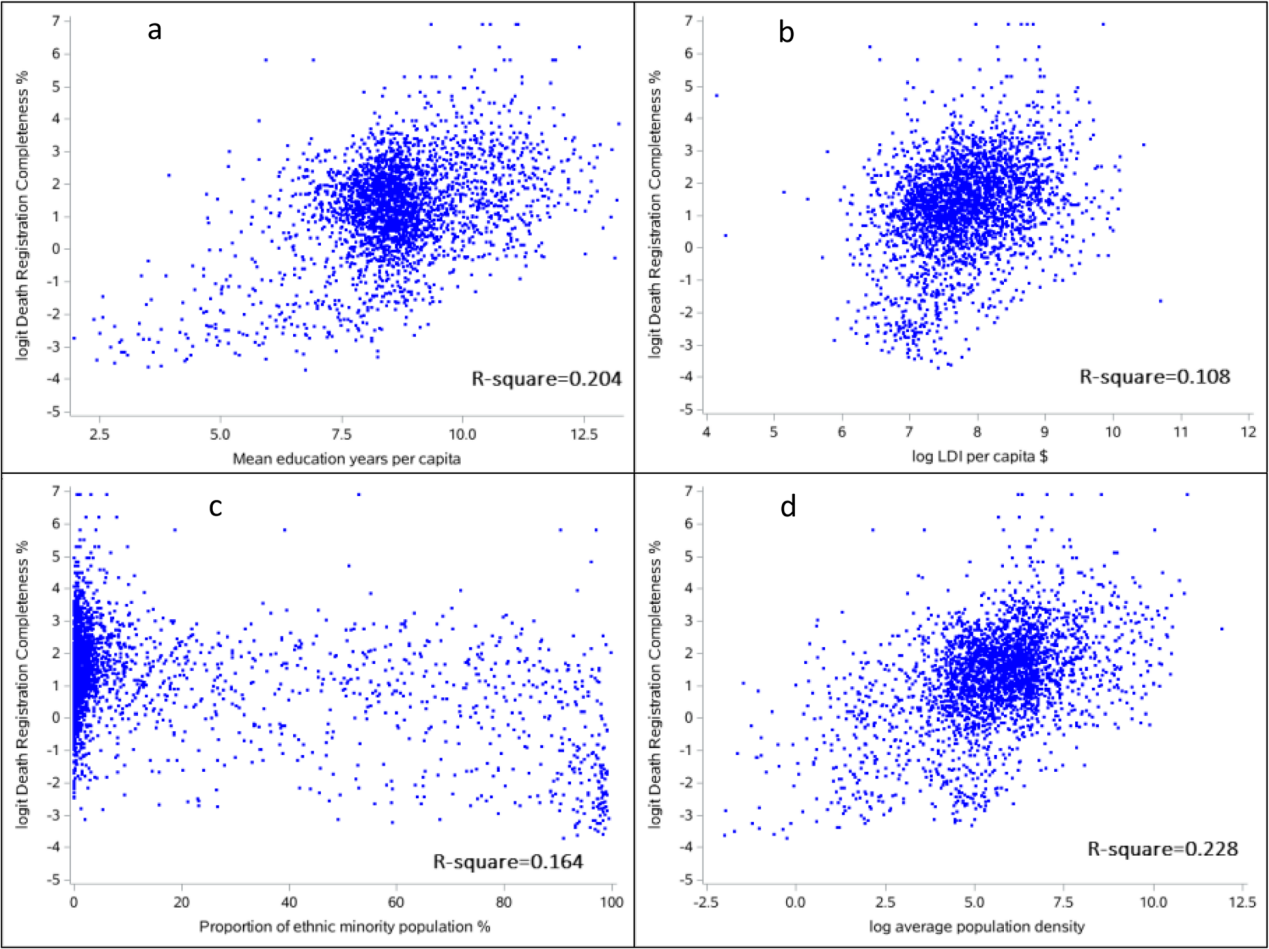
Scatter plots for death registration completeness (%) and socioeconomic determinants for both sexes in 2844 Chinese counties in 2018

To assess how death registration completeness varied with possible determinants, we provide scatter plots to visualize the relationship between completeness and some potential key socioeconomic determinants (Fig. 2). Of these, mean education years per capita (Fig. 2a), lag-distributed income (Fig. 2b), and average population density (Fig. 2d) were weakly positively related to levels of completeness (*R*^2^ respectively of 0.20, 0.11, and 0.23). Conversely, the higher proportion of ethnic minority in the population (Fig. 2c, *R*^2^ = 0.16), the lower the level of completeness, although again, the association was comparatively weak.

We ascertained what might be the main drivers of these observed geographical differences in completeness of death registration by fitting a two-level (province and county) logistic regression model (Table 3). Mean education years, income, and population density were all positively associated with completeness. On the other hand, the regions which had a high proportion of minority ethnicities in the population tended to have lower completeness. There was no statistically significant difference in completeness between males and females (model 1).

**Table 3:** Socio-economic determinants of completeness of death registration (logit) in China in 2018

	Model 1		Model 2	
	OR (95% CI)	*P*	OR (95% CI)	*P*
Fixed effects				
Gender (ref: male)	1		1	
Female	1.05 (0.99, 1.11)	0.10	1.05 (0.99, 1.11)	0.09
Mean education years per capita	1.31 (1.27, 1.36)	< 0.01	1.30 (1.26, 1.35)	< 0.01
Log lag distribute income per capital ($)	1.14 (1.08, 1.21)	< 0.01	1.13 (1.07, 1.19)	< 0.01
Log population density (person per km^2^)	1.04 (1.01, 1.07)	< 0.01	1.03 (1.01, 1.06)	0.02
Proportion of ethnic minority population (%) (ref: 0–0.3)	1		1	
0.3–1.5	0.96 (0.89, 1.05)	0.37	0.96 (0.89, 1.04)	0.34
1.5–13.1	0.92 (0.84, 1.01)	0.09	0.91 (0.83, 1.01)	0.07
13.1–99.8	0.66 (0.59, 0.75)	< 0.01	0.66 (0.59, 0.74)	< 0.01
DSP (ref: yes)			1	
No			0.60 (0.57, 0.64)	< 0.01
HAQ Index			1.04 (1.01, 1.07)	0.01
Random effects				
Province-level variance (SE)	0.617 (0.162)		0.519 (0.139)	
MRR (provinces)	2.12		1.99	
PCV (provinces) (%)	44.14		53.05	
County-level variance (SE)	1.101 (0.021)		1.059 (0.02)	
MRR (counties)	2.72		2.67	
PCV (counties) (%)	13.39		16.68	

After the inclusion of a variable signifying whether the county is a DSP or not, as well as the HAQ Index for each province, PCV for the province level significantly increased, implying better health services were potentially important for promoting death registration completeness, as estimated in model 2. Compared to DSP counties, non-DSP counties had around 40% lower completeness. HAQ Index was positivity associated with completeness. Overall adjustment for all socioeconomic determinants accounted for approximately 53.1% of the province-level and 16.7% of the county-level variation in death registration completeness.

## Discussion

In this study, we assess death registration completeness at both the province and county levels in China and corresponding determinants. These results will be important for central and local governments to target further efforts to strengthen death registration in areas with markedly lower completeness. The results will also aid researchers and public health workers to more reliably adjust deficient death registration data on a county-by-county basis to produce more accurate mortality statistics at the county, provincial, and national levels.

Our findings have immediate relevance for the monitoring of progress with national development goals and improving understanding of health and mortality inequalities in China. As a response to the United Nations Sustainable Development Goals, the Chinese government in 2016 issued *Healthy China 2030* in which they proposed explicit mortality-related targets, such as by 2030, increasing life expectancy to 79.0 years reducing infant and under-5 mortality to 5.0‰ and 6.0‰, respectively; and decreasing maternal mortality to 12 per 100,000 people, and requested each province to set their own targets [21, 22]. Timely and complete death registration data covering the whole population is fundamental for quantifying and monitoring progress on these indicators. We estimate that death registration completeness of the Chinese CDRS was 74.2% in 2018, very similar to that (73.6%) estimated by the GBD study 2017 using quite different methods and data sources [8]. A previous study [23] had reported that the estimated death registration completeness in the CDRS was 56% (6.0 million registered deaths) in 2014 and 58% (6.2 million registered deaths) in 2015 using capture-recapture methods. Over the past 5 years, there has been a tremendous improvement in the coverage and completeness of the CDRS, with the annual number of deaths registered increasing from 6.0 million in 2014 to 7.7 million in 2018. Some studies [24–26] of mortality data reported in China’s Population Census Data have shown serious under-reporting of deaths, especially among children and the population aged 60 years and over. For example, in the 2010 census, the completeness of death registration was 40% and 80% respectively for children and the elderly population [26]. Death registration completeness based on the DSP system has also been reported in previous studies [19, 27] noting that completeness was generally higher in the DSP populations due to the implementation of registration-enhancing policies and strategies.

There are significant geographical differences in death registration completeness across counties in China. The coastal and wealthier regions in eastern and southeastern China generally have higher completeness than those in the west and central regions, although somewhat surprisingly, these provinces with high levels of overall completeness contained several counties with very low completeness, such as Jianchang County in Liaoning, Qinghe District in Jiangsu, Fu’an City in Fujian, and Suixi County in Guangdong. Although it is important for central and provincial governments to provide leadership and promote certain policies and strategies on death registration [28], how these are implemented varies considerably at the county level.

The National Health and Family Planning Commission, the Ministry of Public Security, and the Ministry of Civil Affairs of China have jointly issued guidelines to strengthen the administration of death registration, emphasizing the need for data verification and exchange of information among the three departments [6]. As a result, many regions have strengthened procedures for sharing data on deaths across multiple sectors [29, 30]. Not all have, however, due to the non-mandatory nature of the guidance and the lack of attention paid by local governments to death surveillance. In addition to further promoting multi-sectoral data exchange, there will be scope for greater triangulation of existing data sources in order to better understand data quality and biases. For example, a systematic program of data comparison and verification between the CDRS and the Population Registration Database maintained by the Center for Health Statistics and Information of the National Health Commission in China [22] would yield immediate benefits in terms of improving understanding of data quality and factors affecting completeness.

Interestingly, in some underdeveloped and remote provinces with low levels of overall death registration completeness, such as Guangxi, Guizhou, Sichuan, Qinghai, and Inner Mongolia, there are counties which have very high completeness. For example, within Guizhou, completeness in Kaiyang County, Nanming District, Qingzhen City, and so on exceeded 90%. All of these counties were included in the DSP system where multiple specific strategies are used to promote death registration, including special funding by the central government, dedicated staff in hospitals and CDCs who are responsible for registering deaths, regular training and supervision of staff by national and provincial CDCs, critically reviewing death data annually for completeness and accuracy, conducting periodic surveys of deaths under-reporting, and disseminating statistical reports on mortality to the public to raise awareness [2, 3]. Conversely, non-DSPs counties receive much less support from governments and relevant departments and have less funding and staff. Our study confirms that inclusion in the DSP system, with all of its associated data-promoting advantages, was an important determinant of death registration completeness at the county level, particularly in poorer provinces. This factor alone contributed substantially to increasing completeness among counties in some provinces.

Our study has suggested that more minority populations, dispersed population distribution, and lower quality of healthcare were all important factors associated with lower death registration completeness. The majority of counties in Tibet and Xinjiang have very low completeness, and 91% and 63% of the total population in Tibet and Xinjiang, respectively, were minorities [31]. Given their own special culture, beliefs, and customs, most people choose to die at home [32, 33]. After death, they are generally not cremated, but instead undergo a “celestial” burial (corpses eaten by vultures) or are inhumed [34], neither of which requires a death certificate. Furthermore, Tibet and Xinjiang are both vast and sparsely populated regions [9]. In agriculture areas and remote villages, difficult natural environmental conditions and inconvenient transportation present serious barriers for family members to report the home deaths and for village doctors to conduct surveys of under-reporting. In 2015, health system performance in Tibet and Xinjiang was very poor [10]. Inadequate access to healthcare also undoubtedly contributes to the low registration completeness in these regions. In addition, local governments do not provide funds to support death registration, and mechanisms for data exchange across multiple sectors have not been established.

It is reasonable to expect that higher-level socioeconomic development might contribute substantially to death registration completeness, given the experience of more developed countries where very few deaths go unrecorded [35]. Yet, our results indicated that developmental factors such as income, educational attainment, and population density only account for a low proportion of the geographical variation across China. One might have expected a more substantial contribution from this amalgam of development factors, suggesting that more research is needed to more precisely delineate the impact of socioeconomic development, and how it is being modulated, at local and/or national level, on death registration completeness. The influence of socioeconomic conditions is clearly important and seemingly poorly understood, likely affected by staffing, policy implementation, quality of medical services, access to healthcare, health behavior, and awareness of the public about the importance of registering births and deaths, among others. Indeed, they may well play a greater role in raising levels of death registration completeness than classical measures of development such as education and wealth. It would appear that while socioeconomic development is a major underlying determinant of health policies, strategies, and actions, including death registration, once a certain level of socioeconomic development has been achieved, other factors related to the strength of commitment to improving data for policy may well influence death registration much more.

### Limitations

This first-ever comprehensive estimation of death registration completeness based on the China CDRS at the provincial and county levels has some limitations. First, the empirical completeness model was developed using GBD data, which are themselves based on demographic and statistical models. However, the method’s validity has been demonstrated elsewhere by its high concordance with completeness estimates in national populations not included in the database to develop the model, as well as subnational populations [12]. Second, the 5q0 for each county and province was a model-based estimate taken from the GBD 2017 since this was the most recent and comprehensive source of these input data. We have incorporated the uncertainty around these estimates of 5q0 into our own uncertainty intervals of completeness. Third, due to China not having a fully functional vital registration system, the resident population counts by county have been taken from the NBS. These are projected populations, which could be another source of uncertainty for the completeness estimates. We conducted a sensitivity analysis using independent 5q0 and population counts for three counties of Zhejiang province which maintain their own independent vital registration system to estimate the corresponding death registration completeness in the CDRS.

## Conclusion

While China has been very successful in improving levels of death registration among its nearly 1.4 billion citizens, there are marked inequalities in registration completeness across the country. Overall, completeness is higher in more industrialized, wealthier eastern regions than in more rural, poorer western regions, but regional inequality is actually more complicated with significant variations in completeness across counties within both richer and poorer provinces. Socioeconomic condition, a faciliatory environment such as afforded by the presence of the DSPs, and access to quality medical care were the key drivers of the observed geographical variation in completeness in China. To improve death registration completeness further will require targeted research about how non-developmental factors impede or promote registration in those counties and provinces where levels are low. Doing so will ensure that health and social development policy across China will benefit from less biased information about who is dying prematurely, and where.

## Supplementary information

**Additional file 1.** Online material providing details of empirical completeness method, estimation of the county-level under-5 mortality rate for 2018, model for estimating association between county-level death registration completeness and socio-economic determinants, inter-provincial variation in completeness of death registration and supplemental tables (Table S1-S6).

## Data Availability

The datasets used in the current study are available from the corresponding author on reasonable request.

## Abbreviations

5q0: Under-5 mortality rate
CDC: Centers for Disease Control and Prevention
CDRS: Cause of Death Reporting System
CI: Confidence interval
DSP: Disease Surveillance Points
GBD: Global Burden of Disease
HAQ: Healthcare access and quality
IGME: Inter-agency Group for Child Mortality Estimation
LDI: Lag-distributed income
MRR: Median rate ratio
OR: Odds ratio
PCV: Proportional change in variance
RMSD: Root mean square difference
UI: Uncertainty interval
VIF: Variance inflation factor
