# Supplementary material for: Measuring the completeness of death registration in 2844 Chinese counties in 2018
Source: BMC Med. 2020 Jul 3;18:176. doi: 10.1186/s12916-020-01632-8 (PMC7333385; doi:10.1186/s12916-020-01632-8)
Supplement: Supplementary file 1 — Additional file 1. Online material providing details of empirical completeness method, estimation of the county-level under-5 mortality rate for 2018, model for estimating association between county-level death registration completeness and socio-economic determinants, inter-provincial variation in completeness of death registration and supplemental tables (Table S1-S6). [file 12916_2020_1632_MOESM1_ESM.docx]

**Table S1** The 25^th^, 50^th^, and 75^th^ percentile of the registered deaths and population in 2,844 Chinese counties in 2018

|  | Gender | P25^th^ | P50^th^ | P75^th^ |
| --- | --- | --- | --- | --- |
|  | Both sexes | 1062 | 2219 | 3759 |
| Registered Deaths | Male | 626 | 1285 | 2175 |
|  | Female | 427 | 909 | 1581 |
|  | Both sexes | 225,449 | 390,299 | 653,448 |
| Population | Male | 115,603 | 199,605 | 328,808 |
|  | Female | 110,213 | 190,742 | 319,885 |

**Empirical completeness method**

The random effects model predicts the completeness of death registration as follows:

$$logit\left( C_{jk}^{All} \right)=\beta_{0}+{RegCDRsq}_{jk}\times\beta_{1}+{RegCDR}_{jk}\times\beta_{2}+{\%65}_{jk}\times\beta_{3}+{ln \left( 5q0 \right)}_{jk}\times\beta_{4}+C_{jk}^{5q0}\times\beta_{5}+k\times\beta_{6}+e_{jk}+\gamma_{j}$$

where $C_{jk}^{All}$ is the completeness of registration at all ages, *logit(*$C_{jk}^{All}$*)* is $ln\left( \frac{C_{jk}^{All}}{1-C_{jk}^{All}} \right)$, ${RegCDR}_{jk}$ is the registered crude death rate (CDR), ${RegCDRsq}_{jk}$ is the *RegCDR* squared, ${\%65}_{jk}$ is the fraction of the population aged 65 years and over, ${ln(5q0)}_{jk}$is the natural log of the under-five mortality rate, $C_{jk}^{5q0}$ is the completeness of the registered *5q0*, *k* is calendar year, *e* is an error term, *γ* is a country-level random effect, *j* is country and $\beta_{0}$ to $\beta_{6}$ are the coefficients predicted completeness is converted using the inverse logit: $\frac{e^{logit(C_{jk}^{All})}}{e^{logit(C_{jk}^{All})}+1}$. Due to this being a linear model that predicts the logit of completeness, it is rare for a population to have completeness in excess of 99.5%, due to the large relative difference in logit of a proportion approaching 1 (e.g. logit of 99.95% is 7.60 and logit of 99.5% is 5.29). Hence, any county with completeness over 99.5% can, in practical terms, be assumed to have completeness of 100%.

The original empirical completeness method used in this study was developed using a larger database than its original presentation, hence its coefficients are different (Adair and Lopez 2018). The database now includes China (national) from 2008-2017 and China’s provinces for 2017.

**Table S2** The coefficients from the empirical completeness model, both sexes, males and females

| Variable | Coef. | Both sexes | Male | Female |
| --- | --- | --- | --- | --- |
| Constant | $\beta_{0}$ | 29.0554 | 31.7810 | 22.9998 |
| RegCDR squared | $\beta_{1}$ | -0.0169 | -0.0167 | -0.0191 |
| RegCDR | $\beta_{2}$ | 0.6194 | 0.5792 | 0.6819 |
| %65+ | $\beta_{3}$ | -13.5175 | -12.5888 | -17.1125 |
| ln(5q0) | $\beta_{4}$ | -1.0919 | -1.1021 | -1.1507 |
| C_5q0_ | $\beta_{5}$ | 2.1820 | 1.9784 | 1.9093 |
| Year | $\beta_{6}$ | -0.0171 | -0.0185 | -0.0140 |
| Random effects - China | $\gamma$ | -0.48019 | -0.62376 | -0.39223 |

**Estimation of the county-level under-5 mortality rate for 2018**

The county-level under-5 mortality rate (*5q0*) (and 95% uncertainty interval [UI]) for 2018 was estimated by calculating the ratio of county to province 2012 *5q0* (and 95% UI) for both sexes and multiplying by the 2018 provincial *5q0* for both sexes. The 2018 provincial *5q0* for both sexes combined was estimated by using the average annual % decline in *5q0* during 2012-2017 to multiply the 2017 provincial *5q0* from the GBD 2017 Study. Sex-specific county-level *5q0* (and 95% UI) in 2018 was estimated by calculating the ratio of sex-specific to both sexes provincial *5q0* and multiplying by the county-level *5q0* for both sexes. We assumed that the ratio of county to province *5q0* for both sexes did not vary between 2012 and 2018 and the 2018 county-level ratio of sex-specific to both sexes *5q0* did not vary within each province.

**Table S3** The under-5 mortality rate (1/1000) and completeness of death registration in China for 2018 from the UN Inter-agency group for child mortality estimation

| Gender | Under-5 mortality rate (1/1000) | Under-5 death registration completeness (%) | All-age death registration completeness (%) |
| --- | --- | --- | --- |
| Both sexes | 8.60(7.51, 9.83) | 40.9 | 83.0(77.1,87.1) |
| Male | 9.11(7.92, 10.46) | 42.3 | 83.4(78.1,87.6) |
| Female | 8.06(7.00, 9.25) | 38.7 | 82.1(76.5,86.4) |

**Inter-provincial variation in completeness of death registration**

The root mean square difference (RMSD) of county-level death registration completeness compared with provincial-level death registration completeness was calculated as follows:

$${RMSD}_{j}=\sqrt[2]{\frac{\sum_{i=1}^{n_{j}} {({county\_completeness}_{ij}-{province\_completeness}_{j})}^{2}}{n_{j}}}$$

In this equation,${county\_completeness}_{ij}$ is the county-level completeness at all ages and ${province\_completeness}_{j}$ is the province-level completeness.$i$ is county and $j$ is province. $n_{j}$ is the number of counties for each province.

**Table S4** The RMSD of county-level death registration completeness compared with province-level death registration completeness in China in 2018

| Province | Number of Counties | Roots Mean Square Difference of Completeness | | |
| --- | --- | --- | --- | --- |
|  |  | Both sexes | Male | Female |
| Guizhou | 87 | 34.7 | 34.6 | 33.5 |
| Sichuan | 183 | 28.1 | 28.3 | 27.0 |
| Guangxi | 110 | 27.5 | 26.5 | 28.0 |
| Qinghai | 46 | 27.1 | 27.0 | 26.4 |
| Inner Mongolia | 102 | 24.0 | 24.0 | 24.3 |
| Xinjiang | 98 | 23.9 | 24.1 | 23.4 |
| Tibet | 74 | 23.8 | 24.0 | 23.0 |
| Gansu | 87 | 20.6 | 19.9 | 20.9 |
| Shaanxi | 107 | 19.7 | 19.6 | 19.6 |
| Hebei | 168 | 17.9 | 17.3 | 18.2 |
| Hainan | 21 | 17.8 | 17.3 | 17.7 |
| Jilin | 60 | 17.1 | 16.9 | 16.7 |
| Hubei | 103 | 17.1 | 16.9 | 16.8 |
| Shanghai | 16 | 16.7 | 18.2 | 15.0 |
| Shanxi | 118 | 16.4 | 16.3 | 15.2 |
| Jiangxi | 100 | 16.2 | 16.0 | 15.8 |
| Henan | 159 | 15.2 | 15.0 | 15.2 |
| Heilongjiang | 132 | 14.6 | 13.3 | 16.6 |
| Beijing | 16 | 13.7 | 13.5 | 14.4 |
| Anhui | 104 | 13.4 | 12.8 | 13.5 |
| Liaoning | 100 | 12.9 | 12.3 | 14.5 |
| Ningxia | 22 | 12.5 | 13.8 | 11.9 |
| Chongqing | 38 | 10.8 | 10.5 | 11.1 |
| Fujian | 83 | 9.8 | 9.5 | 10.7 |
| Guangdong | 121 | 9.6 | 9.5 | 10.4 |
| Shandong | 137 | 8.6 | 9.0 | 8.7 |
| Hunan | 122 | 8.5 | 8.5 | 8.6 |
| Yunnan | 129 | 8.0 | 7.9 | 8.9 |
| Tianjin | 16 | 7.8 | 9.5 | 5.6 |
| Zhejiang | 89 | 6.8 | 7.2 | 6.5 |
| Jiangsu | 96 | 6.5 | 7.3 | 6.1 |

**Model for estimating association between county-level death registration completeness and socio-economic determinants**

A two-level (province and county-level) logistic regression model was fitted as follows:

$$logit\left( {completeness}_{ij} \right)=$$

$$\beta_{0ij}+\beta_{1ij}\mathrm{gender}_{ij}+\beta_{2ij}{edu}_{ij}{+\beta}_{3ij}{ln(ldi}_{ij})+\beta_{4ij}\mathrm{minority}_{ij}+\beta_{5ij}{ln(density}_{ij})+\beta_{6ij}{dsp}_{ij}+\beta_{7j}{haq}_{j}+\gamma_{j}+\varepsilon_{ij}$$

Where ${completeness}_{ij}$ is the completeness of death registration for all ages at the county level. *logit (*${completeness}_{ij}$*)* is $ln\frac{{completeness}_{ij}}{1-{completeness}_{ij}}. {edu}_{ij}$ is the mean education years per capita.${ln(ldi}_{ij})$ is the natural log of lag-distributed income. $\mathrm{miority}_{ij}$ is the proportion of ethnic minority population, which include 55 ethnic groups such as Manchu, Hui, Uyghur, Mongolian, Zhuang, Korean, excluding the majority Han group. ${ln(density}_{ij})$ is the natural log of average population density, all measured at the county level. ${dsp}_{ij}$ equal to 1 indicates the county is included in the DSPs.${haq}_{j}$ is the HAQ Index at the province level, which reflects health system capacity for avoiding premature deaths from a range of preventable diseases. $i$ is county and $j$ is province. $\gamma_{j}$ and$\varepsilon_{ij}$ are the province-specific and county-specific random effect, respectively. $\beta_{0}$ to $\beta_{7}$ are the coefficients. A step-wise approach to model-fitting was used, firstly, the covariates relating to the population were included in the model, then system-level covariates (DSP and HAQ) were added.

For either the province or county level, the median rate ratio (*MRR*) was calculated as: $MRR=(exp\sqrt{2\times variance}\times0.6745)$, where 0.6745 being the 75th percentile of the cumulative distribution function of the standard normal distribution. An MRR equal to 1 suggests no geographic variations in death registration completeness, while an MRR above 1 indicates the presence of geographic variations. *PCV* was computed as$PCV={({variance}_{r}-{variance}_{c})}/{{variance}_{r}}$, where ${variance}_{r}$ is the variance in the reference model and ${variance}_{c}$ is the variance in the model adjusted for other determinants.


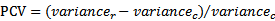

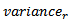

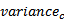


**Table S5** The Variance inflation factor (VIF) for each variable in the two-level logistic regression model

| Variable | Variance inflation factor(VIF) |
| --- | --- |
| Intercept | 0 |
| Gender | 1.00 |
| Mean Education Years per capita | 1.77 |
| Lag Distribute Income per capita $ | 1.25 |
| Proportion of Ethnic Minority Population % | 1.85 |
| Log Population density | 1.19 |
| Disease Surveillance Point | 1.01 |
| Health access and quality Index | 1.85 |

**Table S6** The registered death rate (1/1000) and the completeness (%) of death registration in 2844 Chinese counties in 2018

| Province  name | County  code | County  name | Registered death rate(1/1000) | | |  | Completeness (%) of death registration | | |
| --- | --- | --- | --- | --- | --- | --- | --- | --- | --- |
|  |  |  | Both sexes | Male | Female |  | Both sexes | Male | Female |
| Liaoning | 210106 | Tiexi District | 10.2 | 8.9 | 11.5 |  | 99.9(99.5,100.0) | 99.9(99.1,100.0) | 99.9(99.3,100.0) |
| Liaoning | 210113 | Shenbeixin District | 8.5 | 6.9 | 10.1 |  | 99.7(98.6,100.0) | 99.6(97.3,100.0) | 99.7(97.4,100.0) |
| Liaoning | 210211 | Ganjingzi District | 7.4 | 6.4 | 8.3 |  | 99.5(97.6,99.9) | 99.2(96.5,99.9) | 99.5(97.6,100.0) |
| Liaoning | 210213 | Jinzhouxin District | 6.3 | 5.2 | 7.5 |  | 99.5(98.3,99.9) | 98.8(96.6,99.7) | 99.6(98.3,99.9) |
| Liaoning | 210104 | Dadong District | 8.7 | 7.6 | 9.8 |  | 99.4(97.0,99.9) | 99.2(96.1,99.9) | 99.1(94.9,99.9) |
| Liaoning | 210302 | Tiedong District | 9.6 | 7.9 | 11.4 |  | 99.4(97.8,99.9) | 98.5(94.9,99.7) | 99.6(97.8,100.0) |
| Liaoning | 210303 | Tiexi District | 10.4 | 8.4 | 12.5 |  | 99.3(97.3,99.9) | 99.4(96.7,99.9) | 98.1(90.3,99.8) |
| Liaoning | 211002 | Baita District | 11.7 | 9.4 | 14.1 |  | 99.0(96.1,99.8) | 98.9(94.3,99.9) | 98.2(90.1,99.8) |
| Liaoning | 210902 | Haizhou District | 9.5 | 8.1 | 11.0 |  | 98.9(94.1,99.9) | 98.7(92.6,99.9) | 98.1(85.4,99.9) |
| Liaoning | 210114 | Yuhong District | 9.8 | 8.1 | 11.5 |  | 98.8(96.9,99.6) | 98.9(96.2,99.8) | 97.7(93.6,99.4) |
| Liaoning | 210102 | Heping District | 7.4 | 6.4 | 8.5 |  | 98.7(94.2,99.9) | 98.2(92.2,99.8) | 98.3(91.3,99.9) |
| Liaoning | 210603 | Zhenxing District | 8.6 | 7.8 | 9.3 |  | 98.4(95.4,99.7) | 95.9(88.0,99.1) | 99.3(95.6,99.9) |
| Liaoning | 210304 | Lishan District | 10.0 | 8.6 | 11.5 |  | 98.3(94.4,99.6) | 97.3(90.9,99.6) | 98.5(92.8,99.9) |
| Liaoning | 210212 | Lushunkou District | 6.6 | 5.5 | 7.8 |  | 98.1(91.2,99.7) | 93.9(81.8,99.1) | 99.3(90.1,100.0) |
| Liaoning | 210803 | Xishi District | 9.3 | 8.1 | 10.5 |  | 97.4(85.9,99.8) | 97.6(83.6,99.9) | 94.3(77.4,99.7) |
| Liaoning | 210702 | Guta District | 8.3 | 7.1 | 9.5 |  | 97.3(90.2,99.4) | 96.0(86.1,99.4) | 97.5(87.5,99.8) |
| Liaoning | 210802 | Zhanqian District | 7.6 | 6.3 | 8.9 |  | 97.3(89.8,99.6) | 99.0(93.6,99.9) | 84.6(65.7,96.1) |
| Liaoning | 210103 | Shenhe District | 7.0 | 6.1 | 7.9 |  | 97.2(91.1,99.4) | 98.3(93.0,99.8) | 90.9(79.6,97.4) |
| Liaoning | 210224 | Changhai County | 7.5 | 6.6 | 8.3 |  | 97.2(83.6,99.8) | 81.8(73.1,88.1) | 99.7(84.6,100) |
| Liaoning | 210282 | Pulandian City | 9.2 | 7.9 | 10.3 |  | 97.2(94.6,98.8) | 96.4(92.3,98.6) | 97.4(93.2,99.1) |
| Liaoning | 211004 | Hongwei District | 7.8 | 5.9 | 9.8 |  | 97.2(84.2,99.8) | 98.5(87.4,100) | 86.3(68.1,98.4) |
| Liaoning | 210504 | Mingshan District | 7.4 | 6.4 | 8.4 |  | 96.7(90.2,99.2) | 98.5(92.9,99.8) | 87.7(77.3,96.2) |
| Liaoning | 211102 | Shuangtaizi District | 7.1 | 5.5 | 8.7 |  | 96.7(88.7,99.4) | 98.6(90.7,99.9) | 87.3(72.3,97.7) |
| Liaoning | 210804 | Bayuquan District | 6.3 | 5.6 | 7.0 |  | 96.5(91.4,98.7) | 92.7(84.6,97.3) | 98.4(94.1,99.8) |
| Liaoning | 210283 | Zhuanghe City | 8.7 | 7.7 | 9.7 |  | 96.3(93.0,98.4) | 97.4(93.8,99.1) | 93.0(87.6,96.6) |
| Liaoning | 210502 | Pingshan District | 8.2 | 7.0 | 9.5 |  | 96.3(89.8,99.0) | 92.3(83.2,97.6) | 98.2(91.3,99.8) |
| Liaoning | 210682 | Fengcheng City | 8.7 | 7.0 | 10.4 |  | 95.8(91.8,98.1) | 95.4(90.4,98.2) | 94.0(87.0,98.1) |
| Liaoning | 211011 | Taizihe District | 8.8 | 7.2 | 10.4 |  | 95.7(86.1,99.4) | 94.8(84.9,99.4) | 95.8(84.0,99.7) |
| Liaoning | 210402 | Xinfu District | 11.0 | 9.2 | 12.9 |  | 95.4(88.8,98.7) | 91.7(84.8,97.1) | 96.8(86.1,99.8) |
| Liaoning | 210411 | Shuncheng District | 9.9 | 8.2 | 11.5 |  | 95.4(88.0,99.0) | 96.1(88.3,99.3) | 92.3(80.0,98.4) |
| Liaoning | 210112 | District?Hunnanxin | 7.1 | 5.8 | 8.4 |  | 95.3(90.5,98.1) | 96.3(91.1,98.8) | 92.6(84.3,97.4) |
| Liaoning | 210922 | Zhangwu County | 9.1 | 7.4 | 10.7 |  | 95.3(90.9,97.9) | 93.7(87.7,97.4) | 95.4(89.0,98.6) |
| Liaoning | 210203 | Xigang District | 9.1 | 8.2 | 10.0 |  | 95.2(87.1,98.8) | 96.6(86.7,99.5) | 89.9(78.3,97.7) |
| Liaoning | 210105 | Huanggu District | 7.4 | 6.5 | 8.2 |  | 95.1(87.9,98.9) | 94.4(85.2,98.7) | 94.8(85.1,99.2) |
| Liaoning | 210204 | Shahekou District | 8.3 | 6.9 | 9.8 |  | 95.0(89.9,98.0) | 91.8(85.1,95.9) | 97.0(91.2,99.5) |
| Liaoning | 210423 | Zizhixian | 7.8 | 6.3 | 9.3 |  | 94.9(88.9,97.9) | 97.5(92.7,99.4) | 83.6(73.3,92.3) |
| Liaoning | 211204 | Qinghe District | 9.0 | 7.0 | 11.0 |  | 94.9(81.8,99.4) | 97.4(82.8,99.8) | 83.7(75.6,89.6) |
| Liaoning | 210404 | Wanghua District | 8.4 | 7.4 | 9.3 |  | 94.8(88.1,98.4) | 95.9(87.9,99.1) | 91.2(79.0,98.0) |
| Liaoning | 210522 | Zizhixian | 6.8 | 5.7 | 7.8 |  | 94.8(87.5,97.9) | 97.9(93.4,99.5) | 78.6(70.1,84.9) |
| Liaoning | 211103 | Xinglongtai District | 5.3 | 4.1 | 6.4 |  | 94.7(87.1,98.4) | 95.5(86.7,99.0) | 91.3(77.1,97.9) |
| Liaoning | 211202 | Yinzhou District | 7.5 | 6.5 | 8.5 |  | 94.7(86.7,98.6) | 96.5(87.1,99.5) | 90.5(81.7,97.0) |
| Liaoning | 210602 | Yuanbao District | 8.8 | 7.4 | 10.3 |  | 94.6(83.1,98.9) | 93.3(79.6,98.9) | 95.0(77.3,99.6) |
| Liaoning | 210624 | Zizhixian | 6.9 | 5.9 | 7.9 |  | 94.4(87.7,97.9) | 93.7(83.7,98.2) | 93.0(81.9,98.1) |
| Liaoning | 210782 | Beizhen City | 8.0 | 6.8 | 9.2 |  | 94.1(88.5,97.5) | 92.2(84.0,97.0) | 94.9(88.4,98.5) |
| Liaoning | 210711 | Taihe District | 6.9 | 5.7 | 8.2 |  | 94.0(85.4,98.4) | 93.5(81.7,98.7) | 94.1(83.1,98.9) |
| Liaoning | 211223 | Xifeng County | 7.2 | 5.7 | 8.6 |  | 93.9(87.3,97.6) | 88.3(81.1,94.2) | 97.2(89.3,99.6) |
| Liaoning | 211381 | Beipiao City | 10.4 | 9.1 | 11.7 |  | 93.7(89.2,96.6) | 91.1(85.0,95.2) | 95.0(89.6,98.0) |
| Liaoning | 210422 | Xinbin Manzu Zizhixian | 8.2 | 6.8 | 9.6 |  | 93.1(86.5,96.8) | 95.3(88.2,98.4) | 87.2(78.7,93.6) |
| Liaoning | 210111 | Sujiatun District | 8.3 | 6.9 | 9.6 |  | 93.0(87.3,96.8) | 93.3(85.8,97.2) | 92.3(83.4,96.9) |
| Liaoning | 210281 | Wafangdian City | 8.6 | 7.7 | 9.4 |  | 93.0(88.7,96.3) | 92.9(87.3,96.1) | 92.2(85.5,96.2) |
| Liaoning | 211322 | Jianping County | 9.0 | 7.8 | 10.2 |  | 92.8(88.5,95.7) | 90.2(84.4,94.3) | 94.6(89.7,97.6) |
| Liaoning | 210311 | Qianshan District | 9.5 | 7.7 | 11.4 |  | 92.7(84.2,97.8) | 92.1(83.1,97.9) | 92.3(80.7,98.8) |
| Liaoning | 210181 | Xinmin City | 8.4 | 7.1 | 9.6 |  | 92.6(85.8,96.6) | 89.6(81.4,94.9) | 94.7(88.7,98.3) |
| Liaoning | 210503 | Xihu District | 10.1 | 8.8 | 11.4 |  | 92.3(84.2,97.4) | 95.5(85.5,99.4) | 82.5(74.7,88.7) |
| Liaoning | 210703 | Linghe District | 8.8 | 7.6 | 10.1 |  | 92.3(85.5,96.5) | 93.9(86.3,98.4) | 88.2(82.4,92.5) |
| Liaoning | 210904 | Taiping District | 11.0 | 9.2 | 12.9 |  | 92.2(84.3,97.6) | 94.7(86.2,99.2) | 85.2(77.8,90.6) |
| Liaoning | 210921 | Zizhixian | 8.4 | 6.7 | 10.1 |  | 92.2(87.7,95.1) | 93.9(89.1,96.8) | 87.4(80.2,92.4) |
| Liaoning | 210781 | Linghai City | 8.8 | 7.4 | 10.1 |  | 91.4(85.3,95.1) | 89.0(82.3,94.4) | 93.3(86.4,97.4) |
| Liaoning | 210403 | Dongzhou District | 10.7 | 9.0 | 12.3 |  | 91.0(83.7,96.5) | 93.4(85.5,98.5) | 83.9(76.3,89.4) |
| Liaoning | 211402 | Lianshan District | 6.7 | 5.5 | 8.0 |  | 90.8(81.1,96.2) | 90.6(81.1,96.6) | 88.7(75.2,96.3) |
| Liaoning | 211224 | Changtu County | 7.8 | 6.1 | 9.4 |  | 90.7(85.0,94.3) | 92.0(86.9,95.6) | 88.1(80.7,92.9) |
| Liaoning | 211282 | Kaiyuan City | 7.7 | 6.4 | 9.0 |  | 90.7(84.7,94.5) | 86.0(79.0,91.5) | 94.4(87.5,98.2) |
| Liaoning | 211221 | Tieling County | 6.4 | 5.1 | 7.7 |  | 90.6(79.9,96.7) | 90.8(77.0,97.8) | 88.4(73.0,96.9) |
| Liaoning | 210727 | Yi County | 8.2 | 7.0 | 9.4 |  | 90.4(84.1,94.9) | 87.5(80.3,93.3) | 92.6(83.9,97.7) |
| Liaoning | 210681 | Donggang City | 8.1 | 7.2 | 8.9 |  | 90.1(83.9,94.5) | 89.7(81.3,95.1) | 90.2(83.2,94.9) |
| Liaoning | 211122 | Panshan County | 8.0 | 7.1 | 9.0 |  | 90.0(81.9,94.9) | 88.6(78.9,94.8) | 91.6(83.6,96.6) |
| Liaoning | 210505 | Nanfen District | 7.7 | 6.7 | 8.6 |  | 89.7(72.8,98.8) | 94.0(73.4,99.8) | 78.3(68.9,86.4) |
| Liaoning | 211121 | Dawa County | 6.5 | 5.4 | 7.6 |  | 89.3(82.4,94.5) | 90.4(82.1,95.9) | 87.4(78.5,94.0) |
| Liaoning | 210122 | Liaozhong County | 7.6 | 6.3 | 8.8 |  | 88.6(79.0,95.3) | 89.3(79.1,96.3) | 86.6(74.3,94.6) |
| Liaoning | 210604 | Zhen'an District | 10.0 | 8.5 | 11.5 |  | 88.3(80.0,94.5) | 88.8(78.7,96.5) | 86.6(79.7,91.1) |
| Liaoning | 210321 | Tai'an County | 8.8 | 7.5 | 10.2 |  | 88.2(81.0,93.0) | 87.3(79.5,92.5) | 89.5(81.7,94.7) |
| Liaoning | 211421 | Suizhong County | 7.4 | 6.5 | 8.2 |  | 87.5(81.1,92.6) | 83.7(75.6,89.9) | 91.5(83.8,96.1) |
| Liaoning | 211324 | Zizhixian | 8.9 | 7.8 | 10.0 |  | 87.1(79.8,92.2) | 87.0(79.4,92.4) | 85.5(77.1,92.2) |
| Liaoning | 210903 | Xinqiu District | 12.1 | 10.8 | 13.5 |  | 86.8(80.1,91.6) | 87.2(81.0,91.7) | 84.2(75.6,90.2) |
| Liaoning | 211281 | Diaobingshan City | 7.3 | 6.2 | 8.3 |  | 86.3(74.6,94.9) | 84.7(72.4,95.1) | 88.4(74.9,97.8) |
| Liaoning | 210811 | Laobian District | 6.6 | 5.8 | 7.4 |  | 85.8(70.8,96.2) | 82.5(65.8,96.1) | 89.4(72.5,98.5) |
| Liaoning | 210882 | Dashiqiao City | 7.0 | 5.8 | 8.1 |  | 85.8(78.0,90.6) | 85.2(77.2,90.6) | 86.1(76.7,91.9) |
| Liaoning | 211382 | Lingyuan City | 7.9 | 7.2 | 8.5 |  | 85.8(79.1,90.8) | 82.3(74.8,87.9) | 89.8(83.0,94.3) |
| Liaoning | 210726 | Heishan County | 9.3 | 8.0 | 10.6 |  | 85.7(78.9,90.2) | 84.9(78.2,90.0) | 87.4(81.7,91.8) |
| Liaoning | 210381 | Haicheng City | 6.4 | 5.0 | 7.8 |  | 85.1(78.0,90.1) | 83.5(75.8,89.2) | 85.6(76.7,91.4) |
| Liaoning | 210521 | Benxi Manzu Zizhixian | 6.4 | 5.1 | 7.6 |  | 83.8(72.9,91.8) | 88.2(76.5,95.7) | 76.0(63.8,87.5) |
| Liaoning | 210323 | Xiuyan Manzu Zizhixian | 7.8 | 6.0 | 9.5 |  | 83.6(76.4,89.3) | 81.4(73.9,86.7) | 86.3(77.7,92.8) |
| Liaoning | 211302 | Shuangta District | 5.5 | 4.6 | 6.4 |  | 83.6(71.9,90.9) | 82.0(69.7,91.3) | 84.7(70.1,94.6) |
| Liaoning | 211021 | Liaoyang County | 6.9 | 5.5 | 8.2 |  | 83.5(74.5,89.8) | 86.1(76.0,93.1) | 79.0(68.4,87.6) |
| Liaoning | 210202 | Zhongshan District | 8.3 | 7.1 | 9.6 |  | 82.6(71.3,90.9) | 81.7(69.2,91.8) | 80.5(64.4,93.6) |
| Liaoning | 211303 | Longcheng District | 6.8 | 5.9 | 7.6 |  | 81.6(71.0,89.8) | 76.8(68.6,83.4) | 87.8(75.1,96.3) |
| Liaoning | 210911 | Xihe District | 7.1 | 6.0 | 8.2 |  | 81.5(73.7,87.7) | 83.4(76.2,89.1) | 80.8(72.4,87.3) |
| Liaoning | 211481 | Xingcheng City | 6.8 | 6.0 | 7.5 |  | 81.3(72.8,87.8) | 76.1(65.8,84.3) | 86.7(77.2,93.8) |
| Liaoning | 211321 | Chaoyang County | 6.1 | 5.2 | 7.0 |  | 78.4(68.9,85.8) | 80.0(69.8,87.3) | 75.3(64.1,83.9) |
| Liaoning | 210905 | Qinghemen District | 7.3 | 5.8 | 8.8 |  | 77.0(67.9,84.7) | 81.6(72.2,87.6) | 71.5(58.8,82.5) |
| Liaoning | 211403 | Longgang District | 5.5 | 4.7 | 6.2 |  | 76.5(62.0,89.6) | 77.9(60.9,92.3) | 76.8(62.2,90.8) |
| Liaoning | 211404 | Nanpiao District | 8.1 | 6.9 | 9.4 |  | 75.5(67.1,82.5) | 77.8(69.9,84.6) | 74.1(65.2,81.5) |
| Liaoning | 210124 | Faku County | 6.4 | 5.3 | 7.4 |  | 73.6(63.7,81.8) | 72.0(62.2,80.7) | 75.9(65.3,86.1) |
| Liaoning | 211005 | Gongchangling District | 6.9 | 5.3 | 8.6 |  | 71.0(60.9,80.3) | 78.5(69.4,85.5) | 61.7(49.8,72.4) |
| Liaoning | 210881 | Gaizhou City | 5.5 | 4.5 | 6.5 |  | 69.8(59.6,78.9) | 68.1(56.7,77.8) | 71.6(58.6,83.7) |
| Liaoning | 210123 | Kangping County | 5.5 | 4.4 | 6.5 |  | 67.0(52.1,79.7) | 64.7(52.5,77.3) | 69.5(52.6,86.1) |
| Liaoning | 210421 | Fushun County | 4.2 | 3.1 | 5.2 |  | 58.6(39.8,78.6) | 69.6(47.8,90.4) | 40.1(30.9,50.8) |
| Liaoning | 211081 | Dengta City | 4.5 | 3.5 | 5.6 |  | 57.6(46.8,68.7) | 61.3(50.3,73.0) | 55.0(44.0,69.5) |
| Liaoning | 211003 | Wensheng District | 5.7 | 4.7 | 6.8 |  | 55.4(41.8,70.0) | 63.8(47.7,82.8) | 45.3(35.2,56.2) |
| Liaoning | 211422 | Jianchang County | 1.3 | 0.8 | 1.7 |  | 13.4(9.7,18.9) | 14.8(10.3,20.9) | 13.2(8.6,19.4) |
| Jiangsu | 320211 | Binhu District | 8.5 | 6.7 | 11.0 |  | 99.9(99.6,100.0) | 100.0(99.8,100.0) | 99.6(98.1,99.9) |
| Jiangsu | 320111 | Pukou District | 5.8 | 5.4 | 6.1 |  | 99.6(97.9,100.0) | 99.6(97.3,100.0) | 99.2(95.1,99.9) |
| Jiangsu | 320206 | Huishan District | 4.7 | 4.3 | 5.0 |  | 99.5(97.5,99.9) | 99.2(95.8,99.9) | 99.4(96.3,100.0) |
| Jiangsu | 320282 | Yixing City | 7.2 | 6.5 | 7.8 |  | 99.5(98.4,99.9) | 99.1(97.1,99.8) | 99.5(98.0,99.9) |
| Jiangsu | 321112 | Dantu District | 12.9 | 11.9 | 14.0 |  | 99.5(98.4,99.9) | 99.4(97.3,99.9) | 99.4(97.5,99.9) |
| Jiangsu | 320382 | Pizhou City | 8.5 | 7.7 | 9.3 |  | 99.3(98.4,99.7) | 98.9(97.9,99.5) | 99.2(98.2,99.8) |
| Jiangsu | 320115 | Jiangning District | 4.8 | 4.3 | 5.2 |  | 99.2(97.8,99.8) | 99.2(97.3,99.8) | 98.7(95.6,99.7) |
| Jiangsu | 320585 | Taicang City | 6.0 | 5.8 | 6.1 |  | 99.2(96.4,99.9) | 98.0(90.8,99.8) | 99.5(96.5,100.0) |
| Jiangsu | 321003 | Hanjiang District | 5.6 | 4.7 | 6.4 |  | 99.2(96.7,99.9) | 99.3(96.6,99.9) | 98.0(92.6,99.7) |
| Jiangsu | 320411 | Xinbei District | 5.4 | 4.9 | 5.9 |  | 99.1(97.2,99.8) | 98.3(94.3,99.7) | 99.4(97.0,99.9) |
| Jiangsu | 320722 | Donghai County | 7.6 | 6.4 | 8.9 |  | 99.1(98.0,99.6) | 98.9(97.4,99.6) | 98.8(97.2,99.6) |
| Jiangsu | 320205 | Xishan District | 4.9 | 6.5 | 4.1 |  | 98.9(96.4,99.8) | 96.0(89.3,99.1) | 99.8(98.4,100.0) |
| Jiangsu | 320281 | Jiangyin City | 5.8 | 5.2 | 6.3 |  | 98.9(96.8,99.7) | 96.7(92.0,98.8) | 99.5(98.1,99.9) |
| Jiangsu | 320303 | Yunlong District | 6.0 | 5.5 | 6.4 |  | 98.9(96.4,99.8) | 96.7(89.7,99.3) | 99.6(97.3,100.0) |
| Jiangsu | 320113 | Qixia District | 4.0 | 3.5 | 4.4 |  | 98.7(94.2,99.8) | 98.7(92.7,99.9) | 97.8(89.5,99.8) |
| Jiangsu | 320104 | Qinhuai District | 4.9 | 4.3 | 5.4 |  | 98.6(95.1,99.7) | 97.4(90.8,99.5) | 99.0(94.1,99.9) |
| Jiangsu | 320213 | Chong'an District | 6.4 | 5.8 | 6.9 |  | 98.5(95.6,99.6) | 96.9(91.6,99.2) | 99.2(95.8,99.9) |
| Jiangsu | 321084 | Gaoyou City | 9.0 | 8.1 | 10.0 |  | 98.5(96.3,99.6) | 96.2(90.4,99.0) | 99.2(96.6,99.9) |
| Jiangsu | 320311 | Quanshan District | 6.0 | 5.1 | 6.9 |  | 98.4(94.9,99.7) | 97.7(92.3,99.5) | 98.4(92.9,99.8) |
| Jiangsu | 320582 | Zhangjiagang City | 5.8 | 5.2 | 6.3 |  | 98.1(94.6,99.3) | 97.9(93.8,99.4) | 97.2(92.1,99.3) |
| Jiangsu | 321183 | Jurong City | 7.0 | 6.3 | 7.6 |  | 98.1(94.9,99.4) | 97.8(92.8,99.5) | 97.5(92.8,99.4) |
| Jiangsu | 320106 | Nanjinggulou District | 4.9 | 4.4 | 5.5 |  | 97.9(89.0,99.9) | 97.2(86.7,99.8) | 97.8(88.3,99.9) |
| Jiangsu | 320507 | Xiangcheng District | 4.1 | 3.9 | 4.2 |  | 97.8(90.1,99.8) | 94.6(80.5,99.2) | 99.0(93.8,100.0) |
| Jiangsu | 320611 | Gangzha District | 6.3 | 5.8 | 6.8 |  | 97.7(89.4,99.8) | 95.7(81.0,99.7) | 98.2(85.8,99.9) |
| Jiangsu | 320724 | Guannan County | 6.8 | 6.3 | 7.4 |  | 97.4(94.8,98.9) | 96.4(92.5,98.5) | 97.9(95.3,99.3) |
| Jiangsu | 320826 | Lianshui County | 7.7 | 6.7 | 8.6 |  | 97.4(95.2,98.9) | 96.8(93.7,98.5) | 97.5(94.8,99.0) |
| Jiangsu | 320924 | Sheyang County | 8.0 | 7.7 | 8.4 |  | 97.3(94.5,98.8) | 95.4(90.8,97.8) | 98.1(95.6,99.5) |
| Jiangsu | 321012 | Jiangdu District | 9.0 | 7.8 | 10.3 |  | 97.3(94.4,98.9) | 96.2(91.2,98.6) | 97.4(92.6,99.3) |
| Jiangsu | 320482 | Jintan City | 7.8 | 6.3 | 9.4 |  | 97.2(93.5,99.1) | 97.6(93.0,99.4) | 94.8(86.8,98.6) |
| Jiangsu | 321002 | Guangling District | 8.2 | 7.6 | 8.7 |  | 97.2(92.1,99.3) | 88.3(75.9,95.7) | 99.5(96.6,99.9) |
| Jiangsu | 320803 | Chuzhou District | 7.8 | 7.0 | 8.6 |  | 97.1(94.4,98.8) | 96.9(93.8,98.8) | 96.8(93.5,98.7) |
| Jiangsu | 320583 | Kunshan City | 3.3 | 3.2 | 3.4 |  | 97.0(92.6,99.0) | 96.2(89.9,98.9) | 96.8(92.3,99.0) |
| Jiangsu | 320508 | Gusu District | 6.7 | 5.9 | 7.4 |  | 96.9(93.3,98.8) | 95.2(89.8,98.2) | 97.8(94.1,99.3) |
| Jiangsu | 321322 | Shuyang County | 7.6 | 6.9 | 8.3 |  | 96.9(94.7,98.3) | 96.9(94.6,98.3) | 96.5(93.6,98.1) |
| Jiangsu | 321323 | Siyang County | 7.6 | 7.1 | 8.1 |  | 96.9(94.5,98.4) | 95.7(92.4,97.7) | 97.7(95.2,99.0) |
| Jiangsu | 320302 | Gulou District | 5.9 | 5.0 | 6.8 |  | 96.8(92.8,98.9) | 97.5(93.7,99.3) | 94.4(87.3,98.1) |
| Jiangsu | 320305 | Jiawang District | 6.9 | 6.3 | 7.5 |  | 96.8(93.1,98.6) | 97.1(93.4,99.1) | 95.4(90.0,98.2) |
| Jiangsu | 320322 | Pei County | 6.9 | 6.5 | 7.4 |  | 96.8(94.4,98.4) | 94.9(91.1,97.3) | 98.0(95.9,99.2) |
| Jiangsu | 321181 | Danyang City | 6.7 | 6.2 | 7.2 |  | 96.8(93.1,98.8) | 97.8(94.2,99.4) | 93.9(88.1,97.4) |
| Jiangsu | 320324 | Suining County | 8.5 | 7.5 | 9.6 |  | 96.7(94.1,98.2) | 96.4(93.4,98.1) | 96.2(93.1,98.1) |
| Jiangsu | 320723 | Guanyun County | 6.7 | 6.0 | 7.4 |  | 96.7(94.2,98.5) | 95.1(91.1,97.6) | 97.6(95.0,99.1) |
| Jiangsu | 320481 | Liyang City | 7.6 | 6.7 | 8.4 |  | 96.6(92.6,98.6) | 97.9(94.8,99.4) | 91.5(84.2,96.0) |
| Jiangsu | 320706 | Haizhou District | 4.6 | 4.1 | 5.1 |  | 96.6(91.9,98.9) | 95.2(88.8,98.5) | 96.9(91.3,99.2) |
| Jiangsu | 320902 | Tinghu District | 6.1 | 5.6 | 6.5 |  | 96.6(92.7,98.6) | 94.7(89.4,97.8) | 97.7(94.0,99.4) |
| Jiangsu | 320922 | Binhai County | 7.7 | 7.0 | 8.4 |  | 96.6(93.9,98.1) | 95.8(92.6,97.7) | 96.8(93.9,98.5) |
| Jiangsu | 320682 | Rugao City | 10.4 | 9.0 | 11.8 |  | 96.5(93.3,98.4) | 94.9(90.2,97.6) | 96.7(92.8,98.8) |
| Jiangsu | 321023 | Baoying County | 9.2 | 8.2 | 10.3 |  | 96.5(93.2,98.5) | 94.7(89.2,97.9) | 97.4(93.4,99.3) |
| Jiangsu | 320105 | Jianye District | 3.7 | 3.4 | 4.0 |  | 96.4(88.9,99.2) | 97.7(88.4,99.8) | 90.7(79.3,96.8) |
| Jiangsu | 320381 | Xinyi City | 7.8 | 6.8 | 8.7 |  | 96.4(93.8,98.2) | 96.6(93.8,98.3) | 95.6(91.9,97.6) |
| Jiangsu | 320506 | Wuzhong District | 4.0 | 3.8 | 4.3 |  | 96.4(91.8,98.9) | 93.0(84.0,97.8) | 98.2(94.4,99.6) |
| Jiangsu | 320923 | Funing County | 8.6 | 7.7 | 9.5 |  | 96.4(93.1,98.2) | 94.8(90.8,97.4) | 97.1(93.9,98.9) |
| Jiangsu | 320312 | Tongshan District | 6.3 | 5.5 | 7.1 |  | 96.3(93.4,98.1) | 94.8(91.0,97.2) | 97.4(94.3,99.0) |
| Jiangsu | 320581 | Changshu City | 6.1 | 5.8 | 6.4 |  | 96.1(91.3,98.5) | 95.9(89.5,98.9) | 94.8(88.6,98.5) |
| Jiangsu | 321081 | Yizheng City | 7.4 | 6.6 | 8.2 |  | 96.1(90.1,98.7) | 89.1(81.3,94.7) | 98.8(94.5,99.9) |
| Jiangsu | 321324 | Sihong County | 7.6 | 6.9 | 8.3 |  | 96.1(93.1,97.9) | 94.4(90.7,97.0) | 97.2(94.5,98.7) |
| Jiangsu | 320621 | Hai'an County | 10.3 | 8.7 | 12.0 |  | 95.9(91.2,98.4) | 95.6(89.0,98.6) | 94.8(86.9,98.3) |
| Jiangsu | 320114 | Yuhuatai District | 3.8 | 3.5 | 4.1 |  | 95.8(85.3,99.2) | 96.5(84.6,99.5) | 92.3(76.8,98.9) |
| Jiangsu | 321102 | Jingkou District | 5.1 | 4.7 | 5.5 |  | 95.8(86.7,99.1) | 97.0(86.4,99.7) | 92.3(82.0,98.2) |
| Jiangsu | 320102 | Xuanwu District | 4.2 | 3.8 | 4.5 |  | 95.5(87.3,99.0) | 95.4(83.9,99.3) | 93.9(78.4,99.3) |
| Jiangsu | 320981 | Dongtai City | 10.4 | 9.3 | 11.6 |  | 95.4(91.0,97.9) | 93.6(88.3,97.3) | 95.6(89.5,98.4) |
| Jiangsu | 320509 | Wujiang District | 4.1 | 3.8 | 4.4 |  | 95.2(90.3,98.1) | 93.6(87.2,97.7) | 95.6(89.7,98.5) |
| Jiangsu | 320402 | Tianning District | 5.4 | 4.9 | 6.0 |  | 95.0(88.5,98.3) | 94.0(85.1,98.4) | 95.7(87.6,99.0) |
| Jiangsu | 320721 | Ganyu County | 7.3 | 6.2 | 8.5 |  | 94.9(91.5,97.1) | 93.8(89.8,96.4) | 95.7(92.1,97.9) |
| Jiangsu | 321202 | Hailing District | 5.4 | 4.9 | 5.9 |  | 94.8(85.6,98.6) | 93.1(81.5,98.6) | 95.4(84.4,99.3) |
| Jiangsu | 320903 | Yandu District | 7.2 | 6.5 | 7.8 |  | 94.5(89.9,97.4) | 91.0(83.3,95.9) | 96.5(91.9,99.0) |
| Jiangsu | 320982 | Dafeng City | 8.2 | 7.6 | 8.9 |  | 94.2(88.7,97.3) | 94.6(88.2,98.1) | 91.9(83.7,96.8) |
| Jiangsu | 321111 | Runzhou District | 5.6 | 5.1 | 6.2 |  | 94.1(83.4,98.8) | 93.5(78.9,99.0) | 93.9(79.8,99.3) |
| Jiangsu | 320681 | Qidong City | 10.8 | 9.3 | 12.5 |  | 94.0(89.4,96.9) | 94.4(88.2,97.7) | 91.1(85.1,95.3) |
| Jiangsu | 320684 | Haimen City | 9.7 | 8.4 | 11.4 |  | 94.0(89.0,97.3) | 88.7(80.7,94.1) | 96.3(90.7,98.9) |
| Jiangsu | 320921 | Xiangshui County | 7.0 | 6.5 | 7.5 |  | 94.0(90.1,96.8) | 93.0(87.9,96.6) | 94.7(89.8,97.4) |
| Jiangsu | 320602 | Chongchuan District | 4.8 | 4.2 | 5.4 |  | 93.8(85.1,97.9) | 90.6(78.8,97.4) | 95.8(86.7,99.3) |
| Jiangsu | 320116 | Liuhe District | 5.5 | 5.1 | 6.0 |  | 93.6(88.2,97.2) | 93.6(87.6,97.6) | 91.7(84.4,96.2) |
| Jiangsu | 320703 | Lianyun District | 5.1 | 4.6 | 5.7 |  | 93.4(80.9,98.7) | 94.4(80.6,99.5) | 90.6(77.4,98.3) |
| Jiangsu | 320830 | Xuyi County | 7.3 | 6.6 | 8.1 |  | 93.3(88.5,96.2) | 92.7(87.3,96.3) | 93.6(87.9,96.7) |
| Jiangsu | 321203 | Gaogang District | 9.2 | 8.3 | 10.0 |  | 93.0(86.4,97.3) | 94.3(84.7,98.5) | 89.7(84.3,94.0) |
| Jiangsu | 320404 | Zhonglou District | 4.8 | 4.4 | 5.2 |  | 92.9(83.8,97.5) | 90.3(77.5,97.5) | 94.4(84.7,98.9) |
| Jiangsu | 321182 | Yangzhong City | 6.8 | 6.0 | 7.5 |  | 92.9(83.6,97.6) | 95.9(87.0,99.2) | 83.0(74.8,88.8) |
| Jiangsu | 321302 | Sucheng District | 6.9 | 6.3 | 7.5 |  | 92.9(82.0,98.0) | 90.6(78.8,97.2) | 94.8(84.3,98.7) |
| Jiangsu | 321284 | Jiangyan City | 8.9 | 7.6 | 10.3 |  | 92.7(85.4,97.1) | 91.2(81.9,96.4) | 91.9(81.8,97.6) |
| Jiangsu | 320505 | Huqiu District | 3.7 | 3.3 | 4.0 |  | 92.6(84.4,97.4) | 93.6(83.8,98.4) | 90.5(79.9,97.0) |
| Jiangsu | 320321 | Feng County | 7.6 | 6.9 | 8.3 |  | 92.3(88.1,95.3) | 91.7(86.8,95.0) | 92.8(87.7,96.1) |
| Jiangsu | 321283 | Taixing City | 9.0 | 7.7 | 10.4 |  | 91.8(86.0,95.5) | 92.0(85.9,96.0) | 89.4(82.0,94.4) |
| Jiangsu | 320623 | Rudong County | 10.2 | 8.8 | 11.8 |  | 91.7(85.0,96.0) | 91.6(83.0,96.9) | 88.4(78.6,94.8) |
| Jiangsu | 320412 | Wujin District | 5.0 | 4.7 | 5.3 |  | 91.3(85.1,95.5) | 86.8(77.8,92.8) | 94.2(87.9,97.8) |
| Jiangsu | 320804 | Huaiyin District | 7.2 | 6.3 | 8.2 |  | 91.3(86.5,94.7) | 90.1(84.5,94.0) | 92.5(87.0,96.0) |
| Jiangsu | 321281 | Xinghua City | 9.4 | 8.5 | 10.4 |  | 91.0(86.1,94.6) | 89.8(83.9,93.8) | 90.5(84.0,94.7) |
| Jiangsu | 320829 | Hongze County | 6.9 | 6.3 | 7.5 |  | 90.4(81.9,96.1) | 87.0(74.4,95.1) | 92.8(82.3,98.1) |
| Jiangsu | 320124 | Lishui County | 6.4 | 5.4 | 7.3 |  | 90.3(82.2,95.8) | 90.8(81.7,96.5) | 87.8(76.2,95.5) |
| Jiangsu | 320831 | Jinhu County | 7.4 | 6.5 | 8.3 |  | 89.7(78.4,96.1) | 88.0(74.7,95.8) | 90.2(75.2,97.5) |
| Jiangsu | 320612 | Tongzhou District | 10.0 | 8.6 | 11.5 |  | 89.2(82.8,93.6) | 88.7(81.4,94.1) | 87.4(79.0,92.7) |
| Jiangsu | 320125 | Gaochun County | 7.4 | 6.3 | 8.4 |  | 88.6(79.3,94.3) | 89.3(78.0,96.3) | 83.7(72.3,91.6) |
| Jiangsu | 320925 | Jianhu County | 7.2 | 6.4 | 8.1 |  | 88.1(80.8,93.2) | 84.8(76.1,91.8) | 90.4(81.9,95.5) |
| Jiangsu | 321282 | Jingjiang City | 7.4 | 6.9 | 7.8 |  | 87.8(79.5,93.2) | 84.9(75.0,92.2) | 89.8(81.2,95.6) |
| Jiangsu | 321311 | Suyu District | 5.4 | 4.8 | 6.0 |  | 83.8(76.1,89.6) | 80.6(72.1,87.4) | 87.4(78.7,93.2) |
| Jiangsu | 320811 | Qingpu District | 3.9 | 3.5 | 4.2 |  | 77.5(55.0,93.5) | 78.0(54.7,94.4) | 76.9(56.4,93.1) |
| Jiangsu | 320802 | Qinghe District | 1.8 | 1.6 | 2.0 |  | 44.9(29.0,63.8) | 38.4(24.8,59.8) | 52.8(31.0,79.5) |
| Fujian | 350211 | Jimei District | 1.7 | 1.5 | 1.9 |  | 99.3(95.4,100.0) | 98.1(90.1,99.9) | 99.6(95.8,100.0) |
| Fujian | 350205 | Haicang District | 2.2 | 1.9 | 2.4 |  | 99.0(95.7,99.9) | 99.4(95.6,100.0) | 97.2(88.6,99.7) |
| Fujian | 350502 | Licheng District | 4.5 | 3.9 | 5.1 |  | 98.5(94.7,99.7) | 98.8(94.7,99.8) | 97.2(91.2,99.5) |
| Fujian | 350505 | Quangang District | 8.0 | 6.3 | 9.9 |  | 98.5(96.4,99.4) | 98.7(96.4,99.6) | 97.7(93.7,99.4) |
| Fujian | 350504 | Luojiang District | 6.1 | 4.8 | 7.4 |  | 96.8(91.8,99.1) | 98.1(93.7,99.7) | 93.6(84.5,98.2) |
| Fujian | 350125 | Yongtai County | 7.9 | 6.5 | 9.3 |  | 96.7(93.0,98.6) | 94.5(89.0,97.7) | 97.9(94.2,99.5) |
| Fujian | 350825 | Liancheng County | 8.2 | 6.9 | 9.5 |  | 96.4(92.3,98.5) | 97.1(92.7,99.1) | 93.4(86.3,97.4) |
| Fujian | 350304 | Licheng District | 6.8 | 5.8 | 7.8 |  | 96.2(92.6,98.1) | 96.3(93.0,98.3) | 95.3(90.8,97.8) |
| Fujian | 350822 | Yongding County | 7.9 | 6.7 | 9.1 |  | 95.3(91.1,97.4) | 95.1(91.0,97.5) | 94.8(89.9,97.7) |
| Fujian | 350105 | Mawei District | 3.8 | 3.5 | 4.2 |  | 95.2(82.1,99.3) | 97.8(86.2,99.8) | 87.0(75.0,96.0) |
| Fujian | 350182 | Changle City | 5.3 | 4.5 | 6.1 |  | 95.2(90.9,97.7) | 93.2(87.2,96.9) | 96.8(92.2,99.0) |
| Fujian | 350121 | Minhou County | 5.7 | 5.1 | 6.3 |  | 95.1(90.6,97.5) | 94.5(88.9,97.6) | 95.1(90.0,98.0) |
| Fujian | 350203 | Siming District | 3.4 | 2.6 | 4.2 |  | 95.0(86.4,98.6) | 96.2(88.5,99.2) | 91.7(79.9,97.6) |
| Fujian | 350824 | Wuping County | 7.6 | 6.5 | 8.8 |  | 95.0(90.2,98.0) | 93.7(88.2,97.4) | 95.6(89.1,98.7) |
| Fujian | 350213 | Xiang'an District | 4.9 | 4.6 | 5.2 |  | 94.9(86.4,98.9) | 92.5(82.2,98.2) | 96.7(88.6,99.5) |
| Fujian | 350925 | Zhouning County | 7.3 | 4.9 | 9.5 |  | 94.8(87.8,98.3) | 92.4(83.0,97.4) | 95.8(84.8,99.5) |
| Fujian | 350104 | Cangshan District | 3.8 | 3.3 | 4.2 |  | 94.6(85.9,98.7) | 95.2(85.2,99.0) | 93.6(83.8,98.4) |
| Fujian | 350628 | Pinghe County | 6.6 | 6.2 | 7.1 |  | 94.6(90.4,97.2) | 94.1(89.1,97.3) | 94.6(89.7,97.3) |
| Fujian | 350181 | Fuqing City | 5.3 | 4.6 | 6.0 |  | 94.4(90.6,96.8) | 94.6(90.4,97.2) | 94.1(89.9,97.0) |
| Fujian | 350124 | Minqing County | 7.3 | 6.1 | 8.4 |  | 94.3(88.7,97.5) | 93.4(86.1,97.2) | 94.9(88.7,97.9) |
| Fujian | 350926 | Zherong County | 6.3 | 3.9 | 8.5 |  | 94.2(84.4,98.5) | 96.9(87.0,99.6) | 83.0(68.3,94.6) |
| Fujian | 350626 | Dongshan County | 6.3 | 5.8 | 6.7 |  | 93.6(86.0,97.8) | 95.5(87.9,98.8) | 89.8(80.8,95.6) |
| Fujian | 350681 | Longhai City | 5.3 | 4.7 | 5.9 |  | 93.4(88.8,96.3) | 92.7(87.2,96.0) | 93.7(88.8,96.8) |
| Fujian | 350823 | Shanghang County | 7.9 | 7.1 | 8.7 |  | 93.2(88.6,96.2) | 94.0(89.0,97.1) | 91.2(84.9,95.5) |
| Fujian | 350122 | Lianjiang County | 6.3 | 5.6 | 7.0 |  | 93.0(88.0,96.2) | 90.9(85.0,95.2) | 94.9(89.5,97.6) |
| Fujian | 350303 | Hanjiang District | 5.9 | 5.4 | 6.4 |  | 92.8(87.4,96.5) | 92.0(85.2,96.5) | 93.1(86.1,96.8) |
| Fujian | 350725 | Zhenghe County | 6.7 | 5.4 | 7.9 |  | 92.7(87.2,96.7) | 90.2(82.0,95.4) | 94.5(87.3,98.3) |
| Fujian | 350526 | Dehua County | 5.0 | 4.3 | 5.6 |  | 92.5(85.4,96.8) | 92.4(82.9,97.3) | 91.0(80.0,96.9) |
| Fujian | 350623 | Zhangpu County | 5.6 | 5.0 | 6.2 |  | 92.5(87.5,95.8) | 94.0(89.4,97.0) | 88.9(82.5,93.6) |
| Fujian | 350923 | Pingnan County | 8.0 | 6.5 | 9.3 |  | 92.3(85.7,96.1) | 89.8(81.5,95.4) | 94.6(87.6,97.9) |
| Fujian | 350622 | Yunxiao County | 5.8 | 5.4 | 6.2 |  | 92.2(86.0,96.2) | 88.4(79.7,93.9) | 95.2(89.4,98.3) |
| Fujian | 350426 | Youxi County | 6.6 | 5.4 | 7.8 |  | 92.1(86.5,95.7) | 92.8(87.3,96.5) | 89.6(82.2,94.7) |
| Fujian | 350102 | Gulou District | 3.9 | 3.2 | 4.5 |  | 91.8(78.1,98.3) | 95.2(83.9,99.3) | 83.1(67.0,94.7) |
| Fujian | 350625 | Changtai County | 5.5 | 5.1 | 6.0 |  | 91.6(83.2,96.5) | 91.0(80.9,96.8) | 92.4(83.3,97.4) |
| Fujian | 350212 | Tongan District | 3.3 | 2.9 | 3.7 |  | 91.4(83.6,96.1) | 92.9(84.7,97.5) | 88.1(78.2,94.4) |
| Fujian | 350921 | Xiapu County | 6.0 | 4.8 | 7.2 |  | 91.2(85.9,95.0) | 88.2(81.6,93.3) | 93.4(87.4,96.8) |
| Fujian | 350902 | Jiaocheng District | 5.7 | 4.4 | 6.9 |  | 91.1(85.2,94.9) | 91.7(85.5,95.8) | 90.7(83.4,95.4) |
| Fujian | 350429 | Taining County | 6.2 | 5.1 | 7.2 |  | 90.8(79.8,96.7) | 85.8(74.5,94.6) | 94.3(80.9,99.1) |
| Fujian | 350524 | Anxi County | 5.4 | 4.4 | 6.2 |  | 90.7(85.8,94.1) | 89.4(84.4,93.1) | 93.0(88.2,96.3) |
| Fujian | 350982 | Fuding City | 6.4 | 5.1 | 7.6 |  | 90.7(85.5,94.8) | 92.4(86.8,96.1) | 88.4(81.2,93.4) |
| Fujian | 350603 | Longwen District | 3.9 | 3.5 | 4.4 |  | 90.4(79.8,96.7) | 76.6(64.2,87.1) | 97.2(88.6,99.6) |
| Fujian | 350783 | Jian'ou City | 7.2 | 6.2 | 8.2 |  | 90.4(84.7,94.2) | 89.5(83.4,93.6) | 91.5(85.9,95.3) |
| Fujian | 350781 | Shaowu City | 7.0 | 6.0 | 8.0 |  | 90.2(83.6,95.0) | 87.3(78.6,93.2) | 92.6(84.5,96.9) |
| Fujian | 350629 | Hua'an County | 6.2 | 5.0 | 7.3 |  | 90.1(81.7,95.9) | 88.8(80.2,95.3) | 91.3(79.2,97.1) |
| Fujian | 350627 | Nanjing County | 6.2 | 5.3 | 7.0 |  | 89.9(83.2,94.5) | 91.4(83.7,95.7) | 88.0(79.1,94.3) |
| Fujian | 350922 | Gutian County | 7.1 | 5.9 | 8.2 |  | 89.7(83.7,94.1) | 88.1(80.3,93.7) | 91.2(84.5,95.6) |
| Fujian | 350123 | Luoyuan County | 6.4 | 5.3 | 7.4 |  | 89.6(81.3,95.1) | 88.1(78.0,95.0) | 90.6(80.0,96.6) |
| Fujian | 350582 | Jinjiang City | 3.2 | 2.8 | 3.6 |  | 89.5(83.0,93.9) | 88.7(81.8,93.1) | 91.0(84.6,94.9) |
| Fujian | 350782 | Wiyishan City | 5.1 | 4.1 | 5.9 |  | 89.5(80.7,95.6) | 87.9(75.8,95.2) | 90.3(78.8,96.4) |
| Fujian | 350322 | Xianyou County | 6.6 | 5.7 | 7.6 |  | 89.4(84.0,93.0) | 87.3(81.7,91.7) | 92.4(87.3,95.7) |
| Fujian | 350402 | Meilie District | 4.4 | 3.2 | 5.6 |  | 89.3(71.6,97.5) | 95.4(81.5,99.6) | 68.2(50.4,87.2) |
| Fujian | 350427 | Sha County | 6.7 | 5.7 | 7.6 |  | 89.3(82.6,94.4) | 88.8(79.9,94.7) | 89.3(80.4,95.4) |
| Fujian | 350423 | Qingliu County | 6.5 | 5.6 | 7.3 |  | 88.9(79.0,94.9) | 91.8(82.2,97.4) | 80.2(67.0,90.4) |
| Fujian | 350421 | Mingxi County | 6.7 | 5.6 | 7.8 |  | 88.6(77.2,96.0) | 87.1(73.4,95.9) | 88.2(72.7,96.7) |
| Fujian | 350302 | Chengxiang District | 5.2 | 4.3 | 6.2 |  | 88.5(81.1,93.4) | 90.1(82.7,95.2) | 86.8(77.4,93.5) |
| Fujian | 350428 | Jiangle County | 6.6 | 5.8 | 7.3 |  | 88.4(80.0,94.3) | 86.1(74.6,93.2) | 90.4(80.9,96.5) |
| Fujian | 350821 | Changting County | 6.5 | 5.4 | 7.6 |  | 88.4(82.0,93.1) | 88.7(82.2,93.4) | 87.8(80.2,93.6) |
| Fujian | 350503 | Fengze District | 3.0 | 2.5 | 3.4 |  | 88.3(79.3,94.7) | 88.8(77.6,95.4) | 88.3(77.3,95.2) |
| Fujian | 350602 | Xiangcheng District | 4.5 | 3.8 | 5.2 |  | 88.3(80.7,93.9) | 88.7(78.9,94.8) | 87.6(78.2,94.2) |
| Fujian | 350924 | Shouning County | 6.7 | 5.3 | 8.0 |  | 88.3(80.3,93.9) | 90.5(81.1,96.4) | 84.4(74.0,91.3) |
| Fujian | 350802 | Xinluo District | 4.0 | 3.6 | 4.3 |  | 87.8(80.0,93.3) | 87.7(79.0,93.8) | 87.5(78.6,93.5) |
| Fujian | 350721 | Shunchang County | 7.3 | 6.0 | 8.6 |  | 87.5(79.9,93.4) | 90.3(81.9,95.7) | 83.0(72.3,90.5) |
| Fujian | 350702 | Yanping District | 6.2 | 5.1 | 7.2 |  | 87.2(77.3,94.2) | 89.9(80.5,95.9) | 84.0(73.2,92.5) |
| Fujian | 350430 | Jianning County | 6.8 | 5.6 | 7.9 |  | 87.0(77.6,92.8) | 84.1(74.3,90.8) | 89.2(78.1,96.6) |
| Fujian | 350424 | Ninghua County | 7.0 | 5.8 | 8.3 |  | 86.8(79.9,92.1) | 88.4(81.8,93.4) | 83.9(74.7,90.4) |
| Fujian | 350111 | Jin'an District | 3.1 | 2.8 | 3.4 |  | 86.5(73.1,94.9) | 85.6(70.0,94.8) | 88.4(76.9,95.9) |
| Fujian | 350881 | Zhangping City | 6.1 | 5.2 | 6.9 |  | 86.4(77.5,92.3) | 86.2(76.1,92.8) | 87.2(76.7,93.7) |
| Fujian | 350425 | Datian County | 6.2 | 5.2 | 7.1 |  | 86.3(78.5,91.8) | 83.5(75.0,90.4) | 89.1(79.5,94.9) |
| Fujian | 350581 | Shishi City | 2.8 | 2.3 | 3.1 |  | 86.2(71.3,95.9) | 86.1(70.5,95.9) | 87.3(72.9,95.5) |
| Fujian | 350724 | Songxi County | 7.0 | 5.7 | 8.3 |  | 86.2(74.1,93.9) | 89.1(77.1,96.2) | 81.7(69.9,90.8) |
| Fujian | 350583 | Nan'an City | 3.7 | 3.0 | 4.3 |  | 85.7(79.0,91.0) | 83.4(76.7,89.0) | 88.7(81.5,94.0) |
| Fujian | 350722 | Pucheng County | 8.1 | 6.6 | 9.5 |  | 85.1(77.3,90.6) | 85.4(77.4,91.3) | 85.7(77.2,91.2) |
| Fujian | 350103 | Taijiang District | 4.4 | 4.0 | 4.8 |  | 84.4(72.5,93.7) | 82.8(68.0,94.0) | 85.7(72.3,95.0) |
| Fujian | 350723 | Guangze County | 6.3 | 5.2 | 7.3 |  | 82.9(72.5,90.6) | 86.4(74.5,94.0) | 78.1(66.2,87.6) |
| Fujian | 350784 | Jianyang City | 6.3 | 5.5 | 7.1 |  | 82.1(73.9,88.6) | 81.1(71.0,87.8) | 84.4(76.6,90.9) |
| Fujian | 350481 | Yong'an City | 5.6 | 4.7 | 6.4 |  | 79.9(70.7,87.2) | 79.3(69.5,87.1) | 80.8(70.4,89.2) |
| Fujian | 350525 | Yongchun County | 4.4 | 3.8 | 5.0 |  | 79.2(70.5,86.2) | 77.0(67.4,85.4) | 83.0(74.1,90.9) |
| Fujian | 350206 | Huli District | 1.1 | 0.9 | 1.3 |  | 78.8(60.2,92.4) | 79.6(62.4,93.1) | 81.3(64.3,92.9) |
| Fujian | 350403 | Sanyuan District | 4.0 | 2.9 | 5.1 |  | 75.4(56.7,91.4) | 77.7(58.8,93.1) | 71.3(47.3,91.9) |
| Fujian | 350624 | Zhao'an County | 3.6 | 3.3 | 3.9 |  | 75.1(59.3,87.9) | 73.3(58.2,86.2) | 76.7(62.7,89.0) |
| Fujian | 350521 | Hui'an County | 4.6 | 3.7 | 5.5 |  | 64.7(55.4,73.9) | 76.3(67.9,82.6) | 49.1(38.8,60.6) |
| Fujian | 350305 | Xiuyu District | 2.3 | 1.8 | 2.9 |  | 51.3(39.5,64.3) | 57.8(44.8,71.6) | 46.1(33.2,59.9) |
| Fujian | 350981 | Fu'an City | 1.3 | 0.8 | 1.7 |  | 27.3(19.9,35.9) | 26.7(20.3,35.6) | 33.1(23.7,43.6) |
| Shanghai | 310101 | Huangpu District | 13.2 | 12.1 | 14.3 |  | 99.9(99.6,100.0) | 100.0(99.7,100.0) | 99.4(97.4,99.9) |
| Shanghai | 310110 | Yangpu District | 8.0 | 7.2 | 8.8 |  | 99.7(99.2,99.9) | 99.8(99.1,100.0) | 99.1(97.4,99.8) |
| Shanghai | 310112 | Minxing District | 3.7 | 3.4 | 4.1 |  | 97.8(95.8,98.9) | 97.3(94.8,98.7) | 96.9(93.8,98.6) |
| Shanghai | 310105 | Changning District | 8.0 | 7.1 | 9.1 |  | 96.9(92.3,99.0) | 97.1(91.2,99.4) | 95.3(86.5,99.0) |
| Shanghai | 310107 | Putuo District | 6.9 | 6.3 | 7.5 |  | 96.5(93.7,98.3) | 96.1(92.4,98.3) | 94.5(89.7,97.7) |
| Shanghai | 310104 | Xuhui District | 7.9 | 6.9 | 8.8 |  | 95.5(91.5,98.0) | 93.6(87.7,97.0) | 95.7(90.0,98.6) |
| Shanghai | 310109 | Hongkou District | 8.1 | 7.5 | 8.7 |  | 94.3(89.5,97.2) | 91.4(83.9,95.9) | 95.9(90.1,98.7) |
| Shanghai | 310106 | Jingan District | 8.7 | 8.0 | 9.6 |  | 94.1(89.5,96.7) | 92.8(87.3,96.1) | 94.2(87.9,97.6) |
| Shanghai | 310115 | Pudongxin District | 4.7 | 4.3 | 5.1 |  | 83.4(79.2,86.9) | 80.7(75.8,84.8) | 85.4(80.8,88.9) |
| Shanghai | 310114 | Jiading District | 3.6 | 3.6 | 3.7 |  | 77.5(69.9,83.7) | 73.5(64.7,81.8) | 81.4(72.0,88.4) |
| Shanghai | 310116 | Jinshan District | 5.7 | 5.2 | 6.2 |  | 76.3(66.6,83.8) | 71.2(59.3,80.7) | 79.2(67.4,89.2) |
| Shanghai | 310113 | Baoshan District | 4.3 | 4.1 | 4.6 |  | 72.5(64.9,78.8) | 74.2(65.7,81.2) | 71.8(63.1,78.6) |
| Shanghai | 310120 | Fengxian District | 4.0 | 3.8 | 4.2 |  | 69.3(60.5,76.6) | 65.1(55.2,74.1) | 75.4(65.4,84.1) |
| Shanghai | 310117 | Songjiang District | 3.1 | 2.8 | 3.4 |  | 67.9(59.8,74.9) | 64.1(54.7,72.6) | 74.7(65.3,81.7) |
| Shanghai | 310118 | Qingpu District | 3.4 | 3.3 | 3.5 |  | 57.1(48.7,65.2) | 51.1(42.6,58.9) | 67.6(57.3,76.5) |
| Shanghai | 310230 | Chongming County | 9.2 | 8.6 | 9.7 |  | 52.0(39.5,64.9) | 49.6(36.8,62.2) | 47.4(34.4,61.4) |
| Guangdong | 440802 | Chikan District | 3.6 | 2.9 | 4.3 |  | 99.9(99.5,100.0) | 99.9(98.7,100.0) | 99.9(99.0,100.0) |
| Guangdong | 441303 | Huiyang District | 4.8 | 4.5 | 5.0 |  | 99.9(99.5,100.0) | 99.8(98.9,100.0) | 99.9(99.1,100.0) |
| Guangdong | 440115 | Nansha District | 8.5 | 8.3 | 8.8 |  | 99.8(98.5,100.0) | 99.7(97.5,100.0) | 99.8(98.1,100.0) |
| Guangdong | 442000 | Zhongshan City | 3.5 | 3.0 | 3.9 |  | 99.5(98.6,99.9) | 99.3(98.1,99.8) | 99.4(98.2,99.9) |
| Guangdong | 445202 | Rongcheng District | 7.2 | 6.6 | 7.7 |  | 99.1(97.5,99.7) | 99.1(97.3,99.8) | 98.9(96.9,99.7) |
| Guangdong | 440306 | Baoan District | 0.7 | 0.6 | 0.8 |  | 98.7(96.4,99.6) | 98.1(94.9,99.4) | 98.6(96.2,99.6) |
| Guangdong | 441702 | Jiangcheng District | 5.6 | 5.1 | 6.1 |  | 98.4(95.9,99.5) | 98.2(94.8,99.5) | 98.0(93.9,99.5) |
| Guangdong | 440511 | Jinping District | 6.6 | 6.1 | 7.1 |  | 98.3(96.0,99.4) | 98.2(95.2,99.5) | 98.0(94.5,99.4) |
| Guangdong | 440923 | Dianbai County | 2.7 | 2.0 | 3.4 |  | 98.3(95.1,99.5) | 92.2(84.9,96.6) | 99.6(98.0,99.9) |
| Guangdong | 440114 | Huadu District | 4.2 | 3.8 | 4.5 |  | 98.1(95.5,99.4) | 98.3(95.5,99.5) | 96.2(91.4,98.8) |
| Guangdong | 441203 | Dinghu District | 6.6 | 6.1 | 7.0 |  | 98.1(93.2,99.7) | 98.4(92.1,99.8) | 96.2(85.4,99.6) |
| Guangdong | 440604 | Chancheng District | 3.8 | 3.4 | 4.2 |  | 98.0(94.7,99.4) | 97.5(93.2,99.3) | 97.5(93.1,99.5) |
| Guangdong | 440104 | Yuexiu District | 5.4 | 4.8 | 5.9 |  | 97.8(91.0,99.8) | 98.0(91.5,99.8) | 96.1(86.7,99.6) |
| Guangdong | 441223 | Guangning County | 9.0 | 7.8 | 10.1 |  | 97.8(95.9,99.0) | 97.7(95.2,99.0) | 97.1(93.7,99.0) |
| Guangdong | 445102 | Xiangqiao District | 6.7 | 6.3 | 7.2 |  | 97.8(94.7,99.3) | 95.9(90.2,98.8) | 98.8(96.1,99.7) |
| Guangdong | 440783 | Kaiping City | 7.6 | 7.2 | 8.1 |  | 97.6(95.3,99.0) | 97.4(94.4,99.0) | 97.2(94.0,98.9) |
| Guangdong | 445302 | Yuncheng District | 6.0 | 5.3 | 6.7 |  | 97.5(93.9,99.3) | 94.8(88.0,98.3) | 98.7(94.8,99.8) |
| Guangdong | 440608 | Gaoming District | 4.5 | 4.2 | 4.7 |  | 97.4(92.4,99.4) | 96.8(89.8,99.5) | 97.1(89.0,99.6) |
| Guangdong | 440803 | Xiashan District | 3.2 | 2.6 | 3.7 |  | 97.2(91.0,99.4) | 94.1(83.3,98.9) | 98.4(90.7,99.9) |
| Guangdong | 441424 | Wuhua County | 6.2 | 5.3 | 7.0 |  | 97.2(94.6,98.7) | 96.9(94.4,98.7) | 96.4(93.2,98.4) |
| Guangdong | 441284 | Sihui City | 5.5 | 5.3 | 5.6 |  | 97.0(93.3,98.9) | 95.7(89.7,98.5) | 97.3(92.1,99.3) |
| Guangdong | 440105 | Haizhu District | 4.6 | 4.1 | 5.0 |  | 96.9(86.6,99.6) | 97.5(88.1,99.8) | 93.7(79.7,99.0) |
| Guangdong | 440507 | Longhu District | 4.4 | 4.0 | 4.8 |  | 96.9(92.8,99.0) | 96.3(89.6,99.0) | 97.4(92.0,99.4) |
| Guangdong | 441900 | Dongguan City | 1.7 | 1.5 | 1.9 |  | 96.8(93.7,98.7) | 95.2(90.8,97.9) | 97.4(94.4,99.0) |
| Guangdong | 440883 | Wuchuan City | 5.4 | 4.6 | 6.2 |  | 96.4(93.0,98.4) | 95.8(91.4,98.3) | 96.4(92.0,98.6) |
| Guangdong | 441322 | Boluo County | 5.0 | 4.4 | 5.5 |  | 96.4(92.5,98.6) | 96.6(92.6,98.9) | 95.0(89.4,98.2) |
| Guangdong | 440902 | Maonan District | 3.1 | 2.3 | 3.8 |  | 96.3(91.4,98.7) | 96.8(91.8,99.2) | 92.7(83.6,97.7) |
| Guangdong | 441421 | Mei County | 7.6 | 6.9 | 8.2 |  | 96.3(92.5,98.3) | 94.5(89.6,97.4) | 97.2(93.3,99.1) |
| Guangdong | 441823 | Yangshan County | 10.2 | 8.5 | 11.9 |  | 96.2(93.4,98.1) | 95.9(92.7,97.9) | 94.8(89.9,97.4) |
| Guangdong | 440705 | Xinhui District | 6.7 | 6.2 | 7.2 |  | 96.1(92.6,98.2) | 95.4(91.0,97.9) | 96.1(92.0,98.2) |
| Guangdong | 440103 | Liwan District | 6.6 | 6.2 | 7.1 |  | 96.0(91.4,98.4) | 94.8(89.2,98.2) | 96.3(90.2,98.9) |
| Guangdong | 440113 | Panyu District | 3.0 | 2.4 | 3.5 |  | 96.0(91.8,98.3) | 95.6(91.0,98.4) | 95.0(89.2,98.3) |
| Guangdong | 440704 | Jianghai District | 4.5 | 4.2 | 4.8 |  | 96.0(84.0,99.6) | 94.4(78.8,99.5) | 96.4(84.2,99.8) |
| Guangdong | 440606 | Shunde District | 3.3 | 3.1 | 3.4 |  | 95.8(91.8,98.2) | 95.3(90.8,98.2) | 95.3(90.8,98.2) |
| Guangdong | 440981 | Gaozhou City | 5.3 | 4.4 | 6.2 |  | 95.8(92.5,97.8) | 96.2(93.0,98.2) | 93.4(88.4,96.6) |
| Guangdong | 440203 | Wujiang District | 5.6 | 4.4 | 6.8 |  | 95.7(89.3,98.7) | 96.4(89.8,99.2) | 92.3(80.9,98.1) |
| Guangdong | 440402 | Xiangzhou District | 2.7 | 2.3 | 3.0 |  | 95.7(82.3,99.6) | 92.6(76.1,99.1) | 96.8(84.5,99.8) |
| Guangdong | 440605 | Nanhai District | 2.9 | 2.8 | 3.0 |  | 95.7(92.0,98.1) | 94.6(89.9,97.6) | 95.6(91.6,98.1) |
| Guangdong | 440982 | Huazhou City | 3.0 | 2.2 | 3.8 |  | 95.5(90.2,98.3) | 96.1(90.7,98.8) | 91.4(82.3,96.6) |
| Guangdong | 441523 | Luhe County | 5.2 | 4.1 | 6.3 |  | 95.2(89.2,98.4) | 93.0(85.0,97.4) | 96.6(90.2,99.2) |
| Guangdong | 440403 | Doumen District | 4.4 | 3.7 | 5.1 |  | 95.1(89.5,98.4) | 96.0(90.0,99.0) | 91.9(83.2,97.3) |
| Guangdong | 440307 | Longgang District | 1.2 | 1.0 | 1.4 |  | 95.0(90.0,97.8) | 94.2(88.2,97.6) | 95.1(89.4,98.0) |
| Guangdong | 441302 | Huicheng District | 2.5 | 2.2 | 2.8 |  | 95.0(89.2,97.9) | 94.6(88.6,98.1) | 94.2(87.6,97.9) |
| Guangdong | 441802 | Qingcheng District | 5.3 | 4.9 | 5.7 |  | 95.0(91.1,97.5) | 94.8(90.3,97.8) | 94.1(88.9,97.2) |
| Guangdong | 440607 | Sanshui District | 4.4 | 4.2 | 4.5 |  | 94.7(88.9,97.8) | 93.4(84.9,97.6) | 94.8(88.2,98.4) |
| Guangdong | 440229 | Wengyuan County | 7.2 | 6.2 | 8.2 |  | 94.6(89.6,97.6) | 92.8(86.9,96.4) | 95.9(89.9,98.5) |
| Guangdong | 440781 | Taishan City | 7.9 | 6.8 | 8.9 |  | 94.3(90.4,96.7) | 94.4(90.3,97.1) | 92.6(87.3,95.8) |
| Guangdong | 441602 | Yuancheng District | 4.0 | 3.5 | 4.6 |  | 94.3(87.6,98.0) | 96.1(89.0,99.0) | 88.4(77.9,95.8) |
| Guangdong | 441323 | Huidong County | 3.1 | 2.4 | 3.6 |  | 94.2(86.9,98.3) | 92.3(82.7,97.7) | 95.0(84.9,99.0) |
| Guangdong | 440784 | Heshan City | 5.7 | 5.1 | 6.2 |  | 94.1(87.8,97.8) | 92.0(83.8,96.8) | 95.1(87.5,98.7) |
| Guangdong | 445321 | Xinxing County | 6.8 | 6.5 | 7.2 |  | 94.1(87.7,97.6) | 91.2(83.7,96.2) | 95.6(89.2,98.8) |
| Guangdong | 440183 | Zengcheng City | 4.0 | 3.5 | 4.5 |  | 93.9(89.0,97.2) | 93.6(87.8,97.2) | 91.9(84.8,96.3) |
| Guangdong | 441426 | Pingyuan County | 7.3 | 6.5 | 8.0 |  | 93.9(87.4,97.5) | 91.6(83.2,97.2) | 95.0(87.2,98.8) |
| Guangdong | 441423 | Fengshun County | 7.7 | 6.6 | 8.7 |  | 93.7(88.9,96.6) | 89.7(83.4,93.6) | 96.5(92.6,98.7) |
| Guangdong | 441202 | Duanzhou District | 4.6 | 3.8 | 5.3 |  | 93.5(85.0,97.7) | 94.5(86.5,98.5) | 89.4(75.6,96.9) |
| Guangdong | 440282 | Nanxiong City | 9.5 | 8.7 | 10.5 |  | 93.4(88.9,96.3) | 93.1(87.8,96.3) | 92.4(86.2,96.3) |
| Guangdong | 440205 | Qujiang District | 6.4 | 5.5 | 7.2 |  | 93.2(86.6,96.8) | 93.1(86.4,96.9) | 91.8(83.1,97.2) |
| Guangdong | 440515 | Chenghai District | 6.1 | 5.7 | 6.5 |  | 93.1(82.3,98.2) | 93.9(82.6,98.8) | 91.1(79.7,97.1) |
| Guangdong | 440184 | Conghua City | 4.9 | 4.3 | 5.5 |  | 93.0(87.3,96.6) | 91.7(84.6,96.3) | 93.7(86.0,97.8) |
| Guangdong | 440703 | Pengjiang District | 4.3 | 3.9 | 4.7 |  | 93.0(85.1,97.2) | 93.3(85.1,97.3) | 91.6(83.4,96.9) |
| Guangdong | 445281 | Puning City | 4.7 | 4.0 | 5.4 |  | 92.8(88.3,96.0) | 91.7(86.7,95.2) | 94.0(89.4,96.8) |
| Guangdong | 441481 | Xingning City | 7.0 | 6.4 | 7.6 |  | 92.7(88.5,95.5) | 89.9(84.2,93.7) | 94.7(90.6,97.3) |
| Guangdong | 441723 | Yangdong County | 6.0 | 5.4 | 6.6 |  | 92.6(86.4,97.1) | 94.0(87.1,97.7) | 88.8(79.8,94.6) |
| Guangdong | 441427 | Jiaoling County | 8.2 | 7.4 | 9.0 |  | 92.5(85.9,96.6) | 90.2(80.9,96.3) | 93.8(85.5,97.9) |
| Guangdong | 440106 | Tianhe District | 2.0 | 1.8 | 2.3 |  | 92.4(84.9,96.8) | 90.1(81.3,96.0) | 93.2(85.2,97.8) |
| Guangdong | 440785 | Enping City | 6.7 | 6.0 | 7.3 |  | 92.4(80.5,98.3) | 90.2(76.0,97.4) | 93.4(82.4,98.7) |
| Guangdong | 441402 | Meijiang District | 5.3 | 4.7 | 5.8 |  | 92.4(84.8,97.0) | 90.3(79.1,96.5) | 93.3(83.1,98.3) |
| Guangdong | 441502 | Chengqu | 4.2 | 3.2 | 5.2 |  | 92.4(85.0,96.7) | 91.5(84.1,96.3) | 94.3(86.1,98.3) |
| Guangdong | 440111 | Baiyun District | 2.6 | 2.4 | 2.8 |  | 92.3(86.7,95.9) | 91.3(84.5,95.9) | 92.7(86.4,96.3) |
| Guangdong | 440513 | Chaoyang District | 4.4 | 4.0 | 4.7 |  | 92.0(86.6,95.7) | 91.2(85.6,95.2) | 93.2(87.6,96.5) |
| Guangdong | 441226 | Deqing County | 7.2 | 6.0 | 8.3 |  | 92.0(86.1,95.5) | 94.1(88.3,97.3) | 85.6(77.0,91.7) |
| Guangdong | 441422 | Dapu County | 9.3 | 8.0 | 10.5 |  | 92.0(87.0,95.2) | 90.4(84.4,94.1) | 93.0(87.5,96.1) |
| Guangdong | 440404 | Jinwan District | 2.7 | 2.3 | 3.0 |  | 91.8(68.6,99.6) | 85.5(60.6,98.8) | 95.7(74.9,99.8) |
| Guangdong | 441324 | Longmen County | 6.1 | 5.2 | 6.9 |  | 91.3(83.6,96.2) | 89.6(80.1,95.0) | 92.2(81.8,97.4) |
| Guangdong | 441821 | Fogang County | 7.0 | 6.0 | 7.9 |  | 91.3(85.8,95.4) | 90.3(83.8,95.0) | 92.1(85.4,96.2) |
| Guangdong | 440233 | Xinfeng County | 7.0 | 6.1 | 7.9 |  | 90.8(82.2,95.7) | 88.4(80.3,94.7) | 92.2(82.2,97.7) |
| Guangdong | 440232 | Ruyuan Yaozu Zizhixian | 6.8 | 5.7 | 7.9 |  | 90.7(82.6,96.1) | 90.6(82.0,96.1) | 89.0(76.9,96.3) |
| Guangdong | 441283 | Gaoyao City | 6.1 | 5.8 | 6.4 |  | 90.6(84.0,94.4) | 90.4(84.2,94.9) | 88.7(82.0,93.9) |
| Guangdong | 440512 | Haojiang District | 4.9 | 4.3 | 5.4 |  | 89.8(81.1,95.2) | 88.8(78.8,95.0) | 92.1(82.8,97.5) |
| Guangdong | 441881 | Yingde City | 6.7 | 6.0 | 7.3 |  | 89.8(84.4,93.5) | 87.1(81.1,91.5) | 92.0(86.0,95.5) |
| Guangdong | 445222 | Jiexi County | 5.8 | 4.9 | 6.6 |  | 89.8(83.8,94.6) | 89.6(81.9,94.3) | 90.3(83.8,94.8) |
| Guangdong | 440222 | Shixing County | 7.5 | 7.0 | 8.0 |  | 89.7(82.9,94.5) | 90.1(81.8,95.8) | 87.9(78.4,94.5) |
| Guangdong | 440523 | Nan'ao County | 8.1 | 7.8 | 8.4 |  | 89.2(76.9,97.5) | 90.4(76.5,98.7) | 89.0(78.5,94.8) |
| Guangdong | 440204 | Zhenjiang District | 5.4 | 4.6 | 6.2 |  | 88.7(79.0,95.7) | 87.1(74.6,95.0) | 88.3(73.5,96.7) |
| Guangdong | 441827 | Qingxin County | 6.1 | 5.4 | 6.8 |  | 88.6(81.8,92.6) | 85.4(78.2,90.7) | 91.9(85.8,95.5) |
| Guangdong | 441225 | Fengkai County | 6.3 | 5.0 | 7.6 |  | 88.4(81.0,93.9) | 92.7(86.3,96.5) | 76.8(65.2,86.5) |
| Guangdong | 440881 | Lianjiang City | 4.2 | 3.6 | 4.7 |  | 88.3(82.8,92.8) | 87.8(81.1,92.6) | 87.2(79.8,92.8) |
| Guangdong | 445221 | Jiedong County | 5.3 | 4.7 | 5.8 |  | 88.0(81.2,93.3) | 88.6(81.8,93.9) | 86.6(78.5,92.4) |
| Guangdong | 440303 | Luohu District | 1.7 | 1.2 | 2.1 |  | 87.8(76.9,94.4) | 87.2(75.5,94.4) | 88.7(78.2,96.0) |
| Guangdong | 445323 | Yun'an County | 6.8 | 5.8 | 7.6 |  | 87.8(79.7,93.1) | 89.9(81.8,95.5) | 83.5(73.4,91.3) |
| Guangdong | 440804 | Potou District | 1.4 | 1.2 | 1.5 |  | 87.6(68.2,96.9) | 68.8(45.6,89.2) | 96.2(76.7,99.7) |
| Guangdong | 441224 | Huaiji County | 4.1 | 3.0 | 5.2 |  | 87.3(80.0,92.6) | 90.2(84.5,94.4) | 81.0(70.6,89.2) |
| Guangdong | 440224 | Renhua County | 6.0 | 5.2 | 6.9 |  | 86.8(77.0,93.6) | 89.4(79.5,95.8) | 79.9(66.1,90.7) |
| Guangdong | 445381 | Luoding City | 6.4 | 5.2 | 7.7 |  | 86.7(80.5,91.2) | 86.1(80.1,91.0) | 86.5(79.8,91.9) |
| Guangdong | 445122 | Raoping County | 7.0 | 6.3 | 7.7 |  | 86.0(79.4,90.9) | 85.5(78.9,90.6) | 87.2(80.5,92.0) |
| Guangdong | 445121 | Chaoan County | 6.1 | 5.5 | 6.7 |  | 85.6(79.0,90.6) | 84.0(76.5,89.6) | 87.4(80.5,92.1) |
| Guangdong | 440305 | Nanshan District | 1.2 | 1.0 | 1.4 |  | 85.5(64.3,96.6) | 86.5(66.1,97.6) | 85.5(67.0,96.0) |
| Guangdong | 440304 | Futian District | 1.3 | 1.0 | 1.6 |  | 85.2(74.2,92.8) | 84.6(72.1,93.0) | 85.1(74.6,93.3) |
| Guangdong | 441624 | Heping County | 7.3 | 6.5 | 8.1 |  | 84.0(76.9,89.2) | 83.8(76.4,88.8) | 84.5(76.5,90.5) |
| Guangdong | 445322 | Yu'nan County | 7.3 | 6.2 | 8.4 |  | 83.1(74.8,89.1) | 82.6(73.2,89.5) | 81.8(72.1,88.8) |
| Guangdong | 440281 | Lechang City | 5.6 | 4.4 | 6.6 |  | 82.3(72.6,90.4) | 84.1(73.7,91.3) | 77.3(63.1,88.3) |
| Guangdong | 440514 | Chaonan District | 3.4 | 3.1 | 3.7 |  | 81.7(62.3,92.4) | 80.1(62.8,91.9) | 84.9(70.5,94.6) |
| Guangdong | 441781 | Yangchun City | 5.7 | 5.1 | 6.2 |  | 81.1(65.6,92.1) | 76.0(61.1,88.3) | 85.5(69.4,95.2) |
| Guangdong | 441623 | Lianping County | 6.3 | 5.9 | 6.7 |  | 81.0(64.7,92.6) | 80.5(64.4,92.5) | 81.1(66.1,90.8) |
| Guangdong | 441581 | Lufeng City | 4.4 | 3.5 | 5.2 |  | 80.9(72.3,87.7) | 80.8(72.7,87.6) | 82.2(74.2,88.5) |
| Guangdong | 441521 | Haifeng County | 4.2 | 3.4 | 4.9 |  | 79.3(70.3,86.5) | 79.4(69.4,87.6) | 78.4(67.7,86.8) |
| Guangdong | 440112 | Huangpu District | 2.1 | 1.9 | 2.3 |  | 79.2(67.7,88.6) | 75.9(61.4,87.0) | 83.3(70.8,92.3) |
| Guangdong | 441882 | Lianzhou City | 5.5 | 3.9 | 7.1 |  | 78.5(67.8,86.6) | 86.5(77.1,93.2) | 57.7(42.6,72.0) |
| Guangdong | 445224 | Huilai County | 3.7 | 2.9 | 4.5 |  | 78.5(69.9,85.3) | 82.1(73.6,88.4) | 75.7(66.6,84.2) |
| Guangdong | 440825 | Xuwen County | 2.4 | 2.0 | 2.7 |  | 78.2(66.5,87.5) | 80.6(68.1,90.5) | 72.0(57.5,85.4) |
| Guangdong | 440308 | Yantian District | 1.1 | 0.9 | 1.2 |  | 77.9(58.2,94.0) | 83.3(56.9,97.6) | 76.8(65.3,84.8) |
| Guangdong | 441622 | Longchuan County | 4.5 | 3.5 | 5.4 |  | 76.9(67.0,84.3) | 78.7(68.9,86.2) | 74.1(62.7,83.4) |
| Guangdong | 441721 | Yangxi County | 4.6 | 3.9 | 5.1 |  | 76.5(65.4,85.1) | 70.3(58.6,80.3) | 82.5(70.1,91.9) |
| Guangdong | 441625 | Dongyuan County | 5.3 | 4.6 | 6.0 |  | 75.7(66.3,83.1) | 76.3(66.4,84.3) | 75.8(66.0,84.3) |
| Guangdong | 441826 | Zizhixian | 6.4 | 5.0 | 7.8 |  | 75.0(63.6,84.4) | 77.3(66.0,86.4) | 69.3(53.1,83.8) |
| Guangdong | 440811 | Mazhang District | 2.4 | 2.0 | 2.7 |  | 74.6(61.2,86.5) | 73.9(60.2,86.2) | 73.3(56.5,88.0) |
| Guangdong | 440983 | Xinyi City | 2.8 | 1.8 | 3.7 |  | 71.0(59.8,81.2) | 75.5(64.5,85.9) | 62.6(48.6,75.5) |
| Guangdong | 440882 | Leizhou City | 0.7 | 0.5 | 0.8 |  | 63.9(49.5,77.6) | 62.8(50.0,76.3) | 64.5(49.3,80.1) |
| Guangdong | 441621 | Zijin County | 3.5 | 2.5 | 4.4 |  | 63.3(52.5,72.8) | 65.8(55.7,75.4) | 61.0(48.1,73.0) |
| Guangdong | 441825 | Yaozu Zizhixian | 4.8 | 3.9 | 5.5 |  | 54.2(40.4,67.8) | 60.8(45.2,77.0) | 44.2(31.0,59.4) |
| Guangdong | 440823 | Suixi County | 1.5 | 1.2 | 1.7 |  | 47.6(35.9,61.5) | 48.6(34.6,64.0) | 46.9(34.7,63.0) |
| Yunnan | 530111 | Guandu District | 4.7 | 4.0 | 5.3 |  | 100(99.9,100.0) | 100.0(99.9,100.0) | 99.9(99.4,100.0) |
| Yunnan | 533323 | Fugong County | 6.7 | 5.2 | 8.1 |  | 99.7(98.5,100.0) | 99.6(97.9,100.0) | 99.4(96.4,99.9) |
| Yunnan | 533324 | Nuzu Zizhixian | 8.0 | 5.4 | 10.2 |  | 99.7(97.8,100.0) | 99.8(98.1,100.0) | 98.0(84.6,99.9) |
| Yunnan | 530112 | Xishan District | 4.8 | 4.1 | 5.4 |  | 98.0(94.1,99.5) | 97.8(92.2,99.6) | 97.2(91.8,99.4) |
| Yunnan | 532503 | Mengzi County | 6.0 | 4.9 | 7.0 |  | 97.9(94.9,99.4) | 98.0(94.6,99.5) | 97.2(92.7,99.2) |
| Yunnan | 530421 | Jiangchuan County | 6.6 | 5.7 | 7.4 |  | 97.3(92.5,99.2) | 97.5(91.6,99.5) | 95.7(88.0,98.9) |
| Yunnan | 532823 | Mengla County | 6.3 | 5.0 | 7.6 |  | 96.8(92.6,98.8) | 97.0(92.9,99.1) | 94.7(88.1,98.3) |
| Yunnan | 530802 | Simao District | 5.0 | 4.3 | 5.8 |  | 96.3(91.1,99.0) | 93.4(85.0,97.9) | 97.5(92.1,99.5) |
| Yunnan | 532523 | Zizhixian | 6.5 | 5.4 | 7.5 |  | 96.2(90.6,98.9) | 96.0(88.4,99.1) | 94.6(84.7,98.7) |
| Yunnan | 530724 | Ninglang Yizu Zizhixian | 6.5 | 5.9 | 7.1 |  | 96.0(91.1,98.5) | 96.1(90.4,98.9) | 93.0(85.6,97.0) |
| Yunnan | 532922 | Yangbi Yizu Zizhixian | 6.1 | 5.5 | 6.8 |  | 95.9(87.7,99.1) | 98.1(90.3,99.8) | 87.7(73.3,96.2) |
| Yunnan | 530302 | Qulin City | 5.9 | 5.0 | 6.8 |  | 95.8(92.2,98.1) | 94.8(89.9,97.7) | 95.8(91.5,98.3) |
| Yunnan | 530423 | Tonghai County | 6.5 | 5.9 | 7.1 |  | 95.6(90.0,98.4) | 88.9(79.3,95.4) | 98.4(94.4,99.7) |
| Yunnan | 532530 | Daizu Zizhixian | 6.1 | 4.9 | 7.0 |  | 95.2(90.4,97.9) | 94.5(89.5,97.7) | 94.4(87.8,98.0) |
| Yunnan | 533321 | Lushui County | 6.3 | 4.9 | 7.6 |  | 95.0(88.9,98.3) | 94.3(86.4,98.4) | 93.8(85.2,98.4) |
| Yunnan | 532801 | Jinghong City | 5.9 | 4.9 | 6.8 |  | 94.9(90.4,97.6) | 92.9(86.1,96.8) | 95.7(90.8,98.5) |
| Yunnan | 530114 | Chenggong County | 4.1 | 3.7 | 4.4 |  | 94.8(87.5,98.4) | 95.3(85.9,98.8) | 92.4(83.1,97.5) |
| Yunnan | 532532 | Hekou Yaozu Zizhixian | 6.1 | 4.8 | 7.2 |  | 94.3(85.3,98.4) | 92.1(80.2,98.0) | 94.9(81.3,99.3) |
| Yunnan | 532301 | Chuxiong City | 6.3 | 5.2 | 7.4 |  | 94.1(88.9,97.5) | 93.8(87.7,97.7) | 93.1(86.7,97.2) |
| Yunnan | 532822 | Menghai County | 7.0 | 5.7 | 8.2 |  | 94.0(89.2,97.2) | 95.7(90.8,98.1) | 89.3(81.7,94.4) |
| Yunnan | 530624 | Daguan County | 6.5 | 5.3 | 7.6 |  | 93.9(87.8,97.3) | 93.4(87.2,97.5) | 91.9(84.0,96.9) |
| Yunnan | 530321 | Malong County | 6.4 | 5.6 | 7.3 |  | 93.8(86.5,97.8) | 96.1(89.3,99.0) | 84.7(71.6,93.1) |
| Yunnan | 530402 | Hongta District | 5.6 | 4.9 | 6.4 |  | 93.8(87.7,97.3) | 92.8(84.7,97.4) | 93.8(85.4,97.8) |
| Yunnan | 532924 | Binchuan County | 6.6 | 5.6 | 7.5 |  | 93.0(86.8,96.7) | 94.5(87.9,98.0) | 90.1(81.9,95.3) |
| Yunnan | 533421 | Shanggelila County | 5.7 | 5.0 | 6.3 |  | 92.7(83.8,97.7) | 95.8(87.2,99.1) | 80.8(65.0,92.1) |
| Yunnan | 533423 | Weixi Lisuzu ZizhiXian | 7.0 | 5.0 | 8.7 |  | 92.5(85.2,97.0) | 95.9(89.9,98.9) | 77.7(63.0,88.6) |
| Yunnan | 530122 | Jinning County | 6.4 | 5.6 | 7.1 |  | 92.3(84.5,96.7) | 88.4(77.9,95.6) | 94.2(84.0,98.1) |
| Yunnan | 532524 | Jianshui County | 6.2 | 5.5 | 6.9 |  | 92.3(86.8,96.2) | 92.3(85.3,96.6) | 90.9(83.5,95.6) |
| Yunnan | 530324 | Luoping County | 6.2 | 5.3 | 7.0 |  | 92.0(86.0,95.9) | 90.8(84.4,95.4) | 92.1(86.2,96.2) |
| Yunnan | 530622 | Qiaojia County | 6.6 | 5.6 | 7.5 |  | 91.8(86.0,95.6) | 91.9(85.7,96.0) | 88.9(81.6,94.2) |
| Yunnan | 530427 | Zizhixian | 6.6 | 6.0 | 7.1 |  | 91.7(84.7,96.4) | 92.1(83.2,97.0) | 88.5(78.1,95.0) |
| Yunnan | 530426 | Eshan Yizu Zizhixian | 6.7 | 6.3 | 7.1 |  | 91.6(81.7,97.0) | 91.1(77.9,97.8) | 91.1(79.1,97.6) |
| Yunnan | 532625 | Maguan County | 6.8 | 5.7 | 7.7 |  | 91.6(85.6,95.8) | 91.1(83.8,95.9) | 90.1(82.5,95.6) |
| Yunnan | 532526 | Mile County | 6.4 | 5.5 | 7.3 |  | 91.5(85.5,95.6) | 92.9(86.7,96.8) | 87.4(78.2,93.3) |
| Yunnan | 532622 | Yanshan County | 6.4 | 5.1 | 7.5 |  | 91.4(86.1,95.5) | 92.5(86.3,96.2) | 87.8(81.0,93.4) |
| Yunnan | 530425 | Yimen County | 7.4 | 6.7 | 8.0 |  | 91.3(81.7,97.2) | 86.0(73.0,94.9) | 94.5(84.6,98.8) |
| Yunnan | 530827 | Vazu Zizhixian | 5.9 | 4.6 | 7.2 |  | 91.3(81.7,97.2) | 90.5(78.6,97.2) | 91.0(78.2,97.4) |
| Yunnan | 530325 | Fuyuan County | 5.8 | 5.0 | 6.5 |  | 91.2(86.3,95.4) | 90.5(83.6,94.8) | 90.7(85.3,95.0) |
| Yunnan | 530523 | Longling County | 6.4 | 5.9 | 6.9 |  | 91.1(84.6,96.0) | 88.7(79.3,94.7) | 92.4(84.2,96.7) |
| Yunnan | 532927 | Zizhixian | 6.9 | 5.9 | 7.8 |  | 91.0(77.4,97.6) | 91.8(78.1,98.4) | 87.6(72.5,96.4) |
| Yunnan | 532325 | Yao'an County | 7.5 | 6.6 | 8.4 |  | 90.8(78.4,98.0) | 90.8(77.1,98.4) | 88.6(75.3,97.1) |
| Yunnan | 533102 | Ruili City | 5.5 | 4.1 | 6.7 |  | 90.8(82.2,96.1) | 94.1(85.6,98.2) | 82.7(69.8,92.1) |
| Yunnan | 530424 | Huaning County | 6.4 | 5.5 | 7.2 |  | 90.4(81.1,96.0) | 89.6(79.5,96.5) | 89.9(79.4,96.3) |
| Yunnan | 532626 | Qiubei County | 6.4 | 5.6 | 7.1 |  | 90.3(83.6,94.5) | 87.7(79.3,93.9) | 91.5(84.9,95.8) |
| Yunnan | 532627 | Guangnan County | 6.4 | 5.5 | 7.1 |  | 90.3(84.1,94.7) | 86.8(79.5,92.3) | 91.7(86.2,95.9) |
| Yunnan | 530127 | Songming County | 6.0 | 5.2 | 6.9 |  | 90.2(82.5,95.5) | 87.3(77.5,94.1) | 92.1(83.4,96.7) |
| Yunnan | 532624 | Malipo County | 6.6 | 5.3 | 7.8 |  | 90.0(82.4,95.2) | 91.4(83.8,96.5) | 83.9(71.8,92.6) |
| Yunnan | 532628 | Funing County | 6.3 | 5.2 | 7.4 |  | 90.0(82.7,94.6) | 92.1(84.8,96.4) | 85.0(74.3,91.9) |
| Yunnan | 530103 | Panlong District | 4.0 | 3.1 | 4.9 |  | 89.9(80.7,95.7) | 90.1(79.7,96.7) | 86.8(74.5,94.9) |
| Yunnan | 530925 | Vazu Blangzu Daizu | 6.3 | 5.3 | 7.2 |  | 89.8(81.2,95.5) | 79.8(68.8,88.8) | 95.5(87.6,98.8) |
| Yunnan | 530630 | Shuifu County | 5.9 | 4.5 | 7.1 |  | 89.7(77.9,96.2) | 89.7(76.6,97.4) | 84.7(65.8,96.2) |
| Yunnan | 530902 | Linxiang District | 6.2 | 4.7 | 7.5 |  | 89.4(81.8,94.3) | 91.5(84.4,96.2) | 83.9(71.8,92.0) |
| Yunnan | 533123 | Yingjiang County | 6.0 | 5.0 | 6.9 |  | 89.3(82.4,94.3) | 92.9(86.1,97.1) | 79.7(69.6,87.2) |
| Yunnan | 532501 | Gejiu City | 6.1 | 4.9 | 7.1 |  | 89.1(81.4,94.1) | 90.8(83.6,95.7) | 84.3(74.6,91.9) |
| Yunnan | 533325 | Zizhixian | 6.3 | 5.4 | 7.1 |  | 89.1(80.8,94.6) | 82.7(71.5,90.6) | 93.3(85.2,97.6) |
| Yunnan | 532502 | Kaiyuan City | 6.3 | 5.2 | 7.3 |  | 88.9(80.9,94.5) | 90.4(82.1,95.7) | 85.9(75.2,93.0) |
| Yunnan | 530721 | Zizhixian | 6.6 | 6.0 | 7.3 |  | 88.8(71.9,97.5) | 81.0(61.0,94.9) | 92.4(75.7,99.2) |
| Yunnan | 532601 | Wenshan County | 6.6 | 5.4 | 7.7 |  | 88.5(82.3,93.2) | 90.3(83.0,95.0) | 84.7(76.0,90.8) |
| Yunnan | 530628 | Yiliang County | 6.3 | 5.0 | 7.5 |  | 88.4(81.2,93.3) | 90.0(83.2,94.4) | 83.8(74.9,90.4) |
| Yunnan | 530723 | Huaping County | 6.6 | 6.3 | 6.8 |  | 88.4(77.8,95.2) | 90.7(77.1,97.6) | 84.1(71.2,93.3) |
| Yunnan | 532331 | Lufeng County | 6.7 | 6.0 | 7.4 |  | 88.4(80.2,93.6) | 88.3(78.9,94.6) | 88.0(79.8,93.7) |
| Yunnan | 530825 | Lahuzu Zizhixian | 6.6 | 5.3 | 7.7 |  | 88.2(78.2,94.3) | 91.7(80.9,96.9) | 80.6(69.5,90.1) |
| Yunnan | 530702 | Gucheng District | 5.3 | 4.1 | 6.4 |  | 88.1(76.7,95.4) | 89.5(75.7,96.8) | 85.5(69.9,95.1) |
| Yunnan | 533103 | Mang City | 5.7 | 4.6 | 6.8 |  | 88.1(79.9,93.7) | 89.3(80.4,95.2) | 84.4(73.0,91.8) |
| Yunnan | 532328 | Yuanmou County | 6.9 | 5.5 | 8.1 |  | 87.9(78.4,94.0) | 88.7(79.0,95.1) | 85.3(72.6,94.0) |
| Yunnan | 530422 | Chengjiang County | 6.3 | 5.8 | 6.9 |  | 87.7(77.3,94.5) | 79.8(67.1,90.3) | 93.3(82.7,98.1) |
| Yunnan | 530521 | Shidian County | 7.9 | 7.1 | 8.7 |  | 87.7(79.9,93.1) | 85.5(76.0,92.0) | 89.0(81.3,94.1) |
| Yunnan | 530626 | Suijiang County | 6.3 | 5.4 | 7.1 |  | 87.7(77.6,94.3) | 82.5(70.2,91.8) | 92.0(80.6,97.9) |
| Yunnan | 532326 | Dayao County | 7.8 | 6.7 | 8.8 |  | 87.7(79.2,93.3) | 85.1(75.6,92.0) | 90.0(80.9,95.7) |
| Yunnan | 533124 | Longchuan County | 6.1 | 4.6 | 7.6 |  | 87.7(79.0,93.9) | 90.4(82.1,95.6) | 80.4(67.0,89.5) |
| Yunnan | 530826 | Zizhixian | 5.9 | 4.7 | 7.0 |  | 87.6(76.3,94.9) | 88.1(76.0,96.0) | 84.2(68.6,94.5) |
| Yunnan | 532923 | Xiangyun County | 6.7 | 5.9 | 7.4 |  | 87.2(79.1,92.9) | 82.5(72.4,90.3) | 90.2(82.3,95.2) |
| Yunnan | 532925 | Midu County | 7.3 | 6.4 | 8.2 |  | 87.1(78.5,93.0) | 86.8(77.4,93.3) | 86.8(75.9,93.5) |
| Yunnan | 532322 | Shuangbai County | 6.4 | 5.7 | 7.1 |  | 87.0(75.8,94.8) | 87.8(74.0,96.2) | 85.3(71.6,95.2) |
| Yunnan | 532323 | Mouding County | 6.7 | 5.8 | 7.6 |  | 86.7(75.3,93.9) | 83.6(69.7,92.4) | 88.4(75.9,95.8) |
| Yunnan | 530328 | Zhanyi County | 6.2 | 5.2 | 7.1 |  | 86.6(78.8,92.4) | 89.1(80.7,95.0) | 81.4(72.4,88.9) |
| Yunnan | 532928 | Yongping County | 6.8 | 5.5 | 8.0 |  | 86.2(76.1,93.4) | 90.2(79.8,96.4) | 77.3(66.3,87.2) |
| Yunnan | 530381 | Xuanwei City | 6.1 | 5.0 | 7.0 |  | 86.1(79.4,91.4) | 86.5(79.6,92.0) | 84.4(76.8,89.5) |
| Yunnan | 530828 | Zizhixian | 6.3 | 5.1 | 7.5 |  | 85.9(78.5,91.9) | 89.1(81.0,94.4) | 78.5(68.7,86.6) |
| Yunnan | 530322 | Luliang County | 5.6 | 5.0 | 6.1 |  | 85.8(77.9,91.5) | 80.6(70.3,88.0) | 89.6(82.8,94.6) |
| Yunnan | 530113 | Dongchuan District | 7.2 | 6.0 | 8.4 |  | 85.7(76.3,92.2) | 85.1(75.2,92.4) | 83.8(71.2,92.4) |
| Yunnan | 530126 | Shilin Yizu Zizhixian | 6.7 | 5.8 | 7.5 |  | 85.5(76.5,92.1) | 88.0(76.7,94.4) | 80.5(69.2,88.5) |
| Yunnan | 530323 | Shizong County | 6.0 | 5.4 | 6.5 |  | 85.5(77.2,92.0) | 84.5(74.6,91.6) | 85.0(75.8,91.1) |
| Yunnan | 532327 | Yongren County | 6.6 | 6.2 | 7.0 |  | 85.4(72.3,94.6) | 79.7(62.9,93.0) | 89.0(73.8,97.6) |
| Yunnan | 530502 | Longyang District | 6.6 | 5.8 | 7.4 |  | 84.6(77.5,89.7) | 83.2(74.7,89.5) | 85.6(78.3,90.7) |
| Yunnan | 530621 | Ludian County | 6.2 | 5.3 | 7.0 |  | 84.5(75.9,90.3) | 81.0(70.5,88.4) | 87.4(79.5,92.6) |
| Yunnan | 530602 | Zhaoyang District | 6.0 | 5.0 | 6.9 |  | 84.4(76.9,90.0) | 83.6(75.2,89.7) | 85.1(78.0,90.4) |
| Yunnan | 530625 | Yongshan County | 6.2 | 5.4 | 6.8 |  | 84.4(76.2,90.4) | 82.1(72.7,88.8) | 86.3(77.1,93.0) |
| Yunnan | 530524 | Changning County | 6.3 | 5.7 | 6.8 |  | 84.2(75.0,91.2) | 85.1(73.5,92.9) | 81.1(69.5,89.8) |
| Yunnan | 532527 | Luxi County | 6.2 | 5.4 | 6.8 |  | 84.0(74.8,90.3) | 84.0(73.3,91.2) | 82.8(73.7,89.8) |
| Yunnan | 532623 | Xichou County | 6.7 | 6.0 | 7.2 |  | 83.8(74.1,90.3) | 81.3(69.7,90.5) | 84.6(74.2,92.2) |
| Yunnan | 532930 | Eryuan County | 6.2 | 5.6 | 6.8 |  | 83.8(74.9,90.3) | 84.8(74.3,92.2) | 82.1(71.5,89.6) |
| Yunnan | 530829 | Ximeng Vazu Zizhixian | 6.1 | 5.2 | 6.9 |  | 83.7(71.3,92.1) | 84.3(70.8,94.3) | 81.4(65.7,92.3) |
| Yunnan | 532324 | Nanhua County | 6.7 | 5.9 | 7.6 |  | 83.7(73.4,90.9) | 86.5(75.3,94.3) | 79.4(68.3,89.2) |
| Yunnan | 532926 | Nanjian Yizu Zizhixian | 6.7 | 5.4 | 8.0 |  | 83.0(73.2,90.6) | 83.7(73.7,92.0) | 79.8(66.4,90.3) |
| Yunnan | 533122 | Lianghe County | 6.2 | 5.5 | 6.8 |  | 82.6(72.5,90.7) | 80.3(66.0,89.6) | 84.8(70.6,93.5) |
| Yunnan | 532525 | Shiping County | 6.2 | 5.3 | 7.0 |  | 82.1(72.4,89.6) | 83.2(72.1,91.3) | 79.4(67.8,88.1) |
| Yunnan | 530129 | Zizhixian | 7.3 | 6.1 | 8.5 |  | 81.9(72.4,88.4) | 79.7(69.7,87.3) | 80.9(71.7,88.5) |
| Yunnan | 530821 | Zizhixian | 6.4 | 5.2 | 7.4 |  | 81.9(70.9,90.6) | 83.2(70.4,92.5) | 77.7(61.0,89.6) |
| Yunnan | 530522 | Tengchong County | 6.2 | 5.4 | 6.9 |  | 81.8(73.8,87.7) | 81.5(71.8,87.9) | 81.7(72.4,88.5) |
| Yunnan | 532929 | Yunlong County | 6.8 | 5.7 | 7.7 |  | 81.4(71.4,89.3) | 80.0(67.6,88.9) | 81.4(68.8,90.5) |
| Yunnan | 530124 | Fumin County | 6.7 | 5.6 | 7.9 |  | 81.1(69.1,90.4) | 81.8(67.5,91.5) | 79.4(64.7,90.5) |
| Yunnan | 532932 | Heqing County | 6.5 | 5.7 | 7.4 |  | 80.7(70.7,88.2) | 84.1(73.5,92.2) | 73.9(62.0,83.8) |
| Yunnan | 530823 | Jingdong Yizu Zizhixian | 6.6 | 5.6 | 7.6 |  | 80.0(71.4,87.2) | 80.7(70.9,88.6) | 79.4(69.5,87.4) |
| Yunnan | 530128 | Zizhixian | 7.5 | 6.1 | 8.8 |  | 79.9(70.6,87.1) | 85.0(75.8,91.7) | 67.6(57.5,77.7) |
| Yunnan | 530627 | Zhenxiong County | 5.6 | 4.5 | 6.6 |  | 79.9(71.5,86.3) | 79.5(70.9,86.5) | 79.5(72.1,85.8) |
| Yunnan | 532901 | Dali City | 4.7 | 3.9 | 5.4 |  | 79.8(69.7,88.4) | 86.0(74.8,93.9) | 69.2(57.9,79.3) |
| Yunnan | 530822 | Zizhixian | 6.7 | 5.6 | 7.7 |  | 79.7(70.3,86.4) | 82.4(72.6,89.7) | 72.9(61.9,81.7) |
| Yunnan | 530923 | Yongde County | 6.3 | 5.6 | 7.0 |  | 78.9(70.1,86.1) | 77.2(67.2,86.0) | 80.1(70.1,87.9) |
| Yunnan | 530181 | Anning City | 4.8 | 4.0 | 5.4 |  | 78.8(66.9,88.2) | 76.4(61.4,88.8) | 79.1(65.3,91.6) |
| Yunnan | 530428 | Daizu Zizhixian | 6.8 | 5.8 | 7.7 |  | 78.4(67.9,86.8) | 77.3(67.2,86.1) | 79.6(68.0,88.6) |
| Yunnan | 530924 | Zhenkang County | 6.2 | 5.0 | 7.3 |  | 78.0(66.5,87.3) | 79.8(66.9,89.0) | 72.4(57.9,84.4) |
| Yunnan | 530623 | Yanjin County | 6.0 | 4.8 | 7.1 |  | 77.9(68.9,85.6) | 81.4(72.6,88.5) | 72.8(61.3,80.9) |
| Yunnan | 532329 | Wuding County | 6.4 | 5.4 | 7.4 |  | 77.4(66.6,85.6) | 78.3(67.3,86.8) | 76.5(64.3,85.9) |
| Yunnan | 530922 | Yun County | 6.1 | 4.9 | 7.3 |  | 76.6(66.2,84.9) | 79.4(68.6,87.2) | 71.0(59.6,80.1) |
| Yunnan | 530629 | Weixin County | 5.6 | 4.6 | 6.5 |  | 76.5(66.5,84.2) | 75.1(64.5,84.0) | 78.1(67.9,86.2) |
| Yunnan | 530326 | Huize County | 6.9 | 5.9 | 7.8 |  | 76.4(67.2,83.3) | 75.4(66.3,82.8) | 76.4(68.1,84.0) |
| Yunnan | 530125 | Yiliang County | 6.4 | 5.8 | 6.9 |  | 75.6(66.1,83.5) | 74.6(63.3,84.3) | 77.3(66.7,85.7) |
| Yunnan | 532931 | Jianchuan County | 6.5 | 5.9 | 7.1 |  | 75.5(63.5,85.6) | 77.9(64.5,88.7) | 71.6(58.4,82.2) |
| Yunnan | 530102 | Wuhua District | 4.3 | 3.6 | 4.9 |  | 75.4(57.3,89.9) | 73.8(55.9,89.2) | 78.9(60.6,92.2) |
| Yunnan | 530722 | Yongsheng County | 6.2 | 5.3 | 7.0 |  | 75.3(64.7,84.7) | 76.8(64.5,86.0) | 73.3(61.6,82.6) |
| Yunnan | 532531 | Luchun County | 6.0 | 4.7 | 7.2 |  | 75.2(65.0,83.7) | 78.9(68.0,87.6) | 68.3(56.0,79.0) |
| Yunnan | 532528 | Yuanyang County | 6.3 | 5.2 | 7.3 |  | 74.9(65.1,82.4) | 77.1(68.1,84.4) | 71.2(61.2,80.5) |
| Yunnan | 530926 | Zizhixian | 6.0 | 4.6 | 7.3 |  | 74.5(58.4,87.9) | 76.1(61.1,88.6) | 72.4(55.1,86.7) |
| Yunnan | 530824 | Zizhixian | 6.4 | 5.8 | 7.0 |  | 73.9(65.0,82.1) | 70.8(60.4,79.5) | 79.4(69.0,87.6) |
| Yunnan | 530921 | Fengqing County | 6.2 | 5.2 | 7.1 |  | 72.2(56.1,86.8) | 74.8(56.7,88.5) | 69.4(52.2,84.3) |
| Yunnan | 532529 | Honghe County | 6.2 | 5.0 | 7.2 |  | 66.4(48.8,80.6) | 69.0(50.0,83.7) | 60.8(44.1,76.7) |
| Yunnan | 533422 | Deeqeen County | 6.2 | 5.9 | 6.5 |  | 62.3(45.8,78.9) | 69.7(47.4,87.7) | 50.9(35.0,67.3) |
| Yunnan | 530927 | Zizhixian | 6.0 | 4.6 | 7.4 |  | 52.1(40.6,63.8) | 50.9(39.0,62.4) | 53.0(38.9,67.2) |
| Zhejiang | 330203 | Haishu District | 4.5 | 4.0 | 4.9 |  | 97.1(92.0,99.2) | 97.2(91.5,99.5) | 96.0(88.1,99.1) |
| Zhejiang | 330105 | Gongshu District | 3.9 | 3.5 | 4.2 |  | 96.9(89.7,99.3) | 95.6(85.7,99.3) | 97.0(86.4,99.7) |
| Zhejiang | 330602 | Yuecheng District | 5.2 | 4.4 | 5.9 |  | 96.8(93.1,98.8) | 96.5(91.3,98.8) | 96.4(91.2,98.7) |
| Zhejiang | 330106 | Xihu District | 3.4 | 3.0 | 3.7 |  | 96.6(90.7,99.0) | 95.2(86.4,98.7) | 96.9(89.6,99.4) |
| Zhejiang | 330102 | Shangcheng District | 6.3 | 5.8 | 6.8 |  | 96.2(87.1,99.4) | 94.9(80.9,99.4) | 96.1(80.0,99.7) |
| Zhejiang | 330103 | Xiacheng District | 4.5 | 4.1 | 4.9 |  | 95.5(87.2,99.1) | 93.0(79.8,98.5) | 96.7(85.1,99.5) |
| Zhejiang | 330328 | Wencheng County | 10.0 | 7.4 | 12.4 |  | 94.7(88.7,97.8) | 93.2(85.2,97.6) | 92.9(82.4,97.9) |
| Zhejiang | 330329 | Taishun County | 8.8 | 6.5 | 10.9 |  | 94.4(89.5,97.7) | 92.3(84.8,97.2) | 94.3(86.0,98.3) |
| Zhejiang | 330282 | Cixi City | 5.0 | 4.6 | 5.4 |  | 94.3(89.2,97.3) | 93.3(86.8,97.4) | 94.8(89.4,98.0) |
| Zhejiang | 330902 | Dinghai District | 5.8 | 5.3 | 6.2 |  | 94.1(86.5,97.9) | 90.7(79.8,96.7) | 95.8(88.4,99.1) |
| Zhejiang | 330723 | Wuyi County | 7.0 | 5.9 | 8.0 |  | 93.9(87.7,97.3) | 93.6(86.3,97.7) | 92.2(83.1,97.5) |
| Zhejiang | 330682 | Shangyu City | 7.1 | 5.9 | 8.2 |  | 93.8(87.9,97.1) | 91.5(83.8,96.1) | 94.3(86.2,98.1) |
| Zhejiang | 331022 | Sanmen County | 7.5 | 6.6 | 8.4 |  | 93.7(88.5,97.0) | 91.7(84.3,96.3) | 94.3(86.6,98.4) |
| Zhejiang | 330624 | Xinchang County | 8.5 | 7.1 | 10.0 |  | 93.1(87.2,96.7) | 95.6(89.0,98.6) | 84.9(75.4,91.7) |
| Zhejiang | 330402 | Nanhu District | 5.6 | 5.1 | 6.1 |  | 93.0(86.3,97.0) | 92.8(84.3,97.7) | 91.4(82.4,96.4) |
| Zhejiang | 330104 | Jiangan District | 3.2 | 3.0 | 3.4 |  | 92.9(84.8,97.5) | 88.4(75.7,95.8) | 96.1(87.8,99.2) |
| Zhejiang | 330185 | Lin'an City | 6.2 | 5.6 | 6.7 |  | 92.9(86.3,96.9) | 93.5(85.5,97.5) | 90.7(81.8,96.0) |
| Zhejiang | 330283 | Fenghua City | 6.7 | 5.9 | 7.5 |  | 92.8(85.7,97.1) | 88.8(78.5,95.1) | 95.1(87.2,98.7) |
| Zhejiang | 330108 | Binjiang District | 2.2 | 2.0 | 2.4 |  | 92.5(78.9,98.2) | 85.5(67.8,96.5) | 96.6(82.6,99.7) |
| Zhejiang | 330781 | Lanxi City | 8.6 | 7.2 | 10.0 |  | 92.0(86.3,95.6) | 91.6(85.3,95.7) | 89.9(81.2,94.7) |
| Zhejiang | 330825 | Longyou County | 7.5 | 6.2 | 8.7 |  | 91.8(84.6,96.3) | 92.9(80.9,96.8) | 89.5(78.1,95.9) |
| Zhejiang | 331023 | Tiantai County | 9.2 | 7.8 | 10.6 |  | 91.5(85.2,95.3) | 90.8(83.8,95.4) | 88.2(79.7,94.4) |
| Zhejiang | 330127 | Chun'an County | 8.7 | 7.9 | 9.5 |  | 91.4(84.4,95.8) | 91.5(82.0,96.5) | 88.8(79.9,94.8) |
| Zhejiang | 330327 | Cangnan County | 5.7 | 4.8 | 6.5 |  | 91.4(86.6,94.7) | 90.8(85.6,94.4) | 91.8(85.7,95.5) |
| Zhejiang | 330322 | Dongtou County | 9.4 | 7.9 | 11.0 |  | 91.3(81.5,96.8) | 90.0(80.8,96.4) | 89.8(73.5,98.5) |
| Zhejiang | 330110 | Yuhang District | 4.5 | 4.3 | 4.7 |  | 91.2(85.0,95.0) | 87.6(79.7,93.1) | 93.3(87.3,97.3) |
| Zhejiang | 330726 | Pujiang County | 6.0 | 5.5 | 6.4 |  | 91.2(84.1,95.7) | 83.5(73.2,90.8) | 96.1(88.9,99.0) |
| Zhejiang | 331124 | Songyang County | 9.4 | 7.4 | 11.3 |  | 91.1(83.8,96.1) | 87.6(77.1,94.3) | 91.4(81.4,97.0) |
| Zhejiang | 330109 | Xiaoshan District | 4.7 | 4.2 | 5.1 |  | 90.8(84.7,95.1) | 92.3(85.4,96.2) | 88.1(80.3,93.2) |
| Zhejiang | 330783 | Dongyang City | 6.6 | 5.9 | 7.3 |  | 90.3(83.9,94.6) | 89.8(82.4,94.6) | 88.6(80.2,94.5) |
| Zhejiang | 330523 | Anji County | 6.6 | 5.9 | 7.4 |  | 89.9(82.5,94.5) | 89.8(80.5,95.2) | 89.1(79.7,95.2) |
| Zhejiang | 331024 | Xianju County | 8.8 | 7.2 | 10.3 |  | 89.9(82.4,94.9) | 89.9(82.7,95.2) | 85.4(73.9,93.1) |
| Zhejiang | 330683 | Shengzhou City | 8.2 | 7.0 | 9.5 |  | 89.5(82.9,94.0) | 90.3(82.8,95.0) | 87.0(78.2,93.1) |
| Zhejiang | 330326 | Pingyang County | 6.3 | 5.3 | 7.3 |  | 89.3(82.7,93.6) | 88.9(82.2,93.8) | 90.0(83.1,94.7) |
| Zhejiang | 330381 | Rui'an City | 4.8 | 4.2 | 5.3 |  | 89.3(84.0,93.4) | 90.1(85.0,94.2) | 87.8(81.3,92.4) |
| Zhejiang | 330481 | Haining City | 5.5 | 5.3 | 5.8 |  | 89.3(80.9,94.7) | 86.7(76.6,93.8) | 90.7(81.4,96.3) |
| Zhejiang | 330225 | Xiangshan County | 6.9 | 5.7 | 8.0 |  | 88.8(82.0,94.2) | 88.1(79.1,93.7) | 89.4(80.1,95.2) |
| Zhejiang | 331081 | Wenling City | 6.0 | 5.6 | 6.4 |  | 88.7(83.3,93.0) | 87.1(79.9,92.2) | 89.8(83.4,94.2) |
| Zhejiang | 330922 | Shengsi County | 8.4 | 7.9 | 8.8 |  | 88.4(70.7,97.6) | 77.2(66.8,84.9) | 94.1(69.2,99.8) |
| Zhejiang | 330681 | Zhuji City | 6.7 | 5.7 | 7.7 |  | 88.3(82.1,92.4) | 88.0(81.7,93.0) | 88.1(81.2,92.8) |
| Zhejiang | 330483 | Tongxiang City | 6.2 | 5.4 | 7.0 |  | 88.2(81.5,93.4) | 89.8(82.3,94.8) | 85.0(74.9,91.7) |
| Zhejiang | 330921 | Daishan County | 7.2 | 7.3 | 7.1 |  | 88.2(74.7,96.3) | 86.1(69.3,96.9) | 87.8(69.9,97.6) |
| Zhejiang | 331181 | Longquan City | 8.0 | 6.4 | 9.5 |  | 88.2(79.6,93.7) | 89.8(79.5,95.8) | 84.6(74.9,91.5) |
| Zhejiang | 330212 | Yinzhou District | 3.6 | 3.2 | 3.9 |  | 88.1(80.4,93.4) | 86.6(77.9,92.7) | 90.1(82.9,94.9) |
| Zhejiang | 330727 | Pan'an County | 9.0 | 7.7 | 10.3 |  | 88.1(78.9,94.1) | 87.9(76.0,94.9) | 85.7(73.4,93.9) |
| Zhejiang | 330424 | Haiyan County | 6.0 | 5.7 | 6.3 |  | 87.8(78.2,94.3) | 87.9(75.4,95.8) | 85.8(72.7,94.3) |
| Zhejiang | 330122 | Tonglu County | 6.4 | 5.8 | 6.9 |  | 87.4(80.1,93.9) | 90.1(80.0,96.2) | 82.7(71.6,90.4) |
| Zhejiang | 331003 | Huangyan District | 6.2 | 5.6 | 6.8 |  | 87.3(79.3,92.6) | 87.7(79.1,94.1) | 86.3(76.4,92.6) |
| Zhejiang | 330302 | Lucheng District | 3.5 | 3.2 | 3.7 |  | 86.9(79.3,92.3) | 84.5(76.3,91.5) | 90.1(82.3,95.0) |
| Zhejiang | 330502 | Wuxing District | 5.0 | 4.5 | 5.6 |  | 86.8(78.0,93.2) | 78.2(67.2,87.4) | 92.3(83.0,97.5) |
| Zhejiang | 330421 | Jiashan County | 5.3 | 5.0 | 5.6 |  | 86.5(76.2,93.4) | 83.0(70.1,92.2) | 89.3(77.9,96.3) |
| Zhejiang | 330205 | Jiangbei District | 4.1 | 3.8 | 4.5 |  | 86.4(76.2,93.6) | 79.6(66.5,89.8) | 92.2(80.4,97.8) |
| Zhejiang | 330784 | Yongkang City | 5.1 | 4.6 | 5.5 |  | 86.4(78.9,91.8) | 83.9(75.8,90.5) | 88.2(80.3,93.7) |
| Zhejiang | 330824 | Kaihua County | 9.0 | 7.4 | 10.6 |  | 86.4(77.8,92.0) | 83.8(73.3,91.3) | 85.9(75.9,92.8) |
| Zhejiang | 331122 | Jinyun County | 8.0 | 6.7 | 9.2 |  | 86.2(77.5,92.4) | 85.5(75.0,92.5) | 83.3(72.5,91.5) |
| Zhejiang | 330503 | Nanxue District | 7.1 | 6.5 | 7.6 |  | 86.1(76.5,92.9) | 87.9(76.4,95.3) | 81.8(69.6,90.6) |
| Zhejiang | 331123 | Suichang County | 9.0 | 7.3 | 10.8 |  | 86.1(76.2,92.8) | 85.6(75.5,93.6) | 83.7(72.1,92.3) |
| Zhejiang | 331004 | Luqiao District | 4.4 | 4.2 | 4.6 |  | 85.8(77.5,92.4) | 84.8(73.5,92.2) | 86.9(76.9,93.3) |
| Zhejiang | 330183 | Fuyang City | 3.7 | 3.4 | 3.9 |  | 85.6(77.3,91.8) | 80.5(70.1,88.8) | 89.3(80.1,95.3) |
| Zhejiang | 330281 | Yuyao City | 5.8 | 5.1 | 6.6 |  | 85.2(77.9,90.4) | 84.2(75.5,90.5) | 87.1(77.9,93.1) |
| Zhejiang | 330881 | Jiangshan City | 8.5 | 6.9 | 10.2 |  | 85.2(77.0,90.5) | 84.8(74.7,91.1) | 82.1(72.7,88.8) |
| Zhejiang | 330226 | Ninghai County | 5.6 | 5.0 | 6.2 |  | 84.6(76.0,90.4) | 82.0(73.3,88.8) | 87.8(79.0,93.5) |
| Zhejiang | 330211 | Zhenhai District | 3.5 | 3.0 | 4.0 |  | 84.5(72.3,93.2) | 86.1(70.9,95.4) | 82.8(68.8,92.8) |
| Zhejiang | 330522 | Changxing County | 6.3 | 5.3 | 7.1 |  | 84.3(74.7,91.1) | 80.2(69.5,88.6) | 86.3(73.9,94.4) |
| Zhejiang | 331082 | Linhai City | 6.8 | 5.9 | 7.6 |  | 84.3(77.7,89.5) | 83.4(75.0,88.8) | 84.8(76.0,90.9) |
| Zhejiang | 331002 | Jiaojiang District | 4.7 | 4.2 | 5.1 |  | 84.1(75.8,90.7) | 83.0(73.2,90.8) | 84.6(74.0,91.7) |
| Zhejiang | 330621 | Shaoxing County | 4.5 | 4.1 | 5.0 |  | 84.0(74.9,90.4) | 78.2(67.9,87.3) | 89.2(81.0,95.2) |
| Zhejiang | 330324 | Yongjia County | 6.0 | 5.4 | 6.5 |  | 83.4(76.7,88.9) | 78.1(69.9,84.7) | 89.3(81.8,94.3) |
| Zhejiang | 330803 | Qujiang District | 7.8 | 6.8 | 8.8 |  | 83.4(75.0,90.3) | 83.1(72.0,91.1) | 81.1(69.8,90.1) |
| Zhejiang | 330802 | Kecheng District | 5.0 | 4.2 | 5.8 |  | 83.0(71.5,91.3) | 85.6(73.5,94.5) | 75.9(61.5,87.7) |
| Zhejiang | 330382 | Yueqing City | 4.5 | 4.0 | 5.0 |  | 82.9(75.4,89.0) | 81.6(73.7,87.5) | 84.6(77.2,90.4) |
| Zhejiang | 331127 | Zizhixian | 9.3 | 7.4 | 11.1 |  | 82.9(72.6,90.7) | 74.6(63.7,82.4) | 87.5(77.0,95.4) |
| Zhejiang | 331125 | Yunhe County | 7.3 | 5.6 | 8.8 |  | 82.8(72.0,91.3) | 79.1(70.7,86.1) | 86.4(72.7,96.5) |
| Zhejiang | 330182 | Jiande City | 7.3 | 6.4 | 8.1 |  | 82.6(73.1,90.0) | 81.1(69.7,89.2) | 82.0(69.9,91.2) |
| Zhejiang | 330206 | Beilun District | 3.8 | 3.5 | 4.0 |  | 82.6(72.5,89.5) | 74.7(63.8,84.5) | 90.9(81.0,96.1) |
| Zhejiang | 331102 | Liandu District | 5.5 | 4.6 | 6.3 |  | 82.2(73.8,89.0) | 82.1(71.3,90.2) | 83.1(72.1,90.8) |
| Zhejiang | 330903 | Putuo District | 5.9 | 5.3 | 6.5 |  | 81.8(70.7,90.2) | 77.4(66.3,87.0) | 84.2(69.9,93.9) |
| Zhejiang | 331126 | Qingyuan County | 7.7 | 5.8 | 9.6 |  | 81.0(71.1,89.1) | 81.4(69.3,90.5) | 78.7(66.8,89.1) |
| Zhejiang | 330702 | Wucheng District | 4.8 | 4.1 | 5.5 |  | 80.5(71.4,87.7) | 78.6(69.2,86.3) | 82.8(72.0,90.3) |
| Zhejiang | 330822 | Changshan County | 8.2 | 6.9 | 9.4 |  | 80.4(70.9,88.0) | 80.3(68.3,89.6) | 77.9(66.4,87.1) |
| Zhejiang | 330482 | Pinghu City | 5.5 | 5.2 | 5.8 |  | 80.0(68.7,88.8) | 78.2(64.6,88.6) | 82.0(69.8,91.1) |
| Zhejiang | 330782 | Yiwu City | 3.4 | 3.1 | 3.7 |  | 79.2(70.2,85.9) | 76.7(67.0,85.1) | 81.9(72.3,89.0) |
| Zhejiang | 330521 | Deqing County | 6.1 | 5.6 | 6.6 |  | 78.8(68.4,86.7) | 80.4(69.4,88.9) | 76.9(66.3,87.0) |
| Zhejiang | 330303 | Longwan District | 2.5 | 2.4 | 2.6 |  | 78.7(66.2,88.7) | 76.3(62.7,87.1) | 84.5(73.4,92.9) |
| Zhejiang | 331121 | Qingtian County | 8.0 | 6.7 | 9.3 |  | 78.5(69.6,85.8) | 80.7(71.4,89.0) | 71.7(60.4,80.7) |
| Zhejiang | 331021 | Yuhuan County | 4.3 | 3.9 | 4.6 |  | 77.0(67.6,84.2) | 75.0(65.2,83.6) | 80.8(70.0,88.9) |
| Zhejiang | 330703 | Jindong District | 5.9 | 5.2 | 6.5 |  | 76.3(65.3,85.4) | 76.9(64.9,87.1) | 76.1(62.8,87.0) |
| Zhejiang | 330411 | Xiuzhou District | 4.0 | 3.8 | 4.2 |  | 73.3(62.5,82.0) | 71.6(60.1,82.2) | 78.2(67.5,87.0) |
| Zhejiang | 330304 | Ouhai District | 2.6 | 2.4 | 2.7 |  | 71.2(60.3,80.7) | 65.0(54.1,75.8) | 81.1(71.2,89.3) |
| Shandong | 370503 | Hekou District | 4.9 | 3.9 | 5.9 |  | 98.8(93.1,99.9) | 96.0(82.1,99.6) | 99.5(92.1,100.0) |
| Shandong | 370702 | Weicheng District | 5.9 | 5.2 | 6.6 |  | 98.0(94.0,99.5) | 98.5(94.0,99.7) | 95.3(87.7,98.7) |
| Shandong | 370685 | Zhaoyuan City | 9.8 | 8.6 | 11.0 |  | 97.8(94.4,99.2) | 97.8(93.9,99.4) | 96.3(90.2,98.9) |
| Shandong | 370611 | Fushan District | 7.7 | 7.1 | 8.2 |  | 96.9(91.8,99.2) | 97.1(90.5,99.5) | 96.1(88.6,99.1) |
| Shandong | 370783 | Shouguang City | 7.8 | 7.3 | 8.2 |  | 96.8(93.5,98.6) | 94.5(90.3,97.4) | 97.8(94.9,99.3) |
| Shandong | 370830 | Wenshang County | 7.4 | 6.4 | 8.3 |  | 96.8(93.6,98.6) | 95.3(91.5,97.8) | 97.4(93.8,99.2) |
| Shandong | 370911 | Daiyue District | 8.3 | 7.3 | 9.3 |  | 96.8(94.1,98.5) | 96.3(92.7,98.4) | 96.8(93.0,98.7) |
| Shandong | 370214 | Chengyang District | 4.9 | 4.2 | 5.5 |  | 96.7(91.6,98.9) | 96.0(90.1,98.9) | 96.4(89.7,99.2) |
| Shandong | 370612 | Muping District | 9.0 | 7.6 | 10.3 |  | 96.6(90.6,99.0) | 96.8(89.6,99.2) | 93.8(83.4,98.5) |
| Shandong | 370785 | Gaomi City | 7.9 | 7.2 | 8.6 |  | 96.4(87.8,99.5) | 93.7(84.1,98.4) | 97.8(91.1,99.7) |
| Shandong | 371102 | Donggang District | 6.0 | 5.0 | 6.9 |  | 96.2(92.5,98.4) | 95.7(91.4,98.3) | 95.8(90.8,98.4) |
| Shandong | 370703 | Hanting District | 6.8 | 6.1 | 7.4 |  | 96.0(90.5,98.8) | 96.8(90.7,99.4) | 92.4(81.2,97.8) |
| Shandong | 370725 | Changle County | 7.3 | 6.4 | 8.2 |  | 96.0(92.0,98.2) | 95.1(89.8,97.9) | 95.8(90.8,98.6) |
| Shandong | 370112 | Licheng District | 6.9 | 6.2 | 7.5 |  | 95.8(91.8,98.2) | 96.2(91.9,98.4) | 94.3(88.7,97.3) |
| Shandong | 370502 | Dongying District | 4.4 | 3.7 | 5.1 |  | 95.7(89.2,98.7) | 96.2(90.0,99.1) | 93.3(83.5,97.9) |
| Shandong | 371525 | Guan County | 8.1 | 7.3 | 8.9 |  | 95.7(92.8,97.5) | 94.4(90.7,96.7) | 97.0(94.3,98.6) |
| Shandong | 370687 | Haiyang City | 10.3 | 9.3 | 11.3 |  | 95.4(90.6,98.0) | 93.6(86.7,97.6) | 95.8(89.9,98.6) |
| Shandong | 370784 | Anqiu City | 6.8 | 5.9 | 7.6 |  | 95.2(90.9,97.7) | 94.5(89.5,97.6) | 95.2(89.8,98.2) |
| Shandong | 371311 | Luozhuang District | 7.5 | 7.0 | 7.9 |  | 94.8(90.9,97.4) | 91.5(86.1,95.2) | 97.4(94.4,99.0) |
| Shandong | 370323 | Yiyuan County | 7.2 | 6.2 | 8.2 |  | 94.5(89.5,97.6) | 94.3(87.7,97.9) | 93.1(85.9,97.3) |
| Shandong | 370102 | Lixia District | 5.3 | 4.9 | 5.8 |  | 94.3(87.3,98.1) | 90.7(81.6,96.4) | 96.6(89.4,99.3) |
| Shandong | 371702 | Mudan District | 9.0 | 8.1 | 9.8 |  | 94.3(90.7,96.6) | 93.1(89.1,95.9) | 94.7(90.9,97.0) |
| Shandong | 370281 | Jiaozhou City | 7.1 | 6.2 | 8.0 |  | 94.2(84.7,98.6) | 94.5(84.6,98.9) | 92.9(80.6,98.4) |
| Shandong | 370983 | Feicheng City | 8.0 | 7.1 | 8.9 |  | 94.2(90.4,96.7) | 94.4(90.4,97.0) | 93.7(88.8,96.6) |
| Shandong | 370283 | Pingdu City | 8.1 | 7.0 | 9.2 |  | 94.1(90.1,96.8) | 93.3(88.7,96.3) | 93.5(88.1,96.9) |
| Shandong | 370681 | Longkou City | 7.9 | 6.8 | 8.9 |  | 94.1(88.6,97.4) | 94.3(88.5,98.0) | 91.0(83.2,96.1) |
| Shandong | 370921 | Ningyang County | 8.2 | 7.1 | 9.3 |  | 94.1(90.0,97.1) | 95.3(91.2,98.0) | 90.8(84.4,95.0) |
| Shandong | 371202 | Laicheng District | 7.3 | 6.5 | 8.2 |  | 94.1(88.6,97.4) | 94.7(89.5,97.7) | 92.1(85.2,96.3) |
| Shandong | 370282 | Jimo City | 7.8 | 6.7 | 8.8 |  | 94.0(89.0,97.0) | 93.8(88.5,97.0) | 92.7(87.4,96.3) |
| Shandong | 371302 | Lanshan District | 5.1 | 4.6 | 5.6 |  | 94.0(89.6,97.0) | 92.6(87.2,96.2) | 94.4(89.0,97.4) |
| Shandong | 370724 | Linqu County | 7.4 | 6.9 | 8.0 |  | 93.9(89.7,96.6) | 92.5(87.6,96.0) | 94.3(89.4,97.2) |
| Shandong | 371502 | Dongchangfu District | 6.7 | 5.8 | 7.5 |  | 93.9(89.6,96.4) | 93.9(90.1,96.8) | 92.9(87.8,96.4) |
| Shandong | 370683 | Laizhou City | 9.5 | 8.5 | 10.4 |  | 93.5(88.1,96.8) | 94.9(88.6,98.1) | 88.9(82.2,93.7) |
| Shandong | 370902 | Taishan District | 5.3 | 4.7 | 5.9 |  | 92.8(86.6,97.1) | 88.4(80.0,94.8) | 95.6(88.9,98.8) |
| Shandong | 370782 | Zhucheng City | 7.5 | 6.7 | 8.3 |  | 92.7(87.4,95.9) | 90.2(83.3,94.5) | 94.1(88.7,97.4) |
| Shandong | 370882 | Yanzhou City | 6.9 | 6.1 | 7.6 |  | 92.7(80.2,98.5) | 92.9(81.2,98.3) | 90.8(77.1,97.8) |
| Shandong | 370211 | Huangdao District | 5.9 | 5.3 | 6.4 |  | 92.6(86.7,95.9) | 89.0(82.0,94.0) | 94.9(89.8,97.8) |
| Shandong | 371521 | Yanggu County | 7.2 | 6.4 | 8.0 |  | 92.6(87.8,95.9) | 91.9(87.3,95.7) | 92.6(86.5,96.3) |
| Shandong | 370403 | Xuecheng District | 7.0 | 6.1 | 7.8 |  | 92.5(86.0,96.2) | 91.4(84.7,95.9) | 92.3(84.5,96.9) |
| Shandong | 370213 | Licang District | 4.5 | 3.9 | 5.0 |  | 92.4(83.3,97.4) | 94.8(87.1,98.7) | 84.8(71.5,93.6) |
| Shandong | 370521 | Kenli County | 6.1 | 5.6 | 6.6 |  | 92.4(73.6,99.0) | 93.3(74.1,99.3) | 88.6(68.1,98.4) |
| Shandong | 371312 | Hedong District | 7.1 | 6.8 | 7.4 |  | 92.3(87.4,95.7) | 87.9(80.4,93.0) | 95.4(91.0,97.9) |
| Shandong | 370103 | Shizhong District | 5.3 | 4.8 | 5.8 |  | 92.1(84.9,96.7) | 88.9(79.3,95.2) | 94.0(86.2,98.1) |
| Shandong | 370212 | Laoshan District | 4.1 | 3.7 | 4.6 |  | 92.1(81.5,97.6) | 87.4(73.6,96.2) | 95.1(82.5,99.3) |
| Shandong | 370828 | Jinxiang County | 6.6 | 6.4 | 6.7 |  | 91.7(85.0,95.6) | 88.6(80.8,94.2) | 93.4(87.1,97.1) |
| Shandong | 371426 | Pingyuan County | 6.9 | 6.3 | 7.4 |  | 91.5(84.5,96.1) | 89.0(80.1,95.5) | 91.8(84.6,96.6) |
| Shandong | 370881 | Qufu City | 7.3 | 6.3 | 8.2 |  | 91.4(85.6,95.2) | 89.4(82.8,94.4) | 92.6(85.6,96.7) |
| Shandong | 370522 | Lijin County | 7.8 | 6.9 | 8.6 |  | 91.2(83.1,96.4) | 87.8(77.4,94.6) | 93.0(83.0,98.2) |
| Shandong | 370602 | Zhifu District | 5.5 | 4.5 | 6.4 |  | 91.2(84.0,95.7) | 92.1(84.6,96.9) | 87.7(77.9,95.0) |
| Shandong | 370705 | Kuiwen District | 3.5 | 3.1 | 3.9 |  | 91.2(81.0,97.1) | 84.0(70.5,93.5) | 95.7(85.9,99.2) |
| Shandong | 370781 | Qingzhou City | 6.8 | 6.1 | 7.5 |  | 91.1(85.0,95.3) | 89.5(82.4,94.7) | 91.0(83.1,95.6) |
| Shandong | 371326 | Pingyi County | 8.0 | 7.1 | 8.9 |  | 91.0(85.6,94.9) | 90.3(84.0,94.3) | 89.1(82.2,94.2) |
| Shandong | 371523 | Chiping County | 8.1 | 7.2 | 9.0 |  | 91.0(84.5,95.1) | 89.4(82.6,94.1) | 91.2(83.9,95.7) |
| Shandong | 370613 | Laishan District | 5.2 | 4.6 | 5.8 |  | 90.7(81.0,97.2) | 94.6(83.0,99.0) | 82.0(70.4,91.8) |
| Shandong | 370306 | Zhoucun District | 6.6 | 6.0 | 7.3 |  | 90.4(81.9,95.9) | 85.6(74.0,93.9) | 92.9(82.4,98.3) |
| Shandong | 370113 | Changqing District | 6.6 | 5.9 | 7.2 |  | 90.1(82.7,95.0) | 90.9(83.1,96.0) | 87.8(78.8,94.1) |
| Shandong | 370321 | Huantai County | 7.3 | 6.5 | 8.2 |  | 89.7(82.9,94.9) | 91.7(84.7,96.7) | 85.5(75.8,92.4) |
| Shandong | 371103 | Lanshan District | 6.9 | 6.1 | 7.7 |  | 89.7(81.3,95.1) | 88.0(78.3,94.5) | 90.0(77.9,95.9) |
| Shandong | 370832 | Liangshan County | 6.6 | 5.8 | 7.4 |  | 89.2(82.2,93.7) | 86.9(79.4,92.3) | 90.8(84.0,95.2) |
| Shandong | 370105 | Tianqiao District | 4.8 | 4.3 | 5.3 |  | 89.0(79.3,95.1) | 89.9(80.5,96.2) | 86.6(74.8,93.8) |
| Shandong | 370104 | Huaiyin District | 5.3 | 4.8 | 5.7 |  | 88.8(79.7,94.8) | 88.1(76.6,95.3) | 88.1(77.5,95.8) |
| Shandong | 370831 | Sishui County | 7.8 | 6.6 | 9.0 |  | 88.7(82.4,93.4) | 87.0(79.4,92.0) | 88.7(81.0,94.5) |
| Shandong | 371002 | Huancui District | 5.9 | 5.2 | 6.7 |  | 88.7(72.8,97.0) | 88.2(72.9,96.7) | 88.5(72.8,96.4) |
| Shandong | 371522 | Shen County | 7.5 | 6.5 | 8.3 |  | 88.6(82.0,92.5) | 87.8(81.4,92.4) | 89.1(82.8,93.7) |
| Shandong | 371323 | Yishui County | 8.4 | 7.3 | 9.6 |  | 88.5(82.6,93.1) | 89.1(82.9,93.6) | 84.4(75.3,90.3) |
| Shandong | 371482 | Yucheng City | 7.1 | 6.4 | 7.8 |  | 88.5(81.1,93.8) | 88.4(79.6,94.1) | 87.7(79.7,93.4) |
| Shandong | 370923 | Dongping County | 8.0 | 7.3 | 8.6 |  | 88.4(81.8,93.0) | 85.1(77.6,90.3) | 90.7(84.0,95.2) |
| Shandong | 371122 | Ju County | 6.7 | 5.8 | 7.7 |  | 88.4(82.4,93.4) | 87.3(80.0,92.4) | 87.9(79.0,94.0) |
| Shandong | 370203 | Shibei District | 6.4 | 5.7 | 7.1 |  | 88.0(79.7,93.3) | 88.6(80.0,94.5) | 86.7(76.4,93.4) |
| Shandong | 371329 | Linshu County | 6.8 | 5.9 | 7.7 |  | 88.0(80.6,93.3) | 87.8(79.9,93.1) | 85.7(76.0,92.7) |
| Shandong | 371322 | Tancheng County | 6.9 | 6.3 | 7.5 |  | 87.5(80.7,92.5) | 86.0(79.5,91.4) | 87.2(79.0,92.8) |
| Shandong | 370883 | Zoucheng City | 7.0 | 6.0 | 7.9 |  | 87.3(80.3,91.8) | 86.5(79.5,91.4) | 87.1(79.5,92.7) |
| Shandong | 370404 | Yicheng District | 6.9 | 6.3 | 7.5 |  | 87.2(79.3,92.8) | 87.0(78.0,93.0) | 86.8(77.6,93.2) |
| Shandong | 370704 | Fangzi District | 6.3 | 5.7 | 6.9 |  | 87.2(78.6,93.4) | 85.5(75.8,92.2) | 88.8(77.4,95.8) |
| Shandong | 371581 | Linqing City | 7.6 | 6.6 | 8.6 |  | 87.2(80.3,91.8) | 87.7(81.2,92.2) | 86.8(79.7,92.0) |
| Shandong | 370811 | Rencheng District | 5.1 | 4.6 | 5.6 |  | 87.1(79.7,92.2) | 88.3(80.9,93.6) | 83.8(74.8,90.7) |
| Shandong | 371721 | Cao County | 8.1 | 7.4 | 8.9 |  | 87.1(76.6,94.0) | 85.8(74.1,93.1) | 88.6(79.1,94.8) |
| Shandong | 370682 | Laiyang City | 8.7 | 7.7 | 9.6 |  | 87.0(79.7,92.0) | 83.0(74.2,89.1) | 89.1(81.3,94.0) |
| Shandong | 371524 | Dong'e County | 8.4 | 7.3 | 9.4 |  | 86.8(79.1,92.2) | 86.2(77.8,92.0) | 87.0(77.9,93.3) |
| Shandong | 370304 | Boshan District | 8.0 | 7.3 | 8.7 |  | 86.7(77.4,93.3) | 85.3(75.2,92.6) | 87.0(75.7,95.4) |
| Shandong | 371727 | Dingtao County | 7.9 | 6.8 | 9.0 |  | 86.7(80.2,91.5) | 86.8(79.8,91.5) | 85.6(77.5,91.4) |
| Shandong | 370285 | Laixi City | 8.2 | 7.3 | 9.1 |  | 86.4(78.4,91.9) | 82.2(73.4,88.8) | 89.7(82.4,94.6) |
| Shandong | 371526 | Gaotang County | 7.1 | 6.3 | 7.8 |  | 86.0(78.4,91.5) | 85.3(77.5,92.3) | 84.9(74.9,91.6) |
| Shandong | 370405 | Tai'erzhuang District | 6.6 | 6.1 | 7.0 |  | 85.6(77.0,92.7) | 84.0(72.8,91.6) | 87.3(76.1,94.2) |
| Shandong | 371121 | Wulian County | 6.8 | 5.7 | 7.8 |  | 85.6(75.9,92.6) | 88.0(77.7,94.8) | 77.0(63.2,88.2) |
| Shandong | 370982 | Xintai City | 7.5 | 6.5 | 8.4 |  | 85.5(72.0,94.4) | 86.9(74.8,95.2) | 82.4(66.9,93.1) |
| Shandong | 371424 | Linyi County | 7.1 | 6.3 | 7.9 |  | 85.5(77.9,90.9) | 86.6(78.8,92.5) | 83.0(72.7,90.3) |
| Shandong | 370305 | Linzi District | 6.4 | 5.6 | 7.1 |  | 85.4(76.0,92.1) | 82.2(71.6,89.9) | 87.3(76.4,95.0) |
| Shandong | 370303 | Zhangdian District | 5.2 | 4.7 | 5.6 |  | 85.3(76.7,91.9) | 84.2(74.0,91.9) | 85.9(76.2,92.9) |
| Shandong | 371481 | Leling City | 8.0 | 7.2 | 8.7 |  | 85.1(77.2,90.4) | 85.3(78.3,90.7) | 83.1(74.9,89.5) |
| Shandong | 370322 | Gaoqing County | 7.2 | 6.3 | 8.1 |  | 84.9(75.9,91.9) | 87.3(78.2,94.0) | 80.7(68.4,89.9) |
| Shandong | 370829 | Jiaxiang County | 6.5 | 6.0 | 6.9 |  | 84.9(78.2,90.3) | 83.1(75.1,88.9) | 87.2(80.4,92.8) |
| Shandong | 371624 | Zhanhua County | 7.2 | 6.5 | 7.9 |  | 84.9(75.1,91.7) | 84.2(74.0,91.5) | 84.5(72.8,93.1) |
| Shandong | 371626 | Zouping County | 6.5 | 6.0 | 7.0 |  | 84.1(76.9,90.5) | 83.8(75.6,90.3) | 84.1(74.1,90.6) |
| Shandong | 371428 | Wucheng County | 7.1 | 6.6 | 7.5 |  | 83.9(74.8,90.9) | 80.7(69.9,88.7) | 87.1(78.1,93.3) |
| Shandong | 370181 | Zhangqiu City | 7.3 | 6.7 | 8.0 |  | 83.8(75.9,89.8) | 82.1(74.2,88.4) | 84.3(74.6,90.2) |
| Shandong | 371422 | Ningjin County | 7.9 | 6.8 | 9.1 |  | 83.8(75.7,89.7) | 84.6(76.4,90.2) | 82.7(73.5,89.7) |
| Shandong | 370523 | Guangrao County | 6.7 | 6.2 | 7.1 |  | 82.9(66.5,95.2) | 79.1(61.7,92.3) | 85.7(67.2,96.5) |
| Shandong | 370826 | Weishan County | 6.8 | 6.3 | 7.2 |  | 82.9(75.0,89.6) | 81.5(72.3,88.5) | 84.4(74.9,91.5) |
| Shandong | 371724 | Juye County | 7.4 | 6.6 | 8.2 |  | 82.6(74.6,88.3) | 80.7(72.7,87.2) | 83.9(76.1,90.1) |
| Shandong | 370686 | Qixia City | 10.4 | 9.5 | 11.3 |  | 82.5(74.0,88.6) | 77.5(68.2,84.7) | 85.7(77.7,91.7) |
| Shandong | 371602 | BinCheng District | 5.5 | 4.7 | 6.3 |  | 82.2(73.8,89.9) | 86.5(77.7,92.6) | 76.2(65.6,85.2) |
| Shandong | 371425 | Qihe County | 6.5 | 5.8 | 7.1 |  | 81.9(72.9,88.3) | 80.2(71.4,87.2) | 84.8(77.1,91.0) |
| Shandong | 371623 | Wudi County | 7.2 | 6.5 | 7.8 |  | 81.8(73.0,88.9) | 81.0(72.3,87.9) | 82.1(72.5,89.5) |
| Shandong | 370302 | Zichuan District | 6.0 | 5.3 | 6.7 |  | 81.3(70.9,88.6) | 83.3(73.7,90.4) | 76.6(64.4,86.4) |
| Shandong | 370202 | Shinan District | 5.8 | 5.1 | 6.4 |  | 81.0(69.0,89.9) | 82.9(70.8,91.7) | 77.1(61.7,90.7) |
| Shandong | 371324 | Cangshan County | 6.4 | 5.8 | 6.9 |  | 80.9(73.4,87.2) | 80.8(72.6,87.3) | 81.5(73.2,88.2) |
| Shandong | 371083 | Rushan City | 9.3 | 8.0 | 10.5 |  | 80.7(68.5,89.1) | 72.7(59.8,83.4) | 84.1(71.5,92.6) |
| Shandong | 370124 | Pingyin County | 7.7 | 6.9 | 8.5 |  | 80.3(62.8,91.4) | 78.5(61.6,90.1) | 79.4(60.2,92.2) |
| Shandong | 371203 | Gangcheng District | 5.9 | 5.0 | 6.8 |  | 80.3(68.8,89.2) | 85.9(73.5,94.4) | 73.2(62.3,81.7) |
| Shandong | 371621 | Huimin County | 7.3 | 6.5 | 8.1 |  | 80.1(70.3,87.9) | 81.2(71.8,88.2) | 77.5(66.2,85.9) |
| Shandong | 370786 | Changyi City | 7.6 | 7.0 | 8.2 |  | 80.0(70.5,87.8) | 80.5(70.3,88.4) | 77.0(66.6,85.9) |
| Shandong | 370481 | Tengzhou City | 6.1 | 5.6 | 6.5 |  | 79.9(71.1,86.6) | 78.9(70.3,85.7) | 79.2(70.0,86.6) |
| Shandong | 370634 | Changdao County | 7.3 | 6.6 | 7.9 |  | 79.5(54.9,96.9) | 64.5(50.2,76.4) | 89.1(51.2,99.9) |
| Shandong | 371082 | Rongcheng City | 9.0 | 7.9 | 10.1 |  | 79.3(68.6,87.4) | 73.8(62.1,83.3) | 82.2(71.5,91.3) |
| Shandong | 371327 | Junan County | 8.0 | 7.1 | 8.8 |  | 79.3(70.2,85.7) | 76.6(66.3,84.4) | 80.4(70.6,87.5) |
| Shandong | 370402 | Shizhong District | 5.8 | 4.7 | 6.8 |  | 79.0(69.8,87.0) | 83.3(73.9,90.2) | 73.6(62.7,82.4) |
| Shandong | 371725 | Yuncheng County | 7.3 | 6.5 | 8.2 |  | 78.1(68.9,85.4) | 76.4(66.5,84.0) | 78.6(67.7,86.5) |
| Shandong | 371726 | Juancheng County | 7.3 | 6.3 | 8.3 |  | 77.6(69.1,84.8) | 79.5(70.7,86.3) | 73.1(61.3,82.5) |
| Shandong | 371421 | Ling County | 6.7 | 6.0 | 7.3 |  | 77.3(68.1,84.8) | 75.8(65.2,84.6) | 78.3(68.4,86.1) |
| Shandong | 371427 | Xiajin County | 6.7 | 6.0 | 7.4 |  | 76.8(66.4,84.7) | 75.9(65.9,84.0) | 78.7(68.7,86.9) |
| Shandong | 370406 | Shanting District | 6.5 | 5.9 | 7.0 |  | 76.4(56.4,91.5) | 76.2(55.2,91.6) | 75.7(54.4,91.1) |
| Shandong | 371728 | Dongming County | 7.0 | 6.0 | 7.9 |  | 76.0(66.3,83.0) | 75.4(66.4,82.7) | 76.9(67.0,84.8) |
| Shandong | 371625 | Boxing County | 6.5 | 5.7 | 7.2 |  | 75.9(64.9,84.6) | 76.5(65.5,85.2) | 74.6(60.9,84.6) |
| Shandong | 370125 | Jiyang County | 6.8 | 6.4 | 7.3 |  | 74.8(64.9,82.4) | 72.1(61.2,80.8) | 77.7(67.5,86.0) |
| Shandong | 370684 | Penglai City | 8.1 | 7.0 | 9.2 |  | 74.7(63.3,83.8) | 68.3(56.4,77.4) | 78.6(65.4,89.7) |
| Shandong | 370827 | Yutai County | 5.4 | 5.0 | 5.7 |  | 74.7(64.6,83.8) | 77.0(65.4,86.3) | 70.9(58.7,81.0) |
| Shandong | 370126 | Shanghe County | 7.1 | 6.4 | 7.8 |  | 74.1(64.9,82.7) | 72.9(63.4,81.5) | 75.1(64.6,83.7) |
| Shandong | 371081 | Wendeng City | 8.2 | 7.2 | 9.2 |  | 73.4(52.6,89.7) | 74.7(52.0,93.2) | 65.7(44.8,83.3) |
| Shandong | 371321 | Yinan County | 7.2 | 6.2 | 8.1 |  | 72.3(61.8,80.7) | 73.2(64.2,81.1) | 69.8(59.2,79.1) |
| Shandong | 371723 | Chengwu County | 5.8 | 5.2 | 6.4 |  | 71.1(60.7,80.7) | 69.0(58.5,78.3) | 74.4(62.4,83.9) |
| Shandong | 371622 | Yangxin County | 6.5 | 6.0 | 6.9 |  | 67.8(56.8,77.4) | 66.1(54.4,77.4) | 70.4(59.2,80.6) |
| Shandong | 371402 | DeCheng District | 3.9 | 3.2 | 4.5 |  | 67.5(54.9,78.8) | 66.1(53.3,77.8) | 70.6(56.5,82.9) |
| Shandong | 371722 | Shan County | 8.1 | 7.5 | 8.6 |  | 66.6(55.6,75.1) | 64.0(53.0,73.3) | 68.7(58.0,77.7) |
| Shandong | 371423 | Qingyun County | 6.6 | 6.1 | 7.0 |  | 66.2(56.2,75.4) | 67.6(56.3,77.6) | 66.1(55.1,75.4) |
| Shandong | 371325 | Fei County | 6.2 | 5.6 | 6.7 |  | 65.0(54.4,74.4) | 61.1(50.1,71.4) | 69.1(56.7,79.7) |
| Shandong | 371328 | Mengyin County | 7.0 | 5.9 | 8.0 |  | 59.3(46.7,69.8) | 60.4(48.1,70.6) | 56.6(43.9,68.4) |
| Hunan | 430105 | Kaifu District | 5.6 | 4.9 | 6.3 |  | 98.0(90.7,99.8) | 96.9(87.7,99.6) | 98.3(90.9,99.9) |
| Hunan | 430121 | Changsha County | 5.8 | 5.0 | 6.4 |  | 98.0(95.7,99.2) | 95.7(91.5,98.3) | 98.9(96.8,99.7) |
| Hunan | 431302 | Louxing District | 6.0 | 4.4 | 7.6 |  | 97.3(90.2,99.6) | 98.5(93.6,99.8) | 90.8(78.3,97.9) |
| Hunan | 430602 | Yueyanglou District | 5.9 | 4.8 | 7.0 |  | 96.2(92.3,98.5) | 96.8(92.5,98.9) | 94.9(88.7,98.2) |
| Hunan | 430781 | Jinshi City | 6.6 | 5.9 | 7.3 |  | 96.0(87.4,99.2) | 91.7(77.8,98.1) | 98.0(89.1,99.8) |
| Hunan | 430181 | Liuyang City | 7.5 | 6.5 | 8.5 |  | 93.9(90.2,96.4) | 92.0(87.3,95.4) | 94.9(91.0,97.4) |
| Hunan | 430104 | Yuelu District | 4.2 | 3.5 | 5.0 |  | 93.8(82.2,98.9) | 90.7(77.3,97.5) | 96.4(86.8,99.5) |
| Hunan | 430211 | Tianyuan District | 5.7 | 4.6 | 6.8 |  | 93.8(87.1,97.7) | 94.6(87.4,98.6) | 90.6(79.2,97.0) |
| Hunan | 430124 | Ningxiang County | 7.3 | 6.1 | 8.5 |  | 93.4(89.6,96.2) | 94.1(90.1,96.9) | 90.9(85.5,94.9) |
| Hunan | 430202 | Hetang District | 6.1 | 4.9 | 7.2 |  | 93.0(84.3,97.4) | 90.8(80.4,96.8) | 94.5(83.2,98.8) |
| Hunan | 430681 | Miluo City | 7.2 | 5.9 | 8.3 |  | 93.0(87.8,96.4) | 93.0(87.0,96.6) | 92.3(85.2,96.3) |
| Hunan | 430112 | Wangcheng County | 6.1 | 5.1 | 7.1 |  | 92.2(86.9,95.8) | 90.3(83.1,95.5) | 92.9(86.1,97.3) |
| Hunan | 431002 | Beihu District | 6.3 | 5.2 | 7.4 |  | 92.0(83.7,96.7) | 93.1(85.0,97.5) | 89.7(79.2,95.9) |
| Hunan | 430902 | Ziyang District | 6.5 | 5.8 | 7.2 |  | 91.9(85.3,96.3) | 90.5(82.0,95.5) | 92.4(84.4,97.1) |
| Hunan | 430321 | Xiangtan County | 9.0 | 7.3 | 10.6 |  | 91.8(87.6,94.8) | 92.0(87.2,95.4) | 88.2(81.2,92.7) |
| Hunan | 430102 | Furong District | 5.0 | 4.2 | 5.7 |  | 91.6(79.1,97.7) | 91.3(78.4,98.0) | 91.2(78.7,97.8) |
| Hunan | 430725 | Taoyuan County | 7.6 | 6.5 | 8.7 |  | 91.2(85.7,95.2) | 89.3(81.1,94.4) | 91.7(84.8,96.2) |
| Hunan | 431081 | Zixing City | 7.1 | 6.1 | 8.1 |  | 90.9(84.1,95.2) | 91.5(83.8,96.2) | 88.0(78.6,94.2) |
| Hunan | 430722 | Hanshou County | 6.5 | 5.7 | 7.4 |  | 90.7(84.4,94.9) | 87.8(80.0,93.2) | 92.6(85.6,96.8) |
| Hunan | 431027 | Guidong County | 6.2 | 5.4 | 6.9 |  | 90.4(81.9,95.5) | 88.6(79.4,95.3) | 91.3(80.6,97.0) |
| Hunan | 431025 | Linwu County | 7.0 | 6.2 | 7.8 |  | 89.8(84.2,94.0) | 85.6(78.7,91.0) | 94.5(89.6,97.3) |
| Hunan | 430723 | Li County | 6.6 | 5.4 | 7.8 |  | 89.5(83.6,94.4) | 90.8(83.3,95.6) | 85.4(76.4,92.0) |
| Hunan | 430626 | Pingjiang County | 7.1 | 5.8 | 8.3 |  | 89.4(84.5,93.2) | 90.3(84.6,94.1) | 87.1(80.0,91.7) |
| Hunan | 430221 | Zhuzhou County | 7.8 | 6.3 | 9.2 |  | 88.9(81.7,93.7) | 89.9(82.5,95.1) | 86.4(78.1,92.3) |
| Hunan | 430726 | Shimen County | 7.3 | 6.1 | 8.5 |  | 88.7(80.5,93.7) | 88.4(79.3,94.2) | 86.6(75.9,94.1) |
| Hunan | 430224 | Chaling County | 6.1 | 5.2 | 7.0 |  | 88.3(82.3,92.7) | 89.0(82.8,93.4) | 87.6(80.5,92.5) |
| Hunan | 431022 | Yizhang County | 6.5 | 5.7 | 7.3 |  | 88.3(82.6,92.4) | 86.1(79.7,91.0) | 91.1(84.9,95.0) |
| Hunan | 430724 | Linli County | 7.0 | 5.7 | 8.3 |  | 88.2(80.3,94.3) | 86.5(77.4,93.1) | 87.9(76.9,95.1) |
| Hunan | 431228 | Zizhixian | 8.4 | 7.4 | 9.3 |  | 88.1(81.4,92.7) | 87.1(79.2,93.0) | 87.1(79.4,93.1) |
| Hunan | 433123 | Fenghuang County | 7.0 | 6.3 | 7.7 |  | 88.0(80.6,93.2) | 82.2(72.4,89.9) | 92.3(84.8,97.0) |
| Hunan | 430223 | You County | 7.3 | 6.3 | 8.3 |  | 87.8(81.9,92.0) | 88.0(81.6,92.3) | 87.4(81.1,91.9) |
| Hunan | 431281 | Hongjiang City | 7.2 | 6.9 | 7.5 |  | 87.8(79.8,93.4) | 84.9(74.8,92.4) | 89.1(81.4,94.8) |
| Hunan | 431023 | Yongxing County | 6.2 | 5.1 | 7.1 |  | 87.6(80.8,92.5) | 84.7(77.0,90.1) | 90.9(83.9,95.5) |
| Hunan | 430281 | Liling City | 6.5 | 5.3 | 7.5 |  | 87.1(81.3,91.6) | 85.7(78.5,90.6) | 88.9(82.8,93.3) |
| Hunan | 431024 | Jiahe County | 6.2 | 5.6 | 6.7 |  | 87.0(79.6,92.2) | 83.5(74.8,90.1) | 90.6(82.5,95.7) |
| Hunan | 431225 | Huitong County | 7.1 | 6.4 | 7.8 |  | 86.9(78.4,92.5) | 83.1(72.9,90.2) | 89.0(78.5,95.6) |
| Hunan | 430103 | Tianxin District | 5.7 | 4.8 | 6.6 |  | 86.8(72.4,95.0) | 84.4(70.0,93.4) | 88.9(74.5,96.9) |
| Hunan | 430408 | Zhengxiang District | 4.8 | 4.0 | 5.6 |  | 86.3(75.7,93.4) | 84.1(73.1,91.7) | 89.7(78.1,96.6) |
| Hunan | 430111 | Yuhua District | 4.9 | 4.0 | 5.7 |  | 86.2(77.3,92.9) | 84.5(75.4,91.8) | 87.1(78.0,93.9) |
| Hunan | 431003 | Suxian District | 6.0 | 5.0 | 6.9 |  | 85.9(77.9,91.7) | 86.9(79.3,93.0) | 84.2(74.9,90.8) |
| Hunan | 430821 | Cili County | 7.6 | 6.8 | 8.2 |  | 85.6(78.2,91.7) | 85.4(76.2,91.8) | 84.5(75.6,91.4) |
| Hunan | 430204 | Shifeng District | 5.0 | 4.1 | 5.9 |  | 85.3(71.0,94.7) | 85.5(68.1,95.9) | 84.3(66.3,96.0) |
| Hunan | 431202 | Hecheng District | 5.2 | 3.7 | 6.6 |  | 85.3(77.2,91.4) | 87.2(78.5,92.9) | 82.2(71.2,90.7) |
| Hunan | 430424 | Hengdong County | 6.3 | 5.2 | 7.3 |  | 85.2(77.5,90.5) | 83.3(75.5,89.0) | 87.8(79.8,92.9) |
| Hunan | 431230 | Zizhixian | 6.9 | 6.3 | 7.5 |  | 85.2(75.8,91.8) | 83.1(72.2,91.2) | 86.2(75.8,93.9) |
| Hunan | 433124 | Huayuan County | 6.2 | 5.4 | 7.0 |  | 85.2(77.1,91.4) | 85.8(76.5,92.5) | 83.4(73.9,91.4) |
| Hunan | 431129 | Zizhixian | 7.5 | 6.1 | 8.8 |  | 85.1(77.6,90.2) | 83.9(76.8,89.2) | 86.7(78.9,92.4) |
| Hunan | 430407 | Shigu District | 5.9 | 5.3 | 6.5 |  | 85.0(70.9,94.4) | 85.4(71.0,95.4) | 85.7(73.1,94.9) |
| Hunan | 430802 | Yongding District | 5.9 | 5.2 | 6.6 |  | 85.0(76.5,91.6) | 82.1(72.6,89.5) | 88.4(79.3,94.5) |
| Hunan | 431028 | Anren County | 6.1 | 5.4 | 6.8 |  | 84.9(76.8,90.0) | 83.8(75.8,89.2) | 88.1(81.6,93.0) |
| Hunan | 431021 | Guiyang County | 6.3 | 5.7 | 6.8 |  | 84.7(77.4,89.8) | 82.4(75.1,88.2) | 87.5(81.0,92.2) |
| Hunan | 431127 | Lanshan County | 7.3 | 6.3 | 8.2 |  | 84.7(77.1,89.8) | 83.3(75.9,89.1) | 86.5(78.2,92.2) |
| Hunan | 430603 | Yunxi District | 4.9 | 4.3 | 5.4 |  | 84.4(69.8,93.9) | 78.8(62.7,92.1) | 89.4(72.2,97.9) |
| Hunan | 430304 | Yuetang District | 5.8 | 4.8 | 6.7 |  | 84.3(74.0,92.3) | 84.7(72.5,93.9) | 83.3(69.9,93.4) |
| Hunan | 430702 | Wuling District | 5.2 | 4.4 | 6.1 |  | 83.7(69.6,93.4) | 84.6(70.8,94.0) | 84.4(72.8,93.6) |
| Hunan | 431121 | Qiyang County | 6.7 | 5.7 | 7.7 |  | 83.6(76.4,89.0) | 82.3(73.7,88.2) | 84.4(77.0,90.3) |
| Hunan | 431103 | Lengshuitan District | 6.2 | 5.3 | 7.0 |  | 83.5(75.0,89.9) | 82.4(73.3,89.1) | 84.6(76.3,91.0) |
| Hunan | 430624 | Xiangyin County | 6.4 | 5.4 | 7.4 |  | 83.3(75.1,89.4) | 82.4(73.1,88.8) | 84.1(75.4,90.2) |
| Hunan | 431224 | Xupu County | 6.8 | 6.1 | 7.4 |  | 83.3(76.1,88.8) | 79.1(71.0,86.1) | 86.4(77.9,91.7) |
| Hunan | 430621 | Yueyang County | 6.0 | 5.2 | 6.6 |  | 82.6(74.9,88.4) | 82.5(74.5,88.7) | 83.4(75.1,89.8) |
| Hunan | 430682 | LinXiang City | 5.8 | 5.0 | 6.5 |  | 82.4(73.9,88.7) | 82.3(73.0,88.9) | 83.8(73.8,90.8) |
| Hunan | 431226 | Zizhixian | 6.4 | 5.4 | 7.3 |  | 82.1(72.7,89.3) | 82.1(72.2,89.5) | 79.5(67.2,89.2) |
| Hunan | 431229 | Dongzu Zizhixian | 5.8 | 5.2 | 6.3 |  | 82.1(72.0,89.5) | 81.8(70.9,90.6) | 81.8(69.6,90.5) |
| Hunan | 430406 | Yanfeng District | 5.8 | 4.8 | 6.7 |  | 81.9(65.5,94.2) | 80.9(64.7,93.5) | 82.3(64.7,95.0) |
| Hunan | 431123 | Shuangpai County | 6.2 | 4.7 | 7.5 |  | 81.7(71.0,89.7) | 86.3(74.5,94.0) | 70.6(58.0,81.7) |
| Hunan | 431124 | Dao County | 6.8 | 5.9 | 7.5 |  | 81.6(73.7,87.7) | 79.3(71.6,86.2) | 84.5(76.1,90.1) |
| Hunan | 430203 | Lusong District | 6.6 | 5.1 | 8.2 |  | 81.3(69.0,92.1) | 79.5(68.8,87.4) | 85.8(68.0,96.4) |
| Hunan | 430611 | Junshan District | 6.2 | 4.8 | 7.5 |  | 81.3(70.4,89.7) | 86.8(74.8,94.6) | 70.8(60.3,79.8) |
| Hunan | 431102 | Lingling District | 6.7 | 5.8 | 7.5 |  | 81.2(73.1,87.4) | 81.2(72.3,87.6) | 81.7(73.5,87.7) |
| Hunan | 431227 | Zizhixian | 7.3 | 6.4 | 8.0 |  | 81.2(71.2,88.9) | 79.8(67.3,88.5) | 80.2(66.7,90.2) |
| Hunan | 431322 | Xinhua County | 6.5 | 5.6 | 7.3 |  | 81.1(73.6,86.4) | 77.8(69.1,84.0) | 84.4(76.4,89.8) |
| Hunan | 431026 | Rucheng County | 6.3 | 5.2 | 7.2 |  | 80.9(71.4,87.6) | 77.7(68.7,85.2) | 85.1(75.0,92.0) |
| Hunan | 431381 | Lengshuijiang City | 5.7 | 4.7 | 6.5 |  | 80.9(66.0,91.4) | 78.5(63.2,89.6) | 84.0(68.9,94.2) |
| Hunan | 431382 | Lianyuan City | 6.2 | 5.4 | 7.0 |  | 80.6(72.9,87.1) | 81.2(73.4,87.6) | 78.4(68.8,85.2) |
| Hunan | 430923 | Anhua County | 5.9 | 4.9 | 6.9 |  | 80.5(68.6,89.3) | 78.0(64.8,87.5) | 82.5(69.6,91.6) |
| Hunan | 431126 | Ningyuan County | 5.9 | 5.2 | 6.6 |  | 80.4(72.1,87.1) | 79.9(71.8,87.2) | 80.4(70.0,87.7) |
| Hunan | 431223 | Chenxi County | 6.6 | 5.8 | 7.4 |  | 80.2(70.0,87.8) | 80.1(70.6,88.1) | 79.6(67.7,87.9) |
| Hunan | 430422 | Hengnan County | 6.2 | 5.5 | 6.9 |  | 80.1(72.1,86.6) | 78.1(69.8,84.4) | 83.3(75.1,88.8) |
| Hunan | 430405 | Zhuhui District | 6.0 | 4.9 | 7.1 |  | 79.9(65.4,91.2) | 83.8(66.9,93.7) | 74.5(61.0,86.6) |
| Hunan | 430482 | Changning City | 6.8 | 5.9 | 7.6 |  | 79.9(72.0,86.4) | 77.3(68.5,83.8) | 83.9(75.9,90.1) |
| Hunan | 430922 | Taojiang County | 6.3 | 5.5 | 7.1 |  | 79.7(70.7,86.5) | 77.0(67.8,84.5) | 83.7(74.7,90.1) |
| Hunan | 430581 | Wugang City | 6.5 | 5.5 | 7.4 |  | 79.5(71.3,85.9) | 79.3(70.9,86.1) | 79.2(69.7,86.6) |
| Hunan | 431321 | Shuangfeng County | 5.8 | 5.0 | 6.5 |  | 79.5(71.3,86.1) | 78.4(69.0,85.9) | 79.5(70.6,86.7) |
| Hunan | 430623 | Huarong County | 6.6 | 5.7 | 7.5 |  | 79.4(71.4,86.1) | 80.2(72.4,87.3) | 79.5(71.6,86.4) |
| Hunan | 430511 | Beita District | 5.2 | 4.0 | 6.5 |  | 79.3(62.3,93.1) | 77.1(61.3,90.9) | 82.6(59.1,96.2) |
| Hunan | 430302 | Yuhu District | 5.9 | 4.9 | 6.9 |  | 78.9(69.4,86.7) | 81.4(70.5,89.2) | 76.4(64.1,86.1) |
| Hunan | 430421 | Hengyang County | 6.8 | 5.9 | 7.6 |  | 77.9(69.7,84.5) | 75.3(66.5,82.0) | 81.8(74.2,87.8) |
| Hunan | 430703 | Dingcheng District | 6.0 | 5.2 | 6.9 |  | 77.3(66.9,84.9) | 79.5(68.1,87.6) | 74.1(62.7,83.7) |
| Hunan | 431128 | Xintian County | 6.1 | 5.3 | 6.8 |  | 77.2(67.7,84.5) | 78.1(68.3,85.4) | 78.2(68.2,87.0) |
| Hunan | 431122 | Dong'an County | 6.3 | 5.5 | 7.1 |  | 76.9(67.8,83.9) | 73.9(64.5,81.7) | 82.0(72.3,88.9) |
| Hunan | 431221 | Zhongfang County | 6.1 | 5.4 | 6.8 |  | 76.8(61.5,89.4) | 74.9(57.0,87.9) | 77.8(59.3,90.7) |
| Hunan | 431125 | Jiangyong County | 6.6 | 5.7 | 7.4 |  | 76.1(67.2,83.9) | 72.8(63.5,80.6) | 81.4(71.5,89.6) |
| Hunan | 430381 | Xiangxiang City | 6.1 | 5.0 | 7.2 |  | 76.0(66.6,84.1) | 77.5(68.7,85.4) | 72.7(61.6,81.1) |
| Hunan | 430522 | Xinshao County | 6.4 | 5.7 | 7.0 |  | 76.0(67.3,82.7) | 71.2(62.0,78.8) | 82.4(74.0,88.9) |
| Hunan | 430528 | Xinning County | 5.8 | 5.1 | 6.4 |  | 75.8(67.3,83.4) | 73.7(64.1,81.7) | 78.8(68.4,86.3) |
| Hunan | 433127 | Yongshun County | 6.2 | 5.5 | 6.9 |  | 75.7(66.4,82.7) | 74.5(64.5,82.5) | 76.2(66.4,84.7) |
| Hunan | 430523 | Shaoyang County | 5.9 | 5.3 | 6.4 |  | 75.6(67.6,82.4) | 72.3(63.5,79.4) | 80.5(72.2,87.1) |
| Hunan | 430822 | Sangzhi County | 6.8 | 5.9 | 7.6 |  | 75.3(65.6,83.7) | 71.5(60.9,80.2) | 78.7(67.3,87.8) |
| Hunan | 430423 | Hengshan County | 6.5 | 5.2 | 7.8 |  | 74.9(66.0,82.0) | 76.2(66.8,82.7) | 75.1(64.5,83.4) |
| Hunan | 433130 | Longshan County | 6.6 | 5.6 | 7.6 |  | 74.9(65.4,82.7) | 76.7(66.5,83.5) | 73.0(62.5,81.5) |
| Hunan | 431222 | Yuanling County | 5.9 | 5.3 | 6.5 |  | 74.8(64.6,82.7) | 74.6(64.8,84.1) | 73.3(62.4,82.8) |
| Hunan | 433125 | Baojing County | 6.1 | 5.0 | 7.2 |  | 74.7(63.5,83.8) | 79.5(67.8,88.4) | 64.9(53.0,75.6) |
| Hunan | 430721 | Anxiang County | 6.4 | 5.5 | 7.2 |  | 74.4(63.6,83.6) | 72.3(60.9,82.5) | 75.6(63.1,86.3) |
| Hunan | 430524 | Longhui County | 6.3 | 5.6 | 6.8 |  | 74.0(65.6,80.5) | 71.1(61.9,79.0) | 79.1(69.9,85.5) |
| Hunan | 433101 | Jishou City | 5.5 | 4.4 | 6.5 |  | 74.0(61.8,85.0) | 75.5(62.8,86.1) | 71.7(57.3,83.5) |
| Hunan | 430903 | Heshan District | 5.3 | 4.5 | 6.0 |  | 73.8(64.1,82.0) | 72.5(62.8,81.1) | 77.3(66.9,85.8) |
| Hunan | 430503 | Daxiang District | 6.4 | 5.5 | 7.2 |  | 73.2(56.9,84.9) | 74.8(59.0,87.2) | 71.7(57.5,82.1) |
| Hunan | 430481 | Leiyang City | 5.7 | 4.7 | 6.7 |  | 73.0(64.0,80.9) | 73.9(65.9,80.8) | 74.9(65.5,82.2) |
| Hunan | 430426 | Qidong County | 5.9 | 5.0 | 6.8 |  | 72.7(63.3,80.2) | 72.8(63.3,80.6) | 73.2(63.5,81.2) |
| Hunan | 430382 | Shaoshan City | 6.1 | 5.6 | 6.6 |  | 70.9(52.1,86.6) | 66.4(44.1,86.6) | 73.4(54.2,91.1) |
| Hunan | 430225 | yanling County | 5.1 | 4.7 | 5.5 |  | 70.5(58.0,80.4) | 69.9(56.8,81.2) | 72.6(60.1,83.8) |
| Hunan | 430521 | Shaodong County | 7.1 | 6.0 | 8.1 |  | 70.2(61.7,77.5) | 69.9(60.6,77.8) | 70.7(60.2,78.7) |
| Hunan | 433122 | Luxi County | 6.1 | 5.3 | 6.9 |  | 69.9(59.2,80.0) | 71.5(60.7,80.7) | 70.3(59.5,79.7) |
| Hunan | 430529 | Zizhixian | 6.2 | 5.6 | 6.7 |  | 67.7(56.9,76.6) | 66.3(56.6,76.0) | 72.0(61.4,80.5) |
| Hunan | 430921 | Nan County | 5.2 | 4.2 | 6.1 |  | 66.6(55.9,77.0) | 70.3(59.3,79.7) | 62.9(50.0,74.5) |
| Hunan | 430527 | Suining County | 5.3 | 4.8 | 5.8 |  | 66.3(56.8,75.7) | 66.4(55.8,76.0) | 68.2(57.4,77.5) |
| Hunan | 433126 | Guzhang County | 5.2 | 4.8 | 5.5 |  | 65.5(50.5,79.8) | 64.0(46.4,81.7) | 67.9(52.6,83.3) |
| Hunan | 430412 | Nanyue District | 5.1 | 4.2 | 6.0 |  | 62.6(50.5,73.6) | 63.9(51.2,75.0) | 67.0(54.0,78.6) |
| Hunan | 430811 | Wulingyuan District | 5.4 | 4.4 | 6.3 |  | 61.9(50.7,72.5) | 62.5(50.5,73.8) | 66.1(53.0,77.2) |
| Hunan | 430525 | Dongkou County | 5.4 | 5.0 | 5.7 |  | 59.7(50.1,68.9) | 55.8(45.7,65.2) | 66.0(56.0,76.0) |
| Hunan | 430981 | Yuanjiang City | 4.6 | 3.7 | 5.5 |  | 59.3(48.9,69.9) | 63.6(51.2,74.6) | 54.2(43.9,65.2) |
| Hunan | 430502 | Shuangqing District | 5.2 | 4.4 | 6.0 |  | 55.9(39.8,72.6) | 53.5(37.9,69.9) | 61.0(42.6,79.5) |
| Tianjin | 120104 | Nankai District | 6.2 | 5.9 | 6.5 |  | 96.8(93.0,98.8) | 92.7(85.1,97.0) | 98.6(95.1,99.7) |
| Tianjin | 120106 | Hongqiao District | 8.0 | 7.6 | 8.3 |  | 95.7(90.6,98.4) | 96.1(89.1,99.1) | 92.5(83.8,97.5) |
| Tianjin | 120111 | Xiqing District | 3.9 | 3.6 | 4.1 |  | 95.7(90.3,98.5) | 90.9(81.7,96.3) | 98.1(93.2,99.6) |
| Tianjin | 120105 | Hebei District | 6.6 | 6.4 | 6.7 |  | 92.9(86.0,96.8) | 90.5(80.8,96.4) | 93.9(84.9,98.1) |
| Tianjin | 120101 | Heping District | 7.3 | 7.2 | 7.4 |  | 92.6(83.9,97.5) | 90.6(78.6,97.2) | 93.2(82.4,98.5) |
| Tianjin | 120110 | Dongli District | 4.1 | 4.0 | 4.2 |  | 90.5(83.7,95.4) | 89.9(80.1,95.9) | 91.1(82.5,96.0) |
| Tianjin | 120115 | Baodi District | 5.2 | 5.0 | 5.3 |  | 90.2(84.0,94.2) | 86.5(78.2,92.2) | 94.5(89.3,97.6) |
| Tianjin | 120102 | Hedong District | 6.7 | 6.3 | 7.0 |  | 89.5(78.8,95.9) | 90.1(78.1,96.5) | 86.3(75.2,94.3) |
| Tianjin | 120223 | Jinghai County | 5.2 | 4.5 | 5.9 |  | 86.5(80.2,90.7) | 84.9(77.4,89.8) | 88.5(82.1,92.8) |
| Tianjin | 120221 | Ninghe County | 5.0 | 5.1 | 4.9 |  | 86.2(78.4,92.0) | 78.1(66.8,86.3) | 93.6(86.9,97.4) |
| Tianjin | 120114 | Wuqing District | 5.3 | 5.0 | 5.6 |  | 85.2(78.9,89.8) | 80.4(72.4,87.0) | 89.8(83.4,94.7) |
| Tianjin | 120103 | Hexi District | 6.4 | 6.2 | 6.6 |  | 83.9(75.4,90.6) | 83.8(73.2,91.3) | 81.6(73.1,89.4) |
| Tianjin | 120112 | Jinnan District | 3.6 | 3.5 | 3.7 |  | 83.5(75.3,90.0) | 79.4(69.1,87.5) | 88.7(78.8,94.2) |
| Tianjin | 120113 | Beichen District | 4.5 | 4.2 | 4.7 |  | 80.2(71.2,87.3) | 72.7(62.3,81.9) | 87.8(78.2,94.6) |
| Tianjin | 120225 | Ji County | 7.0 | 6.9 | 7.2 |  | 80.1(72.8,85.3) | 74.7(67.2,81.0) | 86.0(79.6,90.4) |
| Tianjin | 120116 | Binhaixin District | 2.6 | 2.6 | 2.7 |  | 77.1(69.1,84.0) | 72.5(62.4,80.8) | 83.9(75.7,89.7) |
| Beijing | 110102 | Xicheng District | 6.7 | 6.1 | 7.3 |  | 96.0(91.5,98.3) | 96.1(91.0,98.6) | 94.8(88.2,98.3) |
| Beijing | 110109 | Mentougou District | 6.1 | 5.3 | 6.8 |  | 95.5(89.0,98.7) | 93.9(83.5,98.4) | 95.9(84.8,99.4) |
| Beijing | 110229 | Yanqing County | 6.4 | 4.8 | 8.1 |  | 94.8(87.9,98.1) | 96.5(90.1,99.2) | 90.4(81.0,96.6) |
| Beijing | 110101 | Dongcheng District | 6.6 | 6.0 | 7.3 |  | 94.3(88.4,97.9) | 96.1(90.4,98.9) | 90.6(81.4,96.1) |
| Beijing | 110228 | Miyun County | 6.5 | 5.4 | 7.6 |  | 92.7(86.7,96.4) | 90.6(83.2,95.6) | 94.1(86.0,98.1) |
| Beijing | 110107 | Shijingshan District | 5.2 | 4.6 | 5.7 |  | 91.6(84.1,96.3) | 91.2(81.8,96.7) | 91.4(80.9,97.2) |
| Beijing | 110106 | Fengtai District | 4.5 | 3.9 | 5.0 |  | 91.5(81.5,97.6) | 92.6(82.5,97.7) | 89.3(78.4,96.2) |
| Beijing | 110105 | Chaoyang District | 3.5 | 3.1 | 4.0 |  | 89.5(83.9,93.4) | 89.8(83.6,94.0) | 88.4(81.8,93.1) |
| Beijing | 110112 | Tongzhou District | 3.9 | 3.5 | 4.3 |  | 87.9(80.8,92.6) | 84.0(75.7,90.3) | 91.4(84.8,95.7) |
| Beijing | 110114 | Changping District | 2.7 | 2.4 | 2.9 |  | 85.4(68.7,95.7) | 78.6(58.1,91.3) | 92.8(78.1,98.3) |
| Beijing | 110108 | Haidian District | 3.1 | 2.8 | 3.4 |  | 81.9(73.7,88.8) | 83.1(75.2,90.0) | 81.2(71.8,88.3) |
| Beijing | 110113 | Shunyi District | 2.0 | 1.7 | 2.3 |  | 79.7(67.3,89.5) | 72.1(56.8,84.6) | 86.0(71.5,95.0) |
| Beijing | 110111 | Fangshan District | 2.5 | 2.0 | 2.9 |  | 73.1(60.8,83.8) | 69.5(56.7,81.5) | 77.6(63.0,89.2) |
| Beijing | 110115 | Daxing District | 1.5 | 1.3 | 1.7 |  | 72.9(61.3,82.5) | 68.3(56.2,79.7) | 80.2(67.7,89.2) |
| Beijing | 110116 | Huairou District | 2.3 | 2.0 | 2.6 |  | 63.7(47.7,78.0) | 63.0(45.5,81.6) | 66.8(50.2,84.2) |
| Beijing | 110117 | Pinggu District | 1.8 | 1.3 | 2.4 |  | 45.8(31.5,62.8) | 52.3(35.1,72.3) | 36.7(23.2,57.9) |
| Henan | 411421 | Minquan County | 8.7 | 7.6 | 9.9 |  | 97.6(95.3,98.8) | 97.6(95.2,99.0) | 97.1(94.4,98.6) |
| Henan | 411702 | Yicheng District | 5.2 | 4.1 | 6.2 |  | 96.4(83.7,99.8) | 96.2(83.7,99.6) | 95.1(80.3,99.6) |
| Henan | 410506 | Long'an District | 7.0 | 5.6 | 8.3 |  | 96.0(91.0,98.6) | 95.1(89.2,98.4) | 96.9(90.6,99.5) |
| Henan | 411422 | Sui County | 10.1 | 9.1 | 11.2 |  | 95.8(92.5,97.7) | 94.6(90.8,96.8) | 96.7(94.0,98.3) |
| Henan | 410503 | Beiguan District | 6.1 | 4.6 | 7.8 |  | 95.5(88.0,98.9) | 96.9(90.5,99.4) | 89.7(75.1,97.8) |
| Henan | 410725 | Yuanyang County | 6.7 | 5.7 | 7.6 |  | 95.3(91.1,97.8) | 94.1(89.6,97.3) | 96.1(91.4,98.5) |
| Henan | 410782 | Huixian City | 7.2 | 5.9 | 8.6 |  | 95.3(91.3,97.6) | 95.8(92.1,98.1) | 94.0(89.1,97.2) |
| Henan | 410202 | Longting District | 6.5 | 5.2 | 7.9 |  | 94.9(86.4,99.0) | 94.5(83.7,99.1) | 95.8(82.5,99.7) |
| Henan | 411721 | Xiping County | 8.0 | 7.1 | 8.9 |  | 94.9(90.5,97.3) | 93.9(88.7,97.1) | 94.7(89.6,97.8) |
| Henan | 411324 | Zhenping County | 6.9 | 5.9 | 7.9 |  | 94.7(90.2,97.4) | 93.5(88.5,97.1) | 95.7(91.4,98.1) |
| Henan | 410222 | Tongxu County | 7.2 | 6.1 | 8.2 |  | 94.3(90.1,97.1) | 94.3(89.7,97.2) | 94.0(89.2,97.1) |
| Henan | 410923 | Nanle County | 8.1 | 6.3 | 10.1 |  | 94.2(89.7,96.9) | 94.4(90.3,97.1) | 93.1(87.6,96.8) |
| Henan | 410203 | Shunhe Huizu District | 7.2 | 5.9 | 8.5 |  | 93.6(83.3,98.0) | 92.1(80.9,97.9) | 94.0(80.3,99.2) |
| Henan | 411325 | Neixiang County | 8.0 | 7.0 | 9.0 |  | 93.6(88.7,96.4) | 92.4(86.5,95.7) | 95.0(90.8,97.6) |
| Henan | 410423 | Lushan County | 6.9 | 5.9 | 7.8 |  | 92.9(87.3,96.3) | 91.0(84.8,95.6) | 94.9(90.4,97.6) |
| Henan | 411602 | Chuanhui District | 6.5 | 5.4 | 7.5 |  | 92.5(79.2,98.4) | 89.8(75.1,97.6) | 95.3(82.3,99.4) |
| Henan | 410402 | Xinhua District | 6.1 | 4.7 | 7.5 |  | 92.2(84.4,97.0) | 93.7(85.0,98.0) | 87.4(74.9,95.3) |
| Henan | 411627 | Taikang County | 9.2 | 7.6 | 10.7 |  | 92.2(80.8,97.4) | 92.7(81.7,97.7) | 92.0(80.7,97.1) |
| Henan | 411425 | Yucheng County | 8.1 | 7.0 | 9.1 |  | 92.1(87.0,95.3) | 91.0(85.8,94.5) | 93.8(89.8,96.7) |
| Henan | 411424 | Zhecheng County | 9.2 | 7.7 | 10.6 |  | 92.0(86.7,95.1) | 90.3(84.8,94.1) | 93.3(88.6,96.4) |
| Henan | 410327 | Yiyang County | 6.5 | 5.6 | 7.3 |  | 91.3(84.7,95.3) | 91.2(84.8,95.7) | 91.3(84.4,95.5) |
| Henan | 411623 | Shangshui County | 9.2 | 8.0 | 10.5 |  | 91.3(86.3,95.0) | 90.3(84.5,94.3) | 92.6(87.3,95.7) |
| Henan | 411624 | Shenqiu County | 9.9 | 8.5 | 11.3 |  | 91.3(85.7,95.1) | 89.0(82.4,93.3) | 93.3(88.5,96.3) |
| Henan | 410928 | Puyang County | 6.7 | 5.7 | 7.8 |  | 91.2(86.2,95.0) | 90.5(84.1,94.8) | 91.5(85.7,95.4) |
| Henan | 410225 | Lankao County | 6.8 | 5.7 | 8.0 |  | 91.0(84.6,95.2) | 89.0(81.6,94.4) | 92.7(86.5,96.8) |
| Henan | 410221 | Qi County | 6.7 | 5.9 | 7.4 |  | 90.8(84.7,94.9) | 90.2(83.3,94.9) | 91.7(85.8,95.5) |
| Henan | 411725 | Queshan County | 7.7 | 6.0 | 9.4 |  | 90.8(84.2,95.1) | 91.2(84.8,95.5) | 89.7(81.4,95.0) |
| Henan | 410211 | Jinming District | 4.6 | 3.9 | 5.3 |  | 90.5(79.4,97.4) | 87.6(74.5,96.4) | 92.5(78.9,98.4) |
| Henan | 411625 | Dancheng County | 8.9 | 7.7 | 10.1 |  | 90.5(84.7,94.6) | 89.0(82.1,93.7) | 92.2(86.4,95.7) |
| Henan | 410622 | Qi County | 6.4 | 5.7 | 7.1 |  | 90.4(83.7,95.1) | 86.0(77.7,91.8) | 95.2(88.8,98.3) |
| Henan | 410224 | Kaifeng County | 6.8 | 5.8 | 7.8 |  | 90.3(84.0,94.6) | 89.0(82.1,93.6) | 92.3(86.3,95.9) |
| Henan | 411303 | Wolong District | 5.9 | 4.7 | 7.0 |  | 90.0(83.7,94.5) | 89.1(81.9,93.8) | 90.7(83.7,95.1) |
| Henan | 410581 | Linzhou City | 8.8 | 7.4 | 10.3 |  | 89.9(84.2,93.7) | 86.6(80.0,91.5) | 93.3(88.6,96.2) |
| Henan | 411282 | Lingbao City | 6.0 | 5.2 | 6.7 |  | 89.9(82.9,95.2) | 90.0(81.8,95.2) | 89.1(79.8,94.7) |
| Henan | 410326 | Ruyang County | 7.5 | 6.7 | 8.2 |  | 89.6(83.1,94.0) | 83.5(75.0,90.4) | 94.6(89.5,97.6) |
| Henan | 411423 | Ningling County | 7.6 | 6.7 | 8.6 |  | 89.4(83.3,93.8) | 87.2(79.6,92.7) | 92.3(86.3,96.1) |
| Henan | 411728 | Suiping County | 7.3 | 6.9 | 7.7 |  | 89.4(81.7,94.0) | 84.0(74.5,90.8) | 93.7(87.9,97.4) |
| Henan | 410527 | Neihuang County | 6.9 | 5.4 | 8.6 |  | 89.3(81.8,94.0) | 89.6(82.7,94.5) | 88.8(81.5,94.2) |
| Henan | 410182 | Xingyang City | 6.0 | 5.2 | 6.8 |  | 89.1(82.1,93.8) | 88.3(80.7,93.8) | 89.6(82.1,94.3) |
| Henan | 410108 | Huiji District | 4.3 | 3.7 | 4.9 |  | 88.9(77.8,95.5) | 83.7(70.3,93.0) | 93.8(84.0,98.1) |
| Henan | 411281 | Yima City | 5.7 | 3.9 | 7.3 |  | 88.9(72.2,97.1) | 92.9(77.3,98.9) | 74.1(52.7,93.0) |
| Henan | 410522 | Anyang County | 6.5 | 5.7 | 7.3 |  | 88.3(81.0,93.6) | 86.4(78.2,92.6) | 91.5(84.3,96.1) |
| Henan | 411528 | Xi County | 8.1 | 7.6 | 8.7 |  | 88.2(82.0,92.7) | 85.7(78.4,90.6) | 91.1(85.9,94.9) |
| Henan | 411621 | Fugou County | 7.6 | 6.9 | 8.4 |  | 88.2(80.9,93.1) | 85.9(77.5,91.7) | 90.8(84.6,95.2) |
| Henan | 411082 | Changge City | 6.9 | 6.2 | 7.5 |  | 88.1(81.5,92.8) | 86.0(78.5,91.4) | 90.7(85.0,94.7) |
| Henan | 411302 | Yuancheng District | 5.7 | 4.6 | 6.8 |  | 87.9(79.9,93.3) | 87.1(79.3,92.9) | 89.2(81.3,94.7) |
| Henan | 411002 | Weidu District | 4.5 | 3.4 | 5.6 |  | 87.4(77.0,94.7) | 88.3(76.4,95.7) | 85.8(71.5,95.3) |
| Henan | 411322 | Fangcheng County | 8.3 | 7.1 | 9.5 |  | 87.4(80.7,91.8) | 83.9(76.8,89.7) | 90.0(84.6,93.8) |
| Henan | 410902 | Hualong District | 5.7 | 4.4 | 7.0 |  | 86.9(77.9,93.1) | 84.3(74.8,91.5) | 89.6(80.4,95.4) |
| Henan | 411402 | Liangyuan District | 6.4 | 5.6 | 7.3 |  | 86.9(79.0,92.5) | 83.1(74.7,89.9) | 91.9(84.3,96.0) |
| Henan | 411681 | Xiangcheng City | 8.5 | 7.2 | 9.8 |  | 86.9(72.7,95.0) | 86.4(72.1,94.9) | 87.1(73.2,94.7) |
| Henan | 411426 | Xiayi County | 9.1 | 8.1 | 10.0 |  | 86.8(80.2,91.2) | 83.0(75.4,88.7) | 90.9(85.9,94.5) |
| Henan | 410481 | Wugang City | 6.0 | 4.9 | 7.0 |  | 86.5(77.4,93.4) | 83.2(71.5,92.1) | 89.4(78.1,96.4) |
| Henan | 410302 | Laocheng District | 6.1 | 4.9 | 7.3 |  | 86.2(61.8,97.9) | 85.4(60.1,97.8) | 84.4(54.3,98.5) |
| Henan | 410204 | Gulou District | 7.7 | 6.2 | 9.3 |  | 86.0(74.5,94.0) | 87.4(76.0,96.3) | 81.3(65.0,94.1) |
| Henan | 411323 | Xixia County | 6.2 | 4.8 | 7.6 |  | 86.0(77.6,92.4) | 83.5(73.1,90.8) | 88.3(78.5,94.7) |
| Henan | 411081 | Yuzhou City | 7.5 | 6.3 | 8.6 |  | 85.8(78.7,91.1) | 84.2(77.1,90.1) | 87.1(80.9,92.0) |
| Henan | 410781 | Weihui City | 6.5 | 5.6 | 7.4 |  | 85.6(77.7,91.1) | 85.0(77.1,90.6) | 88.0(80.9,92.6) |
| Henan | 410122 | Zhongmu County | 5.6 | 4.9 | 6.3 |  | 85.5(76.7,91.9) | 81.7(71.5,89.6) | 89.7(81.8,95.3) |
| Henan | 410482 | Ruzhou City | 5.7 | 5.0 | 6.3 |  | 85.4(78.1,90.8) | 81.9(72.3,87.8) | 90.7(84.5,94.8) |
| Henan | 410502 | Wenfeng District | 4.6 | 3.6 | 5.6 |  | 84.7(74.7,93.0) | 84.5(73.3,92.1) | 87.2(75.6,95.2) |
| Henan | 410328 | Luoning County | 5.4 | 4.6 | 6.3 |  | 84.6(75.6,91.1) | 84.1(75.3,91.4) | 86.1(77.0,92.2) |
| Henan | 410721 | Xinxiang County | 5.9 | 5.6 | 6.2 |  | 84.5(74.4,92.1) | 77.1(66.4,86.6) | 92.4(84.5,97.2) |
| Henan | 411403 | Suiyang District | 6.7 | 5.7 | 7.7 |  | 84.5(75.6,90.7) | 84.3(74.8,90.4) | 86.1(77.7,92.1) |
| Henan | 410323 | Xin'an County | 6.8 | 5.8 | 7.8 |  | 84.2(75.5,90.5) | 82.8(73.9,89.3) | 86.0(77.3,92.2) |
| Henan | 411503 | Pingqiao District | 5.7 | 4.7 | 6.6 |  | 84.2(75.8,90.4) | 83.8(75.5,90.4) | 86.2(78.1,92.3) |
| Henan | 410223 | Weishi County | 6.1 | 5.5 | 6.7 |  | 84.1(76.1,90.2) | 81.1(72.0,87.6) | 88.9(82.9,93.6) |
| Henan | 411622 | Xihua County | 6.6 | 6.0 | 7.2 |  | 84.0(75.3,90.0) | 82.3(73.2,88.7) | 87.3(79.0,92.3) |
| Henan | 411626 | Huaiyang County | 7.9 | 6.5 | 9.3 |  | 84.0(76.4,89.8) | 83.1(74.8,89.0) | 86.4(79.2,91.2) |
| Henan | 410184 | Xinzheng City | 5.1 | 4.3 | 5.8 |  | 83.6(74.8,90.3) | 82.7(72.6,89.9) | 84.9(75.4,91.5) |
| Henan | 410329 | Yichuan County | 6.8 | 6.0 | 7.5 |  | 83.6(67.9,93.8) | 79.0(61.3,91.3) | 88.9(74.2,96.5) |
| Henan | 411327 | Sheqi County | 6.0 | 4.8 | 7.1 |  | 83.5(74.0,89.8) | 81.5(72.5,88.5) | 86.4(78.1,92.1) |
| Henan | 411221 | Mianchi County | 5.6 | 4.9 | 6.3 |  | 83.2(71.0,91.7) | 84.4(72.6,92.9) | 80.9(68.1,90.8) |
| Henan | 410381 | Yanshi City | 6.9 | 6.0 | 7.8 |  | 82.9(75.4,89.0) | 80.6(72.2,87.1) | 86.3(79.1,91.3) |
| Henan | 411202 | Hubin District | 5.4 | 4.5 | 6.3 |  | 82.3(70.1,91.6) | 85.9(71.0,94.8) | 75.2(62.4,87.0) |
| Henan | 410102 | Zhongyuan District | 4.2 | 3.5 | 4.9 |  | 81.9(58.3,96.4) | 81.3(55.2,96.2) | 82.2(56.0,96.8) |
| Henan | 410106 | Shangjie District | 5.2 | 4.2 | 6.1 |  | 81.9(62.9,94.8) | 86.7(64.5,97.2) | 71.6(53.1,90.4) |
| Henan | 410621 | Xun County | 5.8 | 5.2 | 6.5 |  | 81.3(71.7,88.0) | 74.8(64.3,83.3) | 89.6(81.5,94.4) |
| Henan | 411521 | Luoshan County | 7.8 | 6.7 | 8.8 |  | 81.1(72.1,87.3) | 80.6(72.2,86.8) | 81.3(72.6,87.7) |
| Henan | 410404 | Shilong District | 4.8 | 4.1 | 5.3 |  | 81.0(64.0,95.4) | 82.9(61.3,97.8) | 79.8(67.8,88.4) |
| Henan | 410306 | Jili District | 5.7 | 4.4 | 7.1 |  | 80.5(61.2,94.8) | 76.2(58.4,92.0) | 83.9(58.9,98.4) |
| Henan | 410325 | Song County | 6.7 | 5.6 | 7.8 |  | 79.9(71.2,86.6) | 78.7(69.1,85.0) | 83.1(76.3,88.9) |
| Henan | 411724 | Zhengyang County | 5.9 | 4.8 | 6.9 |  | 79.9(71.2,86.9) | 81.4(72.7,88.2) | 79.6(71.1,86.5) |
| Henan | 410104 | District | 3.7 | 3.2 | 4.2 |  | 79.4(58.4,93.8) | 77.1(54.2,93.8) | 80.3(60.5,96.0) |
| Henan | 411104 | Zhaoling District | 6.5 | 5.6 | 7.5 |  | 79.1(60.2,92.3) | 77.0(58.0,90.8) | 82.3(63.2,93.7) |
| Henan | 410103 | Erqi District | 4.0 | 3.4 | 4.6 |  | 79.0(55.9,94.4) | 79.0(54.8,95.1) | 75.6(54.9,93.5) |
| Henan | 410205 | Liwangtai District | 7.2 | 6.2 | 8.2 |  | 78.9(69.3,86.6) | 80.8(71.9,88.6) | 79.4(70.1,87.6) |
| Henan | 410505 | Yindu District | 5.6 | 4.6 | 6.7 |  | 78.6(68.3,85.6) | 79.0(69.8,85.9) | 80.9(72.5,88.9) |
| Henan | 410322 | Mengjin County | 6.9 | 6.0 | 7.7 |  | 78.4(58.5,92.2) | 76.1(58.2,90.0) | 81.7(62.4,94.1) |
| Henan | 410523 | Tangyin County | 6.4 | 5.2 | 7.7 |  | 77.6(68.6,84.7) | 78.2(68.7,86.0) | 79.2(69.4,86.3) |
| Henan | 411024 | Yanling County | 5.3 | 4.6 | 5.9 |  | 77.5(68.0,84.8) | 80.1(70.7,87.3) | 75.2(65.6,82.7) |
| Henan | 410105 | Jinshui District | 3.3 | 2.7 | 3.8 |  | 76.1(63.4,86.7) | 75.8(61.6,87.8) | 78.8(66.6,89.0) |
| Henan | 411522 | Guangshan County | 6.4 | 5.2 | 7.6 |  | 76.1(65.6,84.6) | 75.0(65.1,82.8) | 78.8(68.6,86.7) |
| Henan | 411628 | Luyi County | 6.5 | 5.6 | 7.5 |  | 76.1(64.9,84.9) | 75.0(64.3,83.2) | 79.7(69.7,87.3) |
| Henan | 410926 | Fan County | 7.9 | 6.1 | 9.6 |  | 76.0(57.0,90.3) | 79.7(61.7,92.0) | 71.4(52.8,88.6) |
| Henan | 410311 | Luolong District | 4.3 | 3.7 | 5.0 |  | 75.7(55.3,92.0) | 76.2(53.2,93.0) | 77.0(55.9,91.4) |
| Henan | 410324 | Luanchuan County | 5.6 | 4.7 | 6.4 |  | 75.7(65.8,83.8) | 71.4(60.8,80.3) | 82.8(73.3,90.5) |
| Henan | 410303 | Xigong District | 4.9 | 4.0 | 5.8 |  | 75.6(53.2,93.0) | 81.1(58.9,96.4) | 66.1(45.0,86.2) |
| Henan | 410425 | Jia County | 6.8 | 6.2 | 7.3 |  | 75.6(66.5,83.3) | 73.0(62.4,81.9) | 79.3(69.8,86.5) |
| Henan | 411502 | Shihe District | 5.7 | 4.6 | 6.8 |  | 75.6(66.2,83.6) | 73.0(63.4,82.0) | 80.7(72.4,88.0) |
| Henan | 410421 | Baofeng County | 5.6 | 5.0 | 6.1 |  | 75.1(63.9,83.8) | 71.0(59.5,80.8) | 81.4(70.7,89.5) |
| Henan | 411023 | Xuchang County | 5.9 | 5.0 | 6.8 |  | 74.6(65.0,82.5) | 75.7(65.1,83.5) | 74.0(64.0,82.3) |
| Henan | 410526 | Hua County | 5.6 | 4.8 | 6.4 |  | 74.0(64.0,82.4) | 73.3(63.1,82.3) | 76.3(66.0,83.8) |
| Henan | 410883 | Mengzhou City | 6.4 | 5.7 | 7.1 |  | 73.3(62.3,82.6) | 67.0(54.5,77.7) | 81.7(70.6,89.5) |
| Henan | 410304 | Chanhe Huizu District | 5.8 | 5.0 | 6.6 |  | 73.2(49.6,91.5) | 71.5(49.9,89.7) | 75.9(51.7,94.7) |
| Henan | 411321 | Nanzhao County | 5.4 | 4.2 | 6.5 |  | 73.0(62.5,82.1) | 71.2(59.7,80.8) | 76.3(66.0,85.2) |
| Henan | 411727 | Runan County | 5.1 | 4.5 | 5.7 |  | 72.7(54.3,87.4) | 69.9(48.3,85.2) | 79.3(59.7,93.0) |
| Henan | 419001 | Jiyuan City(XianJi) | 5.4 | 4.8 | 6.0 |  | 72.5(62.3,81.7) | 70.8(60.5,80.1) | 76.5(67.0,84.2) |
| Henan | 410726 | Yanjin County | 5.0 | 4.4 | 5.5 |  | 72.3(61.6,81.6) | 69.7(56.8,80.1) | 79.6(68.6,88.3) |
| Henan | 411328 | Tanghe County | 6.7 | 5.3 | 8.0 |  | 72.3(61.9,79.9) | 70.0(60.1,78.7) | 73.4(63.5,81.2) |
| Henan | 410185 | Dengfeng City | 4.7 | 4.3 | 5.0 |  | 72.0(61.9,81.2) | 69.3(57.8,78.6) | 77.7(68.8,85.4) |
| Henan | 411025 | Xiangcheng County | 5.8 | 4.9 | 6.6 |  | 72.0(62.7,80.4) | 73.3(63.7,81.8) | 72.4(62.2,80.3) |
| Henan | 411121 | Wuyang County | 6.8 | 6.2 | 7.4 |  | 71.9(62.1,80.7) | 69.3(57.6,79.0) | 74.1(62.7,82.9) |
| Henan | 410724 | Huojia County | 5.7 | 4.9 | 6.4 |  | 71.8(60.6,81.4) | 72.8(60.8,83.4) | 72.2(61.2,81.8) |
| Henan | 411224 | Lushi County | 6.2 | 5.3 | 7.1 |  | 71.7(61.7,81.0) | 69.8(58.5,79.9) | 74.3(63.7,82.9) |
| Henan | 410422 | Ye County | 5.6 | 4.7 | 6.3 |  | 71.6(59.9,82.1) | 71.1(58.4,81.7) | 72.6(60.3,83.3) |
| Henan | 411523 | Xin County | 5.7 | 4.6 | 6.7 |  | 71.2(60.5,80.7) | 74.7(62.7,84.6) | 66.4(56.4,77.2) |
| Henan | 410922 | Qingfeng County | 6.3 | 5.4 | 7.1 |  | 70.2(60.4,78.7) | 69.9(59.6,78.4) | 73.0(63.3,81.5) |
| Henan | 411726 | Biyang County | 5.2 | 4.3 | 6.1 |  | 70.2(58.8,79.1) | 67.6(57.3,77.0) | 74.9(63.9,84.1) |
| Henan | 411526 | Huangchuan County | 5.9 | 5.2 | 6.7 |  | 69.9(59.0,78.8) | 70.4(59.5,79.5) | 72.2(61.4,81.3) |
| Henan | 410603 | Shancheng District | 4.1 | 3.2 | 4.9 |  | 69.6(55.2,83.0) | 76.6(60.9,90.0) | 57.9(46.8,68.8) |
| Henan | 410411 | Zhenhe District | 4.9 | 3.8 | 6.0 |  | 69.4(47.0,88.4) | 66.2(44.0,86.0) | 73.2(48.6,94.4) |
| Henan | 410183 | Xinmi City | 4.9 | 4.3 | 5.5 |  | 68.6(57.6,77.8) | 66.2(55.1,75.7) | 73.5(63.4,82.3) |
| Henan | 411330 | Tongbai County | 6.2 | 4.8 | 7.6 |  | 68.2(57.7,77.2) | 68.2(57.0,77.7) | 68.6(56.6,78.5) |
| Henan | 411102 | Yuanhui District | 6.0 | 5.0 | 7.0 |  | 66.9(45.0,84.0) | 66.8(48.4,84.0) | 68.5(48.0,86.9) |
| Henan | 410927 | Taiqian County | 6.8 | 5.9 | 7.7 |  | 64.3(52.3,74.5) | 66.7(55.6,76.6) | 64.5(54.5,74.6) |
| Henan | 411329 | Xinye County | 6.8 | 5.7 | 7.8 |  | 64.1(53.7,73.9) | 62.7(52.5,73.7) | 66.7(56.6,75.4) |
| Henan | 410703 | Weibin District | 3.8 | 2.6 | 5.0 |  | 63.2(37.1,89.1) | 73.9(46.4,95.4) | 46.1(25.7,77.4) |
| Henan | 411481 | Yongcheng City | 5.2 | 4.6 | 5.7 |  | 61.5(51.7,72.2) | 61.9(51.0,71.5) | 63.2(52.1,72.8) |
| Henan | 410305 | Jianxi District | 4.7 | 3.8 | 5.6 |  | 61.2(38.3,85.9) | 61.3(39.2,82.1) | 63.8(39.9,85.9) |
| Henan | 411103 | Yancheng District | 5.7 | 4.5 | 6.8 |  | 60.7(49.7,71.0) | 62.0(51.0,72.2) | 61.5(50.1,71.2) |
| Henan | 411222 | Shan County | 4.8 | 4.0 | 5.5 |  | 60.6(48.7,72.5) | 61.8(48.6,74.7) | 59.6(46.7,72.5) |
| Henan | 411722 | Shangcai County | 4.9 | 4.3 | 5.4 |  | 60.5(40.4,81.1) | 59.4(39.3,79.2) | 66.0(45.3,85.5) |
| Henan | 410728 | Changyuan County | 4.3 | 3.6 | 5.0 |  | 60.1(47.3,72.2) | 61.5(49.4,73.0) | 60.9(48.1,73.2) |
| Henan | 410825 | Wen County | 5.4 | 4.8 | 5.9 |  | 59.9(48.7,69.7) | 56.5(45.1,66.8) | 66.7(55.4,76.8) |
| Henan | 411524 | Shangcheng County | 6.2 | 4.8 | 7.6 |  | 59.0(47.0,69.6) | 62.2(50.6,74.2) | 54.1(42.7,66.5) |
| Henan | 411381 | Dengzhou City | 5.6 | 4.6 | 6.6 |  | 58.9(49.5,68.8) | 59.2(48.6,68.8) | 60.5(50.1,70.1) |
| Henan | 410802 | Jiefang District | 5.9 | 4.7 | 7.0 |  | 58.5(45.5,70.5) | 60.8(47.4,73.1) | 55.4(42.4,70.3) |
| Henan | 410611 | Qibin District | 4.8 | 3.8 | 5.8 |  | 58.1(45.9,69.7) | 56.5(45.2,67.6) | 63.4(50.0,76.5) |
| Henan | 410882 | Qinyang City | 5.2 | 4.6 | 5.9 |  | 57.0(46.7,67.0) | 56.7(45.7,68.1) | 61.7(51.2,71.2) |
| Henan | 410823 | Wuzhi County | 5.2 | 4.5 | 5.9 |  | 56.7(46.2,67.0) | 54.6(43.7,64.6) | 62.3(51.8,72.2) |
| Henan | 410181 | Gongyi City | 4.5 | 3.8 | 5.1 |  | 56.3(45.7,65.5) | 57.0(46.3,67.7) | 57.9(48.1,67.4) |
| Henan | 411527 | Huaibin County | 4.8 | 4.0 | 5.6 |  | 56.3(45.1,68.3) | 59.4(47.0,70.2) | 55.3(43.9,67.1) |
| Henan | 411122 | Linying County | 5.9 | 5.3 | 6.4 |  | 55.7(45.4,65.8) | 51.1(40.3,61.2) | 64.0(52.6,73.2) |
| Henan | 410702 | Hongqi District | 2.8 | 1.9 | 3.6 |  | 55.0(31.4,82.1) | 56.7(31.6,83.2) | 55.7(30.5,87.3) |
| Henan | 410403 | Weidong District | 4.6 | 3.3 | 5.9 |  | 53.5(41.2,66.1) | 60.3(47.0,73.0) | 47.6(36.0,60.3) |
| Henan | 410602 | Heshan District | 3.9 | 3.0 | 4.8 |  | 53.2(37.9,70.5) | 53.4(40.1,69.1) | 54.5(34.4,79.3) |
| Henan | 410727 | Fengqiu County | 3.4 | 3.0 | 3.8 |  | 53.2(40.2,66.9) | 50.2(38.1,63.3) | 60.9(48.3,73.5) |
| Henan | 410711 | Muye District | 3.9 | 3.0 | 4.8 |  | 51.3(37.5,67.1) | 59.0(43.5,75.6) | 43.7(31.4,59.3) |
| Henan | 410822 | Bo'ai County | 4.9 | 4.5 | 5.3 |  | 50.3(39.2,61.3) | 47.1(36.0,58.6) | 58.9(47.0,70.2) |
| Henan | 411326 | Xichuan County | 3.9 | 3.0 | 4.8 |  | 49.8(38.9,60.7) | 51.4(41.0,62.7) | 50.9(39.4,62.2) |
| Henan | 410811 | Shanyang District | 4.8 | 3.8 | 5.7 |  | 45.2(27.5,70.2) | 42.6(25.0,64.6) | 50.7(28.4,79.6) |
| Henan | 411723 | Pingyu County | 3.5 | 3.1 | 4.0 |  | 43.4(32.4,54.6) | 41.0(31.0,52.1) | 50.7(36.9,64.7) |
| Henan | 410821 | Xiuwu County | 4.7 | 4.3 | 5.0 |  | 41.8(25.1,61.2) | 40.6(23.7,63.5) | 47.2(28.4,64.8) |
| Henan | 410803 | Zhongzhan District | 4.8 | 4.2 | 5.5 |  | 38.7(28.0,51.2) | 39.8(28.3,52.6) | 40.0(27.3,57.4) |
| Henan | 411729 | Xincai County | 2.9 | 2.5 | 3.3 |  | 38.7(27.8,50.1) | 37.2(27.7,48.7) | 43.8(31.1,59.6) |
| Henan | 410804 | Macun District | 4.2 | 3.4 | 4.9 |  | 38.2(27.2,51.3) | 37.8(26.1,51.3) | 40.1(25.6,58.2) |
| Henan | 410704 | Fengquan District | 1.6 | 1.1 | 2.1 |  | 25.8(16.7,40.5) | 20.6(14.0,28.3) | 39.7(20.8,71.1) |
| Henan | 411525 | Gushi County | 3.7 | 3.0 | 4.4 |  | 22.3(16.1,30.1) | 23.8(17.2,32.3) | 22.0(15.6,30.2) |
| Chongqing | 500103 | Yuzhong District | 5.8 | 5.1 | 6.5 |  | 98.9(96.6,99.8) | 94.1(86.4,98.1) | 99.9(98.8,100.0) |
| Chongqing | 500104 | Dadukou District | 5.5 | 4.7 | 6.4 |  | 98.8(94.6,99.9) | 99.4(96.3,100.0) | 94.5(83.6,99.2) |
| Chongqing | 500105 | Jiangbei District | 5.1 | 4.3 | 5.9 |  | 96.2(90.8,98.8) | 94.5(87.6,98.2) | 97.2(91.3,99.5) |
| Chongqing | 500108 | Nan'an District | 4.9 | 4.2 | 5.6 |  | 95.2(89.2,98.2) | 94.8(88.0,98.1) | 95.0(88.5,98.4) |
| Chongqing | 500112 | Yubei District | 5.4 | 4.3 | 6.5 |  | 94.3(89.8,97.3) | 93.9(88.7,97.2) | 93.7(88.1,97.2) |
| Chongqing | 500115 | Changshou District | 7.5 | 6.2 | 8.7 |  | 92.4(87.2,96.3) | 91.4(84.8,95.9) | 92.1(84.2,96.7) |
| Chongqing | 500113 | Banan District | 6.8 | 5.3 | 8.1 |  | 91.5(85.3,95.8) | 92.8(86.2,96.8) | 86.8(76.5,93.8) |
| Chongqing | 500111 | Shuangqiao District | 8.4 | 6.6 | 10.1 |  | 91.4(86.6,95.0) | 91.6(86.0,95.0) | 90.2(83.9,94.5) |
| Chongqing | 500106 | Shapingba District | 5.2 | 4.4 | 6.0 |  | 91.3(84.4,95.7) | 90.1(81.9,95.1) | 92.4(84.9,96.8) |
| Chongqing | 500238 | Wuxi County | 12.4 | 10.1 | 14.7 |  | 91.3(86.5,94.7) | 86.8(79.9,92.0) | 93.0(88.1,95.9) |
| Chongqing | 500107 | Jiulongpo District | 4.7 | 3.9 | 5.5 |  | 91.1(84.3,95.6) | 90.1(82.4,95.8) | 91.2(82.9,96.1) |
| Chongqing | 500109 | Beibei District | 6.6 | 5.4 | 7.9 |  | 88.4(81.5,93.9) | 88.1(79.3,94.2) | 88.2(78.8,94.7) |
| Chongqing | 500119 | Nanchuan District | 7.6 | 6.2 | 8.9 |  | 86.8(79.0,92.0) | 85.6(77.9,91.6) | 87.5(79.4,93.2) |
| Chongqing | 500237 | Wushan County | 8.5 | 7.0 | 9.8 |  | 86.8(79.9,91.5) | 82.4(74.6,88.7) | 91.0(84.3,95.1) |
| Chongqing | 500118 | Yongchuan District | 7.4 | 5.9 | 8.9 |  | 84.3(77.6,89.4) | 83.9(76.2,89.0) | 84.8(76.7,90.3) |
| Chongqing | 500243 | Tujiazu Zizhixian | 8.5 | 7.2 | 9.8 |  | 84.1(76.9,90.2) | 80.3(71.6,87.0) | 86.7(78.7,92.7) |
| Chongqing | 500101 | Wanzhou District | 7.7 | 6.4 | 8.9 |  | 83.9(76.6,89.2) | 81.6(73.0,87.9) | 85.2(77.3,90.7) |
| Chongqing | 500114 | Qianjiang District | 7.1 | 5.9 | 8.2 |  | 83.8(74.9,89.8) | 79.7(71.3,87.6) | 88.1(79.1,93.9) |
| Chongqing | 500230 | Fengdu County | 8.9 | 7.3 | 10.5 |  | 83.6(75.9,89.5) | 84.2(75.8,90.0) | 80.9(71.8,87.6) |
| Chongqing | 500223 | Tongnan County | 7.0 | 5.7 | 8.3 |  | 81.8(73.2,88.8) | 85.1(77.2,91.5) | 72.4(62.2,81.5) |
| Chongqing | 500228 | Liangping County | 8.0 | 6.6 | 9.3 |  | 79.8(71.4,86.4) | 79.8(71.2,86.4) | 78.0(69.4,85.9) |
| Chongqing | 500116 | Jiangjin District | 8.2 | 6.4 | 9.9 |  | 78.6(70.7,84.7) | 77.7(68.9,85.0) | 78.0(69.1,85.2) |
| Chongqing | 500231 | Dianjiang County | 7.7 | 6.5 | 9.0 |  | 78.0(69.0,85.1) | 74.8(64.9,82.6) | 80.4(70.0,88.2) |
| Chongqing | 500236 | Fengjie County | 8.0 | 6.7 | 9.2 |  | 78.0(69.7,85.3) | 74.5(65.7,82.3) | 81.3(73.0,87.2) |
| Chongqing | 500234 | Kai County | 8.3 | 6.7 | 9.9 |  | 76.8(68.3,83.8) | 74.3(65.1,81.6) | 79.0(70.8,85.6) |
| Chongqing | 500117 | Hechuan District | 7.1 | 5.6 | 8.5 |  | 76.7(67.7,83.8) | 78.1(68.2,85.5) | 73.5(64.0,82.0) |
| Chongqing | 500226 | Rongchang County | 7.8 | 6.1 | 9.4 |  | 76.2(66.7,84.0) | 79.9(71.2,86.4) | 69.5(59.3,79.0) |
| Chongqing | 500102 | Fuling District | 7.3 | 6.1 | 8.6 |  | 74.5(65.3,82.1) | 73.9(64.6,81.8) | 75.6(66.2,83.5) |
| Chongqing | 500110 | Wansheng District | 7.8 | 6.2 | 9.4 |  | 74.1(64.8,82.0) | 74.1(64.5,81.8) | 72.3(60.9,81.1) |
| Chongqing | 500233 | Zhong County | 8.5 | 7.2 | 9.8 |  | 73.8(64.8,80.9) | 71.7(62.5,80.0) | 75.0(64.6,83.2) |
| Chongqing | 500241 | Zizhixian | 7.6 | 6.0 | 9.0 |  | 73.2(58.6,83.9) | 71.5(58.1,83.7) | 75.9(61.9,86.9) |
| Chongqing | 500240 | Zizhixian | 7.7 | 6.9 | 8.4 |  | 72.4(55.6,86.5) | 71.6(54.1,86.4) | 73.6(58.8,86.3) |
| Chongqing | 500242 | Miaozu Zizhixian | 7.4 | 6.2 | 8.6 |  | 71.2(61.0,79.1) | 69.2(58.6,78.3) | 72.8(62.3,81.8) |
| Chongqing | 500224 | Tongliang County | 7.2 | 5.5 | 8.9 |  | 70.8(60.8,79.8) | 71.5(61.5,80.7) | 68.0(56.4,78.9) |
| Chongqing | 500232 | Wulong County | 7.9 | 6.7 | 9.2 |  | 70.3(59.2,79.3) | 69.6(57.7,79.6) | 69.3(57.7,79.1) |
| Chongqing | 500227 | Bishan County | 6.4 | 4.8 | 7.9 |  | 69.3(59.2,78.5) | 70.6(60.4,79.7) | 66.4(54.3,76.9) |
| Chongqing | 500229 | Chengkou County | 7.9 | 6.5 | 9.2 |  | 66.3(51.1,79.8) | 66.0(49.1,81.4) | 66.8(51.5,81.1) |
| Chongqing | 500235 | Yunyang County | 6.9 | 5.4 | 8.4 |  | 64.5(54.4,74.7) | 65.3(54.9,74.9) | 63.4(52.1,73.4) |
| Jilin | 220102 | Nanguan District | 6.6 | 5.6 | 7.8 |  | 99.8(98.8,100.0) | 99.9(98.9,100.0) | 98.4(92.0,99.9) |
| Jilin | 220211 | Fengman District | 6.1 | 5.1 | 7.1 |  | 99.8(97.6,100.0) | 99.3(92.7,100.0) | 99.8(94.9,100.0) |
| Jilin | 220103 | Kuancheng District | 6.8 | 5.6 | 8.0 |  | 99.3(96.7,99.9) | 99.5(96.5,100.0) | 98.3(91.2,99.8) |
| Jilin | 222401 | Yanji City | 7.8 | 6.5 | 9.0 |  | 99.1(96.8,99.8) | 98.6(95.3,99.8) | 99.0(95.2,99.9) |
| Jilin | 220105 | Erdao District | 6.6 | 5.3 | 7.9 |  | 99.0(95.4,99.9) | 99.3(94.6,100.0) | 97.6(88.9,99.8) |
| Jilin | 220302 | Tiexi District | 6.6 | 5.5 | 7.6 |  | 98.5(92.0,99.9) | 96.7(82.2,99.7) | 99.2(90.4,100) |
| Jilin | 220183 | Dehui City | 7.1 | 5.9 | 8.2 |  | 97.6(94.9,99.1) | 96.5(92.4,98.7) | 98.1(94.3,99.6) |
| Jilin | 220104 | Chaoyang District | 5.2 | 4.5 | 5.9 |  | 96.1(81.4,99.8) | 95.0(78.1,99.8) | 95.7(77.0,99.8) |
| Jilin | 220402 | Longshan District | 7.4 | 6.1 | 8.7 |  | 95.6(87.1,99.0) | 95.7(85.0,99.4) | 94.2(81.3,99.4) |
| Jilin | 220502 | Dongchang District | 6.2 | 5.1 | 7.3 |  | 95.0(83.7,99.2) | 93.6(78.5,98.9) | 94.7(74.9,99.7) |
| Jilin | 220106 | Luyuan District | 4.8 | 4.0 | 5.5 |  | 94.2(84.2,98.7) | 95.0(82.5,99.1) | 91.5(75.1,98.6) |
| Jilin | 222404 | Huichun City | 7.2 | 6.2 | 8.2 |  | 93.4(84.8,98.2) | 94.0(82.5,98.9) | 90.7(76.9,98.3) |
| Jilin | 220721 | Zizhixian | 6.3 | 5.1 | 7.4 |  | 92.6(86.5,96.2) | 94.5(88.8,97.9) | 87.3(79.0,93.6) |
| Jilin | 220303 | Tiedong District | 6.7 | 5.7 | 7.7 |  | 92.3(80.7,97.8) | 95.7(84.8,99.4) | 79.1(70.2,85.8) |
| Jilin | 220503 | Erdaojiang District | 7.5 | 6.2 | 8.8 |  | 92.3(76.1,99.2) | 96.3(78.2,99.9) | 78.0(67.7,85.9) |
| Jilin | 222403 | Dunhua City | 7.5 | 6.1 | 8.8 |  | 92.3(83.8,96.7) | 88.2(78.6,94.7) | 94.3(84.0,98.8) |
| Jilin | 220421 | Dongfeng County | 7.3 | 5.8 | 8.7 |  | 90.2(83.0,95.2) | 89.9(80.7,95.4) | 90.0(79.8,96.3) |
| Jilin | 220521 | Tonghua County | 6.1 | 4.9 | 7.1 |  | 89.9(78.7,96.5) | 88.5(74.3,96.5) | 90.5(73.8,98.5) |
| Jilin | 220112 | Shuangyang District | 6.3 | 5.2 | 7.4 |  | 89.4(78.8,95.3) | 85.7(75.4,93.8) | 92.9(80.7,98.2) |
| Jilin | 220403 | Xi'an District | 8.8 | 7.2 | 10.4 |  | 88.2(76.9,95.8) | 85.7(78.9,90.2) | 90.0(71.1,99.1) |
| Jilin | 220581 | Meihekou City | 6.3 | 5.1 | 7.4 |  | 88.1(80.4,93.4) | 89.9(81.2,95.4) | 84.7(73.8,92.4) |
| Jilin | 220602 | Badaojiang District | 6.3 | 5.0 | 7.5 |  | 87.5(74.7,95.6) | 92.5(80.3,98.3) | 74.2(60.4,89.3) |
| Jilin | 220881 | Taonan City | 6.7 | 5.5 | 7.8 |  | 87.0(78.3,93.5) | 88.7(77.9,95.6) | 81.9(69.7,91.6) |
| Jilin | 220202 | Changyi District | 4.6 | 3.6 | 5.6 |  | 85.4(71.9,94.3) | 86.8(70.5,96.2) | 80.7(56.3,94.6) |
| Jilin | 220422 | Dongliao County | 5.7 | 4.7 | 6.7 |  | 85.2(74.2,93.2) | 84.0(70.5,93.4) | 87.3(74.8,96.3) |
| Jilin | 220802 | Taobei District | 5.1 | 4.1 | 6.0 |  | 84.2(71.2,93) | 90.2(77,97.1) | 69.7(57.0,84.0) |
| Jilin | 220621 | Fusong County | 6.6 | 5.8 | 7.5 |  | 83.2(65.7,93.3) | 86.4(69.1,96.9) | 70.6(51.7,89.8) |
| Jilin | 220623 | Zizhixian | 6.2 | 5.2 | 7.1 |  | 83.1(55.4,97.5) | 83.1(52.1,98.2) | 74.2(44.2,96.8) |
| Jilin | 220284 | Panshi City | 4.9 | 4.0 | 5.8 |  | 83(71,91.5) | 80.8(68.4,91.4) | 84.2(67.9,94.7) |
| Jilin | 222426 | Antu County | 5.8 | 4.5 | 6.9 |  | 78.3(62.6,90.7) | 73.9(60.6,86.4) | 82.8(56.9,97.2) |
| Jilin | 222406 | Helong City | 8.0 | 6.6 | 9.2 |  | 76.6(63.2,88.4) | 84.7(70.2,95) | 57.5(46.2,69.3) |
| Jilin | 220283 | Shulan City | 6.2 | 5.1 | 7.2 |  | 76.5(67,85.1) | 79.5(69.6,87.3) | 71.1(59.4,80.9) |
| Jilin | 222424 | Wangqing County | 6.8 | 6.0 | 7.7 |  | 75.7(62.5,87.6) | 78.8(64,91.5) | 69.6(54.7,86) |
| Jilin | 220702 | Ningjiang District | 3.5 | 2.8 | 4.2 |  | 74.9(61.6,87.3) | 81(65.8,92) | 68(55.3,81.1) |
| Jilin | 220622 | Jingyu County | 6.0 | 4.7 | 7.3 |  | 74.7(58.9,88) | 81.4(64.7,94.9) | 63.3(50.5,74) |
| Jilin | 220524 | Liuhe County | 5.5 | 4.4 | 6.6 |  | 73.3(62.2,82.4) | 76.3(65.5,85) | 72.1(60.7,82.3) |
| Jilin | 220322 | Lishu County | 4.7 | 3.9 | 5.4 |  | 72.1(61.9,82.1) | 69.4(57.9,78.9) | 77.1(64.9,86.3) |
| Jilin | 220605 | Jiangyuan County | 6.8 | 5.7 | 7.8 |  | 71.2(54.3,87.3) | 65.6(56.4,74.7) | 76.3(49.0,95.9) |
| Jilin | 220182 | Yushu City | 4.9 | 4.1 | 5.7 |  | 71.0(61.8,79.1) | 69.5(59.3,77.3) | 76.9(66.7,84.5) |
| Jilin | 220382 | Shuangliao City | 4.8 | 3.9 | 5.7 |  | 70.1(59.1,80.9) | 76.7(61.9,88.2) | 62.5(53.1,71.6) |
| Jilin | 220281 | Jiaohe City | 5.7 | 4.6 | 6.7 |  | 68.9(57.5,79.5) | 76.5(62.7,87.2) | 58.8(48.6,68.4) |
| Jilin | 220204 | Chuanying District | 3.7 | 2.9 | 4.5 |  | 68.7(49.7,87.5) | 76.4(50.8,94.1) | 56.2(37.2,80.8) |
| Jilin | 220221 | Yongji County | 5.7 | 4.7 | 6.7 |  | 68.7(59.0,76.4) | 69.5(60.3,77.6) | 71.1(61.7,78.9) |
| Jilin | 220181 | Jiutai City | 3.6 | 2.9 | 4.3 |  | 67.6(55.2,79.0) | 69.0(55.9,81.2) | 66.5(52.2,80.6) |
| Jilin | 220282 | Huadian City | 4.8 | 3.9 | 5.7 |  | 66.3(54.3,77.1) | 66.0(55.4,75.7) | 70.0(54.6,84.8) |
| Jilin | 222402 | Tumen City | 7.6 | 6.1 | 9.0 |  | 64.2(54.1,74.4) | 71.7(62.5,79.9) | 53.3(41.4,65.0) |
| Jilin | 220203 | Longtan District | 4.8 | 3.9 | 5.6 |  | 63.9(51.1,76.6) | 59.1(49.9,68.4) | 71.4(50.5,90.3) |
| Jilin | 220582 | Ji'an City | 6.2 | 5.2 | 7.1 |  | 63.2(50.6,75.7) | 64.1(51.2,77.5) | 62.0(47.4,80.6) |
| Jilin | 220681 | Linjiang City | 5.6 | 4.5 | 6.6 |  | 62.1(45.8,81.5) | 64.0(48.2,82.3) | 55.2(33.9,84.4) |
| Jilin | 220523 | Huinan County | 4.6 | 3.7 | 5.5 |  | 58.9(48.2,70.1) | 62.3(50.0,74.6) | 58.3(48.1,68.6) |
| Jilin | 220724 | Fuyu County | 3.6 | 3.0 | 4.2 |  | 58.2(47.3,69.0) | 61.6(49.8,72.9) | 57.5(47.5,67.5) |
| Jilin | 222405 | Longjing City | 7.2 | 6.3 | 8.1 |  | 58.2(46.5,69.2) | 64.4(53.0,74.3) | 48.6(36.8,60.9) |
| Jilin | 220323 | Yitong Manzu Zizhixian | 4.4 | 3.7 | 5.0 |  | 57.2(46.4,68.2) | 55.1(43.0,66.4) | 63.6(50.9,76.9) |
| Jilin | 220821 | Zhenlai County | 4.6 | 3.7 | 5.4 |  | 57.1(45,70.5) | 58.5(46.2,72.4) | 58.3(44.8,74.0) |
| Jilin | 220822 | Tongyu County | 3.4 | 2.7 | 4.1 |  | 55.2(41.6,69.6) | 59.2(43.5,76.4) | 54.7(42.3,69.8) |
| Jilin | 220122 | Nong'an County | 3.5 | 2.9 | 3.9 |  | 50.2(40.7,60.6) | 47.4(39.0,57.2) | 58.5(48.1,69.2) |
| Jilin | 220722 | Changling County | 2.0 | 1.5 | 2.6 |  | 41.3(31.5,52.6) | 41.5(32.0,51.9) | 47.4(35.2,61.7) |
| Jilin | 220381 | Gongzhuling City | 2.5 | 2.0 | 3.1 |  | 40.0(29.8,50.9) | 40.7(30.8,53.1) | 41.4(31.6,54.1) |
| Jilin | 220723 | Qian'an County | 2.8 | 2.1 | 3.4 |  | 39.1(30.1,51.0) | 40.1(29.1,54.5) | 44.2(32.3,60.7) |
| Jilin | 220882 | Da'an City | 2.8 | 2.4 | 3.2 |  | 33.9(23.5,46.1) | 32.8(23.4,45.2) | 38.0(27.4,56.1) |
| Hubei | 420505 | Xiaoting District | 10.2 | 8.4 | 11.8 |  | 98.5(91.3,99.9) | 99.5(91.7,100.0) | 92.1(86.4,95.6) |
| Hubei | 420102 | Jiang'an District | 5.7 | 4.8 | 6.6 |  | 98.3(95.4,99.5) | 96.6(91.8,98.9) | 98.7(95.3,99.8) |
| Hubei | 420204 | Xialu District | 5.3 | 4.6 | 5.9 |  | 97.4(88.8,99.6) | 93.9(79.2,99.1) | 98.4(87.2,99.9) |
| Hubei | 420202 | Huangshigang District | 5.0 | 4.1 | 6.0 |  | 97.0(87.3,99.5) | 98.3(90.1,99.9) | 88.3(67.8,98.1) |
| Hubei | 420902 | Xiaonan City | 6.1 | 5.3 | 6.9 |  | 96.9(93.4,98.8) | 95.8(91.0,98.4) | 97.2(92.8,99.1) |
| Hubei | 421102 | Huangzhou District | 5.9 | 5.0 | 6.8 |  | 96.7(90.2,99.2) | 94.8(85.7,98.8) | 97.4(89.6,99.7) |
| Hubei | 420114 | Caidian District | 6.9 | 6.5 | 7.3 |  | 96.4(92.3,98.6) | 95.0(88.8,98.2) | 97.0(92.4,99.2) |
| Hubei | 420322 | Yunxi County | 8.5 | 6.9 | 9.9 |  | 94.3(90.3,97.0) | 95.0(91.0,97.7) | 91.1(84.8,95.5) |
| Hubei | 421223 | Chongyang County | 6.0 | 5.0 | 7.0 |  | 94.1(89.3,97.0) | 94.6(89.6,97.8) | 92.5(86.0,96.4) |
| Hubei | 420503 | Wujiagang District | 4.6 | 3.7 | 5.5 |  | 93.9(80.0,98.9) | 96.7(85.0,99.7) | 83.3(61.4,97.3) |
| Hubei | 420205 | Tieshan District | 7.2 | 5.6 | 8.7 |  | 93.8(66.2,99.8) | 78.4(67.9,86.4) | 99.0(56.5,100.0) |
| Hubei | 421182 | Wuxue City | 7.3 | 6.4 | 8.2 |  | 93.2(88.5,96.4) | 91.5(85.8,95.6) | 94.0(88.2,97.2) |
| Hubei | 420606 | Fancheng District | 5.3 | 4.4 | 6.3 |  | 92.9(79.0,98.6) | 94.0(83.9,98.6) | 90.1(75.4,97.2) |
| Hubei | 421022 | Gong'an County | 8.1 | 6.9 | 9.2 |  | 92.7(88.1,95.8) | 92.1(86.9,95.7) | 92.2(86.1,95.9) |
| Hubei | 420602 | Xiangcheng District | 5.6 | 4.5 | 6.8 |  | 92.5(78.4,98.7) | 94.0(81.3,99.3) | 88.1(68.1,97.5) |
| Hubei | 420625 | Gucheng County | 8.0 | 6.4 | 9.6 |  | 92.1(87.3,95.4) | 91.7(86.5,95.2) | 91.4(85.0,95.4) |
| Hubei | 421002 | Shashi District | 5.4 | 4.6 | 6.2 |  | 92.1(84.5,96.9) | 93.9(85.5,98.0) | 88.9(78.5,95.5) |
| Hubei | 421202 | Xian'an District | 6.2 | 5.3 | 7.0 |  | 92.0(86.1,95.7) | 92.3(86.7,96.4) | 90.1(83.3,95.0) |
| Hubei | 421281 | Chibi City | 6.5 | 5.7 | 7.3 |  | 91.5(85.3,95.4) | 91.6(85.3,96.1) | 89.4(81.5,95.2) |
| Hubei | 420325 | Fang County | 8.4 | 7.0 | 9.7 |  | 91.4(85.3,95.4) | 91.6(85.6,96.0) | 87.6(79.2,93.5) |
| Hubei | 420104 | Qiaokou District | 4.9 | 4.1 | 5.7 |  | 90.5(83.2,95.7) | 87.1(78.1,92.9) | 93.7(86.0,98.1) |
| Hubei | 420922 | Dawu County | 6.9 | 5.7 | 8.1 |  | 90.3(84.5,94.1) | 89.9(83.3,94.3) | 89.5(82.3,94.5) |
| Hubei | 420982 | Anlu City | 7.0 | 6.3 | 7.8 |  | 90.1(83.5,94.7) | 89.8(82.8,94.7) | 89.6(81.2,94.9) |
| Hubei | 420921 | Xiaochang City | 6.1 | 5.6 | 6.7 |  | 90.0(84.1,94.1) | 89.1(82.2,93.9) | 90.3(83.4,94.9) |
| Hubei | 429004 | Xiantao City | 7.6 | 7.2 | 8.0 |  | 89.6(83.7,93.7) | 88.4(81.3,93.1) | 89.5(83.3,94.2) |
| Hubei | 421222 | Tongcheng County | 7.1 | 5.9 | 8.3 |  | 89.4(83.1,93.6) | 90.3(84.4,94.3) | 86.3(77.6,92.2) |
| Hubei | 429006 | Tianmen City | 7.5 | 7.0 | 7.9 |  | 89.3(84.2,92.8) | 86.1(80.4,90.8) | 92.0(87.0,95.2) |
| Hubei | 420684 | Yicheng City | 6.3 | 5.4 | 7.1 |  | 89.1(82.3,93.5) | 92.5(86.7,96.4) | 82.0(73.8,88.2) |
| Hubei | 420703 | Huarong District | 6.6 | 6.2 | 7.0 |  | 89.0(80.4,94.5) | 85.9(77.2,93.0) | 91.3(82.4,96.8) |
| Hubei | 421003 | Jingzhou District | 6.1 | 5.4 | 6.7 |  | 89.0(81.6,94.8) | 88.8(79.8,95.1) | 90.0(81.3,95.7) |
| Hubei | 420506 | Yiling District | 7.4 | 6.5 | 8.4 |  | 88.9(81.5,94.0) | 87.5(78.3,93.6) | 89.6(81.0,95.2) |
| Hubei | 420821 | Jingshan County | 6.7 | 6.0 | 7.4 |  | 88.6(82.0,93.5) | 89.3(81.1,94.5) | 87.1(78.8,93.0) |
| Hubei | 420704 | Echeng District | 4.3 | 3.7 | 4.8 |  | 88.3(80.5,94.5) | 89.6(80.6,95.5) | 84.5(74.0,92.4) |
| Hubei | 420682 | Laohekou City | 6.6 | 5.5 | 7.7 |  | 87.6(80.8,92.6) | 87.3(80.5,93.1) | 87.5(78.7,93.5) |
| Hubei | 420981 | Yingcheng City | 7.0 | 6.2 | 7.8 |  | 87.3(80.1,92.8) | 83.7(75.0,89.9) | 89.8(81.7,95.0) |
| Hubei | 421087 | Songzi City | 6.5 | 5.4 | 7.6 |  | 86.9(79.2,92.2) | 90.2(83.3,94.9) | 80.5(69.0,88.7) |
| Hubei | 420203 | Xiseshan District | 5.9 | 4.5 | 7.3 |  | 86.7(75.6,94.6) | 90.1(78.4,97.2) | 79.3(61.9,92.3) |
| Hubei | 420502 | Xiling District | 4.4 | 3.6 | 5.2 |  | 86.7(74.9,95.0) | 87.9(72.0,96.6) | 83.4(64.9,94.6) |
| Hubei | 421127 | Huangmei County | 6.5 | 5.6 | 7.4 |  | 86.7(79.4,92.3) | 85.2(77.2,91.3) | 86.9(77.9,93.0) |
| Hubei | 420381 | Danjiangkou City | 6.4 | 4.9 | 7.8 |  | 86.6(79.4,92.0) | 86.0(78.2,91.7) | 86.2(76.9,93.0) |
| Hubei | 420881 | Zhongxiang City | 6.0 | 5.4 | 6.7 |  | 86.5(79.7,91.4) | 86.7(79.2,92.0) | 86.4(79.1,92.0) |
| Hubei | 420302 | Maojian District | 3.3 | 2.6 | 4.0 |  | 86.1(65.3,97.8) | 88.2(65.3,98.5) | 81.2(58.7,95.9) |
| Hubei | 420702 | Liangzihu District | 5.6 | 4.8 | 6.3 |  | 85.9(75.1,93.0) | 85.3(74.7,93.5) | 85.8(72.0,95.0) |
| Hubei | 420583 | Zhijiang City | 7.1 | 6.1 | 8.1 |  | 85.8(75.8,92.7) | 90.7(80.4,96.8) | 74.3(61.3,84.5) |
| Hubei | 421122 | Hong'an County | 6.5 | 5.6 | 7.4 |  | 85.8(77.7,90.9) | 87.1(79.9,92.8) | 81.7(71.5,89.4) |
| Hubei | 420607 | Xiangyang District | 6.0 | 5.1 | 6.9 |  | 85.7(72.6,94.7) | 86.6(73.2,95.1) | 84.5(70.7,94.3) |
| Hubei | 421125 | Xishui County | 6.4 | 5.7 | 7.1 |  | 85.7(78.5,91.2) | 85.2(77.6,91.0) | 85.9(78.2,91.4) |
| Hubei | 420802 | Dongbao District | 5.6 | 4.8 | 6.3 |  | 84.9(67.3,95.8) | 74.7(58.5,88.8) | 92.4(74.8,98.9) |
| Hubei | 420281 | Daye City | 4.9 | 4.2 | 5.5 |  | 84.5(77.3,90.6) | 84.4(76.5,90.5) | 82.9(73.0,90.2) |
| Hubei | 421303 | Cengdu District | 5.9 | 5.0 | 6.8 |  | 84.0(75.7,89.5) | 83.3(73.9,89.2) | 84.3(75.8,91.1) |
| Hubei | 421381 | Guangshui City | 6.2 | 5.1 | 7.2 |  | 84.0(77.4,89.6) | 84.8(78.0,90.6) | 81.9(72.6,88.6) |
| Hubei | 420321 | Yun County | 7.3 | 6.2 | 8.3 |  | 83.8(75.9,89.1) | 84.5(76.7,90.0) | 80.9(72.6,87.7) |
| Hubei | 421023 | Jianli County | 7.7 | 7.0 | 8.4 |  | 83.7(75.3,89.5) | 83.0(75.4,89.1) | 82.6(73.3,89.5) |
| Hubei | 420581 | Yidu City | 8.1 | 6.9 | 9.3 |  | 83.3(74.6,90.2) | 80.0(69.5,89.0) | 84.6(72.6,93.4) |
| Hubei | 421321 | Sui County | 6.9 | 6.0 | 7.8 |  | 83.2(68.1,94.0) | 81.6(64.4,93.0) | 84.6(69.1,94.4) |
| Hubei | 421024 | Jiangling County | 7.0 | 6.5 | 7.4 |  | 83.0(67.0,94.2) | 83.9(67.3,95.9) | 80.9(64.4,93.4) |
| Hubei | 421081 | Shishou City | 6.9 | 6.0 | 7.7 |  | 82.9(74.2,89.4) | 83.4(74.1,90.2) | 80.9(70.7,89.3) |
| Hubei | 420683 | Zaoyang City | 6.9 | 5.9 | 7.9 |  | 82.8(75.9,88.3) | 81.4(73.7,87.0) | 84.1(76.0,90.2) |
| Hubei | 421181 | Macheng City | 8.6 | 7.3 | 9.8 |  | 82.6(76.0,88.2) | 80.9(73.2,86.9) | 81.7(73.6,88.4) |
| Hubei | 420923 | Yunmeng County | 6.8 | 6.4 | 7.2 |  | 81.5(73.8,88.0) | 78.4(69.5,85.4) | 85.6(76.9,91.8) |
| Hubei | 420504 | Dianjun District | 7.6 | 6.4 | 8.9 |  | 80.9(72.3,87.4) | 83.1(74.1,89.0) | 79.6(69.7,86.9) |
| Hubei | 421126 | Qichun County | 6.6 | 5.6 | 7.5 |  | 80.9(73.0,87.0) | 78.7(70.4,85.9) | 83.2(73.8,89.8) |
| Hubei | 420303 | Zhangwan District | 4.0 | 3.3 | 4.7 |  | 80.6(57.3,95.6) | 77.3(54.5,94.7) | 82.4(59.2,96.9) |
| Hubei | 420525 | Yuan'an County | 7.4 | 6.6 | 8.2 |  | 80.5(68.8,89.6) | 76.6(62.2,87.8) | 82.0(68.4,92.1) |
| Hubei | 421083 | Honghu City | 6.9 | 6.5 | 7.2 |  | 80.5(72.0,87.6) | 79.3(69.0,86.4) | 81.4(69.8,89.1) |
| Hubei | 420222 | Yangxin County | 5.0 | 4.0 | 6.0 |  | 80.3(71.5,87.4) | 79.1(71.1,85.7) | 82.1(72.2,89.5) |
| Hubei | 420526 | Xingshan County | 8.1 | 7.1 | 9.1 |  | 80.1(68.1,89.2) | 80.5(67.6,90.8) | 76.8(62.9,87.3) |
| Hubei | 420984 | Hanchuan City | 6.6 | 6.2 | 7.0 |  | 79.8(71.0,86.6) | 76.4(67.7,83.3) | 83.8(75.5,90.1) |
| Hubei | 420822 | Shayang County | 6.6 | 5.8 | 7.3 |  | 79.3(61.6,91.5) | 77.5(60.5,90.0) | 81.9(63.7,92.9) |
| Hubei | 429005 | Qianjiang City | 6.3 | 5.6 | 6.9 |  | 78.8(69.9,86.3) | 80.4(70.4,87.5) | 78.7(69.2,85.8) |
| Hubei | 420528 | Zizhixian | 8.1 | 6.9 | 9.2 |  | 78.3(68.1,86.2) | 80.7(69.8,89.0) | 72.1(60.0,81.9) |
| Hubei | 420624 | Nanzhang County | 6.5 | 5.5 | 7.5 |  | 76.8(67.1,84.4) | 75.0(65.8,83.5) | 78.2(67.8,86.8) |
| Hubei | 420529 | Zizhixian | 8.7 | 7.6 | 9.8 |  | 76.0(64.9,84.7) | 75.7(63.1,86.1) | 72.9(59.0,84.2) |
| Hubei | 420527 | Zigui County | 8.3 | 6.9 | 9.7 |  | 75.7(65.7,83.6) | 80.3(70.3,88.3) | 64.4(53.7,75.2) |
| Hubei | 420324 | Zhuxi County | 6.6 | 5.4 | 7.6 |  | 75.4(64.9,84.0) | 75.3(63.9,83.6) | 74.8(62.1,85.0) |
| Hubei | 421221 | Jiayu County | 6.4 | 5.7 | 7.1 |  | 75.4(65.5,84.5) | 76.8(66.3,85.4) | 74.0(62.2,83.7) |
| Hubei | 420323 | Zhushan County | 7.9 | 6.8 | 8.8 |  | 74.7(64.8,82.1) | 73.2(63.7,81.0) | 73.8(64.2,82.7) |
| Hubei | 420103 | Jianghan District | 3.6 | 3.0 | 4.2 |  | 74.3(62.1,85.2) | 74.8(61.4,87.1) | 74.3(58.6,87.8) |
| Hubei | 420804 | Duodao District | 3.6 | 3.0 | 4.1 |  | 73.4(50.6,91.4) | 68.7(46.2,89.5) | 80.0(56.3,96.4) |
| Hubei | 421124 | Yingshan County | 6.2 | 5.0 | 7.4 |  | 73.1(60.5,83.6) | 74.5(62.4,85.7) | 67.5(51.9,81.4) |
| Hubei | 422825 | Xuan'en County | 8.3 | 7.0 | 9.6 |  | 72.8(62.7,80.5) | 72.4(62.0,81.7) | 68.8(56.9,78.8) |
| Hubei | 421224 | Tongshan County | 5.0 | 4.2 | 5.6 |  | 72.6(61.7,81.3) | 72.9(62.0,82.2) | 74.2(61.9,83.0) |
| Hubei | 429021 | Shennongjia Linqu | 7.5 | 6.5 | 8.3 |  | 70.7(54.2,84.8) | 61.4(45.2,78.8) | 76.9(55.8,92.6) |
| Hubei | 420582 | Dangyang City | 7.1 | 6.3 | 8.0 |  | 70.5(59.7,79.3) | 73.5(61.8,82.8) | 66.1(54.2,75.9) |
| Hubei | 420105 | Hanyang District | 2.3 | 1.8 | 2.8 |  | 70.1(53.2,86.1) | 72.7(53.6,87.7) | 63.6(43.4,84.3) |
| Hubei | 422801 | Enshi City | 7.0 | 5.8 | 8.1 |  | 69.5(59.8,77.6) | 71.2(61.6,80.3) | 65.2(53.7,74.4) |
| Hubei | 422827 | Laifeng County | 7.4 | 6.0 | 8.9 |  | 68.2(57.4,77.9) | 73.6(62.8,82.3) | 57.1(45.5,69.0) |
| Hubei | 422802 | Lichuan City | 8.1 | 6.6 | 9.5 |  | 67.8(58.6,75.7) | 68.1(57.6,76.4) | 64.9(54.4,73.5) |
| Hubei | 422822 | Jianshi County | 8.1 | 6.7 | 9.5 |  | 67.7(57.5,76.8) | 68.0(57.2,77.6) | 63.5(52.1,74.5) |
| Hubei | 420626 | Baokang County | 7.9 | 6.8 | 9.0 |  | 67.6(56.3,77.8) | 68.6(56.1,79.1) | 64.1(52.0,75.2) |
| Hubei | 422826 | Xianfeng County | 8.0 | 6.6 | 9.3 |  | 67.2(56.5,76.8) | 70.1(59.8,79.5) | 60.3(49.2,71.7) |
| Hubei | 422823 | Badong County | 8.6 | 7.0 | 10.1 |  | 66.5(57.0,75.4) | 67.1(56.8,76.1) | 62.2(50.0,72.0) |
| Hubei | 420115 | Jiangxia District | 2.6 | 2.2 | 2.9 |  | 65.4(53.8,76.4) | 69.9(56.4,79.9) | 62.6(50.4,74.5) |
| Hubei | 421123 | Luotian County | 5.7 | 5.1 | 6.3 |  | 64.6(54.1,73.3) | 64.8(54.6,74.8) | 64.0(52.6,73.1) |
| Hubei | 420107 | Qingshan District | 4.4 | 3.3 | 5.4 |  | 63.3(49.4,77.9) | 68.9(54.2,83.0) | 54.2(36.9,76.1) |
| Hubei | 422828 | Hefeng County | 7.5 | 6.6 | 8.4 |  | 59.6(39.0,80.9) | 59.9(39.2,80.2) | 57.1(36.4,78.0) |
| Hubei | 421121 | Tuanfeng County | 5.4 | 4.9 | 5.9 |  | 50.6(40.2,61.0) | 51.0(39.1,62.4) | 50.7(39.9,61.7) |
| Hubei | 420106 | Wuchang District | 2.3 | 1.8 | 2.8 |  | 46.6(36.2,59.8) | 52.5(39.4,66.0) | 41.7(30.9,57.4) |
| Hubei | 420111 | Hongshan District | 0.8 | 0.6 | 1.0 |  | 43.3(31.7,56.6) | 43.3(30.9,56.6) | 48.3(36.5,62.4) |
| Hubei | 420112 | Dongxihu District | 0.4 | 0.3 | 0.5 |  | 31.1(20.7,46.0) | 27.3(17.6,40.8) | 40.3(25.0,63.6) |
| Hubei | 420113 | Hannan District | 0.2 | 0.2 | 0.2 |  | 16.8(10.8,25.1) | 14.1(9.5,20.8) | 23.2(14.0,38.4) |
| Hubei | 420116 | Huangpi District | 0.8 | 0.5 | 1.1 |  | 13.6(9.0,19.9) | 14.8(9.8,21.9) | 13.5(9.1,19.4) |
| Hubei | 420117 | Xinzhou District | 0.2 | 0.1 | 0.2 |  | 10.8(7.2,16.5) | 11.7(7.6,17.5) | 11.8(7.9,17.0) |
| Ningxia | 640381 | Qingtongxia City | 5.6 | 4.9 | 6.3 |  | 95.0(89.9,97.8) | 93.1(84.7,97.5) | 95.1(88,98.5) |
| Ningxia | 640121 | Yongning County | 5.0 | 4.2 | 5.8 |  | 94.8(87.8,98.3) | 94.0(83.2,98.6) | 94.8(85.2,98.9) |
| Ningxia | 640303 | Hongsibao District | 5.0 | 4.5 | 5.6 |  | 94.8(87.0,98.4) | 90.5(80.0,97.3) | 97.3(91.2,99.4) |
| Ningxia | 640221 | Pingluo County | 6.3 | 5.5 | 7.0 |  | 94.1(88.2,97.5) | 94.3(86.6,98.3) | 92.6(84.3,97.5) |
| Ningxia | 640106 | Jinfeng District | 4.0 | 3.3 | 4.7 |  | 93.4(84.2,97.7) | 96.3(86.8,99.3) | 85.4(71.9,94.3) |
| Ningxia | 640122 | Helan County | 5.7 | 5.1 | 6.2 |  | 91.5(84.1,95.9) | 89.7(79.5,95.7) | 92.6(83.6,97.4) |
| Ningxia | 640105 | Xixia District | 3.9 | 3.1 | 4.7 |  | 90.7(76.7,97.7) | 90.5(71.2,98.3) | 87.8(65.5,97.4) |
| Ningxia | 640202 | Dawukou District | 5.3 | 4.3 | 6.1 |  | 89.7(76.8,96.9) | 91.2(77.2,97.8) | 84.8(67.9,95.8) |
| Ningxia | 640205 | Huinong District | 6.1 | 5.2 | 6.8 |  | 89.4(76.1,97.0) | 89.9(72.6,97.8) | 86.3(66.5,96.7) |
| Ningxia | 640323 | Yanchi County | 5.0 | 4.5 | 5.4 |  | 82.0(69.1,91.3) | 62.6(49.8,75.3) | 94.8(83.3,99.0) |
| Ningxia | 640521 | Zhongning County | 5.2 | 4.1 | 6.3 |  | 81.3(73.8,87.6) | 83.6(74.3,90.2) | 78.4(67.5,87.7) |
| Ningxia | 640402 | Yuanzhou District | 5.1 | 4.3 | 5.9 |  | 81.0(73.5,87.0) | 83.0(75.1,89.2) | 78.2(69.1,86.0) |
| Ningxia | 640502 | Zhongwei City Chengqu | 5.3 | 4.5 | 6.1 |  | 79.0(70.0,85.6) | 74.9(64.4,84.0) | 82.4(71.9,90.4) |
| Ningxia | 640522 | Haiyuan County | 4.7 | 4.0 | 5.4 |  | 73.6(66.2,80.0) | 71.4(61.1,79.4) | 76.0(67.1,83.5) |
| Ningxia | 640324 | Tongxin County | 3.5 | 3.3 | 3.7 |  | 72.2(64.3,80.2) | 64.6(54.4,75.3) | 80.2(70.0,87.7) |
| Ningxia | 640425 | Pengyang County | 4.7 | 3.6 | 5.8 |  | 71.3(60.6,82.0) | 66.6(53.9,77.7) | 76.3(61.0,89.5) |
| Ningxia | 640104 | Xingqing District | 3.3 | 2.7 | 3.9 |  | 71.1(61.5,78.8) | 69.7(59.6,78.8) | 75.2(64.0,83.7) |
| Ningxia | 640422 | Xiji County | 4.7 | 4.1 | 5.3 |  | 70.0(61.3,76.9) | 67.1(56.5,75.9) | 74.1(64.4,82.0) |
| Ningxia | 640181 | Lingwu City | 3.7 | 3.4 | 4.0 |  | 69.7(58.8,79.6) | 66.2(53.2,78.7) | 74.9(61.7,85.5) |
| Ningxia | 640302 | Litong District | 3.9 | 3.4 | 4.4 |  | 69.7(59.6,78.2) | 68.0(55.8,77.9) | 72.4(60.6,81.3) |
| Ningxia | 640423 | Longde County | 5.2 | 4.3 | 6.1 |  | 66.9(54.2,78.2) | 61.0(48.0,73.4) | 72.5(54.9,87.1) |
| Ningxia | 640424 | Jingyuan County | 4.7 | 3.7 | 5.8 |  | 62.7(48.5,76.0) | 68.2(51.6,83.2) | 54.2(38.6,73.7) |
| Guangxi | 450102 | Xingning District | 5.8 | 4.7 | 6.8 |  | 99.9(99.4,100.0) | 99.9(99.5,100.0) | 99.2(96.8,99.9) |
| Guangxi | 450103 | Qingxiu District | 6.0 | 4.7 | 7.2 |  | 99.7(99.2,99.9) | 99.9(99.6,100.0) | 97.9(94.3,99.4) |
| Guangxi | 451321 | Xincheng County | 17.2 | 14.6 | 19.6 |  | 99.2(98.3,99.7) | 99.0(97.3,99.6) | 98.8(97.4,99.5) |
| Guangxi | 450108 | Liangqing District | 4.8 | 4.2 | 5.3 |  | 98.1(95.1,99.4) | 97.7(93.1,99.5) | 97.9(93.2,99.5) |
| Guangxi | 450802 | Gangbei District | 6.2 | 4.9 | 7.5 |  | 97.2(94.0,98.9) | 97.7(94.7,99.3) | 95.5(89.4,98.4) |
| Guangxi | 450105 | Jiangnan District | 5.1 | 4.4 | 5.7 |  | 96.6(92.8,98.8) | 97.5(93.5,99.2) | 93.8(86.5,97.9) |
| Guangxi | 450107 | Xixiangtang District | 4.3 | 3.5 | 5.0 |  | 96.6(93.6,98.6) | 97.1(93.8,98.9) | 94.4(88.9,97.7) |
| Guangxi | 450303 | Diecai District | 5.7 | 4.3 | 7.1 |  | 96.5(88.9,99.2) | 95.7(85.1,99.2) | 96.2(82.9,99.6) |
| Guangxi | 450311 | Yanshan District | 4.8 | 3.8 | 5.9 |  | 96.0(81.1,99.6) | 98.6(84.4,100) | 86.0(63.8,98.3) |
| Guangxi | 450502 | Haicheng District | 4.8 | 4.0 | 5.6 |  | 95.8(90.1,98.5) | 96.7(90.9,99.2) | 93.2(83.7,97.7) |
| Guangxi | 450323 | Lingchuan County | 7.6 | 7.0 | 8.2 |  | 95.2(91.5,97.6) | 95.4(90.6,98.0) | 94.3(88.4,97.3) |
| Guangxi | 450203 | Yufeng District | 5.7 | 4.8 | 6.5 |  | 94.8(86.1,98.6) | 96.4(87.9,99.4) | 91.3(80.1,97.4) |
| Guangxi | 451002 | Youjiang District | 6.1 | 5.1 | 7.1 |  | 94.6(89.8,97.7) | 96.7(91.8,98.9) | 90.3(81.8,95.8) |
| Guangxi | 450302 | Xiufeng District | 5.1 | 3.9 | 6.4 |  | 94.5(82.1,98.9) | 93.8(80.3,99.0) | 93.8(71.8,99.5) |
| Guangxi | 450804 | Tantang District | 6.8 | 5.6 | 7.9 |  | 94.1(88.6,97.3) | 94.7(89.6,97.6) | 93.0(86.3,97.1) |
| Guangxi | 450223 | Luzhai County | 8.2 | 7.1 | 9.1 |  | 93.8(89.6,96.4) | 93.6(88.9,96.6) | 92.7(87.9,96.1) |
| Guangxi | 450503 | Yinhai District | 3.5 | 2.6 | 4.3 |  | 92.9(83.3,97.8) | 94.8(83.8,98.9) | 86.1(67.4,95.9) |
| Guangxi | 450521 | Hepu County | 5.7 | 4.7 | 6.7 |  | 92.9(89.4,95.6) | 93.3(89.3,96.1) | 90.7(85.4,94.8) |
| Guangxi | 450205 | Liubei District | 5.6 | 4.4 | 6.8 |  | 92.6(83.7,97.7) | 96.0(88.1,99.3) | 84.5(71.1,94.4) |
| Guangxi | 450126 | Binyang County | 7.8 | 6.7 | 8.8 |  | 92.1(86.4,95.9) | 91.5(85.8,95.1) | 91.7(85.9,96.1) |
| Guangxi | 450703 | Qinbei District | 8.0 | 6.5 | 9.3 |  | 91.8(87.7,94.7) | 92.3(88.5,95.0) | 90.6(85.5,94.0) |
| Guangxi | 450902 | Yuzhou District | 4.2 | 3.3 | 5.0 |  | 91.6(86.6,95.0) | 93.6(88.9,96.4) | 87.9(80.2,93.3) |
| Guangxi | 450603 | Fangcheng District | 5.9 | 4.4 | 7.3 |  | 91.5(86.7,94.8) | 92.9(87.8,96.2) | 88.6(80.6,94.1) |
| Guangxi | 450405 | Changzhou District | 5.6 | 4.9 | 6.2 |  | 90.8(79.2,97.3) | 89.1(76.3,96.8) | 91.2(78.3,98.0) |
| Guangxi | 451202 | Jinchengjiang District | 6.0 | 4.9 | 7.1 |  | 90.8(84.6,95.0) | 87.1(79.2,92.6) | 93.2(85.2,97.4) |
| Guangxi | 450331 | Lipu County | 8.0 | 6.8 | 9.1 |  | 90.3(83.9,94.5) | 89.2(81.8,94.1) | 89.9(80.7,94.8) |
| Guangxi | 450222 | Liucheng County | 6.8 | 5.4 | 8.0 |  | 90.2(84.5,94.5) | 92.0(86.1,96.2) | 85.5(75.6,92.4) |
| Guangxi | 450304 | Xiangshan District | 4.7 | 3.6 | 5.8 |  | 89.8(79.5,95.9) | 94.6(84.9,98.7) | 77.5(63.5,88.4) |
| Guangxi | 450305 | Qixing District | 3.4 | 3.0 | 3.8 |  | 89.7(77.6,96.5) | 84.1(67.5,94.5) | 93.3(79.6,98.5) |
| Guangxi | 450923 | Bobai County | 6.4 | 5.3 | 7.5 |  | 89.6(81.9,94.3) | 90.3(83.4,94.9) | 88.4(80.4,93.7) |
| Guangxi | 450122 | Wuming County | 7.3 | 6.3 | 8.2 |  | 89.5(83.9,93.6) | 89.7(84.1,93.9) | 87.3(79.9,92.6) |
| Guangxi | 450124 | Mashan County | 7.6 | 6.1 | 9.2 |  | 89.5(84.2,93.1) | 92.3(87.9,95.4) | 82.5(74.0,88.7) |
| Guangxi | 451302 | Laibin District | 6.4 | 5.1 | 7.6 |  | 89.5(85.1,92.7) | 91.4(87.3,94.3) | 86.9(80.4,90.8) |
| Guangxi | 450330 | Pingle County | 7.8 | 6.5 | 9.0 |  | 88.6(80.9,94.1) | 88.9(80.9,93.9) | 85.8(75.7,92.8) |
| Guangxi | 451423 | Longzhou County | 6.9 | 5.7 | 8.0 |  | 87.7(78.9,94.3) | 89.4(80.1,95.1) | 82.3(67.2,93.2) |
| Guangxi | 450127 | Heng County | 7.9 | 6.3 | 9.5 |  | 87.6(80.4,92.3) | 90.1(84.2,94.1) | 81.6(71.9,88.4) |
| Guangxi | 450803 | Gangnan District | 6.0 | 5.1 | 7.0 |  | 87.4(79.7,93.2) | 87.7(79.6,92.5) | 87.8(78.6,93.6) |
| Guangxi | 450204 | Liunan District | 4.9 | 3.9 | 5.9 |  | 87.0(76.5,94.0) | 92.2(82.1,97.8) | 79.3(66.7,89.7) |
| Guangxi | 450922 | Luchuan County | 5.4 | 4.3 | 6.5 |  | 87.0(81.9,91.1) | 88.8(83.3,92.4) | 84.7(77.9,89.8) |
| Guangxi | 450702 | Qinnan District | 6.0 | 4.9 | 6.9 |  | 86.8(80.7,91.4) | 88.2(82.2,92.5) | 85.0(78.5,90.4) |
| Guangxi | 451027 | Lingyun County | 6.8 | 5.8 | 7.7 |  | 86.6(78.6,92.3) | 83.0(73.2,90.5) | 88.7(78.2,95.7) |
| Guangxi | 450681 | Dongxing City | 4.3 | 3.7 | 4.8 |  | 86.5(74.4,94.2) | 92.5(81.0,98.2) | 73.2(60.4,84.7) |
| Guangxi | 450602 | Gangkou District | 4.5 | 3.7 | 5.1 |  | 86.4(76.2,93.4) | 82.3(70.0,91.2) | 90.7(79.1,96.9) |
| Guangxi | 451421 | Fusui County | 6.6 | 5.9 | 7.3 |  | 86.2(79.6,91.4) | 84.9(78.4,90.3) | 88.1(79.7,93.8) |
| Guangxi | 450125 | Shanglin County | 7.9 | 6.9 | 8.9 |  | 86.1(80.2,90.4) | 85.4(78.5,90.7) | 85.9(78.5,91.8) |
| Guangxi | 450221 | Liujiang County | 4.9 | 4.2 | 5.6 |  | 86.1(79.7,90.9) | 83.7(75.5,89.6) | 87.6(79.8,93.3) |
| Guangxi | 450332 | Zizhixian | 7.3 | 5.9 | 8.7 |  | 86.1(78.6,91.7) | 85.0(75.8,91.5) | 84.4(73.8,92.2) |
| Guangxi | 450722 | Pubei County | 6.2 | 5.0 | 7.2 |  | 85.7(80.9,89.8) | 88.2(82.2,91.9) | 82.0(74.4,87.1) |
| Guangxi | 450512 | Tieshangang District | 5.6 | 4.6 | 6.5 |  | 85.3(74.0,93.0) | 82.4(71.0,91.3) | 88.6(74.1,96.7) |
| Guangxi | 450324 | Quanzhou County | 6.6 | 5.6 | 7.6 |  | 85.1(76.1,91.7) | 84.0(74.8,90.7) | 85.0(74.9,92.6) |
| Guangxi | 450423 | Meng shan County | 6.7 | 5.6 | 7.8 |  | 85.1(76.3,91.5) | 87.0(77.5,93.4) | 80.3(68.4,89.2) |
| Guangxi | 451225 | Zizhixian | 7.5 | 6.1 | 8.8 |  | 84.6(77.1,89.7) | 88.1(80.9,93.0) | 76.6(66.0,85.0) |
| Guangxi | 450421 | Cangwu County | 6.5 | 5.0 | 7.9 |  | 83.7(76.5,89.3) | 89.1(82.5,93.6) | 72.6(63.0,80.7) |
| Guangxi | 450921 | Rong County | 6.7 | 5.1 | 8.2 |  | 83.2(75.4,89.7) | 85.5(77.7,90.8) | 80.6(70.3,87.9) |
| Guangxi | 450881 | Guiping City | 6.2 | 5.0 | 7.3 |  | 83.0(77.8,87.5) | 84.0(78.2,87.9) | 82.5(76.7,87.6) |
| Guangxi | 451102 | Babu District | 5.6 | 4.6 | 6.6 |  | 82.2(75.4,87.2) | 84.5(78.5,89.9) | 78.7(70.7,85.0) |
| Guangxi | 450123 | Long'an County | 8.0 | 7.1 | 8.8 |  | 82.0(75.1,87.3) | 82.7(75.3,88.1) | 79.8(71.2,86.6) |
| Guangxi | 450621 | Shangsi County | 6.0 | 5.0 | 6.9 |  | 82.0(72.1,89.0) | 80.8(71.7,88.6) | 82.9(70.2,91.4) |
| Guangxi | 450821 | Pingnan County | 5.7 | 4.6 | 6.7 |  | 81.9(75.9,86.8) | 84.6(78.6,89.0) | 78.4(71.2,84.7) |
| Guangxi | 451021 | Tianyang County | 6.5 | 5.7 | 7.2 |  | 81.6(74.1,88.1) | 81.5(72.2,88.3) | 82.4(71.1,90.6) |
| Guangxi | 451424 | Daxin County | 7.7 | 6.6 | 8.8 |  | 81.3(72.9,87.5) | 84.2(76.2,90.2) | 76.3(64.9,84.4) |
| Guangxi | 450109 | Yongning District | 5.9 | 4.9 | 6.9 |  | 81.0(71.6,87.6) | 82.8(73.4,90.1) | 76.9(65.4,86.7) |
| Guangxi | 450481 | Cenxi City | 5.9 | 4.9 | 6.8 |  | 81.0(74.3,86.2) | 82.3(75.8,87.3) | 79.1(71.1,84.8) |
| Guangxi | 451022 | Tiandong County | 7.4 | 6.2 | 8.5 |  | 81.0(73.8,86.7) | 81.6(74.0,87.1) | 80.2(70.6,87.3) |
| Guangxi | 450403 | Wanxiu District | 5.9 | 5.1 | 6.7 |  | 80.1(66.9,90.7) | 85.4(70.9,94.6) | 71.8(58.0,84.3) |
| Guangxi | 450202 | Chengzhong District | 4.1 | 3.5 | 4.8 |  | 78.9(66.3,89.6) | 77.7(64.2,88.4) | 83.9(71.4,93.6) |
| Guangxi | 450322 | Lin'gui County | 3.3 | 3.0 | 3.6 |  | 76.4(64.1,87.2) | 73.5(61.0,86.7) | 77.9(62.9,89.6) |
| Guangxi | 451123 | Zizhixian | 6.5 | 5.3 | 7.6 |  | 75.5(66.8,83.0) | 74.5(64.9,81.9) | 75.2(63.3,85.3) |
| Guangxi | 451381 | Heshan City | 7.0 | 5.0 | 8.8 |  | 75.5(65.2,84.1) | 81.1(70.2,89.3) | 66.3(53.6,79.9) |
| Guangxi | 450981 | Beiliu City | 4.3 | 3.7 | 4.9 |  | 75.0(67.7,80.8) | 75.2(67.5,81.7) | 77.1(69.5,83.3) |
| Guangxi | 451481 | Pingxiang City | 5.0 | 4.3 | 5.7 |  | 73.3(59.6,86.3) | 67.5(54.7,78.2) | 80.4(62.8,94.4) |
| Guangxi | 451224 | Donglan County | 6.9 | 5.7 | 8.1 |  | 72.2(62.7,80.9) | 79.1(69.4,86.6) | 59.1(46.8,71.4) |
| Guangxi | 451281 | Yizhou City | 3.9 | 3.1 | 4.8 |  | 71.9(62.6,80.3) | 78.2(68.3,86.1) | 63.1(51.3,74.4) |
| Guangxi | 450321 | Yangshuo County | 5.8 | 4.9 | 6.6 |  | 71.5(58.8,82.4) | 77.3(64.4,87.3) | 63.2(50.3,76.3) |
| Guangxi | 451402 | Jiangzhou District | 4.7 | 4.0 | 5.3 |  | 71.4(61.7,80.1) | 74.1(62.8,83.0) | 68.0(55.2,80.4) |
| Guangxi | 451030 | Xilin County | 5.8 | 4.5 | 7.1 |  | 70.9(60.4,79.4) | 72.7(61.2,81.3) | 68.8(55.3,82.1) |
| Guangxi | 450325 | Xing'an County | 5.0 | 4.1 | 5.7 |  | 69.6(58.9,78.1) | 71.4(58.9,81.9) | 66.5(54.6,77.7) |
| Guangxi | 451221 | Nandan County | 4.3 | 3.5 | 4.9 |  | 68.8(57.4,78.5) | 71.9(60.0,81.7) | 63.9(50.5,76.8) |
| Guangxi | 450224 | Rong'an County | 5.7 | 4.8 | 6.4 |  | 68.1(57.4,77.8) | 67.0(55.6,77.8) | 67.3(53.6,80.5) |
| Guangxi | 450406 | Dieshan District | 4.2 | 3.3 | 5.0 |  | 64.7(50.8,77.3) | 67.2(53.7,80.7) | 62.8(48.3,78.4) |
| Guangxi | 451323 | Wuxuan County | 3.9 | 3.3 | 4.4 |  | 64.2(54.6,73.3) | 68.1(57.1,78.7) | 59.1(47.4,71.1) |
| Guangxi | 451023 | Pingguo County | 4.3 | 3.4 | 5.1 |  | 62.5(52.6,71.0) | 69.7(60.0,77.7) | 53.6(42.8,65.4) |
| Guangxi | 450326 | Yongfu County | 4.1 | 3.6 | 4.6 |  | 56.7(44.4,68.4) | 58.9(46.6,71.4) | 53.7(41.1,67.5) |
| Guangxi | 450226 | Zizhixian | 5.4 | 4.5 | 6.2 |  | 54.0(44.4,62.2) | 59.8(49.4,69.2) | 45.2(35.0,55.2) |
| Guangxi | 451322 | Xiangzhou County | 4.1 | 3.5 | 4.6 |  | 51.2(40.4,61.3) | 56.7(45.3,68.5) | 44.7(34.9,56.9) |
| Guangxi | 450721 | Lingshan County | 2.5 | 1.8 | 3.1 |  | 46.3(38.0,55.2) | 51.3(42.7,59.8) | 43.8(34.2,53.0) |
| Guangxi | 451422 | Ningming County | 3.6 | 2.9 | 4.1 |  | 45.2(33.9,57.8) | 52.2(39.6,65.0) | 40.4(29.8,53.4) |
| Guangxi | 451425 | Tiandeng County | 4.1 | 3.5 | 4.7 |  | 44.8(32.8,59.7) | 51.0(37.7,64.9) | 38.6(25.5,53.6) |
| Guangxi | 450903 | Yuzhou District | 0.7 | 0.4 | 0.9 |  | 42.7(31.7,56.6) | 41.4(30.0,55.7) | 48.8(34.9,67.4) |
| Guangxi | 450328 | Zizhixian | 2.8 | 2.3 | 3.3 |  | 42.1(28.7,57.9) | 41.5(27.4,61.1) | 42.2(26.1,65.6) |
| Guangxi | 451324 | Jinxiu Yaozu Zizhixian | 3.1 | 2.5 | 3.7 |  | 41.1(28.8,57.3) | 56.3(35.8,78.8) | 27.2(19.5,35.1) |
| Guangxi | 450225 | Zizhixian | 2.9 | 2.4 | 3.4 |  | 31.3(24.1,39.9) | 35.7(27.4,44.9) | 25.7(18.4,35.6) |
| Guangxi | 450924 | Xingye County | 1.2 | 0.9 | 1.6 |  | 29.0(20.8,38.3) | 34.0(24.9,45.0) | 24.7(17.1,34.5) |
| Guangxi | 451229 | Dahua Yaozu Zizhixian | 2.2 | 1.6 | 2.8 |  | 28.5(21.4,36.5) | 34.8(26.7,44.4) | 22.6(16.0,31.8) |
| Guangxi | 451228 | Du'an Yaozu Zizhixian | 2.7 | 2.2 | 3.2 |  | 26.9(20.6,34.2) | 35.1(28.0,43.9) | 21.0(15.7,27.3) |
| Guangxi | 450327 | Guanyang County | 2.5 | 1.9 | 3.0 |  | 24.3(17.3,33.1) | 27.7(18.9,39.1) | 20.3(14.3,28.4) |
| Guangxi | 450422 | Teng County | 0.9 | 0.6 | 1.2 |  | 19.2(13.8,25.4) | 20.2(15.0,27.2) | 19.9(14.3,27.3) |
| Guangxi | 451121 | Zhaoping County | 1.7 | 1.3 | 2.0 |  | 19.0(13.8,25.6) | 21.7(15.6,28.9) | 17.3(12.5,24.0) |
| Guangxi | 451024 | Debao County | 1.6 | 1.2 | 2.0 |  | 17.8(12.3,25.0) | 24.4(16.6,34.4) | 12.2(8.1,18.0) |
| Guangxi | 451222 | Tian'e County | 1.6 | 1.3 | 1.9 |  | 17.2(12.0,23.9) | 21.8(14.5,30.6) | 13.7(8.9,20.9) |
| Guangxi | 451223 | Fengshan County | 0.6 | 0.4 | 0.7 |  | 17.1(10.8,28.6) | 17.3(10.8,27.5) | 17.4(9.5,33.0) |
| Guangxi | 451026 | Napo County | 2.4 | 1.8 | 2.8 |  | 17.0(11.7,24.5) | 22.3(14.9,32.1) | 12.2(7.6,19.1) |
| Guangxi | 450329 | Ziyuan County | 0.6 | 0.4 | 0.8 |  | 16.6(9.8,27.4) | 18.7(10.3,33.0) | 14.0(8.6,25.6) |
| Guangxi | 451029 | Tianlin County | 1.2 | 0.9 | 1.5 |  | 16.2(11.3,22.8) | 20.6(14.1,29.6) | 13.1(8.7,19.9) |
| Guangxi | 451122 | Zhongshan County | 2.0 | 1.3 | 2.7 |  | 15.9(11.0,22.7) | 20.2(14.1,28.3) | 12.1(8.1,17.5) |
| Guangxi | 451227 | Bama Yaozu Zizhixian | 0.4 | 0.2 | 0.6 |  | 14.6(10.0,21.7) | 18.4(11.7,27.7) | 11.7(7.6,17.9) |
| Guangxi | 451226 | Zizhixian | 0.4 | 0.2 | 0.5 |  | 11.1(7.3,16.8) | 14.8(9.0,22.8) | 7.9(5.1,12.0) |
| Guangxi | 451031 | Longlin Gezu Zizhixian | 1.5 | 1.1 | 1.9 |  | 11.0(8.0,14.9) | 13.5(9.8,17.6) | 9.7(6.9,13.3) |
| Guangxi | 451028 | Leye County | 0.4 | 0.2 | 0.5 |  | 10.3(7.3,15.1) | 10.5(7.4,15.4) | 11.4(7.3,19.1) |
| Guangxi | 451025 | Jingxi County | 0.5 | 0.3 | 0.6 |  | 8.9(5.9,13.4) | 11.1(7.6,16.3) | 7.0(4.6,10.7) |
| Heilongjiang | 230502 | Jianshan District | 9.9 | 7.7 | 12.2 |  | 99.6(97.2,100.0) | 99.6(97.0,100.0) | 98.5(87.9,99.9) |
| Heilongjiang | 230306 | Chengzihe District | 8.7 | 7.4 | 9.9 |  | 99.1(88.5,100.0) | 96.0(80.4,99.9) | 99.8(81.2,100.0) |
| Heilongjiang | 230706 | Cuiluan District | 8.5 | 7.2 | 9.8 |  | 98.8(59.0,100.0) | 99.8(64.9,100.0) | 57.0(36.8,75.2) |
| Heilongjiang | 230381 | Hulin City | 8.0 | 6.5 | 9.4 |  | 98.7(93.1,99.8) | 96.9(86.9,99.6) | 99.1(90.7,100.0) |
| Heilongjiang | 230709 | Jinshantun District | 8.9 | 7.1 | 10.5 |  | 97.9(68.6,100.0) | 99.6(73.7,100.0) | 66.9(48.9,80.9) |
| Heilongjiang | 230702 | Yichun District | 8.2 | 7.2 | 9.3 |  | 96.6(74.3,99.9) | 98.9(78.9,100.0) | 79.0(67.6,86.9) |
| Heilongjiang | 232721 | Huma County | 7.4 | 5.5 | 9.1 |  | 96.4(64.1,100.0) | 78.8(65.3,87.6) | 99.7(54.4,100.0) |
| Heilongjiang | 230711 | Wumahe District | 9.2 | 9.1 | 9.3 |  | 96.3(66.8,100.0) | 76.1(61.4,86.4) | 99.8(65.6,100.0) |
| Heilongjiang | 230202 | Longsha District | 8.9 | 7.4 | 10.3 |  | 95.8(82.9,99.6) | 96.1(80.6,99.8) | 92.4(73.3,99.5) |
| Heilongjiang | 232701 | Jiagedaqi District | 8.6 | 6.8 | 10.4 |  | 95.8(82.5,99.7) | 89.2(73.3,98.8) | 98.0(79.4,100.0) |
| Heilongjiang | 230904 | Qiezihe District | 7.0 | 5.3 | 8.7 |  | 95.7(86.0,99.2) | 97.0(87.7,99.7) | 89.6(77.5,97.9) |
| Heilongjiang | 230405 | Xingan District | 11.9 | 10.1 | 13.7 |  | 95.5(88.1,99.2) | 92.6(85.6,96.4) | 96.7(86.8,99.9) |
| Heilongjiang | 230703 | Nancha District | 8.6 | 7.1 | 10.1 |  | 95.5(70.4,99.9) | 80.4(69.6,87.9) | 98.8(59.3,100.0) |
| Heilongjiang | 230705 | Xilin District | 6.7 | 5.9 | 7.5 |  | 95.1(60.3,100.0) | 73.5(60.8,84.7) | 99.5(59.7,100.0) |
| Heilongjiang | 230803 | Xiangyang District | 8.5 | 7.2 | 9.8 |  | 95.1(84.5,99.4) | 95.6(83.9,99.5) | 91.8(76.8,98.7) |
| Heilongjiang | 230713 | Dailing District | 8.0 | 6.9 | 9.1 |  | 94.6(53.2,100.0) | 69.7(53.7,81.9) | 99.3(42.1,100.0) |
| Heilongjiang | 232702 | Songling District | 7.8 | 7.4 | 8.1 |  | 94.5(60.9,100.0) | 65.4(49.1,78.8) | 99.6(65.0,100.0) |
| Heilongjiang | 230522 | Youyi County | 8.5 | 7.1 | 9.9 |  | 94.3(74.6,99.7) | 93.3(74.1,99.8) | 94.4(68.1,99.9) |
| Heilongjiang | 230604 | Ranghulu District | 6.4 | 5.2 | 7.6 |  | 94.2(81.7,99.0) | 92.1(77.8,98.1) | 95.6(82.6,99.6) |
| Heilongjiang | 230902 | Xinxing District | 7.3 | 5.9 | 8.6 |  | 94.1(85.2,98.5) | 92.8(83.3,98.3) | 95.1(82.2,99.4) |
| Heilongjiang | 230521 | Jixian County | 6.7 | 5.8 | 7.7 |  | 93.6(79.1,98.9) | 91.9(74.4,99.1) | 93.8(73.9,99.5) |
| Heilongjiang | 231024 | Dongning County | 8.2 | 6.2 | 10.2 |  | 92.1(76.5,98.7) | 93.4(79.6,99.0) | 84.3(63.2,97.8) |
| Heilongjiang | 230207 | Nianzishan District | 9.5 | 8.6 | 10.5 |  | 91.8(68.3,99.8) | 88.7(63.3,99.5) | 92.1(62.7,99.9) |
| Heilongjiang | 230421 | Luobei County | 7.9 | 6.4 | 9.4 |  | 91.3(79.8,98.1) | 92.0(78.1,98.4) | 86.2(64.2,98.2) |
| Heilongjiang | 230404 | Nanshan District | 9.7 | 8.0 | 11.4 |  | 91.2(80.1,97.2) | 87.6(80.0,92.8) | 92.8(76.9,99.2) |
| Heilongjiang | 230523 | Baoqing County | 6.6 | 5.4 | 7.8 |  | 91.2(81.1,97.0) | 89.5(76.4,96.7) | 91.0(78.0,97.7) |
| Heilongjiang | 230710 | Wuying District | 7.0 | 6.3 | 7.7 |  | 91.0(34.5,100.0) | 97.8(41.4,100.0) | 37.8(22.0,57.9) |
| Heilongjiang | 230833 | Fuyuan County | 6.6 | 6.1 | 7.0 |  | 90.9(77.6,98.1) | 82.7(65.5,95.3) | 96.5(82.2,99.8) |
| Heilongjiang | 231004 | Aimin District | 7.6 | 6.0 | 9.2 |  | 90.6(73.6,98.5) | 95.9(82.1,99.8) | 68.3(53.0,81.9) |
| Heilongjiang | 230704 | Youhao District | 10.3 | 8.7 | 11.9 |  | 90.5(60.1,99.9) | 97.8(65.1,100.0) | 59.3(42.6,75.1) |
| Heilongjiang | 230110 | Xiangfang District | 6.8 | 5.9 | 7.7 |  | 90.1(77.8,96.9) | 85.1(70.4,95.0) | 92.9(77.7,98.5) |
| Heilongjiang | 230304 | Didao District | 8.0 | 6.6 | 9.3 |  | 89.5(68.0,99.6) | 95.7(76.0,100.0) | 72.6(55.1,85.7) |
| Heilongjiang | 230102 | Daoli District | 6.3 | 5.5 | 7.0 |  | 88.9(77.6,96.0) | 89.3(75.7,96.4) | 83.6(66.3,94.5) |
| Heilongjiang | 231081 | Suifenhe City | 3.8 | 3.3 | 4.3 |  | 88.6(68.2,98.4) | 79.4(59.5,97.6) | 95.4(71.9,99.8) |
| Heilongjiang | 230382 | Mishan City | 7.7 | 6.9 | 8.4 |  | 87.8(77.5,94.4) | 83.6(69.4,93.2) | 90.8(79.4,97.2) |
| Heilongjiang | 230208 | Meilisi Daurzu District | 6.9 | 5.4 | 8.3 |  | 87.6(68.9,97.3) | 74.8(61.0,84.5) | 95.6(77.7,99.8) |
| Heilongjiang | 230708 | Meixi District | 7.2 | 6.1 | 8.1 |  | 87.2(36.2,100) | 96.8(39.4,100.0) | 42.0(23.0,62.9) |
| Heilongjiang | 230921 | Boli County | 7.0 | 5.9 | 8.2 |  | 86.9(70.4,96.1) | 87.9(71.9,97.7) | 84.5(67.7,95.6) |
| Heilongjiang | 230302 | Jiguan District | 7.2 | 6.2 | 8.1 |  | 86.5(71.5,96.6) | 79.4(67.0,88.9) | 91.9(75.3,99.4) |
| Heilongjiang | 230603 | Longfeng District | 6.8 | 5.7 | 8.0 |  | 86.5(75.9,94.1) | 86.4(75.0,95.2) | 87.9(76.6,96.4) |
| Heilongjiang | 230203 | Jianhua District | 6.1 | 4.9 | 7.3 |  | 85.9(65.6,97.5) | 79.0(61.1,94.6) | 88.9(63.2,99.3) |
| Heilongjiang | 230307 | Mashan District | 5.5 | 4.2 | 6.6 |  | 84.9(39.7,99.8) | 93.7(48.1,100.0) | 44.6(26.5,65.1) |
| Heilongjiang | 231123 | Xunke County | 6.7 | 5.3 | 8.1 |  | 84.9(68.3,95.9) | 84.6(69.0,97.0) | 85.3(66.4,97.4) |
| Heilongjiang | 230227 | Fuyu County | 5.7 | 4.5 | 6.9 |  | 84.8(71.8,94.1) | 77.8(64.4,89.6) | 91.1(74.7,98.3) |
| Heilongjiang | 230722 | Jiayin County | 6.2 | 5.3 | 7.0 |  | 84.6(58.6,98.0) | 60.4(47.0,73.3) | 95.9(61.6,100) |
| Heilongjiang | 230204 | Tiefeng District | 8.2 | 7.1 | 9.2 |  | 84.4(70.8,94.6) | 88.5(74.7,97.3) | 72.7(61.4,82.1) |
| Heilongjiang | 230602 | Sairt District | 6.0 | 5.1 | 6.9 |  | 84.4(67.5,95.7) | 88.9(71.9,98.3) | 74.3(55.7,90.6) |
| Heilongjiang | 231003 | Yangming District | 6.6 | 5.7 | 7.5 |  | 84.1(68.3,95.4) | 79.3(63.5,93.0) | 86.7(62.6,98.4) |
| Heilongjiang | 230108 | Pingfang District | 8.2 | 7.1 | 9.2 |  | 84.0(62.6,97.7) | 78.0(58.4,96.4) | 86.8(56.4,99.4) |
| Heilongjiang | 231202 | Beilin District | 6.5 | 5.6 | 7.5 |  | 83.6(73.2,90.8) | 82.0(70.6,90.3) | 85.9(75.9,93.4) |
| Heilongjiang | 230103 | Nangang District | 5.4 | 4.6 | 6.2 |  | 83.4(71.8,92.1) | 82.2(69.5,91.7) | 82.3(68.2,92.9) |
| Heilongjiang | 231002 | Dong'an District | 6.9 | 5.7 | 8.2 |  | 83.1(63.1,96.4) | 80.6(63.6,95.3) | 83.4(56.6,98.8) |
| Heilongjiang | 230403 | Gongnong District | 6.8 | 5.4 | 8.3 |  | 82.6(66.2,95.4) | 88.6(71.3,98.3) | 71.9(58.7,82.2) |
| Heilongjiang | 230881 | Tongjiang City | 6.3 | 4.9 | 7.6 |  | 82.6(68.4,92.3) | 83.6(70.6,93.9) | 81.0(65.9,93.8) |
| Heilongjiang | 230303 | Hengshan District | 8.2 | 6.8 | 9.6 |  | 81.8(64.8,96.3) | 78.8(67.9,87.3) | 82.6(55.4,99.2) |
| Heilongjiang | 230223 | Yi'an County | 5.9 | 4.7 | 7.2 |  | 81.2(68.7,90.3) | 85.9(71.5,94.3) | 73.5(61.4,84.3) |
| Heilongjiang | 230781 | Tieli City | 6.9 | 5.9 | 8.1 |  | 80.8(64.6,93.1) | 80.0(60.6,93.0) | 78.8(53.6,94.8) |
| Heilongjiang | 230321 | Jidong County | 6.3 | 4.8 | 7.8 |  | 80.7(68.0,91.7) | 85.5(73.3,95.1) | 74.4(62.8,83.9) |
| Heilongjiang | 231182 | Wudalianchi City | 6.7 | 5.4 | 7.9 |  | 80.2(67.6,90.9) | 81.0(66.8,92.9) | 77.9(63.1,91.0) |
| Heilongjiang | 230903 | Taoshan District | 5.5 | 4.5 | 6.6 |  | 80.0(66.2,91.0) | 80.7(68.1,91.8) | 82.2(67.9,93.4) |
| Heilongjiang | 230230 | Kedong County | 5.9 | 4.5 | 7.3 |  | 79.7(66.8,89.7) | 85.5(71.0,95.4) | 71.6(61.1,80.5) |
| Heilongjiang | 231102 | Aihui District | 6.9 | 6.0 | 7.9 |  | 79.7(66.5,90.4) | 77.9(64.6,89.8) | 81.0(66.0,93.6) |
| Heilongjiang | 230221 | Longjiang County | 6.0 | 4.9 | 7.1 |  | 79.6(68.1,88.9) | 76.2(64.8,85.8) | 84.2(72.8,93.3) |
| Heilongjiang | 232723 | Mohe County | 7.5 | 6.3 | 8.5 |  | 79.5(69.4,87.1) | 79.3(67.7,87.8) | 80.7(69.6,88.9) |
| Heilongjiang | 231283 | Hailun City | 6.4 | 5.0 | 7.8 |  | 79.2(68.9,87.2) | 79.0(68.6,86.9) | 79.4(66.6,88.9) |
| Heilongjiang | 230882 | Fujin City | 5.6 | 4.5 | 6.7 |  | 78.7(66.0,88.3) | 78.1(65.5,87.9) | 79.7(66.4,90.6) |
| Heilongjiang | 230811 | Dongfeng District | 6.2 | 5.2 | 7.2 |  | 78.6(62.0,92.0) | 83.4(65.7,95.9) | 70.5(54.2,82.3) |
| Heilongjiang | 231084 | Ning'an City | 6.6 | 5.2 | 8.0 |  | 78.6(61.9,90.7) | 79.5(64.6,90.9) | 76.4(56.9,92.1) |
| Heilongjiang | 230622 | Zhaoyuan County | 6.2 | 5.0 | 7.4 |  | 77.8(67.2,87.1) | 78.5(68.4,87.0) | 79.6(67.6,89.3) |
| Heilongjiang | 230225 | Gannan County | 5.8 | 4.6 | 7.1 |  | 77.6(66.3,86.3) | 78.2(67.4,87.9) | 78.9(66.0,89.4) |
| Heilongjiang | 230826 | Huachuan County | 6.8 | 5.4 | 8.1 |  | 77.4(62.6,89.7) | 78.9(64.3,92.3) | 75.1(62.0,86.1) |
| Heilongjiang | 230822 | Huanan County | 6.2 | 5.0 | 7.3 |  | 76.9(65.8,86.5) | 76.5(64.5,86.1) | 78.3(64.9,89.3) |
| Heilongjiang | 230112 | Acheng District | 6.1 | 4.9 | 7.3 |  | 76.3(65.5,84.8) | 75.6(65.2,84.2) | 79.5(67.0,89.3) |
| Heilongjiang | 230422 | Suibin County | 7.2 | 5.8 | 8.6 |  | 76.3(62.4,88.2) | 79.1(63.3,91.1) | 72.4(56.2,84.3) |
| Heilongjiang | 230224 | Tailai County | 5.0 | 3.8 | 6.3 |  | 75.9(62.0,87.3) | 82.1(68.1,93.6) | 65.5(51.1,81.0) |
| Heilongjiang | 230104 | Daowai District | 6.7 | 5.6 | 7.8 |  | 75.8(57.7,90.1) | 84.0(67.2,94.7) | 54.3(36.1,77.8) |
| Heilongjiang | 230524 | Raohe County | 6.0 | 4.8 | 7.2 |  | 75.8(58.5,89.8) | 82.5(62.5,95.7) | 64.7(50.7,75.9) |
| Heilongjiang | 230828 | Tangyuan County | 6.0 | 5.2 | 6.7 |  | 74.3(58.9,87.6) | 78.4(60.2,93.5) | 67.5(54.6,78.7) |
| Heilongjiang | 230805 | Dongfeng District | 6.6 | 5.2 | 7.9 |  | 74.2(53.7,92.3) | 79.8(58.9,96.4) | 63.8(46.4,78.8) |
| Heilongjiang | 231005 | Xi'an District | 7.0 | 6.0 | 8.2 |  | 74.2(56.7,89.6) | 76.4(59.9,91.6) | 70.2(50.7,90.7) |
| Heilongjiang | 230205 | Ang'angxi District | 7.6 | 6.0 | 9.1 |  | 73.9(60.3,85.1) | 77.2(63.2,86.7) | 68.9(51.9,81.5) |
| Heilongjiang | 231124 | Sunwu County | 6.0 | 5.0 | 7.0 |  | 73.9(58.6,88.1) | 68.1(56.5,77.7) | 81.7(63.0,96.2) |
| Heilongjiang | 230128 | Tonghe County | 6.4 | 5.4 | 7.4 |  | 73.6(60.4,84.8) | 63.5(51.6,73.9) | 84.2(67.4,95.1) |
| Heilongjiang | 230623 | Lindian County | 5.7 | 4.2 | 7.1 |  | 72.8(60.3,83.2) | 72.0(60.8,82.4) | 75.4(60.9,88.6) |
| Heilongjiang | 230804 | Qianjin District | 6.6 | 5.5 | 7.6 |  | 72.2(49.7,91.6) | 79.1(55.5,97.1) | 58.6(39.5,74.6) |
| Heilongjiang | 232703 | Xinlin District | 7.2 | 5.5 | 8.8 |  | 71.5(58.4,81.7) | 76.3(62.7,85.9) | 66.7(51.2,80.9) |
| Heilongjiang | 230123 | Yilan County | 6.4 | 5.1 | 7.7 |  | 71.1(58.6,81.4) | 70.3(58.1,80.5) | 72.0(58.2,84.1) |
| Heilongjiang | 230231 | Baiquan County | 4.6 | 3.7 | 5.6 |  | 71.0(57.8,82.8) | 72.3(58.7,84.3) | 69.1(56.2,81.6) |
| Heilongjiang | 230624 | Zizhixian | 5.9 | 4.4 | 7.3 |  | 71.0(58.8,82.0) | 74.5(61.5,85.5) | 66.4(52.3,80.8) |
| Heilongjiang | 230606 | Datong District | 5.6 | 4.7 | 6.5 |  | 70.1(53.4,85.7) | 65.1(49.5,78.0) | 78.7(58.3,94.1) |
| Heilongjiang | 231121 | Nenjiang County | 5.9 | 4.6 | 7.2 |  | 69.2(56.6,81.2) | 69.4(56.3,81.7) | 70.5(54.5,84.2) |
| Heilongjiang | 230111 | Hulan District | 3.9 | 3.0 | 4.8 |  | 69.1(57.8,79.6) | 69.8(58.1,80.6) | 74.2(63.4,84.4) |
| Heilongjiang | 230402 | Xiangyang District | 6.9 | 5.9 | 7.9 |  | 68.7(51.0,88.9) | 76.7(54.8,94.9) | 57.1(42.0,71.7) |
| Heilongjiang | 230407 | Xingshan District | 7.9 | 6.8 | 9.1 |  | 68.0(50.5,81.4) | 73.2(56.0,84.9) | 60.3(39.1,76.5) |
| Heilongjiang | 231281 | Anda City | 6.2 | 4.9 | 7.4 |  | 67.5(55.5,78.7) | 68.5(56.7,79.1) | 67.7(55.0,80.7) |
| Heilongjiang | 230406 | Dongshan District | 6.1 | 5.3 | 6.9 |  | 67.4(42.8,91.0) | 66.4(43.8,92.2) | 64.5(41.9,91.9) |
| Heilongjiang | 230182 | Shuangcheng City | 5.5 | 4.5 | 6.3 |  | 67.3(51.8,81.9) | 69.5(52.2,83.3) | 65.8(51.5,78.2) |
| Heilongjiang | 231224 | Qing'an County | 5.3 | 4.2 | 6.4 |  | 67.1(55.0,78.1) | 66.1(53.2,77.4) | 71.4(57.2,84.0) |
| Heilongjiang | 230281 | Nehe City | 4.4 | 3.5 | 5.3 |  | 66.8(49.1,82.8) | 66.7(48.8,84.5) | 68.8(50.4,85.3) |
| Heilongjiang | 230714 | Wuyiling District | 8.4 | 6.5 | 10.3 |  | 66.7(48.9,80.3) | 74.3(59.5,85.5) | 52.8(31.6,73.6) |
| Heilongjiang | 230109 | Songbei District | 4.6 | 3.9 | 5.3 |  | 66.4(46.8,86.8) | 69.0(46.9,88.9) | 64.2(45.4,82.4) |
| Heilongjiang | 231282 | Zhaodong City | 5.3 | 4.2 | 6.4 |  | 66.4(51.4,80.2) | 67.4(52.6,79.8) | 68.1(51.9,82.9) |
| Heilongjiang | 230124 | Fangzheng County | 6.3 | 4.8 | 7.7 |  | 66.3(50.8,80.8) | 67.2(52.2,79.9) | 66.8(49.8,85.7) |
| Heilongjiang | 231223 | Qinggang County | 5.4 | 4.5 | 6.2 |  | 66.3(51.4,77.7) | 63.6(50.2,76.8) | 72.1(56.6,86.1) |
| Heilongjiang | 231025 | Linkou County | 4.9 | 3.9 | 5.9 |  | 66.1(45.3,85.7) | 58.4(41.5,77.6) | 77.1(53.0,95.4) |
| Heilongjiang | 230129 | Yanshou County | 5.5 | 4.5 | 6.5 |  | 65.9(54.0,76.0) | 66.5(52.8,79.4) | 68.3(56.6,78.9) |
| Heilongjiang | 230712 | Tangwanghe District | 7.9 | 7.2 | 8.6 |  | 65.9(51.2,79.2) | 69.2(53.2,82.2) | 62.5(42.3,78.8) |
| Heilongjiang | 231222 | Lanxi County | 5.7 | 4.5 | 6.9 |  | 65.9(50.7,79.1) | 68.3(54.5,81.7) | 65.6(50.1,80.5) |
| Heilongjiang | 230605 | Honggang District | 6.3 | 5.1 | 7.4 |  | 65.7(51.3,78.5) | 68.6(54.7,80.8) | 65.1(48.8,79.1) |
| Heilongjiang | 230229 | Keshan County | 4.5 | 3.4 | 5.7 |  | 65.6(48.7,80.7) | 67.3(51.2,83.3) | 64.2(45.1,83.1) |
| Heilongjiang | 230707 | Xinqing District | 7.7 | 6.7 | 8.7 |  | 65.3(50.7,76.8) | 70.8(55.4,82.4) | 57.1(40.0,72.7) |
| Heilongjiang | 231221 | Wangkui County | 5.3 | 3.9 | 6.7 |  | 65.0(53.2,76.5) | 65.3(54.6,76.2) | 66.7(50.2,82.9) |
| Heilongjiang | 230127 | Mulan County | 5.2 | 3.9 | 6.4 |  | 64.6(52.5,77.3) | 69.1(54.5,82.3) | 61.6(48.8,75.2) |
| Heilongjiang | 230183 | Shangzhi City | 5.8 | 4.7 | 6.8 |  | 63.1(46.3,77.2) | 64.5(49.1,78.2) | 63.9(48.6,78.1) |
| Heilongjiang | 231226 | Suileng County | 6.0 | 5.3 | 6.7 |  | 63.0(50.1,75.3) | 56.9(46.5,67.3) | 73.4(57.5,87.9) |
| Heilongjiang | 232722 | Tahe County | 7.2 | 6.5 | 8.0 |  | 62.1(49.0,74.8) | 61.5(47.6,74.1) | 65.2(51.0,78.5) |
| Heilongjiang | 230206 | Hulan Ergi District | 8.5 | 7.5 | 9.6 |  | 61.9(41.4,81.8) | 60.5(42.4,79.4) | 59.4(35.8,84.6) |
| Heilongjiang | 230505 | Sifangtai District | 4.5 | 4.0 | 5.0 |  | 61.9(44.9,76.7) | 61.2(43.2,76.2) | 69.1(51.4,83.6) |
| Heilongjiang | 230305 | Lishu District | 7.2 | 5.9 | 8.5 |  | 59.6(42.2,75.2) | 68.9(52.4,81.3) | 46.2(27.8,64.7) |
| Heilongjiang | 231083 | Hailin City | 6.8 | 5.6 | 8.0 |  | 59.5(46.5,71.2) | 64.9(52.1,77.4) | 52.0(39.7,65.4) |
| Heilongjiang | 231085 | Muleng City | 5.9 | 4.8 | 6.8 |  | 58.0(44.8,71.0) | 57.6(45.3,68.5) | 60.9(45.4,78.1) |
| Heilongjiang | 230503 | Lingdong District | 7.1 | 6.3 | 7.8 |  | 57.8(41.9,71.5) | 63.8(48.2,76.1) | 48.0(30.6,65.7) |
| Heilongjiang | 230506 | Baoshan District | 5.4 | 4.6 | 6.2 |  | 56.5(37.0,84.3) | 67.9(42.6,95.0) | 40.9(28.6,56.3) |
| Heilongjiang | 231181 | Bei'an City | 5.3 | 4.4 | 6.3 |  | 56.5(43.7,68.6) | 57.6(44.9,70.7) | 56.0(41.2,73.1) |
| Heilongjiang | 230716 | Shangganling District | 8.3 | 6.4 | 10.4 |  | 55.3(38.0,71.3) | 71.5(54.2,84.4) | 30.1(13.0,50.9) |
| Heilongjiang | 232704 | Huzhong District | 5.6 | 4.3 | 6.9 |  | 55.1(40.4,70.0) | 60.4(44.9,75.7) | 50.9(33.3,67.8) |
| Heilongjiang | 231225 | Mingshui County | 3.9 | 2.9 | 5.0 |  | 52.2(38.0,66.1) | 56.0(42.2,73.5) | 50.1(36.0,65.9) |
| Heilongjiang | 230184 | Wuchang City | 4.6 | 3.6 | 5.5 |  | 49.8(38.4,62.3) | 50.6(38.4,63.1) | 52.0(38.9,66.7) |
| Heilongjiang | 230126 | Bayan County | 4.4 | 3.6 | 5.1 |  | 49.5(35.4,65.8) | 50.0(35.2,63.9) | 52.1(37.0,69.7) |
| Heilongjiang | 230125 | Bin County | 3.9 | 3.0 | 4.8 |  | 46.4(34.4,58.9) | 50.4(38.2,64.7) | 45.5(33.8,58.4) |
| Heilongjiang | 230715 | Hongxing District | 7.1 | 5.7 | 8.5 |  | 45.1(28.4,64.1) | 54.0(35.0,71.0) | 32.3(16.1,53.2) |
| Heilongjiang | 230621 | Zhaozhou County | 2.2 | 1.8 | 2.7 |  | 28.9(20.7,38.9) | 30.3(21.3,42.2) | 31.6(22.7,41.5) |
| Anhui | 340402 | Datong District | 5.9 | 4.4 | 8.0 |  | 94.4(85.9,98.6) | 97.1(89.9,99.5) | 87.0(70.9,96.4) |
| Anhui | 340208 | Samshan District | 7.8 | 7.2 | 8.5 |  | 93.8(84.3,98.0) | 96.3(85.6,99.5) | 85.5(74.5,94.0) |
| Anhui | 340504 | Yushan District | 5.3 | 4.6 | 5.9 |  | 93.1(82.5,98.4) | 92.4(77.1,98.5) | 93.1(77.1,99.0) |
| Anhui | 340122 | Feidong County | 7.0 | 6.1 | 8.0 |  | 93.0(87.6,96.5) | 91.3(84.9,95.8) | 93.5(87.1,97.4) |
| Anhui | 341002 | Tunxi District | 5.9 | 4.9 | 6.8 |  | 91.3(80.9,97.1) | 88.3(75.5,96.0) | 92.9(78.3,98.4) |
| Anhui | 341823 | Jing County | 8.2 | 6.6 | 9.7 |  | 91.1(83.3,96.2) | 90.5(81.4,96.4) | 89.1(78.0,95.9) |
| Anhui | 340406 | Panji District | 6.6 | 5.0 | 8.3 |  | 90.6(84.0,95.3) | 91.5(84.7,96.0) | 88.1(79.5,94.3) |
| Anhui | 341503 | Yu'an District | 6.6 | 5.9 | 7.2 |  | 89.7(83.9,93.8) | 86.6(79.6,91.4) | 92.8(87.6,96.5) |
| Anhui | 340503 | Huashan District | 5.1 | 4.3 | 5.8 |  | 89.6(78.7,96.3) | 89.3(76.2,97.0) | 88.4(71.8,97.1) |
| Anhui | 340123 | Feixi County | 6.2 | 5.0 | 7.3 |  | 89.5(82.8,94.5) | 90.2(82.6,95.3) | 87.0(78.2,93.2) |
| Anhui | 340506 | Bowang District | 6.5 | 5.9 | 7.0 |  | 89.3(79.0,95.4) | 86.3(73.2,94.8) | 91.0(77.3,97.9) |
| Anhui | 340124 | Lujiang County | 7.4 | 6.2 | 8.7 |  | 87.5(80.9,92.4) | 87.5(80.5,92.2) | 85.9(77.9,91.5) |
| Anhui | 341103 | Nanqiao District | 6.6 | 5.7 | 7.4 |  | 87.5(77.7,94.4) | 89.8(77.9,96.7) | 82.4(70.9,91.4) |
| Anhui | 341521 | Shou County | 6.9 | 5.9 | 7.8 |  | 87.1(81.1,91.8) | 86.2(79.1,91.2) | 88.0(81.0,93.1) |
| Anhui | 341226 | Yingshang County | 7.2 | 5.8 | 8.6 |  | 86.8(80.1,91.3) | 86.9(80.5,91.6) | 86.8(79.8,91.4) |
| Anhui | 340207 | Jiujiang District | 5.5 | 4.9 | 6.2 |  | 86.2(76.8,93.3) | 89.6(78.6,95.9) | 78.8(65.8,89.7) |
| Anhui | 340811 | Yixiu District | 4.3 | 3.9 | 4.7 |  | 85.6(72.4,95.2) | 74.8(57.1,89.4) | 93.2(79.5,98.6) |
| Anhui | 341003 | Huangshan District | 7.3 | 6.3 | 8.2 |  | 85.6(73.8,94.3) | 91.2(75.8,98.4) | 78.2(68.6,86.3) |
| Anhui | 340827 | Wangjiang County | 6.6 | 5.8 | 7.5 |  | 85.5(77.8,91.6) | 84.9(75.4,91.4) | 85.5(76.5,92.2) |
| Anhui | 340104 | Shushan District | 2.7 | 2.4 | 3.0 |  | 85.1(63.6,96.7) | 85.5(66.5,97.2) | 83.1(64.1,95.9) |
| Anhui | 341323 | Lingbi County | 6.5 | 5.7 | 7.4 |  | 83.6(76.3,89.2) | 85.2(78.0,90.5) | 80.9(73.3,87.5) |
| Anhui | 340121 | Changfeng County | 6.0 | 4.9 | 7.2 |  | 83.4(74.3,90.0) | 83.1(73.7,90.6) | 81.4(71.1,90.0) |
| Anhui | 341502 | Jin'an District | 5.7 | 4.8 | 6.5 |  | 83.2(75.3,89.1) | 82.8(74.0,89.7) | 83.5(74.2,90.1) |
| Anhui | 341881 | Ningguo City | 6.9 | 6.0 | 7.8 |  | 82.7(72.7,89.9) | 85.9(74.8,93.8) | 78.6(66.9,87.9) |
| Anhui | 341181 | Tianchang City | 6.4 | 5.6 | 7.2 |  | 82.6(73.7,89.3) | 85.1(75.4,92.4) | 79.5(69.3,87.5) |
| Anhui | 341302 | Yongqiao District | 5.9 | 5.1 | 6.7 |  | 82.1(65.9,93.3) | 84.0(68.3,94.0) | 79.2(61.5,90.4) |
| Anhui | 340881 | Tongcheng City | 6.5 | 5.7 | 7.4 |  | 82.0(73.5,88.8) | 81.7(72.5,89.4) | 83.4(74.4,90.8) |
| Anhui | 341523 | Shucheng County | 7.3 | 6.0 | 8.6 |  | 82.0(72.5,88.2) | 84.1(74.3,90.4) | 77.1(67.1,85.2) |
| Anhui | 341182 | Mingguang City | 6.5 | 5.6 | 7.5 |  | 81.9(72.4,88.4) | 79.4(70.2,86.7) | 85.0(75.2,92.1) |
| Anhui | 341824 | Jixi County | 7.7 | 6.8 | 8.7 |  | 81.6(68.9,91.1) | 86.1(71.8,95.7) | 72.6(62.3,81.9) |
| Anhui | 340621 | Suixi County | 6.2 | 5.4 | 6.9 |  | 81.5(73.8,87.6) | 81.9(73.2,88.0) | 79.8(70.5,86.4) |
| Anhui | 341802 | Xuanzhou District | 6.0 | 5.2 | 6.9 |  | 81.5(72.9,88.6) | 78.6(68.3,86.5) | 84.1(74.1,91.6) |
| Anhui | 341102 | Langya District | 5.3 | 4.6 | 5.9 |  | 81.3(70.0,90.0) | 79.4(66.9,89.6) | 83.2(70.2,93.1) |
| Anhui | 340523 | He County | 7.3 | 6.9 | 7.7 |  | 81.1(71.3,88.5) | 78.5(66.7,87.6) | 81.5(70.2,90.4) |
| Anhui | 340721 | Tongling County | 7.0 | 5.9 | 8.1 |  | 81.1(72.1,89.2) | 81.2(70.8,89.4) | 81.6(70.0,92.1) |
| Anhui | 341524 | Jinzhai County | 6.8 | 6.4 | 7.0 |  | 80.7(72.4,87.5) | 77.4(67.3,85.8) | 83.1(74.5,90.1) |
| Anhui | 341821 | Langxi County | 6.2 | 4.9 | 7.5 |  | 80.6(69.0,88.9) | 87.4(76.6,94.5) | 69.9(58.3,79.6) |
| Anhui | 340202 | Jinghu District | 6.0 | 5.4 | 6.7 |  | 79.5(69.0,87.8) | 76.4(64.9,85.6) | 81.7(70.3,90.8) |
| Anhui | 340705 | Shizishan District | 6.0 | 4.8 | 7.3 |  | 79.5(69.4,87.5) | 83.5(73.7,91.8) | 73.2(60.8,83.2) |
| Anhui | 340103 | Luyang District | 3.5 | 3.0 | 4.1 |  | 79.1(58.2,93.8) | 78.8(58.9,94.4) | 79.0(56.4,93.5) |
| Anhui | 340181 | Juchao District | 6.8 | 6.0 | 7.7 |  | 79.0(67.8,88.1) | 78.8(68.0,87.8) | 75.5(63.1,84.7) |
| Anhui | 341825 | Jingde County | 8.3 | 7.5 | 9.1 |  | 79.0(62.1,92.4) | 77.1(58.8,92.3) | 79.5(61.4,92.6) |
| Anhui | 341622 | Mengcheng County | 5.9 | 4.9 | 6.9 |  | 78.9(70.2,85.4) | 80.3(72.2,86.6) | 77.5(68.3,85.0) |
| Anhui | 340223 | Nanling County | 7.8 | 6.5 | 9.0 |  | 78.8(70.0,86.1) | 78.8(70.1,86.6) | 77.4(65.7,87.0) |
| Anhui | 341126 | Fengyang County | 6.4 | 5.8 | 6.9 |  | 78.4(68.3,85.7) | 77.4(67.3,84.6) | 80.0(70.9,87.5) |
| Anhui | 340323 | Guzhen County | 7.0 | 5.8 | 8.2 |  | 78.2(68.9,85.5) | 79.4(70.2,86.3) | 76.7(66.8,84.3) |
| Anhui | 341203 | Yingdong District | 6.5 | 5.2 | 7.6 |  | 78.1(68.5,85.2) | 78.0(68.3,85.5) | 77.4(66.8,86.3) |
| Anhui | 340102 | Yaohai District | 3.4 | 2.9 | 3.9 |  | 78.0(58.8,91.9) | 77.8(59.5,92.4) | 77.0(58.7,91.0) |
| Anhui | 341222 | Taihe County | 6.8 | 6.0 | 7.7 |  | 77.5(68.9,83.7) | 78.2(70.0,85.2) | 77.6(69.2,84.5) |
| Anhui | 340802 | Yingjiang District | 5.0 | 4.4 | 5.6 |  | 77.4(60.9,90.3) | 77.1(57.8,92.8) | 76.0(56.2,92.2) |
| Anhui | 341124 | Quanjiao County | 6.9 | 6.2 | 7.5 |  | 77.4(66.8,86.6) | 75.3(65.1,84.9) | 79.8(67.6,88.9) |
| Anhui | 341722 | Shitai County | 7.7 | 6.1 | 9.3 |  | 77.2(64.0,88.2) | 85.1(68.4,94.6) | 66.5(54.3,76.3) |
| Anhui | 341324 | Si County | 6.6 | 5.8 | 7.4 |  | 77.1(68.3,83.7) | 76.9(68.7,84.6) | 77.7(68.7,84.8) |
| Anhui | 341282 | Jieshou City | 5.9 | 5.1 | 6.8 |  | 76.9(67.8,85.2) | 80.3(71.1,87.5) | 71.5(60.9,79.8) |
| Anhui | 340222 | Fangchang County | 6.5 | 5.7 | 7.2 |  | 76.8(64.5,86.9) | 77.3(63.5,89.0) | 74.6(59.6,87.3) |
| Anhui | 340803 | Daguan District | 5.1 | 4.4 | 5.8 |  | 76.5(61.0,88.1) | 80.5(63.1,93.6) | 68.7(55.2,84.5) |
| Anhui | 341204 | Yingquan District | 6.7 | 5.8 | 7.5 |  | 76.5(67.8,83.8) | 78.5(69.7,85.4) | 74.2(65.0,82.7) |
| Anhui | 340311 | Huaishang District | 6.3 | 6.3 | 6.3 |  | 76.2(64.5,85.1) | 71.6(58.2,82.9) | 80.9(70.0,90.0) |
| Anhui | 340825 | Taihu County | 6.8 | 6.0 | 7.5 |  | 76.1(65.7,83.2) | 74.8(64.6,83.1) | 77.8(67.4,86.0) |
| Anhui | 340403 | Tianjia'an District | 3.8 | 3.0 | 4.5 |  | 75.7(62.6,86.8) | 76.9(63.3,88.2) | 70.4(55.8,84.2) |
| Anhui | 341023 | Yi County | 7.5 | 6.3 | 8.7 |  | 75.7(61.1,88.0) | 71.4(59.6,80.6) | 79.4(61.6,94.9) |
| Anhui | 341221 | Linquan County | 6.3 | 5.3 | 7.4 |  | 75.5(62.4,85.4) | 79.1(67.2,87.8) | 73.4(58.9,84.6) |
| Anhui | 340521 | Dangtu County | 6.8 | 6.0 | 7.5 |  | 74.8(64.3,83.6) | 73.8(61.8,83.5) | 76.5(65.3,85.8) |
| Anhui | 341202 | Yingzhou District | 6.0 | 5.2 | 6.6 |  | 74.7(65.3,82.1) | 74.8(65.8,82.2) | 75.6(66.1,82.8) |
| Anhui | 340822 | Huaining County | 6.5 | 5.9 | 7.0 |  | 74.5(64.0,82.9) | 71.7(61.6,81.0) | 76.9(65.8,85.4) |
| Anhui | 340221 | Wuhu County | 6.9 | 6.1 | 7.6 |  | 74.4(57.5,87.3) | 76.1(56.9,90.4) | 71.1(53.5,86.1) |
| Anhui | 340302 | Longzihu District | 6.8 | 6.5 | 7.1 |  | 74.1(62.8,83.9) | 68.5(55.9,80.6) | 79.8(66.6,90.2) |
| Anhui | 341723 | Qingyang County | 6.5 | 5.7 | 7.3 |  | 73.4(63.5,82.1) | 74.8(63.0,84.7) | 74.6(64.1,83.1) |
| Anhui | 341004 | Huizhou District | 6.1 | 5.8 | 6.3 |  | 73.3(63.4,81.1) | 70.5(59.0,80.0) | 78.8(68.7,86.6) |
| Anhui | 341721 | Dongzhi County | 6.3 | 5.4 | 7.3 |  | 72.7(63.1,80.8) | 76.3(66.4,84.1) | 72.1(62.5,81.1) |
| Anhui | 340828 | Yuexi County | 6.2 | 5.3 | 7.1 |  | 72.6(62.2,81.2) | 72.9(61.7,82.5) | 73.2(61.2,83.0) |
| Anhui | 341022 | Xiuning County | 6.0 | 5.5 | 6.7 |  | 72.6(59.8,82.2) | 66.2(54.5,76.4) | 79.0(63.7,90.3) |
| Anhui | 341225 | Funan County | 6.2 | 5.2 | 7.3 |  | 72.1(63.5,80.8) | 76.1(67.5,83.5) | 70.5(60.9,78.9) |
| Anhui | 341125 | Dingyuan County | 6.2 | 5.4 | 7.0 |  | 71.4(61.9,79.3) | 71.5(62.1,80.4) | 71.3(60.6,80.7) |
| Anhui | 340111 | Baohe District | 2.7 | 2.4 | 3.0 |  | 71.3(52.1,89.8) | 71.7(49.4,89.7) | 72.4(49.6,90.4) |
| Anhui | 340303 | Bangshan District | 4.8 | 4.4 | 5.2 |  | 71.1(58.7,82.0) | 72.4(57.7,86.1) | 67.4(54.7,80.0) |
| Anhui | 341024 | Qimen County | 6.3 | 5.7 | 7.0 |  | 71.1(58.6,81.8) | 76.1(61.5,88.7) | 67.1(55.9,76.7) |
| Anhui | 340322 | Wuhe County | 6.4 | 5.1 | 7.8 |  | 70.7(61.0,79.7) | 78.1(68.7,85.6) | 62.6(52.0,73.2) |
| Anhui | 341623 | Lixin County | 5.8 | 4.5 | 7.1 |  | 70.7(61.1,79.0) | 76.2(66.7,83.6) | 64.8(53.8,74.9) |
| Anhui | 341702 | Chizhou City | 4.7 | 4.1 | 5.3 |  | 70.1(59.3,79.2) | 73.9(62.3,83.9) | 67.4(56.5,77.3) |
| Anhui | 340225 | Wuwei County | 6.3 | 5.6 | 7.0 |  | 69.9(59.7,78.6) | 69.6(58.7,78.8) | 70.7(60.2,80.0) |
| Anhui | 340522 | Hanshan County | 6.9 | 6.2 | 7.6 |  | 69.5(52.7,84.1) | 63.2(47.7,77.8) | 78.0(59.3,91.1) |
| Anhui | 341122 | Lai'an County | 6.0 | 5.3 | 6.9 |  | 69.3(58.6,79.5) | 69.7(57.7,79.0) | 69.4(57.7,80.4) |
| Anhui | 341021 | She County | 6.9 | 5.8 | 7.9 |  | 69.1(58.8,78.0) | 72.5(61.2,80.8) | 65.4(52.7,76.8) |
| Anhui | 341321 | Dangshan County | 6.1 | 5.5 | 6.8 |  | 68.3(59.3,77.3) | 69.8(59.5,77.7) | 70.0(59.7,78.7) |
| Anhui | 341322 | Xiao County | 5.9 | 5.2 | 6.6 |  | 68.1(57.9,76.5) | 68.9(60.5,77.7) | 69.1(59.5,78.1) |
| Anhui | 340421 | Fengtai County | 5.5 | 4.7 | 6.3 |  | 67.6(56.7,76.9) | 69.6(58.7,79.3) | 61.3(50.6,72.9) |
| Anhui | 341525 | Huoshan County | 5.8 | 5.4 | 6.0 |  | 66.8(54.9,77.8) | 65.4(51.0,77.2) | 69.0(56.3,79.5) |
| Anhui | 341822 | Guangde County | 5.3 | 4.3 | 6.3 |  | 65.1(54.0,76.1) | 65.4(53.9,75.8) | 67.0(54.3,78.3) |
| Anhui | 341522 | Huoqiu County | 5.3 | 3.9 | 7.0 |  | 63.0(53.2,72.2) | 70.5(61.9,78.1) | 57.3(47.0,67.2) |
| Anhui | 340203 | Gejiang District | 4.2 | 3.7 | 4.7 |  | 61.8(47.3,77.5) | 59.1(43.0,75.8) | 64.0(45.0,82.5) |
| Anhui | 340321 | Huaiyuan County | 5.6 | 4.5 | 6.8 |  | 60.4(49.9,69.9) | 65.2(55.0,74.2) | 57.8(47.8,67.6) |
| Anhui | 340824 | Qianshan County | 4.7 | 4.1 | 5.3 |  | 60.2(47.7,71.8) | 58.3(46.9,69.9) | 64.9(51.9,78.2) |
| Anhui | 340604 | Lieshan District | 3.3 | 2.6 | 3.8 |  | 59.5(45.6,74.3) | 59.6(45.3,76.4) | 59.7(41.5,79.7) |
| Anhui | 340826 | Susong County | 5.8 | 5.1 | 6.4 |  | 56.8(46.2,66.3) | 57.6(46.9,67.8) | 56.3(45.0,67.4) |
| Anhui | 340404 | Xiejiaji District | 5.3 | 4.4 | 6.2 |  | 55.6(45.2,66.4) | 55.2(44.9,64.7) | 58.6(47.4,69.7) |
| Anhui | 340603 | Xiangshan District | 2.3 | 1.9 | 2.6 |  | 55.2(42.0,71.0) | 55.2(40.7,71.9) | 58.0(42.5,75.5) |
| Anhui | 340711 | Jiao District | 3.5 | 2.9 | 4.1 |  | 53.4(39.6,69.3) | 54.5(39.8,70.5) | 54.9(37.5,75.1) |
| Anhui | 340602 | Duji District | 3.4 | 2.9 | 4.0 |  | 52.0(40.0,63.7) | 56.9(44.3,71.4) | 49.5(39.4,60.4) |
| Anhui | 340304 | Yuhui District | 3.9 | 3.8 | 4.1 |  | 48.4(36.5,61.0) | 46.8(34.0,62.0) | 49.7(38.0,62.8) |
| Anhui | 341602 | Qiaocheng District | 2.9 | 2.6 | 3.1 |  | 43.8(35.0,53.8) | 45.7(36.0,55.7) | 46.5(36.2,56.2) |
| Anhui | 340823 | Zongyang County | 2.7 | 2.4 | 3.0 |  | 40.6(29.5,51.2) | 41.3(31.1,53.9) | 43.1(32.7,56.7) |
| Anhui | 341621 | Guoyang County | 2.3 | 1.8 | 2.8 |  | 35.8(27.5,45.2) | 39.8(30.8,50.2) | 35.3(26.4,44.6) |
| Anhui | 340405 | Bagongshan Qi | 1.7 | 0.9 | 2.6 |  | 18.7(12.6,28.3) | 30.2(19.3,51.1) | 12.4(8.2,17.8) |
| Sichuan | 510822 | Qingchuan County | 8.3 | 6.7 | 9.9 |  | 86.0.(76.9,92.7) | 88.4(76.6,95.5) | 80.8(70.7,88.8) |
| Sichuan | 510122 | Shuangliu County | 8.2 | 6.8 | 9.5 |  | 99.5(98.4,99.9) | 99.4(97.8,99.9) | 99.4(98.0,99.9) |
| Sichuan | 510108 | Chenghua District | 5.0 | 4.1 | 5.8 |  | 97.7(92.5,99.6) | 94.3(84.7,98.7) | 99.0(93.5,99.9) |
| Sichuan | 510703 | Fucheng District | 5.3 | 4.3 | 6.3 |  | 97.7(93.2,99.5) | 97.6(91.5,99.6) | 97.2(89.6,99.5) |
| Sichuan | 510114 | Xindu District | 6.8 | 5.7 | 8.0 |  | 97.5(94.1,99.3) | 96.2(90.9,98.9) | 97.8(93.2,99.4) |
| Sichuan | 510115 | Wenjiang District | 6.0 | 4.8 | 7.2 |  | 96.7(91.8,99.0) | 96.9(92.4,99.3) | 94.9(87.2,98.5) |
| Sichuan | 510904 | Anju District | 8.1 | 6.6 | 9.5 |  | 96.3(92.3,98.6) | 95.8(90.5,98.6) | 96.0(90.4,98.8) |
| Sichuan | 510104 | Jinjiang District | 4.8 | 4.0 | 5.5 |  | 96.1(77.6,99.8) | 92.5(71.1,99.3) | 97.9(84.7,99.9) |
| Sichuan | 510124 | Pi County | 4.5 | 3.9 | 5.1 |  | 95.8(89.1,98.8) | 93.7(83.3,98.1) | 96.1(88.0,99.2) |
| Sichuan | 511529 | Pingshan County | 7.4 | 6.1 | 8.6 |  | 95.4(89.6,98.4) | 96.4(90.5,99.2) | 91.1(82.1,97.0) |
| Sichuan | 510626 | Luojiang County | 8.9 | 7.0 | 10.9 |  | 95.3(83.3,99.5) | 95.1(81.9,99.7) | 92.7(77.8,99.0) |
| Sichuan | 511602 | Guang'an District | 9.6 | 8.0 | 11.2 |  | 95.2(90.8,98.1) | 94.2(88.7,97.4) | 94.8(88.7,98.2) |
| Sichuan | 510113 | Qingbaijiang District | 8.3 | 6.9 | 9.8 |  | 94.3(87.5,98.0) | 92.3(84.3,97.1) | 94.4(86.5,98.6) |
| Sichuan | 510683 | Mianzhu City | 8.8 | 7.1 | 10.5 |  | 94.3(88.5,98.0) | 93.5(85.5,97.9) | 93.6(85.1,98.5) |
| Sichuan | 510823 | Jian'ge County | 10.0 | 8.0 | 11.9 |  | 93.8(88.3,97.0) | 95.1(89.6,98.2) | 88.8(80.3,94.3) |
| Sichuan | 510105 | Qingyang District | 4.5 | 3.6 | 5.5 |  | 93.6(85.2,98.1) | 91.0(78.4,97.3) | 94.5(83.7,99.1) |
| Sichuan | 510112 | Longquanyi District | 5.0 | 3.9 | 6.1 |  | 93.5(86.4,97.4) | 95.0(88.1,98.5) | 87.3(76.6,94.6) |
| Sichuan | 511011 | Dongxing District | 7.4 | 6.0 | 8.8 |  | 93.5(82.5,98.6) | 92.0(80.6,98.3) | 94.1(82.7,99.0) |
| Sichuan | 510704 | Youxian District | 6.8 | 5.6 | 8.0 |  | 93.3(85.8,98.2) | 92.8(82.9,98.0) | 92.9(81.9,98.5) |
| Sichuan | 511302 | Shunqing District | 5.9 | 4.7 | 7.3 |  | 93.3(85.7,97.7) | 94.8(87.6,98.4) | 88.9(78.7,95.4) |
| Sichuan | 510903 | Chuanshan District | 6.5 | 5.2 | 7.8 |  | 93.2(85.8,97.4) | 94.1(87.5,97.8) | 90.9(81.3,96.5) |
| Sichuan | 510132 | Xinjin County | 7.7 | 6.4 | 9.0 |  | 92.4(83.9,97.4) | 91.9(80.3,97.7) | 90.4(77.7,97.3) |
| Sichuan | 513434 | Yuexi County | 6.2 | 4.8 | 7.6 |  | 92.3(84.9,96.5) | 91.7(84.1,96.6) | 91.1(82.0,96.2) |
| Sichuan | 511527 | Junlian County | 7.2 | 6.2 | 8.2 |  | 92.0(85.6,96.0) | 93.4(86.1,97.4) | 87.0(77.0,93.4) |
| Sichuan | 511603 | Guang'an District | 9.0 | 7.4 | 10.6 |  | 91.7(84.2,96.5) | 89.6(80.3,95.5) | 92.1(82.6,97.8) |
| Sichuan | 510411 | Renhe District | 5.9 | 5.3 | 6.4 |  | 91.2(79.2,97.3) | 85.9(69.2,96.0) | 93.6(81.2,98.7) |
| Sichuan | 510781 | Jiangyou City | 7.2 | 5.8 | 8.6 |  | 91.1(83.6,95.7) | 88.8(79.8,94.5) | 92.6(83.7,97.3) |
| Sichuan | 511524 | Changning County | 9.1 | 7.4 | 10.7 |  | 91.1(84.4,95.3) | 91.5(84.6,96.1) | 87.6(78.9,93.5) |
| Sichuan | 511623 | Linshui County | 8.7 | 7.1 | 10.2 |  | 91.0(84.5,95.5) | 92.4(86.3,96.5) | 84.0(74.5,90.4) |
| Sichuan | 510311 | Yantan District | 6.2 | 5.0 | 7.4 |  | 90.6(78.4,96.7) | 90.3(75.1,97.5) | 88.6(73.8,96.9) |
| Sichuan | 511621 | Yuechi County | 8.9 | 7.0 | 10.8 |  | 90.6(83.0,95.4) | 91.3(83.7,96.1) | 85.5(75.6,92.3) |
| Sichuan | 511523 | Jiang'an County | 8.0 | 6.5 | 9.4 |  | 90.5(82.2,95.7) | 91.2(82.4,96.5) | 86.0(75.0,93.7) |
| Sichuan | 510322 | Fushun County | 9.1 | 7.2 | 11.0 |  | 89.9(83.4,94.3) | 88.8(81.5,94.2) | 88.8(81.0,94.1) |
| Sichuan | 510421 | Miyi County | 6.5 | 5.6 | 7.5 |  | 89.9(77.3,96.5) | 90.5(75.8,97.4) | 83.5(67.9,94.4) |
| Sichuan | 511112 | Wutongqiao District | 7.3 | 6.1 | 8.5 |  | 89.8(77.8,96.6) | 88.1(75.1,96.5) | 90.1(74.7,97.8) |
| Sichuan | 510623 | Zhongjiang County | 8.3 | 6.7 | 9.8 |  | 89.4(83.0,93.9) | 88.6(81.5,93.5) | 88.0(80.5,93.2) |
| Sichuan | 511423 | Hongya County | 8.1 | 7.1 | 9.1 |  | 88.9(78.2,95.4) | 85.4(73.0,93.6) | 90.4(79.2,97.0) |
| Sichuan | 510106 | Jinniu District | 4.1 | 3.5 | 4.7 |  | 88.3(76.4,95.5) | 90.2(77.6,96.9) | 81.0(67.6,92.5) |
| Sichuan | 510802 | Lizhou District | 5.3 | 4.4 | 6.1 |  | 88.2(77.3,94.8) | 91.0(79.5,97.4) | 82.8(71.0,92.0) |
| Sichuan | 511111 | Shawan District | 7.9 | 6.7 | 9.1 |  | 88.2(75.6,96.4) | 91.7(76.3,98.7) | 79.0(69.3,86.3) |
| Sichuan | 510682 | Shifang City | 8.0 | 6.6 | 9.3 |  | 88.1(78.2,94.3) | 83.9(72.9,92.4) | 90.4(79.1,96.9) |
| Sichuan | 511303 | Gaoping District | 6.8 | 5.6 | 7.9 |  | 88.0(80.1,93.8) | 80.5(70.8,88.7) | 93.7(85.3,98.0) |
| Sichuan | 510131 | Pujiang County | 7.6 | 6.3 | 8.9 |  | 87.8(76.2,95.3) | 88.2(75.3,96.6) | 83.7(68.6,94.6) |
| Sichuan | 512081 | Jianyang City | 8.9 | 6.9 | 10.9 |  | 87.6(79.8,92.8) | 86.5(78.3,92.2) | 84.4(75.8,91.0) |
| Sichuan | 511503 | Nanxi District | 7.5 | 6.2 | 8.8 |  | 87.5(79.6,93.4) | 85.6(76.4,92.6) | 88.4(78.4,94.6) |
| Sichuan | 511526 | Gong County | 6.6 | 5.7 | 7.6 |  | 87.5(78.1,94.2) | 85.8(74.7,93.7) | 87.5(77.2,94.8) |
| Sichuan | 511525 | Gao County | 8.0 | 6.4 | 9.5 |  | 87.3(79.6,92.9) | 88.5(80.6,94.1) | 82.8(73.2,89.9) |
| Sichuan | 511827 | Baoxing County | 6.6 | 5.6 | 7.5 |  | 87.3(62.7,98.4) | 93.6(69.8,99.7) | 59.7(45.3,74.0) |
| Sichuan | 510321 | Rong County | 9.6 | 7.9 | 11.3 |  | 87.2(79.1,92.8) | 85.4(76.6,92.0) | 86.2(77.3,92.5) |
| Sichuan | 510524 | Xuyong County | 8.1 | 6.9 | 9.2 |  | 87.2(80.3,92.4) | 85.3(75.5,91.2) | 88.5(80.7,93.6) |
| Sichuan | 510129 | Dayi County | 7.7 | 6.5 | 8.8 |  | 87.1(77.6,93.6) | 85.0(73.1,93.3) | 87.6(76.8,94.8) |
| Sichuan | 510184 | Chongzhou City | 8.1 | 6.7 | 9.5 |  | 87.0(78.4,92.9) | 87.6(76.8,94.2) | 83.3(73.6,91.7) |
| Sichuan | 510181 | Dujiangyan City | 7.0 | 5.8 | 8.2 |  | 86.9(77.0,93.5) | 85.9(74.9,93.5) | 86.2(73.6,93.5) |
| Sichuan | 510723 | Yanting County | 9.0 | 7.6 | 10.5 |  | 86.7(76.9,93.4) | 85.4(74.0,93.4) | 84.7(71.9,93.3) |
| Sichuan | 510502 | Jiangyang District | 6.5 | 5.1 | 8.0 |  | 86.4(76.9,92.3) | 84.0(75.0,90.9) | 89.1(77.4,95.7) |
| Sichuan | 510403 | Xi District | 5.5 | 4.1 | 6.8 |  | 86.3(61.6,99.0) | 90.8(60.5,99.6) | 77.3(51.6,97.2) |
| Sichuan | 510681 | Guanghan City | 7.6 | 6.2 | 9.1 |  | 86.3(76.8,92.5) | 85.1(75.0,93.2) | 84.9(72.8,93.3) |
| Sichuan | 511424 | Danling County | 7.0 | 5.9 | 8.2 |  | 86.2(71.4,96.1) | 86.3(66.4,96.9) | 83.3(63.1,96.7) |
| Sichuan | 510182 | Pengzhou City | 7.8 | 6.5 | 9.1 |  | 86.0(77.8,92.4) | 84.4(74.6,91.6) | 86.6(76.5,93.4) |
| Sichuan | 511425 | Qingshen County | 9.0 | 7.6 | 10.4 |  | 86.0(71.7,95.4) | 80.5(65.4,91.6) | 89.5(67.8,98.0) |
| Sichuan | 510811 | Yuanba District | 7.1 | 6.4 | 7.8 |  | 85.6(70.4,94.1) | 79.5(63.1,92.8) | 89.3(71.7,97.8) |
| Sichuan | 511528 | Xingwen County | 8.2 | 6.7 | 9.5 |  | 85.3(76.9,91.4) | 85.9(76.7,92.0) | 82.5(72.5,89.8) |
| Sichuan | 511422 | Pengshan County | 8.0 | 6.5 | 9.5 |  | 85.2(73.6,93.4) | 86.9(73.9,95.4) | 79.3(63.3,91.3) |
| Sichuan | 511502 | Cuiping District | 6.7 | 5.5 | 7.8 |  | 85.2(75.3,91.3) | 83.2(74.7,90.1) | 87.2(77.6,93.3) |
| Sichuan | 510812 | Chaotian District | 7.4 | 6.4 | 8.5 |  | 85.0(73.6,93.2) | 82.5(70.1,93.0) | 87.1(73.6,95.4) |
| Sichuan | 510504 | Longmatan District | 6.5 | 5.1 | 7.9 |  | 84.9(73.5,92.4) | 86.2(75.4,93.9) | 81.5(67.9,92.3) |
| Sichuan | 510183 | Qionglai City | 8.0 | 6.8 | 9.1 |  | 84.7(75.1,91.4) | 80.5(68.5,88.9) | 86.8(76.2,94.0) |
| Sichuan | 510304 | Daan District | 6.7 | 5.2 | 8.1 |  | 84.7(73.6,92.1) | 82.4(71.3,91.1) | 85.1(71.4,94.4) |
| Sichuan | 510923 | Daying County | 6.8 | 5.8 | 7.8 |  | 84.4(68.3,95.8) | 85.4(66.0,96.1) | 81.9(66.4,92.8) |
| Sichuan | 512002 | Yanjiang District | 7.2 | 5.5 | 8.9 |  | 84.4(75.8,90.5) | 85.7(76.6,91.8) | 79.7(69.5,87.9) |
| Sichuan | 512022 | Lezhi County | 10.4 | 8.5 | 12.4 |  | 84.2(65.2,95.1) | 84.0(60.4,96.1) | 75.5(55.2,89.9) |
| Sichuan | 510503 | Naxi District | 6.4 | 5.3 | 7.5 |  | 84.1(74.7,91.0) | 85.2(75.0,92.8) | 81.9(70.8,90.4) |
| Sichuan | 511826 | Lushan County | 7.3 | 6.5 | 8.0 |  | 84.1(66.9,94.5) | 81.6(60.7,95.7) | 84.1(64.9,95.1) |
| Sichuan | 511133 | Mabian Yizu Zizhixian | 6.2 | 5.4 | 7.0 |  | 83.5(71.5,92.2) | 83.1(70.0,92.4) | 82.2(68.7,91.1) |
| Sichuan | 511304 | Jialing District | 7.1 | 6.1 | 8.1 |  | 83.1(73.8,90.3) | 78.2(67.2,86.9) | 87.8(78.4,94.4) |
| Sichuan | 511681 | Huaying City | 8.0 | 6.3 | 9.7 |  | 83.1(63.4,95.0) | 81.5(63.1,94.2) | 82.7(62.4,96.4) |
| Sichuan | 510121 | Jintang County | 8.6 | 6.9 | 10.2 |  | 83.0(73.7,88.9) | 82.1(72.1,89.7) | 80.9(71.3,88.7) |
| Sichuan | 511823 | Hanyuan County | 6.7 | 5.8 | 7.5 |  | 82.9(71.1,91.2) | 86.8(72.6,94.6) | 73.5(60.9,83.2) |
| Sichuan | 511803 | Mingshan District | 7.0 | 6.1 | 7.9 |  | 82.8(70.7,91.3) | 82.4(68.9,92.2) | 81.6(68.4,91.3) |
| Sichuan | 511521 | Yibin County | 6.9 | 5.6 | 8.2 |  | 82.6(73.5,89.3) | 81.0(71.3,88.6) | 83.0(73.6,90.1) |
| Sichuan | 511822 | Yingjing County | 5.5 | 4.8 | 6.2 |  | 82.4(64.5,94.4) | 78.1(58.3,93.0) | 85.3(64.0,97.0) |
| Sichuan | 510603 | Jingyang District | 6.4 | 5.2 | 7.6 |  | 81.8(71.7,89.2) | 83.2(72.6,90.8) | 79.4(68.9,88.2) |
| Sichuan | 511181 | Emeishan City | 6.2 | 5.2 | 7.3 |  | 81.7(68.6,91.4) | 82.5(65.9,93.2) | 77.6(59.9,91.4) |
| Sichuan | 511126 | Jiajiang County | 7.1 | 6.2 | 7.9 |  | 81.6(69.2,91.2) | 81.3(65.5,93.4) | 78.4(63.6,91.3) |
| Sichuan | 511102 | Shizhong District | 6.2 | 5.2 | 7.2 |  | 81.5(70.3,90.5) | 83.9(71.5,93.1) | 77.1(64.9,87.8) |
| Sichuan | 511402 | Dongpo District | 6.9 | 5.6 | 8.3 |  | 81.4(71.7,89.0) | 84.1(74.8,91.9) | 77.1(66.2,85.9) |
| Sichuan | 511921 | Tongjiang County | 7.2 | 6.2 | 8.1 |  | 81.1(71.4,88.3) | 79.6(68.7,87.4) | 82.4(73.5,90.5) |
| Sichuan | 510402 | Dong District | 5.0 | 3.9 | 6.1 |  | 81.0(54.6,96.5) | 82.9(56.5,97.5) | 75.8(51.1,94.5) |
| Sichuan | 510302 | Ziliujing District | 4.8 | 3.8 | 5.9 |  | 80.9(68.0,91.7) | 81.5(66.0,93.3) | 80.1(64.6,92.8) |
| Sichuan | 510726 | Zizhixian | 7.4 | 5.8 | 8.9 |  | 80.8(67.8,90.5) | 80.3(66.5,90.0) | 78.2(58.6,92.4) |
| Sichuan | 512021 | Anyue County | 8.0 | 6.5 | 9.5 |  | 80.8(62.3,92.7) | 81.0(60.8,93.4) | 78.1(59.2,90.8) |
| Sichuan | 511124 | Jingyan County | 9.3 | 7.2 | 11.4 |  | 80.6(60.5,94.3) | 79.2(59.1,94.5) | 76.2(53.2,92.9) |
| Sichuan | 510921 | Pengxi County | 8.2 | 6.4 | 9.9 |  | 79.9(69.8,88.0) | 77.9(67.2,86.6) | 80.2(67.8,89.8) |
| Sichuan | 511622 | Wusheng County | 8.4 | 7.0 | 9.7 |  | 79.9(69.3,88.3) | 77.0(64.8,85.9) | 81.0(69.0,90.0) |
| Sichuan | 511025 | Zizhong County | 7.1 | 5.7 | 8.5 |  | 79.4(70.4,86.4) | 80.8(70.7,88.0) | 76.8(67.1,84.5) |
| Sichuan | 511028 | Longchang County | 7.4 | 6.0 | 8.7 |  | 79.4(70.4,86.5) | 77.1(66.9,84.3) | 82.2(71.9,90.0) |
| Sichuan | 511825 | Tianquan County | 7.0 | 6.2 | 7.7 |  | 79.4(64.3,90.5) | 80.0(62.4,93.0) | 76.2(61.4,88.2) |
| Sichuan | 511123 | Qianweixian | 8.7 | 7.3 | 10.1 |  | 78.6(68.5,86.5) | 75.9(64.4,85.2) | 79.7(68.5,88.6) |
| Sichuan | 510722 | Santai County | 8.4 | 6.8 | 10.0 |  | 78.3(68.1,85.5) | 75.4(64.6,83.1) | 79.0(69.0,86.2) |
| Sichuan | 511903 | Bazhou District | 6.8 | 6.0 | 7.5 |  | 77.1(52.4,91.9) | 79.8(54.3,95.6) | 68.3(45.6,86.5) |
| Sichuan | 511922 | Nanjiang County | 6.4 | 5.3 | 7.5 |  | 76.7(64.6,86.1) | 81.2(68.6,90.3) | 66.8(54.7,77.3) |
| Sichuan | 511129 | Muchuan County | 6.6 | 5.3 | 7.8 |  | 76.4(62.6,87.0) | 74.0(59.2,87.2) | 76.4(59.0,90.7) |
| Sichuan | 511421 | Renshou County | 8.4 | 6.6 | 10.2 |  | 76.2(66.0,83.9) | 77.3(66.1,85.4) | 71.4(60.9,80.0) |
| Sichuan | 511002 | Shizhong District | 6.7 | 5.5 | 7.9 |  | 75.9(55.5,91.5) | 78.6(58.0,92.6) | 71.3(50.1,88.0) |
| Sichuan | 511721 | Da County | 7.2 | 6.1 | 8.2 |  | 75.6(66.1,84.4) | 75.3(65.6,83.8) | 76.1(66.3,84.7) |
| Sichuan | 511702 | Tongchuan District | 4.5 | 3.7 | 5.3 |  | 75.4(62.5,86.4) | 74.3(60.2,87.2) | 76.2(61.2,88.8) |
| Sichuan | 511024 | Weiyuan County | 6.6 | 5.3 | 7.9 |  | 74.7(63.5,83.6) | 73.8(62.8,83.0) | 74.7(61.8,86.0) |
| Sichuan | 510522 | Hejiang County | 8.4 | 6.7 | 10.1 |  | 74.6(55.7,88.7) | 75.1(54.4,87.9) | 72.4(51.9,87.1) |
| Sichuan | 510725 | Zitong County | 7.4 | 6.3 | 8.5 |  | 74.5(62.2,84.2) | 74.4(61.4,85.8) | 73.3(59.3,85.5) |
| Sichuan | 511802 | Yucheng District | 6.1 | 5.2 | 6.9 |  | 74.4(62.1,86.1) | 70.2(56.4,83.3) | 79.9(64.1,92.4) |
| Sichuan | 510521 | Lu County | 8.9 | 6.9 | 10.9 |  | 74.3(54.7,88.4) | 77.3(57.9,90.1) | 65.7(47.3,81.8) |
| Sichuan | 511324 | Yilong County | 6.2 | 5.0 | 7.4 |  | 74.2(62.4,83.7) | 74.4(61.9,84.2) | 70.1(56.8,81.3) |
| Sichuan | 511132 | Ebian Yizu Zizhixian | 5.3 | 4.3 | 6.1 |  | 73.8(55.8,86.7) | 68.8(52.0,85.6) | 75.5(56.4,92.2) |
| Sichuan | 510525 | Gulin County | 7.2 | 6.1 | 8.3 |  | 73.6(55.6,87.7) | 72.5(52.5,87.4) | 76.6(56.4,89.2) |
| Sichuan | 510303 | Gongjing District | 8.2 | 6.6 | 9.8 |  | 73.0(59.6,83.5) | 68.5(56.2,79.7) | 75.2(59.1,90.3) |
| Sichuan | 511323 | Peng'an County | 6.4 | 5.3 | 7.5 |  | 73.0(60.5,83.1) | 70.6(56.1,82.8) | 75.0(61.3,86.2) |
| Sichuan | 511325 | Xichong County | 7.2 | 5.9 | 8.6 |  | 73.0(60.3,83.6) | 72.4(59.4,83.8) | 69.2(55.3,82.5) |
| Sichuan | 511824 | Shimian County | 6.4 | 5.3 | 7.4 |  | 73.0(57.9,85.3) | 72.0(57.3,86.6) | 72.1(51.3,88.0) |
| Sichuan | 511923 | Pingchang County | 6.8 | 6.1 | 7.6 |  | 72.9(52.1,88.9) | 70.5(48.8,86.2) | 77.6(56.9,90.3) |
| Sichuan | 510724 | An County | 7.3 | 5.9 | 8.8 |  | 72.5(62.4,81.7) | 67.7(56.3,77.1) | 77.9(64.9,88.2) |
| Sichuan | 511902 | Bazhou District | 6.2 | 5.1 | 7.2 |  | 70.5(51.8,87.4) | 67.9(46.8,85.3) | 76.1(55.4,92.0) |
| Sichuan | 510422 | Yanbian County | 6.3 | 5.7 | 6.8 |  | 69.8(55.6,82.1) | 63.8(47.8,78.5) | 73.9(57.8,87.1) |
| Sichuan | 510821 | Wangcang County | 6.4 | 5.4 | 7.4 |  | 69.8(56.3,81.6) | 69.1(55.4,81.2) | 70.9(55.4,84.0) |
| Sichuan | 511724 | Dazhu County | 7.3 | 6.2 | 8.4 |  | 69.0(56.7,78.5) | 71.2(59.3,81.0) | 63.2(50.9,73.8) |
| Sichuan | 511722 | Xuanhan County | 7.0 | 5.9 | 8.0 |  | 68.7(57.1,77.7) | 67.0(55.6,77.0) | 70.2(58.8,80.0) |
| Sichuan | 513222 | Li County | 5.5 | 5.3 | 5.8 |  | 68.1(46.3,89.4) | 51.1(32.0,78.2) | 80.2(50.8,97.1) |
| Sichuan | 510107 | Wuhou District | 2.6 | 2.2 | 3.0 |  | 67.7(52.7,82.0) | 64.7(48.8,81.5) | 70.9(55.4,85.4) |
| Sichuan | 511725 | Qu County | 6.8 | 5.6 | 8.1 |  | 67.6(56.8,77.0) | 68.6(57.5,78.0) | 66.0(54.9,75.3) |
| Sichuan | 513221 | Wenchuan County | 4.2 | 3.4 | 4.9 |  | 66.7(45.8,86.8) | 78.8(50.9,95.7) | 46.4(34.9,58.1) |
| Sichuan | 511723 | Kaijiang County | 7.6 | 6.0 | 9.2 |  | 65.8(54.7,75.8) | 69.3(57.1,79.2) | 58.6(47.3,69.9) |
| Sichuan | 511381 | Langzhong City | 7.9 | 6.5 | 9.4 |  | 65.4(52.7,76.7) | 68.0(55.0,79.2) | 57.9(45.0,70.8) |
| Sichuan | 513427 | Ningnan County | 5.9 | 5.3 | 6.5 |  | 64.2(49.4,79.4) | 55.0(39.4,71.8) | 73.5(54.1,88.0) |
| Sichuan | 510922 | Shehong County | 6.4 | 5.1 | 7.7 |  | 63.1(52.0,73.7) | 66.1(54.0,77.4) | 59.0(45.4,70.1) |
| Sichuan | 513437 | Leibo County | 3.7 | 3.0 | 4.3 |  | 62.2(45.0,79.0) | 60.4(44.6,77.6) | 62.2(46.2,79.1) |
| Sichuan | 513224 | Songpan County | 4.0 | 3.1 | 4.7 |  | 62.1(40.5,82.6) | 74.4(47.9,93.7) | 35.9(22.0,57.8) |
| Sichuan | 510727 | Pingwu County | 6.2 | 5.2 | 7.3 |  | 57.6(45.7,70.0) | 56.3(44.3,67.3) | 60.8(46.4,78.7) |
| Sichuan | 511113 | Jinkouhe District | 4.9 | 4.3 | 5.5 |  | 57.3(38.3,74.5) | 57.4(37.3,76.8) | 60.6(39.4,79.2) |
| Sichuan | 511781 | Wanyuan City | 6.9 | 5.5 | 8.3 |  | 52.1(39.3,64.0) | 53.2(40.8,66.5) | 49.0(37.6,61.7) |
| Sichuan | 513401 | Xichang City | 3.6 | 3.2 | 4.0 |  | 50.3(36.7,65.8) | 47.3(32.6,64.4) | 55.2(40.0,70.4) |
| Sichuan | 513336 | County | 4.4 | 4.4 | 4.5 |  | 49.1(30.8,73.7) | 38.3(23.6,63.2) | 62.4(36.4,88.7) |
| Sichuan | 510824 | Cangxi County | 6.9 | 5.7 | 8.2 |  | 48.4(36.4,61.2) | 50.2(37.7,62.7) | 45.0(33.4,59.1) |
| Sichuan | 513321 | County | 3.7 | 3.4 | 4.0 |  | 48.4(34.3,65.2) | 40.6(26.8,58.4) | 59.9(41.2,79.5) |
| Sichuan | 513322 | Luding County | 4.7 | 3.8 | 5.6 |  | 48.3(33.9,65.7) | 46.4(32.7,63.7) | 50.9(32.9,74.3) |
| Sichuan | 511322 | Yingshan County | 5.8 | 4.9 | 6.7 |  | 45.8(35.2,57.2) | 46.0(35.0,58.6) | 45.7(34.5,58.1) |
| Sichuan | 513227 | Xiaojin County | 4.5 | 4.2 | 4.8 |  | 43.1(20.3,75.3) | 36.2(17.2,73.7) | 50.5(23.8,84.5) |
| Sichuan | 513425 | Huili County | 3.5 | 3.2 | 3.9 |  | 38.9(27.6,53.6) | 34.7(23.0,49.3) | 44.2(30.1,62.6) |
| Sichuan | 513424 | Dechang County | 3.7 | 2.9 | 4.4 |  | 38.8(21.5,66.0) | 43.4(22.4,69.4) | 37.8(20.9,61.0) |
| Sichuan | 513426 | Huidong County | 3.9 | 3.1 | 4.6 |  | 35.5(24.7,49.3) | 38.0(25.4,52.9) | 33.3(22.0,48.0) |
| Sichuan | 513233 | Hongyuan County | 3.1 | 2.8 | 3.4 |  | 35.2(20.7,58.4) | 32.5(16.5,60.6) | 37.3(20.9,64.3) |
| Sichuan | 513225 | Jiuzhaigou County | 2.1 | 1.8 | 2.3 |  | 33.2(17.0,62.3) | 37.8(16.2,80.1) | 32.6(19.1,50.6) |
| Sichuan | 513223 | Mao County | 2.6 | 2.5 | 2.7 |  | 29.4(14.7,59.7) | 23.1(10.8,47.4) | 37.9(17.3,73.2) |
| Sichuan | 513230 | Zamtang County | 5.4 | 4.8 | 5.9 |  | 28.3(17.9,44.3) | 32.3(17.4,53.4) | 23.0(12.8,36.6) |
| Sichuan | 511321 | Nanbu County | 4.3 | 3.4 | 5.3 |  | 27.4(18.9,37.7) | 31.9(21.6,44.4) | 22.8(16.0,31.9) |
| Sichuan | 513323 | County | 4.4 | 3.6 | 5.1 |  | 25.1(12.8,49.0) | 31.2(14.2,67.2) | 18.7(9.2,32.4) |
| Sichuan | 513337 | County | 5.1 | 5.1 | 5.1 |  | 24.0(12.3,44.1) | 20.9(9.8,40.7) | 31.5(14.8,58.4) |
| Sichuan | 513333 | Seertar County | 4.3 | 4.4 | 4.2 |  | 23.9(16.2,33.9) | 18.5(12.0,28.2) | 35.6(23.8,52.7) |
| Sichuan | 513232 | Zoigee County | 3.1 | 3.0 | 3.3 |  | 23.2(15.3,34.0) | 18.7(12.3,28.5) | 31.6(18.6,51.0) |
| Sichuan | 513431 | Zhaojue County | 2.7 | 1.6 | 3.7 |  | 22.4(15.7,31.9) | 26.1(18.4,36.4) | 21.0(13.8,30.5) |
| Sichuan | 513338 | Deerong County | 2.5 | 2.8 | 2.3 |  | 22.1(10.0,53.0) | 19.0(7.7,55.6) | 23.8(10.6,60.6) |
| Sichuan | 513433 | Mianning County | 2.7 | 2.3 | 3.0 |  | 21.4(14.3,30.9) | 19.6(12.8,28.1) | 26.0(17.7,37.0) |
| Sichuan | 513335 | Batang County | 3.1 | 2.8 | 3.3 |  | 20.6(14.2,29.0) | 19.6(12.6,28.5) | 26.0(17.1,37.3) |
| Sichuan | 513229 | Barkam County | 4.0 | 3.4 | 4.5 |  | 19.8(9.7,36.3) | 18.6(9.6,32.8) | 22.0(10.4,49.5) |
| Sichuan | 513329 | County | 3.2 | 3.0 | 3.5 |  | 17.7(10.8,28.5) | 16.5(9.9,29.4) | 21.1(12.3,35.6) |
| Sichuan | 513228 | Heishui County | 2.9 | 2.9 | 2.8 |  | 15.6(7.8,31.6) | 15.7(6.7,37.8) | 16.5(8.2,32.3) |
| Sichuan | 513231 | Aba(Ngawa) County | 1.8 | 1.8 | 1.9 |  | 15.5(9.2,29.3) | 15.7(8.3,34.1) | 15.7(9.0,30.9) |
| Sichuan | 513226 | County | 2.6 | 2.3 | 2.8 |  | 14.3(6.0,36.0) | 16.8(6.0,24.5) | 11.2(5.3,21.8) |
| Sichuan | 513324 | Jiulong(Gyaisi) County | 1.3 | 1.0 | 1.6 |  | 13.9(8.1,24.4) | 14.4(8.6,24.7) | 14.3(8.4,27.9) |
| Sichuan | 513331 | Baiyu County | 1.4 | 1.4 | 1.5 |  | 13.0(7.4,25.1) | 14.1(7.0,31.7) | 11.7(7.5,17.9) |
| Sichuan | 513432 | Xide County | 1.3 | 1.1 | 1.6 |  | 12.6(8.4,19.3) | 11.4(7.6,17.3) | 17.0(10.9,25.2) |
| Sichuan | 513423 | Yanyuan County | 0.8 | 0.6 | 0.9 |  | 11.2(7.3,16.8) | 11.0(7.0,16.8) | 13.5(9.0,19.7) |
| Sichuan | 513332 | SeerxuuXian | 0.5 | 0.5 | 0.6 |  | 10.3(5.3,22.0) | 9.8(4.4,23.7) | 12.7(6.4,26.8) |
| Sichuan | 513325 | County | 1.4 | 1.1 | 1.6 |  | 8.8(5.4,14.2) | 8.4(5.1,13.3) | 10.4(5.9,20.4) |
| Sichuan | 513326 | Dawu County | 1.3 | 1.4 | 1.3 |  | 8.6(5.4,13.8) | 6.6(4.2,10.0) | 13.3(8.1,24.6) |
| Sichuan | 513327 | Luhuo(Zhaggo) County | 1.6 | 1.4 | 1.8 |  | 8.4(5.0,14.6) | 6.7(3.9,12.1) | 13.1(6.7,27.7) |
| Sichuan | 513422 | Muli Zangzu Zizhixian | 0.6 | 0.5 | 0.7 |  | 8.1(4.8,13.5) | 8.2(4.8,14.1) | 8.7(5.3,15.2) |
| Sichuan | 513334 | Litang County | 1.3 | 1.3 | 1.3 |  | 7.7(4.8,12.2) | 6.3(3.9,9.7) | 11.6(7.1,19.7) |
| Sichuan | 513435 | Ganluo County | 0.4 | 0.3 | 0.6 |  | 7.3(4.9,10.8) | 7.5(4.8,11.2) | 8.4(5.7,13.0) |
| Sichuan | 513328 | Garzee County | 1.0 | 1.0 | 0.9 |  | 7.2(4.6,11.3) | 5.8(3.5,9.0) | 11.1(6.8,19.5) |
| Sichuan | 513430 | Jinyang County | 1.0 | 0.7 | 1.3 |  | 6.9(4.6,10.6) | 7.2(4.5,10.6) | 8.1(5.3,12.3) |
| Sichuan | 513428 | Puge County | 0.4 | 0.3 | 0.5 |  | 6.3(4.0,9.8) | 5.6(3.5,8.8) | 8.8(5.4,14.6) |
| Sichuan | 513330 | Deegee County | 0.3 | 0.3 | 0.3 |  | 6.1(3.7,10.5) | 5.3(3.1,9.0) | 8.2(4.7,15.3) |
| Sichuan | 513436 | Meigu County | 0.4 | 0.2 | 0.6 |  | 4.1(2.0,7.8) | 3.8(1.8,7.6) | 5.4(2.7,10.7) |
| Sichuan | 513429 | Butuo County | 1.3 | 0.9 | 1.6 |  | 4.0(2.4,6.3) | 4.3(2.6,6.9) | 4.0(2.4,6.4) |
| Hebei | 130304 | Beidaihe District | 10.8 | 8.3 | 13.7 |  | 99.7(96.7,100.0) | 99.5(94.3,100.0) | 99.4(91.4,100.0) |
| Hebei | 131082 | Sanhe City | 6.3 | 5.3 | 7.2 |  | 96.4(88.1,99.4) | 94.9(83.6,98.9) | 97.0(87.9,99.6) |
| Hebei | 130503 | Qiaoxi District | 7.5 | 5.9 | 9.1 |  | 95.8(90.0,98.5) | 94.8(88.6,98.3) | 95.6(88.7,98.8) |
| Hebei | 130502 | Qiaodong District | 6.0 | 5.1 | 7.0 |  | 94.4(86.8,98.3) | 92.9(83.2,98.0) | 95.3(86.8,99.0) |
| Hebei | 130104 | Qiaoxi District | 6.0 | 4.9 | 7.2 |  | 93.3(79.1,98.8) | 94.8(82.2,99.2) | 88.6(72.9,97.5) |
| Hebei | 130527 | Nanhe County | 8.5 | 7.2 | 9.8 |  | 92.8(87.9,95.9) | 91.7(85.7,95.3) | 94.2(89.2,97.0) |
| Hebei | 130302 | Haigang District | 5.7 | 4.8 | 6.6 |  | 92.2(85.2,96.8) | 84.3(73.5,91.9) | 97.1(90.9,99.4) |
| Hebei | 130303 | Shanhaiguan District | 7.5 | 6.9 | 8.0 |  | 90.7(76.7,98.1) | 76.5(66.2,84.6) | 97.2(81.6,99.9) |
| Hebei | 130526 | Ren County | 8.8 | 7.7 | 9.9 |  | 89.9(84.0,94.0) | 88.4(82.1,93.4) | 91.3(85.3,95.4) |
| Hebei | 130102 | Changan District | 7.1 | 5.8 | 8.4 |  | 89.2(75.9,96.3) | 90.1(77.0,96.8) | 87.5(72.3,96.1) |
| Hebei | 130622 | Qingyuan County | 6.4 | 5.6 | 7.2 |  | 88.9(82.4,93.2) | 86.1(78.8,91.5) | 92.2(85.7,96.4) |
| Hebei | 130703 | Qiaoxi District | 6.9 | 5.7 | 8.2 |  | 88.9(76.9,96.5) | 87.4(73.3,96.4) | 89.5(73.0,97.8) |
| Hebei | 130124 | Luancheng County | 6.1 | 5.2 | 7.0 |  | 88.7(80.9,94.0) | 86.6(78.3,93.1) | 89.5(80.4,95.4) |
| Hebei | 130603 | Beishi District | 4.7 | 4.0 | 5.5 |  | 88.5(74.0,96.6) | 89.9(77.4,97.8) | 86.6(71.7,95.9) |
| Hebei | 130903 | Yunhe District | 5.6 | 4.5 | 6.9 |  | 88.3(80.0,94.7) | 86.0(74.9,93.5) | 90.8(79.6,96.9) |
| Hebei | 130803 | Shuangluan District | 6.4 | 5.2 | 7.5 |  | 88.2(74.8,95.3) | 89.5(75.9,97.1) | 83.9(70.7,95.1) |
| Hebei | 130582 | Shahe City | 7.0 | 6.2 | 7.9 |  | 87.6(80.7,92.7) | 85.6(77.6,91.4) | 90.9(84.0,95.2) |
| Hebei | 130921 | Cang County | 6.7 | 5.8 | 7.5 |  | 87.6(75.4,95.0) | 84.3(70.8,93.7) | 91.3(80.3,97.4) |
| Hebei | 130625 | Xushui County | 6.4 | 5.1 | 7.6 |  | 87.3(79.0,92.6) | 87.6(79.3,93.3) | 86.4(77.3,92.5) |
| Hebei | 130425 | Daming County | 6.9 | 5.8 | 8.0 |  | 86.3(79.3,91.7) | 86.4(79.3,91.4) | 87.6(80.5,92.7) |
| Hebei | 130638 | Xiong County | 7.4 | 6.4 | 8.3 |  | 85.8(78.4,91.4) | 86.1(78.9,91.1) | 87.0(79.9,92.1) |
| Hebei | 130205 | Kaiping District | 6.4 | 5.3 | 7.5 |  | 85.2(73.8,93.7) | 87.3(75.8,95.5) | 82.8(70.1,92.7) |
| Hebei | 131023 | Yongqing County | 7.3 | 6.6 | 8.1 |  | 84.5(76.6,89.9) | 83.0(74.3,89.4) | 86.4(79.9,92.2) |
| Hebei | 130529 | Julu County | 6.7 | 5.7 | 7.8 |  | 84.4(76.4,89.7) | 82.6(74.0,88.9) | 86.8(77.7,93.1) |
| Hebei | 130223 | Luan County | 6.4 | 5.4 | 7.3 |  | 84.2(75.8,90.2) | 82.1(72.9,88.2) | 86.6(77.4,93.1) |
| Hebei | 130523 | Neiqiu County | 7.8 | 6.3 | 9.2 |  | 84.2(68.4,93.1) | 86.7(73.3,94.8) | 79.3(65.6,90.0) |
| Hebei | 130804 | District | 7.4 | 5.9 | 8.9 |  | 84.2(63.1,97.9) | 75.4(63.9,83.9) | 90.5(55.3,99.9) |
| Hebei | 131028 | Zizhixian | 7.1 | 6.4 | 7.8 |  | 84.2(67.5,95.7) | 75.3(57.6,88.8) | 91.5(75.4,98.8) |
| Hebei | 130283 | Qian'an City | 6.7 | 5.8 | 7.6 |  | 83.9(77.1,89.0) | 83.5(75.8,89.2) | 84.5(76.6,90.0) |
| Hebei | 130626 | Dingxing County | 6.6 | 5.6 | 7.7 |  | 83.9(70.1,93.3) | 79.6(66.8,89.3) | 90.1(77.4,96.5) |
| Hebei | 130632 | Anxin County | 6.6 | 6.0 | 7.2 |  | 83.9(76.4,89.9) | 82.0(73.4,88.3) | 88.3(81.2,93.1) |
| Hebei | 130535 | Linxi County | 7.1 | 6.0 | 8.1 |  | 82.7(74.0,89.2) | 82.3(73.4,89.3) | 83.5(73.2,90.9) |
| Hebei | 130602 | Xinshi District | 5.4 | 4.4 | 6.4 |  | 82.5(64.0,95.0) | 85.2(66.3,96.2) | 79.0(59.8,93.5) |
| Hebei | 130827 | Zizhixian | 6.0 | 5.1 | 6.8 |  | 82.0(71.7,89.5) | 81.3(71.5,89.7) | 83.4(73.1,91.4) |
| Hebei | 130432 | Guangping County | 6.6 | 5.6 | 7.7 |  | 81.9(72.9,88.3) | 82.7(73.6,89.4) | 82.6(72.9,89.2) |
| Hebei | 130902 | Xinhua District | 6.3 | 5.1 | 7.6 |  | 81.7(66.2,93.4) | 72.5(58.9,83.8) | 90.8(74.4,98.4) |
| Hebei | 130633 | Yi County | 6.6 | 5.2 | 7.9 |  | 81.5(73.0,88.3) | 82.5(74.1,88.8) | 81.5(71.4,89.2) |
| Hebei | 130404 | Fuxing District | 7.1 | 6.2 | 8.0 |  | 81.2(61.5,93.9) | 78.6(60.3,93.6) | 82.3(62.5,95.2) |
| Hebei | 131026 | Wen'an County | 6.5 | 5.9 | 7.1 |  | 81.1(73.6,87.2) | 77.9(69.2,84.9) | 85.9(78.3,91.5) |
| Hebei | 130430 | Qiu County | 6.0 | 5.0 | 7.1 |  | 80.6(71.7,87.4) | 80.7(71.2,88.0) | 83.7(74.7,91.4) |
| Hebei | 130105 | Xinhua District | 3.8 | 3.1 | 4.5 |  | 80.1(65.4,90.5) | 84.7(70.5,94.7) | 69.5(54.7,85.0) |
| Hebei | 130131 | Pingshan County | 7.5 | 6.6 | 8.4 |  | 80.1(72.2,86.9) | 78.9(69.3,85.7) | 83.0(74.7,89.0) |
| Hebei | 130533 | Wei County | 6.9 | 6.2 | 7.5 |  | 80.1(72.2,86.5) | 79.2(70.8,85.6) | 83.5(75.9,89.5) |
| Hebei | 130534 | Qinghe County | 7.1 | 6.3 | 7.8 |  | 79.8(71.1,86.8) | 80.5(71.5,86.7) | 81.0(72.8,87.7) |
| Hebei | 130824 | Luanping County | 5.5 | 4.6 | 6.4 |  | 79.8(69.4,87.9) | 79.3(68.1,88.4) | 81.0(68.6,90.9) |
| Hebei | 130207 | Fengnan District | 6.1 | 5.6 | 6.6 |  | 79.7(69.9,87.2) | 76.8(65.9,86.0) | 85.1(76.4,91.2) |
| Hebei | 130629 | Rongcheng County | 6.3 | 5.4 | 7.1 |  | 79.7(69.2,87.7) | 79.8(68.2,88.8) | 80.5(69.4,88.3) |
| Hebei | 130431 | Jize County | 6.4 | 5.5 | 7.4 |  | 79.3(70.5,86.4) | 79.0(71.0,85.8) | 82.7(73.6,88.9) |
| Hebei | 130481 | Wu'an City | 6.1 | 5.2 | 6.9 |  | 79.2(70.5,85.2) | 79.9(71.7,86.4) | 80.1(72.1,86.6) |
| Hebei | 130623 | Laishui County | 6.3 | 5.5 | 7.2 |  | 79.0(68.4,87.3) | 82.4(71.3,91.0) | 74.5(63.6,83.6) |
| Hebei | 130702 | Qiaodong District | 5.4 | 4.4 | 6.2 |  | 78.7(62.5,91.7) | 83.0(63.8,95.2) | 68.8(50.1,87.5) |
| Hebei | 130521 | Xingtai County | 6.5 | 5.7 | 7.3 |  | 78.6(70.3,85.6) | 77.8(68.9,85.1) | 81.3(72.9,87.7) |
| Hebei | 130722 | Zhangbei County | 7.5 | 5.6 | 9.5 |  | 78.6(68.0,87.3) | 80.3(69.5,89.2) | 73.9(59.9,86.1) |
| Hebei | 130923 | Dongguang County | 6.3 | 5.2 | 7.4 |  | 78.6(69.0,86.0) | 77.4(67.2,86.0) | 81.7(71.1,89.0) |
| Hebei | 131024 | Xianghe County | 6.4 | 5.5 | 7.3 |  | 78.3(67.6,86.4) | 78.0(66.5,87.0) | 78.2(65.2,88.1) |
| Hebei | 130126 | Lingshou County | 6.4 | 5.4 | 7.4 |  | 78.0(69.2,85.2) | 75.5(65.4,83.3) | 82.3(71.8,89.7) |
| Hebei | 130922 | Qing County | 6.4 | 5.2 | 7.6 |  | 77.9(67.9,86.0) | 77.8(67.4,85.8) | 79.3(69.3,87.3) |
| Hebei | 131081 | Bazhou City | 6.4 | 5.5 | 7.3 |  | 77.8(64.8,87.3) | 77.6(64.5,86.9) | 80.2(67.2,89.3) |
| Hebei | 130406 | Fengfengkuang District | 7.2 | 6.5 | 7.8 |  | 77.5(67.9,85.0) | 73.3(62.7,82.7) | 82.1(71.4,90.0) |
| Hebei | 130227 | Qianxi County | 6.5 | 5.6 | 7.4 |  | 77.3(68.2,85.3) | 76.4(66.0,84.7) | 79.7(70.6,87.4) |
| Hebei | 130983 | Huanghua City | 6.1 | 5.1 | 7.1 |  | 77.3(67.4,85.6) | 78.3(67.3,87.0) | 76.8(66.7,86.1) |
| Hebei | 130108 | Yuhua District | 3.1 | 2.5 | 3.8 |  | 77.1(60.9,89.6) | 82.2(64.2,94.5) | 68.0(50.8,84.4) |
| Hebei | 130423 | Linzhang County | 6.1 | 4.8 | 7.4 |  | 76.8(67.3,84.0) | 78.7(69.3,85.5) | 77.6(67.7,85.3) |
| Hebei | 130681 | Zhuozhou City | 6.2 | 5.1 | 7.2 |  | 76.6(66.2,84.4) | 75.9(64.8,83.6) | 80.1(69.1,87.7) |
| Hebei | 130123 | Zhengding County | 6.4 | 5.3 | 7.5 |  | 76.5(67.8,83.7) | 79.6(70.1,86.5) | 73.4(63.7,81.9) |
| Hebei | 130621 | Mancheng County | 6.5 | 5.6 | 7.4 |  | 76.5(63.0,87.1) | 76.3(62.7,87.6) | 79.0(65.3,90.2) |
| Hebei | 130981 | Botou City | 6.0 | 5.1 | 7.0 |  | 76.5(66.7,83.5) | 77.5(68.2,85.6) | 76.7(65.8,84.5) |
| Hebei | 130184 | Xinle City | 6.2 | 5.0 | 7.3 |  | 76.4(61.6,87.9) | 78.7(62.2,89.4) | 75.2(60.0,87.2) |
| Hebei | 130433 | Guantao County | 6.3 | 5.3 | 7.3 |  | 76.3(66.7,83.9) | 75.9(66.4,84.0) | 80.4(71.3,88.1) |
| Hebei | 130635 | Li County | 5.6 | 4.7 | 6.5 |  | 76.1(66.6,83.6) | 75.6(65.2,83.3) | 78.7(68.5,86.8) |
| Hebei | 130581 | Nangong City | 6.2 | 5.7 | 6.8 |  | 76.0(67.0,83.3) | 76.9(67.3,84.4) | 76.5(68.1,84.2) |
| Hebei | 130684 | Gaobeidian City | 5.4 | 4.6 | 6.1 |  | 75.8(59.7,88.9) | 74.4(56.4,89.1) | 80.3(65.1,91.3) |
| Hebei | 130925 | Yanshan County | 6.1 | 5.3 | 6.9 |  | 75.8(65.6,83.6) | 76.0(65.7,84.4) | 77.5(67.9,85.8) |
| Hebei | 130281 | Zunhua City | 6.7 | 5.8 | 7.6 |  | 75.7(66.1,83.2) | 74.3(64.7,81.9) | 81.0(71.5,87.8) |
| Hebei | 130624 | Fuping County | 6.6 | 5.2 | 8.1 |  | 75.7(59.5,88.1) | 75.7(59.7,87.8) | 77.5(60.3,90.0) |
| Hebei | 130628 | Gaoyang County | 5.8 | 4.9 | 6.6 |  | 75.7(65.7,84.7) | 76.4(65.3,85.9) | 78.1(68.0,86.3) |
| Hebei | 130634 | Quyang County | 6.1 | 5.0 | 7.1 |  | 75.6(66.2,82.9) | 76.0(67.1,84.2) | 78.2(68.8,85.4) |
| Hebei | 130209 | Caofeidian District | 5.6 | 5.8 | 5.4 |  | 75.4(60.1,89.3) | 62.0(50.0,72.1) | 88.2(72.7,98.4) |
| Hebei | 130631 | Wangdu County | 6.6 | 5.4 | 7.8 |  | 75.1(64.1,83.7) | 75.8(64.9,84.4) | 75.1(62.2,85.6) |
| Hebei | 130403 | Congtai District | 6.4 | 5.4 | 7.3 |  | 74.8(59.6,87.7) | 76.6(60.4,89.3) | 73.8(58.6,87.4) |
| Hebei | 130434 | Wei County | 6.1 | 5.3 | 6.9 |  | 74.6(66.0,82.1) | 74.7(66.0,82.4) | 78.1(69.1,85.4) |
| Hebei | 130825 | Longhua County | 6.0 | 4.7 | 7.3 |  | 74.4(64.3,82.6) | 76.1(66.1,85.6) | 72.4(60.8,83.1) |
| Hebei | 130129 | Zanhuang County | 6.3 | 5.8 | 6.9 |  | 74.3(62.8,83.5) | 73.4(61.2,83.5) | 75.5(64.4,84.7) |
| Hebei | 130821 | Chengde County | 6.4 | 5.4 | 7.3 |  | 74.3(65.1,82.3) | 74.1(63.5,82.6) | 75.3(64.2,84.2) |
| Hebei | 130682 | Dingzhou City | 6.1 | 5.1 | 7.1 |  | 74.0(65.0,82.1) | 73.3(63.6,81.7) | 77.5(68.4,84.9) |
| Hebei | 131025 | Dacheng County | 6.5 | 5.5 | 7.5 |  | 73.8(60.1,84.2) | 74.0(61.5,84.6) | 76.1(63.3,86.4) |
| Hebei | 130528 | Ningjin County | 6.5 | 5.4 | 7.5 |  | 73.7(64.7,81.2) | 75.0(65.8,82.1) | 74.5(65.1,82.1) |
| Hebei | 130203 | Lubei District | 4.7 | 3.9 | 5.6 |  | 73.4(60.7,85.9) | 74.0(59.7,85.7) | 73.7(59.6,87.2) |
| Hebei | 130202 | Lunan District | 4.3 | 3.7 | 4.8 |  | 73.0(53.8,90.0) | 72.5(53.1,89.9) | 72.6(51.9,90.6) |
| Hebei | 130128 | Shenze County | 6.9 | 6.3 | 7.5 |  | 72.7(55.4,86.8) | 71.4(53.3,85.9) | 75.0(56.0,89.4) |
| Hebei | 130224 | Luannan County | 6.1 | 5.5 | 6.8 |  | 72.7(61.9,81.4) | 69.9(59.3,79.3) | 78.4(68.1,86.5) |
| Hebei | 130924 | Haixing County | 6.5 | 5.1 | 8.0 |  | 72.7(61.5,82.7) | 75.4(64.1,85.4) | 70.6(57.2,82.6) |
| Hebei | 130424 | Cheng'an County | 5.8 | 5.0 | 6.8 |  | 72.3(61.4,81.7) | 72.7(62.5,81.4) | 75.9(66.2,84.4) |
| Hebei | 130822 | Xinglong County | 5.4 | 4.1 | 6.6 |  | 72.0(61.2,81.0) | 75.9(65.2,84.8) | 68.9(59.0,79.3) |
| Hebei | 130125 | Xingtang County | 6.5 | 5.5 | 7.5 |  | 71.7(62.3,79.3) | 73.1(63.7,81.5) | 71.7(61.2,80.2) |
| Hebei | 130826 | Zizhixian | 6.1 | 4.3 | 7.8 |  | 71.7(60.5,80.7) | 77.9(68.4,85.9) | 62.7(52.2,73.6) |
| Hebei | 130182 | Gaocheng City | 6.0 | 5.2 | 6.8 |  | 71.2(54.4,84.9) | 70.4(54.5,83.6) | 76.5(58.0,89.2) |
| Hebei | 130130 | Wuji County | 6.3 | 5.4 | 7.2 |  | 71.1(61.0,78.7) | 72.1(62.2,80.1) | 71.5(61.4,80.3) |
| Hebei | 130733 | Chongli County | 6.7 | 5.2 | 8.1 |  | 71.1(55.3,85.3) | 69.4(52.9,85.7) | 71.8(51.8,90.9) |
| Hebei | 130630 | Laiyuan County | 4.9 | 4.0 | 5.7 |  | 70.7(57.9,82.3) | 72.9(58.1,86.6) | 68.1(56.0,80.7) |
| Hebei | 130929 | Xian County | 5.7 | 4.7 | 6.8 |  | 70.5(61.0,78.6) | 69.5(60.4,77.6) | 75.6(64.8,84.1) |
| Hebei | 130429 | Yongnian County | 5.8 | 5.2 | 6.5 |  | 70.4(61.1,78.6) | 68.9(59.7,76.9) | 76.2(67.8,83.5) |
| Hebei | 130637 | Boye County | 6.2 | 4.9 | 7.4 |  | 70.2(59.6,78.6) | 73.5(63.2,82.1) | 69.6(58.2,79.3) |
| Hebei | 130428 | Feixiang County | 6.4 | 5.8 | 7.1 |  | 69.9(60.0,79.0) | 68.9(58.3,77.7) | 74.6(64.6,83.2) |
| Hebei | 130133 | Zhao County | 6.3 | 5.5 | 7.2 |  | 69.6(54.0,82.4) | 67.3(51.3,81.3) | 74.4(58.2,86.3) |
| Hebei | 130984 | Hejian City | 6.2 | 5.2 | 7.3 |  | 69.4(59.8,78.1) | 70.7(60.8,78.9) | 71.0(60.7,79.5) |
| Hebei | 130204 | Dongkuang District | 6.1 | 5.4 | 6.8 |  | 69.3(57.7,80.9) | 72.2(58.5,85.5) | 68.4(57.2,78.7) |
| Hebei | 130402 | Hanshan District | 5.9 | 5.2 | 6.7 |  | 69.3(52.4,83.4) | 67.6(51.0,82.6) | 72.7(55.5,86.7) |
| Hebei | 130185 | Luquan City | 6.0 | 5.1 | 6.9 |  | 69.2(51.3,84.8) | 65.1(47.1,79.8) | 76.3(55.4,91.4) |
| Hebei | 130726 | Yu County | 5.5 | 4.4 | 6.5 |  | 69.1(59.6,78.1) | 67.5(55.9,77.7) | 72.9(63.2,82.3) |
| Hebei | 130229 | Yutian County | 6.3 | 5.3 | 7.2 |  | 69.0(59.8,77.2) | 67.3(57.9,76.3) | 73.4(63.5,81.4) |
| Hebei | 130927 | Nanpi County | 5.1 | 4.2 | 6.2 |  | 68.9(57.6,79.5) | 67.5(56.5,78.0) | 72.2(58.2,84.8) |
| Hebei | 131022 | Gu'an County | 6.4 | 5.6 | 7.2 |  | 68.6(53.4,82.0) | 66.7(51.4,80.2) | 73.9(58.7,86.3) |
| Hebei | 130127 | Gaoyi County | 6.0 | 5.4 | 6.6 |  | 68.4(58.4,78.6) | 67.6(55.4,77.6) | 72.6(60.0,83.1) |
| Hebei | 130324 | Lulong County | 6.8 | 5.8 | 7.8 |  | 68.1(55.7,77.8) | 66.1(54.7,76.6) | 71.3(59.2,81.5) |
| Hebei | 130627 | Tang County | 6.1 | 5.1 | 7.1 |  | 67.7(58.1,77.3) | 71.5(61.6,79.8) | 66.3(55.3,75.3) |
| Hebei | 130132 | Yuanshi County | 6.3 | 5.5 | 7.2 |  | 67.5(58.0,75.6) | 66.5(57.0,75.1) | 71.9(61.6,80.8) |
| Hebei | 130802 | Shuangqiao District | 5.0 | 4.2 | 5.9 |  | 67.2(55.3,78.0) | 64.6(53.6,75.2) | 73.3(58.8,85.3) |
| Hebei | 130982 | Renqiu City | 5.3 | 4.3 | 6.4 |  | 67.2(55.8,76.6) | 68.1(56.8,77.3) | 68.4(56.5,78.8) |
| Hebei | 130522 | Lincheng County | 4.9 | 4.2 | 5.6 |  | 66.9(53.8,80.1) | 63.5(50.3,76.3) | 72.8(56.1,86.9) |
| Hebei | 130683 | Anguo City | 5.9 | 4.9 | 6.8 |  | 66.9(56.0,76.3) | 67.2(55.6,78.4) | 69.2(57.0,79.6) |
| Hebei | 130926 | Suning County | 5.5 | 4.6 | 6.3 |  | 66.6(56.2,77.4) | 67.9(56.5,78.6) | 68.4(56.5,79.3) |
| Hebei | 130426 | She County | 6.1 | 5.2 | 7.0 |  | 65.8(55.6,74.9) | 61.9(50.4,72.3) | 72.1(60.3,81.9) |
| Hebei | 130828 | Mongolzu Zizhixian | 5.5 | 4.3 | 6.6 |  | 65.8(54.4,75.3) | 66.4(55.9,76.2) | 66.9(54.9,79.9) |
| Hebei | 130435 | Quzhou County | 6.2 | 5.4 | 7.0 |  | 65.1(54.0,74.6) | 66.2(56.0,74.9) | 67.2(57.2,76.3) |
| Hebei | 130532 | Pingxiang County | 6.2 | 5.6 | 6.9 |  | 65.0(53.7,75.2) | 62.9(51.5,73.1) | 71.9(59.9,81.3) |
| Hebei | 130525 | Longyao County | 6.3 | 5.0 | 7.5 |  | 64.8(54.8,73.9) | 67.8(58.5,75.8) | 63.1(52.9,72.8) |
| Hebei | 130225 | Leting County | 6.3 | 5.6 | 7.0 |  | 64.6(53.5,74.8) | 60.9(48.3,71.9) | 70.2(58.8,81.1) |
| Hebei | 130636 | Shunping County | 5.5 | 4.7 | 6.3 |  | 64.4(54.0,74.1) | 64.7(54.2,74.9) | 68.3(55.0,80.0) |
| Hebei | 130928 | Wuqiao County | 6.2 | 4.7 | 7.8 |  | 64.4(53.0,74.8) | 72.1(60.2,82.3) | 55.5(44.7,65.9) |
| Hebei | 131121 | Zaoqiang County | 6.6 | 5.8 | 7.4 |  | 63.3(53.6,72.3) | 60.9(51.4,70.2) | 69.1(58.4,78.6) |
| Hebei | 130183 | Jinzhou City | 6.5 | 5.8 | 7.2 |  | 61.8(48.1,74.9) | 61.7(47.6,74.9) | 64.9(49.9,77.8) |
| Hebei | 131002 | Anci District | 4.7 | 3.8 | 5.5 |  | 61.1(49.5,71.2) | 64.3(53.2,76.2) | 59.9(49.6,70.9) |
| Hebei | 130930 | Zizhixian | 4.8 | 4.3 | 5.3 |  | 60.0(48.7,71.7) | 56.5(45.6,67.1) | 69.2(55.1,80.9) |
| Hebei | 131127 | Jing County | 6.4 | 5.6 | 7.2 |  | 58.6(42.9,73.5) | 57.4(40.7,72.4) | 63.5(48.5,78.5) |
| Hebei | 131181 | Jizhou City | 6.2 | 5.7 | 6.7 |  | 58.3(47.7,68.4) | 48.8(38.8,58.9) | 71.2(58.5,82.3) |
| Hebei | 130181 | Xinji City | 6.1 | 5.3 | 6.9 |  | 57.2(40.3,72.1) | 59.8(42.9,74.4) | 57.8(41.3,72.7) |
| Hebei | 131126 | Gucheng County | 5.2 | 4.6 | 5.9 |  | 56.8(47.6,67.2) | 55.2(44.1,65.2) | 63.4(52.1,72.7) |
| Hebei | 130705 | Xuanhua District | 5.2 | 4.2 | 6.2 |  | 56.4(43.3,70.3) | 60.1(44.7,74.1) | 53.1(40.9,67.2) |
| Hebei | 130323 | Funing County | 4.2 | 3.5 | 4.8 |  | 53.3(42.1,65.5) | 52.2(41.4,64.2) | 58.8(45.2,72.1) |
| Hebei | 131003 | Guangyang District | 3.6 | 2.8 | 4.4 |  | 51.9(34.6,72.7) | 56.7(37.9,76.0) | 51.7(33.3,70.9) |
| Hebei | 130121 | Jingxing County | 6.5 | 5.8 | 7.1 |  | 51.5(41.2,62.5) | 49.7(38.1,61.3) | 54.5(42.3,65.5) |
| Hebei | 130531 | Guangzong County | 6.3 | 5.2 | 7.5 |  | 50.9(40.3,61.3) | 55.2(44.6,65.7) | 47.9(37.9,59.0) |
| Hebei | 131182 | Shenzhou City | 6.1 | 5.6 | 6.6 |  | 48.9(39.0,59.3) | 46.5(37.1,57.0) | 54.2(43.0,64.6) |
| Hebei | 130107 | Jingxingkuang District | 6.2 | 5.5 | 6.9 |  | 48.5(38.3,60.9) | 49.9(38.6,62.3) | 48.9(35.1,61.6) |
| Hebei | 130729 | Wanquan County | 5.0 | 3.9 | 6.0 |  | 47.1(31.7,63.0) | 47.6(32.1,65.4) | 50.9(34.4,67.7) |
| Hebei | 130208 | Fengrun District | 3.6 | 3.1 | 4.0 |  | 45.2(35.0,56.1) | 42.8(32.6,54.7) | 53.4(41.1,64.8) |
| Hebei | 130427 | Ci County | 3.8 | 3.2 | 4.3 |  | 42.7(33.8,52.0) | 43.9(34.2,54.0) | 46.0(36.4,55.9) |
| Hebei | 130731 | Zhuolu County | 3.9 | 3.0 | 4.7 |  | 42.4(26.9,61.3) | 45.9(28.3,66.6) | 40.0(25.9,55.5) |
| Hebei | 130706 | Xiahuayuan District | 5.3 | 4.4 | 6.1 |  | 41.5(29.6,53.4) | 43.8(31.8,57.4) | 39.7(27.0,53.8) |
| Hebei | 131125 | Anping County | 5.0 | 4.2 | 5.7 |  | 40.9(31.7,51.8) | 43.4(33.5,54.2) | 39.7(29.8,50.7) |
| Hebei | 130732 | Chicheng County | 3.5 | 2.4 | 4.5 |  | 40.2(29.4,54.4) | 45.7(32.9,62.3) | 35.2(26.2,45.6) |
| Hebei | 130823 | Pingquan County | 4.3 | 3.5 | 5.0 |  | 39.7(30.0,49.5) | 38.6(30.2,47.9) | 44.7(33.9,57.8) |
| Hebei | 130524 | Baixiang County | 4.4 | 3.9 | 4.8 |  | 37.8(28.6,47.9) | 38.3(28.7,49.7) | 40.3(30.3,51.1) |
| Hebei | 130727 | Yangyuan County | 3.6 | 2.8 | 4.4 |  | 37.4(26.8,50.1) | 40.4(27.6,55.2) | 36.7(27.1,47.8) |
| Hebei | 130530 | Xinhe County | 3.2 | 2.6 | 3.8 |  | 35.3(25.5,47.5) | 35.1(26.1,45.9) | 39.6(26.7,59.7) |
| Hebei | 131124 | Raoyang County | 5.2 | 4.5 | 6.0 |  | 33.8(21.7,48.0) | 36.0(22.5,52.1) | 32.1(20.2,44.7) |
| Hebei | 130724 | Guyuan County | 3.1 | 2.4 | 3.8 |  | 33.4(22.7,48.5) | 36.7(23.6,55.4) | 30.7(21.4,46.9) |
| Hebei | 130321 | Zizhixian | 4.2 | 3.7 | 4.7 |  | 31.5(23.3,40.6) | 30.7(23.0,40.7) | 35.3(26.8,45.5) |
| Hebei | 130322 | Changli County | 2.0 | 1.5 | 2.6 |  | 28.2(18.7,42.1) | 32.9(21.9,49.0) | 26.0(17.9,36.5) |
| Hebei | 130723 | Kangbao County | 4.1 | 3.0 | 5.2 |  | 26.6(19.2,36.3) | 30.5(21.4,43.2) | 23.3(16.5,31.5) |
| Hebei | 131122 | Wuyi County | 3.6 | 3.0 | 4.2 |  | 24.8(18.4,32.9) | 25.6(18.3,34.0) | 27.0(19.9,35.9) |
| Hebei | 130730 | Huailai County | 3.3 | 2.6 | 4.0 |  | 24.5(18.6,31.7) | 25.0(18.6,32.4) | 27.3(20.2,35.4) |
| Hebei | 131102 | Taocheng District | 1.8 | 1.2 | 2.3 |  | 21.5(15.3,28.8) | 23.4(16.5,32.6) | 22.4(15.6,31.0) |
| Hebei | 131128 | Fucheng County | 2.4 | 2.0 | 2.8 |  | 20.4(14.2,27.8) | 19.2(13.2,26.5) | 24.6(17.3,34.8) |
| Hebei | 131123 | Wuqiang County | 2.7 | 2.2 | 3.3 |  | 16.7(11.8,22.6) | 18.2(12.9,24.1) | 17.4(12.0,24.6) |
| Hebei | 130728 | Huai'an County | 2.9 | 2.1 | 3.8 |  | 16.5(11.2,24.5) | 16.7(10.7,25.1) | 17.1(11.2,26.1) |
| Hebei | 130725 | Shangyi County | 1.9 | 1.3 | 2.5 |  | 14.4(9.1,23.6) | 15.9(9.7,29.0) | 13.8(8.9,20.7) |
| Inner Mongolia | 150802 | Linhe District | 5.9 | 4.6 | 7.0 |  | 95.0(89.9,98.1) | 91.1(83.3,96.4) | 97.0(90.8,99.3) |
| Inner Mongolia | 150781 | Manzhouli City | 9.2 | 7.5 | 10.9 |  | 94.8(86.3,98.8) | 89.6(75.5,97.2) | 97.1(86.6,99.8) |
| Inner Mongolia | 150703 | Hailar District | 6.7 | 5.6 | 7.8 |  | 93.2(76.5,98.9) | 91.3(70.2,99.2) | 93.4(71.3,99.5) |
| Inner Mongolia | 150423 | Bairin Youqi | 6.2 | 4.7 | 7.5 |  | 92.8(84.5,97.5) | 95.3(88.1,98.8) | 82.2(66.4,93.0) |
| Inner Mongolia | 150302 | Haibowan District | 5.5 | 4.1 | 6.8 |  | 92.6(83.5,97.3) | 87.3(77.2,94.9) | 95.7(84.9,99.3) |
| Inner Mongolia | 150627 | Ejin Horo Qi | 5.2 | 4.5 | 5.7 |  | 91.7(83.1,97.3) | 92.9(81.5,98.5) | 89.8(78.2,96.7) |
| Inner Mongolia | 150523 | Kailu County | 6.5 | 5.5 | 7.6 |  | 90.8(82.9,95.6) | 89.2(80.5,95.0) | 92.0(82.9,97.1) |
| Inner Mongolia | 150402 | Hongshan District | 3.6 | 2.9 | 4.2 |  | 90.6(77.7,97.4) | 90.1(74.1,97.5) | 86.7(65.6,97.1) |
| Inner Mongolia | 150404 | Songshan District | 4.6 | 3.8 | 5.3 |  | 90.5(83.0,95.4) | 88.3(78.9,94.4) | 91.6(81.9,97.2) |
| Inner Mongolia | 150602 | Dongsheng District | 3.4 | 2.6 | 4.1 |  | 90.0(81.8,95.4) | 92.7(84.4,97.2) | 85.1(73.7,93.1) |
| Inner Mongolia | 150502 | Horqin District | 5.3 | 4.5 | 6.0 |  | 89.8(82.7,94.4) | 86.8(79.2,92.7) | 92.9(85.8,97.5) |
| Inner Mongolia | 150926 | Qahar Youyi Qianqi | 8.3 | 6.1 | 10.5 |  | 87.9(70.8,96.8) | 73.7(63.1,82.1) | 93.4(68.2,99.5) |
| Inner Mongolia | 150403 | Yuanbaoshan District | 7.0 | 5.8 | 8.2 |  | 86.7(76.7,93.8) | 78.5(68.6,86.4) | 93.8(83.0,98.7) |
| Inner Mongolia | 150726 | Xin Barag Zuoqi | 7.9 | 6.4 | 9.3 |  | 86.7(73.0,95.4) | 85.7(71.8,95.5) | 86.0(64.5,98.8) |
| Inner Mongolia | 150426 | Ongniud Qi | 5.9 | 4.7 | 7.1 |  | 85.7(76.1,92.6) | 84.1(74.7,91.5) | 86.5(74.5,94.9) |
| Inner Mongolia | 150425 | Hexigten Qi | 6.6 | 5.8 | 7.4 |  | 85.1(74.3,92.6) | 81.0(68.8,90.1) | 89.2(77.1,96.4) |
| Inner Mongolia | 152525 | Dong Ujimqin Qi | 5.2 | 4.5 | 5.9 |  | 84.8(67.1,95.5) | 80.6(60.2,95.2) | 88.1(67.7,98.2) |
| Inner Mongolia | 150724 | Ewenkizu Zizhiqi | 7.2 | 6.0 | 8.4 |  | 84.4(71.4,93.4) | 79.5(66.1,90.3) | 88.4(71.6,97.4) |
| Inner Mongolia | 150221 | Tumd Youqi | 7.2 | 6.3 | 8.1 |  | 84.3(75.0,91.7) | 84.0(72.3,91.4) | 83.7(72.5,91.0) |
| Inner Mongolia | 152502 | Xilinhot City | 4.4 | 3.4 | 5.3 |  | 83.9(68.3,94.4) | 86.3(68.6,96.1) | 81.4(62.8,94.7) |
| Inner Mongolia | 150525 | Naiman Qi | 6.2 | 5.0 | 7.4 |  | 83.4(75.1,89.4) | 86.1(77.6,92.3) | 78.6(68.0,87.5) |
| Inner Mongolia | 152921 | Alxa Zuoqi | 4.3 | 3.8 | 4.8 |  | 82.2(64.5,93.6) | 79.5(57.9,93.2) | 83.2(61.6,96.4) |
| Inner Mongolia | 150581 | Holingol City | 3.8 | 2.8 | 4.8 |  | 82.1(64.8,94.0) | 90.3(71.0,98.3) | 59.8(43.3,79.7) |
| Inner Mongolia | 150821 | Wuyuan County | 6.6 | 5.7 | 7.6 |  | 82.0(70.9,90.3) | 76.1(63.8,87.1) | 87.3(75.2,95.1) |
| Inner Mongolia | 150430 | Aohan Qi | 5.7 | 4.5 | 7.0 |  | 81.6(72.6,88.7) | 80.5(71.6,88.3) | 82.9(70.8,91.9) |
| Inner Mongolia | 150205 | Shiguai District | 4.6 | 3.7 | 5.3 |  | 81.5(22.5,100.0) | 90.3(26.1,100.0) | 25.9(14.8,41.9) |
| Inner Mongolia | 150722 | Zizhiqi | 6.1 | 4.7 | 7.5 |  | 81.3(72.8,88.0) | 82.6(73.0,89.7) | 80.7(70.1,88.6) |
| Inner Mongolia | 150822 | Dengkou County | 6.5 | 5.4 | 7.5 |  | 81.0(62.6,94.6) | 75.7(56.6,92.4) | 84.3(59.3,98.0) |
| Inner Mongolia | 150303 | Hainan District | 4.1 | 3.2 | 4.8 |  | 80.1(58.6,94.4) | 86.5(59.8,98.4) | 66.7(45.9,90.4) |
| Inner Mongolia | 150524 | Hure Qi | 6.6 | 5.1 | 7.9 |  | 80.1(69.1,88.5) | 83.9(72.5,92.0) | 73.0(60.2,84.3) |
| Inner Mongolia | 150783 | Zalantun City | 7.6 | 5.8 | 9.3 |  | 79.9(71.0,86.8) | 78.2(69.3,85.6) | 80.5(69.2,89.3) |
| Inner Mongolia | 152527 | Taibus Qi | 9.8 | 7.4 | 12.2 |  | 79.9(69.4,89.6) | 74.2(63.5,82.6) | 81.0(64.2,93.8) |
| Inner Mongolia | 150521 | Horqin Zuoyi Zhongqi | 5.7 | 4.5 | 6.9 |  | 79.4(71.0,86.3) | 81.1(72.5,88.6) | 78.8(67.9,86.6) |
| Inner Mongolia | 150702 | Hailar District | 5.4 | 4.7 | 6.0 |  | 79.0(65.3,90.6) | 86.5(70.4,96.1) | 65.1(54.5,74.7) |
| Inner Mongolia | 150204 | Qingshan District | 4.8 | 3.8 | 5.8 |  | 78.9(63.2,90.6) | 75.4(59.8,88.6) | 78.8(57.8,93.0) |
| Inner Mongolia | 150721 | Arun Qi | 6.5 | 4.9 | 7.9 |  | 78.4(68.7,85.5) | 75.8(67.0,84.0) | 82.8(71.2,91.1) |
| Inner Mongolia | 150826 | Hanggin Houqi | 5.7 | 4.5 | 6.7 |  | 77.8(64.9,88.9) | 67.7(54.5,79.6) | 86.5(70.0,97.1) |
| Inner Mongolia | 150125 | Wuchuan County | 9.9 | 8.8 | 10.9 |  | 77.5(66.1,86.7) | 69.5(57.4,81.2) | 84.2(71.9,92.9) |
| Inner Mongolia | 150625 | Hanggin Qi | 5.1 | 4.2 | 6.0 |  | 77.2(58.6,93.1) | 82.5(58.0,96.6) | 66.3(47.3,87.2) |
| Inner Mongolia | 150725 | Chen Barag Qi | 7.2 | 5.6 | 8.6 |  | 77.2(61.8,89.4) | 69.8(58.3,79.1) | 83.2(56.5,97.6) |
| Inner Mongolia | 150526 | Jarud Qi | 5.0 | 3.8 | 6.2 |  | 76.6(52.9,93.1) | 78.6(56.5,93.8) | 78.4(53.0,95.4) |
| Inner Mongolia | 150727 | Xin Barag Youqi | 7.8 | 6.4 | 9.0 |  | 75.9(63.7,84.8) | 77.0(64.2,85.8) | 74.6(58.2,85.9) |
| Inner Mongolia | 150428 | Harqin Qi | 7.2 | 5.6 | 8.6 |  | 75.5(66.1,83.3) | 75.1(66.1,83.6) | 76.2(64.0,86.4) |
| Inner Mongolia | 150624 | Otog Qi | 3.2 | 2.7 | 3.7 |  | 74.6(57.3,89.6) | 71.5(50.7,88.7) | 78.6(58.2,93.8) |
| Inner Mongolia | 152523 | Sonid Zuoqi | 5.3 | 4.5 | 6.0 |  | 73.6(48.6,93.8) | 55.0(39.8,69.3) | 90.2(51.4,99.6) |
| Inner Mongolia | 150429 | Ningcheng County | 6.3 | 5.4 | 7.2 |  | 72.5(63.4,80.2) | 71.8(61.3,80.0) | 75.3(65.6,83.0) |
| Inner Mongolia | 150304 | Ud District | 4.5 | 3.6 | 5.3 |  | 71.2(49.0,89.8) | 78.1(50.6,96.3) | 58.7(40.3,84.8) |
| Inner Mongolia | 150422 | Bairin Zuoqi | 5.7 | 4.6 | 6.9 |  | 70.9(59.6,79.6) | 70.7(59.2,80.8) | 72.0(60.1,83.4) |
| Inner Mongolia | 152530 | Zhenglan Qi | 5.8 | 4.9 | 6.6 |  | 70.5(53.9,86.7) | 63.7(49.0,80.0) | 78.7(55.8,95.4) |
| Inner Mongolia | 152922 | Alxa Youqi | 4.9 | 4.6 | 5.3 |  | 70.0(36.8,97.0) | 80.9(36.4,99.8) | 51.1(32.7,68.5) |
| Inner Mongolia | 152526 | Xi Ujimqin Qi | 5.3 | 4.0 | 6.3 |  | 68.6(55.4,81.0) | 73.1(58.0,87.9) | 63.2(51.1,75.2) |
| Inner Mongolia | 152522 | Abag Qi | 5.8 | 4.9 | 6.6 |  | 68.1(49.9,88.8) | 72.1(49.1,94.4) | 61.2(45.0,74.7) |
| Inner Mongolia | 152528 | Xianghuang Qi | 6.4 | 4.9 | 7.8 |  | 67.8(48.6,88.7) | 75.4(50.6,95.8) | 55.8(38.7,71.9) |
| Inner Mongolia | 150623 | Otog Qianqi | 5.3 | 4.9 | 5.7 |  | 67.7(50.6,84.6) | 59.0(42.5,79.9) | 77.5(54.6,94.1) |
| Inner Mongolia | 152531 | Duolun County | 5.3 | 4.0 | 6.5 |  | 66.7(48.2,81.2) | 69.1(51.5,83.1) | 67.2(47.3,82.1) |
| Inner Mongolia | 152222 | Horqin Youyi Zhongqi | 5.6 | 4.6 | 6.5 |  | 65.6(53.5,76.5) | 69.8(56.9,80.3) | 62.7(50.7,73.2) |
| Inner Mongolia | 150622 | Jungar Qi | 3.0 | 2.4 | 3.5 |  | 65.1(50.4,78.5) | 66.7(50.8,81.9) | 64.9(51.4,78.4) |
| Inner Mongolia | 150784 | Ergun City | 6.6 | 5.7 | 7.5 |  | 65.1(49.0,84.0) | 71.5(50.7,93.0) | 57.0(43.4,70.4) |
| Inner Mongolia | 152524 | Sonid Youqi | 6.1 | 4.6 | 7.5 |  | 64.1(48.9,81.2) | 71.1(53.3,89.9) | 54.2(41.7,67.5) |
| Inner Mongolia | 150424 | Linxi County | 5.4 | 3.7 | 7.1 |  | 63.9(50.6,76.6) | 73.7(59.2,86.0) | 51.0(37.7,67.0) |
| Inner Mongolia | 150824 | Urad Zhongqi | 4.3 | 3.5 | 5.1 |  | 63.9(46.8,80.8) | 64.7(48.7,85.1) | 61.7(43.0,84.8) |
| Inner Mongolia | 150421 | Ar Horqin Qi | 6.2 | 5.1 | 7.2 |  | 63.6(52.5,73.0) | 64.6(54.0,75.0) | 64.7(52.1,74.9) |
| Inner Mongolia | 150902 | Jining District | 1.0 | 0.7 | 1.4 |  | 63.0(38.1,87.8) | 42.5(26.4,73.6) | 82.9(44.9,98.9) |
| Inner Mongolia | 150823 | Urad Qianqi | 6.0 | 5.0 | 6.9 |  | 61.1(49.5,72.2) | 60.7(47.9,72.9) | 60.8(48.4,73.7) |
| Inner Mongolia | 150723 | Oroqen Zizhiqi | 7.8 | 5.8 | 9.8 |  | 60.3(47.3,72.6) | 65.3(52.7,77.2) | 49.3(35.4,66.9) |
| Inner Mongolia | 150626 | Uxin Qi | 3.6 | 2.9 | 4.2 |  | 59.8(43.1,78.0) | 69.2(48.2,87.2) | 47.0(33.4,63.9) |
| Inner Mongolia | 150782 | Yakeshi City | 8.0 | 6.9 | 9.0 |  | 59.4(47.7,70.7) | 65.6(51.7,77.9) | 47.1(36.8,57.9) |
| Inner Mongolia | 150825 | Urad Houqi | 4.0 | 3.2 | 4.8 |  | 58.9(40.2,82.3) | 67.7(42.6,94.3) | 51.3(39.0,65.2) |
| Inner Mongolia | 150202 | Donghe District | 5.0 | 4.2 | 5.7 |  | 58.1(35.9,80.2) | 61.3(41.2,84.6) | 52.5(31.7,74.7) |
| Inner Mongolia | 150522 | Horqin Zuoyi Houqi | 4.1 | 3.0 | 5.1 |  | 57.6(46.5,68.8) | 54.7(45.0,65.7) | 64.2(48.8,79.5) |
| Inner Mongolia | 150103 | Huimin District | 4.9 | 4.1 | 5.7 |  | 56.4(44.0,68.6) | 53.7(42.5,65.4) | 64.4(50.0,77.7) |
| Inner Mongolia | 150223 | Lianheqi | 4.6 | 3.7 | 5.5 |  | 55.5(44.7,66.5) | 55.9(44.7,68.9) | 58.9(46.3,70.6) |
| Inner Mongolia | 150929 | Dorbod Qi | 4.4 | 3.0 | 5.7 |  | 53.1(43.0,63.8) | 56.9(45.4,67.5) | 52.3(40.9,63.2) |
| Inner Mongolia | 150123 | Horinger County | 5.2 | 4.1 | 6.2 |  | 49.6(37.6,61.9) | 48.1(35.5,63.5) | 52.5(40.5,68.4) |
| Inner Mongolia | 150785 | Genhe City | 7.0 | 6.2 | 7.8 |  | 49.2(33.0,72.5) | 51.3(33.5,75.7) | 41.2(23.8,75.2) |
| Inner Mongolia | 150105 | Saihan District | 3.5 | 2.9 | 4.0 |  | 48.7(37.2,61.1) | 50.3(38.6,62.9) | 48.6(36.9,60.5) |
| Inner Mongolia | 150102 | Xincheng District | 3.5 | 2.9 | 4.1 |  | 47.3(27.3,70.1) | 45.4(26.6,69.3) | 51.3(29.4,79.2) |
| Inner Mongolia | 150121 | Tumd Zuoqi | 5.9 | 5.0 | 6.8 |  | 45.3(35.0,55.4) | 42.6(33.4,52.7) | 50.2(38.6,62.8) |
| Inner Mongolia | 150203 | Kundulun District | 3.6 | 2.9 | 4.1 |  | 44.4(34.4,54.5) | 42.7(33.0,54.7) | 49.6(38.8,62.0) |
| Inner Mongolia | 152923 | Ejin Qi | 3.0 | 3.1 | 3.0 |  | 44.4(27.4,77.2) | 31.2(20.5,43.9) | 65.2(35.8,95.7) |
| Inner Mongolia | 152529 | Zhengxiangbai Qi | 5.9 | 4.4 | 7.3 |  | 44.0(32.8,56.2) | 49.4(36.8,62.5) | 39.1(26.7,53.8) |
| Inner Mongolia | 150207 | Jiuyuan District | 2.7 | 2.3 | 3.0 |  | 43.9(32.6,57.5) | 39.1(28.8,51.9) | 54.0(38.9,72.4) |
| Inner Mongolia | 152201 | Ulan Hot City | 2.9 | 2.0 | 3.9 |  | 43.1(32.0,56.9) | 51.6(36.7,66.0) | 36.1(25.0,51.7) |
| Inner Mongolia | 150104 | Yuquan District | 2.9 | 2.4 | 3.4 |  | 41.7(32.3,52.3) | 40.2(29.9,51.5) | 48.3(36.7,62.2) |
| Inner Mongolia | 150621 | Dalad Qi | 3.0 | 2.3 | 3.8 |  | 37.0(28.1,47.6) | 34.7(26.2,45.9) | 44.4(32.2,60.3) |
| Inner Mongolia | 152501 | Erenhot City | 2.6 | 1.6 | 3.7 |  | 33.3(17.8,58.4) | 41.0(21.9,74.2) | 30.7(15.2,50.8) |
| Inner Mongolia | 150124 | Qingshuihe County | 5.0 | 4.1 | 5.7 |  | 31.6(22.9,41.7) | 29.1(19.9,39.8) | 37.7(27.5,50.4) |
| Inner Mongolia | 150206 | Baiyun Kuangqu | 2.6 | 2.0 | 3.1 |  | 31.1(21.5,43.7) | 33.4(21.8,49.2) | 34.1(22.2,49.2) |
| Inner Mongolia | 152223 | Jalaid Qi | 1.9 | 1.5 | 2.3 |  | 30.6(23.1,39.6) | 29.3(22.0,38.1) | 38.4(28.3,50.9) |
| Inner Mongolia | 150122 | Togtoh County | 4.0 | 2.9 | 4.8 |  | 27.1(19.7,36.1) | 27.5(20.2,37.6) | 27.7(19.7,38.8) |
| Inner Mongolia | 150925 | Liangcheng County | 2.3 | 1.7 | 2.9 |  | 24.9(16.3,39.1) | 27.5(16.6,47.1) | 24.6(17.1,34.3) |
| Inner Mongolia | 152202 | Arxan City | 2.4 | 2.2 | 2.6 |  | 21.6(11.5,39.4) | 25.1(11.9,45.6) | 19.9(8.8,38.3) |
| Inner Mongolia | 152224 | Tuquan County | 1.5 | 1.1 | 1.9 |  | 18.9(13.5,26.4) | 20.5(14.1,30.1) | 20.5(14.4,27.3) |
| Inner Mongolia | 150927 | Qahar Youyi Zhongqi | 2.1 | 1.7 | 2.4 |  | 17.7(10.6,34.0) | 18.3(9.5,46.2) | 18.3(12.4,25.7) |
| Inner Mongolia | 150222 | Guyang County | 1.0 | 0.6 | 1.3 |  | 16.2(9.9,28.3) | 12.3(8.4,17.9) | 23.9(10.8,54.0) |
| Inner Mongolia | 150923 | Shangdu County | 1.8 | 1.2 | 2.4 |  | 14.9(9.5,24.6) | 15.7(9.6,28.0) | 14.8(8.8,29.2) |
| Inner Mongolia | 150921 | Zhuozi County | 0.9 | 0.6 | 1.2 |  | 14.4(6.3,40.0) | 12.1(5.7,42.4) | 15.2(6.2,58.4) |
| Inner Mongolia | 150924 | Xinghe County | 1.6 | 1.2 | 2.0 |  | 14.0(9.8,20.0) | 14.0(9.3,20.0) | 16.4(11.6,22.9) |
| Inner Mongolia | 150928 | Qahar Youyi Houqi | 1.8 | 1.3 | 2.3 |  | 14.0(9.2,20.3) | 15(9.6,21.9) | 15.1(9.8,22.0) |
| Inner Mongolia | 150981 | Fengzhen District | 0.5 | 0.3 | 0.7 |  | 13.3(8,23.2) | 10.8(6.9,16.9) | 18.9(10.5,43.9) |
| Inner Mongolia | 152221 | Horqin Youyi Qianqi | 0.7 | 0.4 | 0.9 |  | 13.3(9.2,19.1) | 13.7(9.4,19.3) | 16.7(11.5,23.6) |
| Inner Mongolia | 150922 | Huade County | 1.0 | 0.7 | 1.4 |  | 4.2(2.6,6.4) | 4.4(2.7,7.0) | 4.5(3.0,7.0) |
| Shanxi | 140702 | Yuci District | 5.5 | 4.6 | 6.5 |  | 96.7(91.8,99.0) | 95.0(88.0,98.4) | 97.1(91.5,99.4) |
| Shanxi | 140802 | Yanhu District | 5.8 | 4.8 | 6.9 |  | 92.7(85.7,96.7) | 93.2(86.5,97.4) | 91.4(83.4,96.7) |
| Shanxi | 140431 | Qinyuan County | 6.8 | 5.9 | 7.6 |  | 92.5(84.4,97.5) | 86.2(73.8,94.5) | 96.4(87.7,99.4) |
| Shanxi | 140224 | Lingqiu County | 5.8 | 4.5 | 6.9 |  | 92.3(83.7,97.4) | 90.5(79.6,96.9) | 93.4(83.2,98.6) |
| Shanxi | 141021 | Quwo County | 6.2 | 5.6 | 6.8 |  | 92.2(84.2,97.0) | 89.5(79.7,96.1) | 93.4(83.3,98.1) |
| Shanxi | 140105 | Xiaodian District | 2.5 | 2.1 | 2.9 |  | 91.2(66.5,99.2) | 79.6(52.8,96.4) | 96.1(76.7,99.9) |
| Shanxi | 140502 | Cheng District | 2.7 | 2.0 | 3.5 |  | 90.5(74.6,98.1) | 89.2(70.8,97.8) | 91.3(75.8,98.7) |
| Shanxi | 140181 | Gujiao City | 3.5 | 2.5 | 4.4 |  | 89.4(71.1,97.6) | 89.9(71.4,98.2) | 84.5(60.1,97.1) |
| Shanxi | 140581 | Gaoping City | 6.2 | 5.6 | 6.9 |  | 87.9(78.3,94.3) | 82.7(70.9,91.8) | 92.7(84.8,97.3) |
| Shanxi | 141022 | Yicheng County | 6.1 | 4.8 | 7.3 |  | 87.1(77.8,93.5) | 89.2(79.7,95.7) | 83.2(71.1,92.0) |
| Shanxi | 141081 | Houma City | 5.7 | 4.7 | 6.8 |  | 86.9(72.6,96.1) | 86.0(70.3,95.8) | 86.9(69.2,97.0) |
| Shanxi | 140109 | Wanbailin District | 2.9 | 2.4 | 3.4 |  | 86.8(57.2,98.2) | 86.6(56.6,98.3) | 82.8(53.1,97.9) |
| Shanxi | 140521 | Qinshui County | 6.9 | 6.2 | 7.5 |  | 86.3(76.6,93.8) | 83.2(70.4,93.5) | 90.4(80.5,97.1) |
| Shanxi | 140827 | Yuanqu County | 5.3 | 4.7 | 5.9 |  | 85.2(74.3,92.7) | 77.9(67.0,88.3) | 91.5(80.6,97.8) |
| Shanxi | 140821 | Linyi County | 5.7 | 5.1 | 6.3 |  | 84.8(76.1,91.5) | 79.2(68.9,87.5) | 90.5(81.9,95.9) |
| Shanxi | 141024 | Hongdong County | 4.8 | 4.1 | 5.5 |  | 84.6(75.7,90.9) | 86.1(77.6,92.0) | 85.1(77.0,91.9) |
| Shanxi | 140303 | Kuang District | 4.1 | 2.7 | 5.4 |  | 84.4(67.8,95.7) | 85.5(65.7,96.5) | 81.4(58.0,96.8) |
| Shanxi | 140522 | Yangcheng County | 6.8 | 6.1 | 7.4 |  | 83.8(64.8,95.3) | 83.0(61.4,96.2) | 84.4(67.2,94.1) |
| Shanxi | 140524 | Lingchuan County | 7.1 | 6.1 | 8.0 |  | 83.6(73.3,90.9) | 80.7(70.1,89.4) | 88.0(78.4,94.6) |
| Shanxi | 140823 | Wenxi County | 5.3 | 4.8 | 5.8 |  | 83.6(72.9,91.9) | 78.5(66.2,89.4) | 88.3(75.8,95.9) |
| Shanxi | 140427 | Huguan County | 5.7 | 4.9 | 6.5 |  | 82.9(73.5,90.3) | 82.3(71.2,90.5) | 85.2(75.2,91.7) |
| Shanxi | 140881 | Yongji City | 6.2 | 5.7 | 6.7 |  | 82.7(74.5,89.5) | 81.9(72.0,89.9) | 84.5(74.7,91.0) |
| Shanxi | 140830 | Ruicheng County | 5.4 | 4.8 | 6.0 |  | 82.6(71.9,91.1) | 83.3(70.2,92.8) | 79.6(66.5,89.4) |
| Shanxi | 141023 | Xiangfen County | 5.7 | 5.2 | 6.3 |  | 82.3(71.3,90.8) | 80.3(67.5,89.8) | 84.3(71.2,93.3) |
| Shanxi | 140725 | Shouyang County | 7.0 | 6.2 | 7.7 |  | 80.8(67.4,91.4) | 64.6(48.4,79.3) | 91.7(79.5,97.9) |
| Shanxi | 140106 | Yingze District | 3.7 | 2.9 | 4.5 |  | 80.4(51.5,97.3) | 80.3(53.2,97.0) | 79.1(45.7,97.5) |
| Shanxi | 140121 | Qingxu County | 5.3 | 4.7 | 5.9 |  | 80.3(70.5,88.6) | 73.7(62.2,85.0) | 87.2(76.0,94.6) |
| Shanxi | 140421 | Changzhi County | 5.5 | 4.8 | 6.3 |  | 78.6(67.3,87.4) | 75.3(63.5,85.4) | 84.3(72.9,92.6) |
| Shanxi | 140424 | Tunliu County | 4.8 | 4.1 | 5.5 |  | 77.9(66.9,86.3) | 72.3(61.2,82.6) | 85.7(73.8,94.3) |
| Shanxi | 140426 | Licheng County | 7.3 | 5.4 | 8.9 |  | 77.8(66.2,87.0) | 76.9(66.0,86.0) | 79.3(66.0,90.2) |
| Shanxi | 141002 | Yaodu District | 3.9 | 3.3 | 4.6 |  | 77.7(54.7,93.1) | 81.6(56.5,96.4) | 74.0(53.6,89.3) |
| Shanxi | 141082 | Huozhou City | 4.1 | 3.3 | 4.8 |  | 77.5(55.7,93.3) | 74.9(55.5,90.7) | 83.2(60.2,97.6) |
| Shanxi | 140227 | Datong County | 6.4 | 5.1 | 7.6 |  | 76.7(65.9,85.8) | 74.8(65.0,82.4) | 81.4(68.4,92.0) |
| Shanxi | 141031 | Xi County | 4.8 | 4.0 | 5.6 |  | 75.8(61.1,87.9) | 70.8(59.6,80.7) | 83.8(66.1,96.4) |
| Shanxi | 140822 | Wanrong County | 6.1 | 5.4 | 6.9 |  | 75.5(64.5,85.1) | 73.8(62.2,83.3) | 77.8(65.2,87.7) |
| Shanxi | 140728 | Pingyao County | 5.2 | 4.6 | 5.7 |  | 75.3(65.1,84.3) | 67.6(55.0,79.1) | 83.5(72.3,91.9) |
| Shanxi | 140882 | Hejin City | 4.8 | 4.3 | 5.2 |  | 74.7(63.2,84.9) | 71.2(58.1,82.6) | 80.4(67.9,91.0) |
| Shanxi | 140321 | Pingding County | 5.9 | 4.9 | 6.8 |  | 74.5(54.7,90.2) | 71.8(52.4,87.3) | 81.0(59.6,95.2) |
| Shanxi | 140829 | Pinglu County | 5.4 | 4.8 | 5.9 |  | 74.5(60.2,85.7) | 75.1(60.9,88.3) | 73.4(60.0,84.8) |
| Shanxi | 140726 | Taigu County | 5.2 | 4.3 | 5.9 |  | 73.9(60.3,85.6) | 74.9(59.6,88.1) | 69.9(53.3,83.9) |
| Shanxi | 141029 | Xiangning County | 4.7 | 3.8 | 5.6 |  | 73.9(61.1,84.5) | 70.7(58.9,81.2) | 81.4(66.2,93.6) |
| Shanxi | 140825 | Xinjiang County | 5.8 | 5.2 | 6.3 |  | 73.2(62.8,82.3) | 71.0(59.2,81.4) | 77.5(65.5,87.4) |
| Shanxi | 140110 | Jinyuan District | 4.2 | 3.7 | 4.7 |  | 72.9(56.5,87.0) | 76.3(58.5,92.0) | 71.8(58.4,85.5) |
| Shanxi | 141032 | Yonghe County | 4.7 | 4.1 | 5.2 |  | 72.4(56.0,87.9) | 64.1(50.4,74.6) | 85.2(64.1,97.8) |
| Shanxi | 140602 | Shuocheng District | 3.0 | 2.3 | 3.6 |  | 72.3(57.1,86.1) | 73.4(57.7,87.6) | 73.5(58.1,86.4) |
| Shanxi | 141025 | Gu County | 4.9 | 4.4 | 5.5 |  | 71.6(53.0,88.2) | 71.8(51.1,90.0) | 74.8(57.2,87.3) |
| Shanxi | 141129 | Zhongyang County | 3.8 | 3.4 | 4.1 |  | 71.6(56.8,86.6) | 64.7(49.5,82.3) | 82.1(65.9,94.0) |
| Shanxi | 140824 | Jishan County | 6.1 | 5.3 | 6.9 |  | 71.1(59.4,80.9) | 73.2(61.1,83.5) | 70.1(57.8,80.3) |
| Shanxi | 141122 | Jiaocheng County | 3.8 | 3.4 | 4.1 |  | 71.0(55.5,83.6) | 67.9(52.0,82.3) | 76.3(63.5,87.9) |
| Shanxi | 140729 | Lingshi County | 4.4 | 4.0 | 4.8 |  | 70.3(57.4,81.2) | 66.1(51.7,77.5) | 76.2(61.0,87.8) |
| Shanxi | 140402 | Cheng District | 3.7 | 2.9 | 4.5 |  | 69.8(46.8,88.3) | 69.7(47.7,89.4) | 72.2(49.5,93.2) |
| Shanxi | 140902 | Xinfu District | 4.2 | 3.3 | 5.1 |  | 69.8(48.9,87.7) | 67.8(47.3,85.6) | 75.7(52.1,92.7) |
| Shanxi | 141026 | Anze County | 5.2 | 4.5 | 5.8 |  | 69.7(49.2,88.4) | 71.0(48.3,92.6) | 72.8(53.4,87.3) |
| Shanxi | 140981 | Yuanping City | 5.5 | 4.2 | 6.6 |  | 69.6(57.5,79.0) | 71.7(59.8,82.1) | 70.1(59.0,79.5) |
| Shanxi | 140425 | Pingshun County | 5.7 | 4.8 | 6.6 |  | 69.2(55.6,81.0) | 73.3(57.1,87.4) | 64.3(53.1,74.3) |
| Shanxi | 140923 | Dai County | 5.7 | 4.5 | 6.9 |  | 68.9(57.3,79.5) | 70.5(58.1,82.3) | 69.6(58.2,78.8) |
| Shanxi | 140428 | Zhangzi County | 5.0 | 4.3 | 5.6 |  | 68.3(57.2,77.6) | 65.1(54.2,75.7) | 76.1(64.9,85.6) |
| Shanxi | 141182 | Fenyang City | 4.8 | 4.0 | 5.6 |  | 68.3(56.9,78.6) | 67.3(55.1,78.8) | 70.2(57.4,82.4) |
| Shanxi | 140423 | Xiangyuan County | 4.9 | 4.3 | 5.5 |  | 68.1(56.0,79.5) | 66.7(52.9,79.3) | 73.5(62.3,84.7) |
| Shanxi | 140107 | Xinghualing District | 5.0 | 4.1 | 5.8 |  | 67.9(44.7,89.6) | 67.3(45.2,87.7) | 69.6(43.3,92.6) |
| Shanxi | 140525 | Zezhou County | 5.4 | 4.9 | 5.9 |  | 67.7(47.0,83.8) | 62.9(44.3,80.1) | 75.8(57.6,90.1) |
| Shanxi | 140411 | Jiao District | 3.8 | 3.4 | 4.3 |  | 67.6(52.8,80.9) | 64.5(49.8,79.4) | 75.3(60.5,87.7) |
| Shanxi | 141121 | Wenshui County | 4.4 | 3.6 | 5.1 |  | 67.3(54.2,78.2) | 66.5(53.8,78.8) | 71.2(58.1,82.2) |
| Shanxi | 141027 | Fushan County | 5.4 | 4.6 | 6.2 |  | 66.6(55.9,76.8) | 66.7(55.0,76.7) | 71.3(60.3,81.3) |
| Shanxi | 141124 | Lin County | 4.4 | 3.7 | 5.2 |  | 66.1(55.2,76.4) | 63.3(52.3,74.5) | 73.4(60.4,83.3) |
| Shanxi | 141102 | Lishi District | 1.4 | 1.0 | 1.7 |  | 65.4(47.2,82.8) | 61.1(44.1,81.6) | 74.1(55.6,91.6) |
| Shanxi | 140202 | Cheng District | 2.2 | 1.7 | 2.8 |  | 64.8(49.6,80.0) | 68.9(51.3,84.9) | 61.8(45.1,80.1) |
| Shanxi | 140826 | Jiang County | 5.7 | 5.0 | 6.4 |  | 64.6(52.6,76.2) | 61.9(49.8,74.1) | 70.1(55.6,83.0) |
| Shanxi | 141125 | Liulin County | 3.0 | 2.9 | 3.2 |  | 64.6(51.7,77.2) | 64.9(49.2,80.2) | 68.7(58.2,78.4) |
| Shanxi | 140225 | Hunyuan County | 4.1 | 3.1 | 5.0 |  | 64.0(50.5,76.4) | 66.4(53.2,78.8) | 66.4(52.1,79.2) |
| Shanxi | 140828 | Xia County | 5.3 | 5.0 | 5.6 |  | 63.2(51.5,73.2) | 60.2(48.3,71.4) | 67.3(55.4,77.2) |
| Shanxi | 140481 | Lucheng City | 4.7 | 4.0 | 5.4 |  | 62.6(51.1,74.1) | 57.2(46.0,68.9) | 73.0(58.1,85.9) |
| Shanxi | 141028 | Ji County | 4.8 | 4.2 | 5.4 |  | 62.4(41.4,82.4) | 62.2(40.9,86.8) | 66.9(47.8,82.3) |
| Shanxi | 140722 | Zuoquan County | 5.9 | 4.9 | 6.7 |  | 62.1(49.1,72.7) | 57.1(43.9,69.6) | 68.9(55.5,82.7) |
| Shanxi | 140223 | Guangling County | 4.5 | 3.5 | 5.4 |  | 61.9(51.8,71.5) | 62.2(52.2,72.3) | 67.2(56.7,76.7) |
| Shanxi | 140727 | Qi County | 5.2 | 4.5 | 5.9 |  | 61.2(49.7,71.3) | 60.6(47.7,73.7) | 64.7(53.3,74.7) |
| Shanxi | 140430 | Qin County | 5.0 | 4.2 | 5.8 |  | 60.1(47.3,72.8) | 57.6(44.6,70.9) | 65.8(50.0,83.6) |
| Shanxi | 140781 | Jiexiu City | 4.6 | 4.0 | 5.3 |  | 60.1(49.5,70.7) | 61.2(49.1,72.7) | 60.6(50.5,71.8) |
| Shanxi | 141033 | Pu County | 5.1 | 4.6 | 5.4 |  | 59.3(47.6,70.4) | 56.1(44.6,67.7) | 68.1(55.4,78.3) |
| Shanxi | 141126 | Shilou County | 2.6 | 2.3 | 3.0 |  | 59.2(43.5,77.9) | 59.4(42.2,83.2) | 64.7(52.2,76.0) |
| Shanxi | 140122 | Yangqu County | 5.7 | 4.5 | 6.8 |  | 59.1(41.9,74.7) | 57.8(39.6,76.6) | 59.3(41.4,81.2) |
| Shanxi | 140922 | Wutai County | 5.5 | 4.7 | 6.2 |  | 58.4(38.6,79.6) | 54.5(33.8,75.3) | 66.4(43.3,88.4) |
| Shanxi | 140927 | Shenchi County | 4.4 | 3.6 | 5.1 |  | 57.7(45.9,69.3) | 57.4(46.2,69.7) | 65.0(52.2,76.8) |
| Shanxi | 140929 | Kelan County | 3.7 | 3.0 | 4.4 |  | 57.7(41.7,80.3) | 61.0(41.1,87.7) | 55.7(41.6,69.4) |
| Shanxi | 140322 | Yu County | 4.9 | 4.2 | 5.6 |  | 56.8(37.2,75.8) | 50.0(31.9,71.0) | 68.8(47.3,87.0) |
| Shanxi | 140212 | Xinrong District | 2.5 | 2.0 | 3.1 |  | 55.8(38.8,79.4) | 59.7(40.5,86.3) | 55.5(43.3,68.2) |
| Shanxi | 140724 | Xiyang County | 6.6 | 5.8 | 7.3 |  | 54.5(36.3,73.8) | 51.3(30.7,71.6) | 60.5(41.2,78.1) |
| Shanxi | 140932 | Pianguan County | 4.6 | 3.9 | 5.3 |  | 54.5(42.6,67.2) | 51.6(40.5,64.6) | 63.3(50.6,75.1) |
| Shanxi | 141034 | Fenxi County | 4.3 | 3.6 | 5.1 |  | 54.4(42.2,67.1) | 54.8(42.8,67.6) | 58.9(45.4,71.9) |
| Shanxi | 141130 | Jiaokou County | 3.2 | 2.7 | 3.7 |  | 53.2(39.1,70.1) | 47.3(36.1,59.2) | 67.1(48.2,87.0) |
| Shanxi | 140221 | Yanggao County | 3.5 | 3.0 | 4.0 |  | 53.1(39.9,67.8) | 53.4(37.1,72.2) | 57.9(44.9,70.0) |
| Shanxi | 141123 | Xing County | 2.7 | 2.2 | 3.2 |  | 52.2(39.6,65.2) | 52.2(39.8,66.0) | 58.9(46.3,70.3) |
| Shanxi | 141030 | Daning County | 4.1 | 3.2 | 4.9 |  | 52.1(36.7,70.2) | 56.1(39.1,77.1) | 49.0(35.7,62.8) |
| Shanxi | 140928 | Wuzhai County | 2.4 | 1.7 | 3.1 |  | 51.9(32.0,76.7) | 53.4(30.5,84.6) | 48.7(30.2,82.8) |
| Shanxi | 141181 | Xiaoyi City | 3.1 | 2.6 | 3.6 |  | 51.7(39.6,66.1) | 53.2(38.7,67.7) | 54.0(40.6,69.1) |
| Shanxi | 140621 | Shanyin County | 2.6 | 1.9 | 3.2 |  | 51.6(35.9,70.4) | 45.2(32.2,60.8) | 60.5(40.8,83.1) |
| Shanxi | 141128 | Fangshan County | 3.0 | 2.4 | 3.6 |  | 51.5(37.8,67.4) | 48.0(35.6,60.0) | 61.9(43.1,82.3) |
| Shanxi | 140624 | Huairen County | 1.9 | 1.4 | 2.4 |  | 50.2(36.4,66.1) | 44.3(33.7,55.8) | 66.1(43.6,87.6) |
| Shanxi | 140226 | Zuoyun County | 2.6 | 1.9 | 3.3 |  | 50.1(32.8,67.9) | 51.4(34.9,69.3) | 56.1(36.1,75.6) |
| Shanxi | 140302 | Cheng District | 3.8 | 2.9 | 4.8 |  | 49.2(35.9,66.7) | 53.1(37.9,73.1) | 47.9(33.3,70.5) |
| Shanxi | 140930 | Hequ County | 3.7 | 3.2 | 4.1 |  | 48.9(36.3,60.9) | 46.9(34.3,60.7) | 56.1(42.4,69.2) |
| Shanxi | 140723 | Heshun County | 5.2 | 4.4 | 5.9 |  | 48.5(37.3,60.8) | 43.9(32.9,54.9) | 58.6(44.3,73.6) |
| Shanxi | 140926 | Jingle County | 2.4 | 1.9 | 2.8 |  | 47.8(25.1,77.0) | 41.6(23.6,74.8) | 54.8(30.1,88.7) |
| Shanxi | 140721 | Yushe County | 5.3 | 4.9 | 5.5 |  | 47.7(31.0,65.9) | 41.8(26.1,58.9) | 60.9(40.4,78.4) |
| Shanxi | 140921 | Dingxiang County | 3.9 | 3.5 | 4.4 |  | 47.1(34.8,61.6) | 44.1(31.2,61.4) | 54.4(41.0,69.4) |
| Shanxi | 140311 | Jiao District | 3.9 | 3.5 | 4.3 |  | 46.0(28.3,64.3) | 45.4(28.2,67.1) | 48.9(30.4,66.9) |
| Shanxi | 140108 | Jiancaoping District | 2.9 | 2.6 | 3.3 |  | 44.9(32.8,59.5) | 42.8(30.8,58.7) | 51.4(35.6,69.2) |
| Shanxi | 140924 | Fanzhi County | 2.9 | 2.2 | 3.6 |  | 44.1(32.5,56.6) | 45.7(32.2,61.9) | 47.5(36.5,59.8) |
| Shanxi | 140603 | Pinglu District | 1.3 | 0.9 | 1.6 |  | 43.4(27.4,68.2) | 43.4(25.9,71.3) | 45.8(28.8,72.6) |
| Shanxi | 140203 | Kuang District | 2.1 | 1.5 | 2.6 |  | 43.0(30.0,59.1) | 44.5(31.8,60.6) | 45.2(31.5,60.6) |
| Shanxi | 140931 | Baode County | 2.2 | 1.8 | 2.6 |  | 42.3(31.6,53.9) | 41.6(31.4,53.7) | 50.2(38.6,62.3) |
| Shanxi | 141127 | Lan County | 2.3 | 1.6 | 3.0 |  | 42.1(29.8,58.3) | 46.6(32.8,66.0) | 42.3(31.3,55.3) |
| Shanxi | 140429 | Wuxiang County | 4.7 | 3.8 | 5.4 |  | 41.9(31.6,53.2) | 40.4(30.6,51.4) | 46.6(35.1,61.0) |
| Shanxi | 140925 | Ningwu County | 1.8 | 1.4 | 2.1 |  | 39.5(25.3,58.8) | 37.8(23.0,63.3) | 43.8(29.0,68.7) |
| Shanxi | 140222 | Tianzhen County | 3.0 | 2.3 | 3.6 |  | 32.6(23.8,45.0) | 29.8(20.7,40.5) | 40.8(28.8,57.2) |
| Shanxi | 140123 | Loufan County | 2.4 | 2.3 | 2.5 |  | 31.5(23.1,41.9) | 27.5(18.8,38.2) | 44.4(33.3,57.3) |
| Shanxi | 140622 | Ying County | 1.5 | 1.3 | 1.8 |  | 28.7(18.7,42.7) | 25.6(17.1,39.0) | 36.9(24.4,55.8) |
| Shanxi | 140623 | Youyu County | 1.3 | 1.0 | 1.6 |  | 23.9(16.0,33.2) | 22.2(14.8,31.5) | 30.7(20.4,42.6) |
| Qinghai | 632128 | Zizhixian | 5.8 | 5.3 | 6.3 |  | 98.1(94.4,99.6) | 97.3(92.2,99.5) | 98.3(94.1,99.7) |
| Qinghai | 632822 | Dulan County | 4.8 | 3.6 | 5.9 |  | 95.4(75.2,99.8) | 91.9(68.8,99.5) | 96.2(75.4,99.8) |
| Qinghai | 632523 | Guide County | 6.5 | 4.8 | 8.1 |  | 94.0(85.7,97.9) | 96.3(89.1,99.2) | 85.1(69.2,95.3) |
| Qinghai | 632221 | Zizhixian | 4.8 | 4.1 | 5.5 |  | 93.7(85.1,98.3) | 89.4(76.1,96.9) | 95.8(87.7,99.1) |
| Qinghai | 632621 | Maqeen County | 4.0 | 3.0 | 4.8 |  | 91.3(75.2,98.0) | 94.3(77.2,99.3) | 79.7(55.6,95.8) |
| Qinghai | 632823 | Tianjun County | 4.5 | 4.9 | 4.3 |  | 90.9(62.3,99.5) | 98.1(64.1,100.0) | 73.1(50.1,86.8) |
| Qinghai | 632323 | Zeekog County | 3.4 | 2.9 | 3.9 |  | 90.7(76.0,97.8) | 84.6(63.3,96.4) | 93.3(77.9,98.8) |
| Qinghai | 632624 | Tarlag County | 4.3 | 3.9 | 4.6 |  | 89.4(70.4,97.8) | 93.7(68.2,99.5) | 78.1(50.3,94.8) |
| Qinghai | 632802 | Delhi City | 4.0 | 3.5 | 4.6 |  | 88.0(60.5,98.8) | 77.5(48.7,96.7) | 93.7(65.7,99.9) |
| Qinghai | 632521 | Gonghe County | 5.3 | 4.7 | 5.9 |  | 84.9(72.2,93.1) | 83.5(68.5,93.9) | 86.4(73.3,94.5) |
| Qinghai | 632127 | Zizhixian | 5.2 | 4.4 | 6.0 |  | 84.4(75.3,91.7) | 86.1(75.2,94.1) | 82.4(72.0,90.2) |
| Qinghai | 632321 | Tongren County | 4.8 | 4.6 | 4.9 |  | 84.2(70.5,94.3) | 82.2(62.8,94.0) | 85.5(67.2,95.1) |
| Qinghai | 632524 | Xinghai County | 3.5 | 2.9 | 4.1 |  | 82.3(65.8,93.2) | 73.5(53.1,90.5) | 89.4(71.9,97.1) |
| Qinghai | 632522 | Tongde County | 4.4 | 3.9 | 5.0 |  | 81.6(65.9,92.0) | 73.4(54.0,88.5) | 87.0(68.9,97.0) |
| Qinghai | 632123 | Ledu County | 5.1 | 4.3 | 6.0 |  | 79.7(69.1,88.6) | 78.8(66.6,88.2) | 81.1(68.1,89.9) |
| Qinghai | 632126 | Huzhu Tuzu Zizhixian | 6.1 | 5.3 | 6.8 |  | 76.5(67.0,84.6) | 77.6(66.5,86.4) | 76.4(66.7,84.8) |
| Qinghai | 630104 | Chengxi District | 3.8 | 2.9 | 4.7 |  | 73.6(49.4,92.4) | 67.4(43.2,88.6) | 77.9(45.2,97.2) |
| Qinghai | 632525 | Guinan County | 4.6 | 3.9 | 5.3 |  | 73.4(57.2,86.5) | 71.1(54.3,87.2) | 77.3(60.2,91.0) |
| Qinghai | 630122 | Huangzhong County | 4.9 | 4.1 | 5.7 |  | 71.8(61.4,81.0) | 72.5(60.7,82.8) | 72.1(61.1,80.8) |
| Qinghai | 632324 | Zizhixian | 4.3 | 3.3 | 5.3 |  | 69.2(49.3,87.0) | 63.2(40.1,84.2) | 72.9(44.5,93.4) |
| Qinghai | 630123 | Huangyuan County | 4.8 | 4.2 | 5.4 |  | 66.6(52.4,81.2) | 63.9(48.0,80.5) | 73.0(56.6,87.3) |
| Qinghai | 632801 | Golmud City | 1.6 | 1.4 | 1.7 |  | 59.3(39.2,79.4) | 58.9(38.1,82.2) | 58.9(40.9,80.0) |
| Qinghai | 630105 | Chengbei District | 3.8 | 3.1 | 4.4 |  | 59.2(40.8,79.1) | 49.0(35.3,65.9) | 72.3(49.1,92.4) |
| Qinghai | 632122 | Zizhixian | 4.4 | 3.7 | 5.2 |  | 58.4(45.0,71.4) | 59.2(44.3,73.6) | 55.2(40.8,70.1) |
| Qinghai | 632224 | Gangcha County | 3.6 | 3.1 | 4.1 |  | 58.2(39.8,80.3) | 69.8(43.9,90.9) | 45.0(30.4,65.1) |
| Qinghai | 632322 | Jainca County | 3.0 | 2.5 | 3.4 |  | 57.7(36.7,80.9) | 49.3(28.1,77.7) | 65.3(37.8,90.5) |
| Qinghai | 630121 | Zizhixian | 5.3 | 4.5 | 6.1 |  | 56.2(45.0,68.8) | 55.4(42.8,67.5) | 58.9(46.3,70.3) |
| Qinghai | 632622 | Baima County | 3.0 | 3.0 | 3.0 |  | 54.8(30.0,82.1) | 66.3(31.0,93.5) | 37.0(20.5,62.0) |
| Qinghai | 630103 | Chengzhong District | 4.0 | 3.4 | 4.7 |  | 53.1(35.1,74.3) | 53.8(36.8,76.2) | 53.7(35.1,76.4) |
| Qinghai | 632824 | Lenghu Xingwei | 4.1 | 1.2 | 6.8 |  | 53.0(26.9,79.1) | 71.5(33.9,90.5) | 28.1(11.6,61.2) |
| Qinghai | 632222 | Qilian County | 4.2 | 3.9 | 4.6 |  | 50.9(35.1,67.5) | 54.0(33.9,79.3) | 50.1(37.3,63.6) |
| Qinghai | 630102 | Chengdong District | 3.1 | 2.6 | 3.5 |  | 45.5(30.1,67.1) | 51.4(32.4,73.0) | 38.7(24.9,58.1) |
| Qinghai | 632223 | Haiyan County | 3.4 | 3.0 | 3.9 |  | 45.3(27.7,74.8) | 48.5(25.6,85.0) | 47.6(29.6,65.5) |
| Qinghai | 632623 | Gadee County | 1.9 | 1.9 | 1.9 |  | 37.8(19.9,67.5) | 37.4(16.6,71.3) | 35.3(17.8,64.1) |
| Qinghai | 632821 | Ulan County | 2.3 | 1.8 | 2.7 |  | 34.1(18.0,67.9) | 30.6(18.5,48.2) | 42.2(20.4,86.1) |
| Qinghai | 632721 | Yushu County | 2.1 | 1.9 | 2.4 |  | 30.9(21.5,44.0) | 31.5(20.9,47.5) | 34.0(22.9,49.6) |
| Qinghai | 632726 | Qumarleeb County | 2.3 | 1.9 | 2.8 |  | 30.8(18.6,51.4) | 22.7(14.3,34.3) | 45.8(23.3,78.6) |
| Qinghai | 632724 | Zhidoi County | 1.2 | 0.8 | 1.7 |  | 23.8(13.4,44.9) | 26.5(13.4,57.0) | 22.5(12.2,45.3) |
| Qinghai | 632825 | Dachaidan Xingwei | 1.6 | 1.2 | 1.9 |  | 23.2(13.2,38.0) | 23.8(12.3,41.4) | 29.9(16.3,48.8) |
| Qinghai | 632722 | Zadoi County | 0.9 | 0.6 | 1.2 |  | 22.3(13.5,36.9) | 22.5(12.7,38.5) | 24.2(13.6,42.5) |
| Qinghai | 632826 | Mangya Xingwei | 0.6 | 0.4 | 0.9 |  | 19.9(9.5,54.6) | 14.8(8.2,25.7) | 31.6(13.1,85.2) |
| Qinghai | 632625 | Jigzhi County | 2.2 | 2.4 | 2.1 |  | 17.9(10.1,31.9) | 15.6(7.6,33.1) | 21.1(11.2,39.3) |
| Qinghai | 632626 | Madoi County | 2.5 | 0.9 | 4.0 |  | 16.0(9.4,25.9) | 23.7(12.7,40.7) | 10.9(6.4,19.4) |
| Qinghai | 632725 | Nangqeen County | 0.6 | 0.5 | 0.7 |  | 15.4(9.9,24.0) | 11.8(7.6,17.8) | 24.6(14.9,44.3) |
| Qinghai | 632723 | Chindu County | 0.7 | 0.5 | 0.9 |  | 10.4(6.5,16.5) | 9.7(6.1,16.1) | 13.1(7.9,21.9) |
| Qinghai | 632121 | Ping'an County | 0.0 | 0.0 | 0.0 |  | 7.6(5.0,11.4) | 7.1(4.6,11.1) | 10.1(6.8,15.3) |
| Jiangxi | 360122 | Xinjian County | 5.4 | 4.7 | 6.0 |  | 97.0(94.4,98.7) | 95.0(90.5,97.6) | 98.0(95.2,99.4) |
| Jiangxi | 360702 | ZhangGong District | 5.6 | 4.8 | 6.3 |  | 92.2(85.9,96.4) | 91.1(83.4,95.9) | 92.1(84.2,97.0) |
| Jiangxi | 360726 | Anyuan County | 5.5 | 4.9 | 6.1 |  | 90.5(83.0.,95.5) | 89.8(80.6,95.6) | 88.0(76.9,94.9) |
| Jiangxi | 360827 | Suichuan County | 6.2 | 5.0 | 7.3 |  | 89.8(83.7,94.1) | 91.3(85.2,95.7) | 86.0(77.9,92.5) |
| Jiangxi | 360721 | Gan County | 6.7 | 6.0 | 7.4 |  | 88.8(82.2,93.4) | 85.9(78.7,92.2) | 90.7(82.9,95.5) |
| Jiangxi | 360105 | Wanli District | 6.9 | 6.4 | 7.4 |  | 88.6(72.9,96.7) | 87.5(65.8,97.6) | 87.3(72.9,97.4) |
| Jiangxi | 360123 | Anyi County | 6.3 | 5.4 | 7.1 |  | 88.3(78.1,94.8) | 85.1(72.7,93.8) | 90.1(76.9,97.1) |
| Jiangxi | 360423 | Wuning County | 6.2 | 5.3 | 7.1 |  | 86.4(79.1,91.7) | 85.0(77.5,91.4) | 87.6(78.6,93.4) |
| Jiangxi | 360502 | Yushui District | 5.5 | 4.5 | 6.4 |  | 85.0(77.7,90.7) | 85.6(77.8,91.8) | 83.6(74.8,90.9) |
| Jiangxi | 360821 | Ji'an County | 6.1 | 5.3 | 6.8 |  | 85.0(77.5,90.4) | 85.3(77.4,91.3) | 84.6(75.6,90.8) |
| Jiangxi | 361024 | Chongren County | 6.3 | 4.9 | 7.6 |  | 84.7(76.0,90.4) | 83.2(74.2,89.8) | 86.3(76.6,93.5) |
| Jiangxi | 360802 | Jizhou District | 5.9 | 5.0 | 6.8 |  | 84.0(73.4,92.2) | 85.2(72.8,93.8) | 79.0(65.3,90.3) |
| Jiangxi | 360102 | Donghu District | 5.1 | 4.1 | 6.1 |  | 83.5(73.9,91.5) | 84.1(72.9,93.0) | 80.1(67.5,91.1) |
| Jiangxi | 360982 | Zhangshu City | 6.1 | 5.4 | 6.8 |  | 82.8(74.9,89.1) | 82.7(73.4,90.0) | 83.5(73.7,90.6) |
| Jiangxi | 361129 | Wannian County | 5.8 | 4.8 | 6.7 |  | 82.7(74.0,89.3) | 82.7(73.6,90.5) | 81.1(70.3,89.3) |
| Jiangxi | 360482 | Gongqingcheng City | 5.0 | 4.1 | 5.9 |  | 80.9(66.5,91.7) | 84.8(68.8,94.7) | 76.0(60.9,89.3) |
| Jiangxi | 360121 | Nanchang County | 4.6 | 4.2 | 5.1 |  | 79.7(71.0,86.8) | 78.0(67.8,85.5) | 81.7(71.6,89.4) |
| Jiangxi | 361002 | Linchuan District | 4.7 | 4.0 | 5.2 |  | 79.4(71.6,86.2) | 76.1(67.3,83.8) | 83.8(75.0,90.5) |
| Jiangxi | 360734 | Xunwu County | 5.3 | 4.5 | 6.1 |  | 79.1(68.2,87.4) | 83.4(71.6,92.4) | 70.2(58.3,81.1) |
| Jiangxi | 360824 | Xingan County | 5.0 | 4.6 | 5.3 |  | 77.4(66.6,86.2) | 78.7(67.2,88.1) | 75.0(62.8,85.1) |
| Jiangxi | 360922 | Wanzai County | 6.5 | 5.3 | 7.6 |  | 77.0(68.9,83.6) | 80.3(71.9,86.5) | 74.0(64.7,81.5) |
| Jiangxi | 360322 | Shangli County | 5.8 | 5.1 | 6.5 |  | 76.2(66.5,84.0) | 75.9(65.3,84.7) | 77.6(68.2,85.3) |
| Jiangxi | 360727 | Longnan County | 5.9 | 5.1 | 6.7 |  | 75.7(51.7,93.1) | 74.8(52.8,92.0) | 76.6(53.7,93.8) |
| Jiangxi | 360803 | Qingyuan District | 3.7 | 3.3 | 4.0 |  | 75.2(46.8,94.7) | 79.4(49.6,95.6) | 64.8(40.4,87.0) |
| Jiangxi | 360421 | Jiujiang County | 4.3 | 3.9 | 4.7 |  | 74.5(61.9,85.9) | 74.7(58.6,87.7) | 74.8(60.8,86.6) |
| Jiangxi | 360902 | Yuanzhou District | 6.2 | 5.1 | 7.3 |  | 73.5(64.5,80.7) | 74.6(66.7,81.0) | 73.2(63.4,80.7) |
| Jiangxi | 360481 | Ruichang City | 4.6 | 3.9 | 5.2 |  | 73.3(62.0,82.5) | 74.9(63.8,83.8) | 71.6(60.3,82.3) |
| Jiangxi | 360202 | Changjiang District | 5.0 | 4.0 | 5.8 |  | 73.1(59.9,85.2) | 76.2(61.7,88.8) | 70.0(57.1,84.8) |
| Jiangxi | 360622 | Yujiang County | 6.0 | 5.0 | 6.9 |  | 73.0(63.0,80.8) | 76.2(66.9,84.8) | 68.5(59.0,77.1) |
| Jiangxi | 360681 | Guixi City | 4.9 | 4.0 | 5.7 |  | 72.9(63.6,80.8) | 76.5(65.9,84.8) | 68.0(58.5,77.2) |
| Jiangxi | 360735 | Shicheng County | 5.2 | 4.7 | 5.7 |  | 72.5(61.3,82.8) | 62.8(50.4,74.3) | 83.0(68.6,92.4) |
| Jiangxi | 360427 | Xingzi County | 3.3 | 2.6 | 3.8 |  | 72.3(57.8,85.2) | 72.8(57.9,86.8) | 70.8(53.9,85.7) |
| Jiangxi | 360983 | Gao'an City | 5.1 | 4.6 | 5.6 |  | 72.2(63.2,79.5) | 71.1(62.1,79.1) | 71.7(61.0,79.9) |
| Jiangxi | 361029 | Dongxiang County | 4.4 | 4.0 | 4.8 |  | 72.0(61.9,81.1) | 73.2(62.2,83.7) | 71.9(61.2,81.4) |
| Jiangxi | 360725 | Chongyi County | 5.7 | 4.7 | 6.7 |  | 71.9(59.7,83.3) | 74.0(61.0,86.4) | 68.9(53.0,83.1) |
| Jiangxi | 360104 | Qingyunpu District | 4.7 | 4.0 | 5.4 |  | 71.5(55.4,86.5) | 67.6(49.9,84.1) | 76.7(57.4,91.7) |
| Jiangxi | 361027 | Jinxi County | 3.7 | 3.2 | 4.2 |  | 71.4(60.0,82.0) | 73.9(61.1,84.4) | 68.3(56.3,80.3) |
| Jiangxi | 360926 | Tonggu County | 6.1 | 5.3 | 6.9 |  | 71.3(58.8,81.2) | 68.4(55.8,79.5) | 76.6(64.0,88.0) |
| Jiangxi | 360729 | Quannan County | 5.9 | 5.4 | 6.4 |  | 71.0(58.0,82.0) | 66.6(54.0,78.5) | 77.2(61.0,89.3) |
| Jiangxi | 360733 | Huichang County | 6.4 | 5.4 | 7.2 |  | 71.0(61.5,78.8) | 72.2(62.6,80.3) | 70.4(59.2,79.3) |
| Jiangxi | 360923 | Shanggao County | 6.0 | 5.2 | 6.8 |  | 71.0(60.1,78.8) | 70.5(59.9,80.0) | 71.8(60.8,81.4) |
| Jiangxi | 361022 | Lichuan County | 4.4 | 3.5 | 5.2 |  | 71.0(56.8,83.9) | 67.8(52.9,81.2) | 74.0(56.7,88.7) |
| Jiangxi | 360822 | Jishui County | 3.9 | 3.4 | 4.4 |  | 70.9(59.1,80.4) | 70.7(59.1,80.6) | 70.8(56.0,83.7) |
| Jiangxi | 360281 | Leping City | 3.5 | 2.9 | 4.1 |  | 70.5(61.4,80.1) | 66.4(55.5,75.7) | 76.9(66.1,87.0) |
| Jiangxi | 360724 | Shangyou County | 5.1 | 4.0 | 6.1 |  | 70.5(57.2,82.3) | 69.6(56.5,81.1) | 71.1(52.5,87.9) |
| Jiangxi | 360830 | Yongxin County | 5.8 | 4.9 | 6.7 |  | 70.5(60.9,79.0) | 71.8(61.6,79.9) | 70.8(60.0,80.8) |
| Jiangxi | 360723 | Dayu County | 6.4 | 5.5 | 7.4 |  | 69.2(58.7,79.1) | 70.6(58.9,81.0) | 67.7(55.6,78.7) |
| Jiangxi | 360823 | Xiajiang County | 5.0 | 4.4 | 5.6 |  | 68.9(56.8,80.4) | 70.6(57.2,82.9) | 66.5(52.7,79.2) |
| Jiangxi | 360222 | Fuliang County | 5.2 | 4.7 | 5.6 |  | 68.2(57.0,78.2) | 62.5(51.8,73.0) | 77.6(65.3,87.2) |
| Jiangxi | 360425 | Yongxiu County | 3.4 | 2.8 | 4.0 |  | 67.7(53.9,80.7) | 69.3(54.0,82.9) | 65.7(49.5,81.7) |
| Jiangxi | 360426 | De'an County | 3.6 | 3.2 | 4.1 |  | 67.2(54.3,82.8) | 63.6(48.1,82.2) | 74.4(57.1,89.9) |
| Jiangxi | 361025 | Le'an County | 4.1 | 3.5 | 4.7 |  | 66.7(54.5,78.4) | 70.7(57.0,83.3) | 61.4(48.4,73.6) |
| Jiangxi | 360323 | Luxi County | 5.3 | 4.5 | 6.0 |  | 66.3(54.2,76.6) | 63.7(51.5,75.1) | 70.5(57.0,82.6) |
| Jiangxi | 360730 | Ningdu County | 4.6 | 3.9 | 5.4 |  | 65.2(55.7,74.4) | 67.4(57.3,76.1) | 62.3(51.9,72.2) |
| Jiangxi | 360981 | Fengcheng City | 5.0 | 4.5 | 5.4 |  | 65.0(55.4,73.2) | 63.3(54.5,71.6) | 67.9(58.0,76.4) |
| Jiangxi | 360925 | Jing'an County | 5.8 | 5.2 | 6.3 |  | 64.5(51.8,75.9) | 56.6(43.9,70.0) | 74.5(60.8,87.5) |
| Jiangxi | 361121 | Shangrao County | 4.5 | 3.9 | 5.1 |  | 63.9(53.6,72.8) | 64.1(53.3,74.1) | 65.5(54.8,75.1) |
| Jiangxi | 360781 | Ruijin City | 5.2 | 4.3 | 6.1 |  | 63.3(54.1,72.9) | 64.3(53.9,73.5) | 64.4(53.8,74.3) |
| Jiangxi | 360728 | Dingnan County | 5.1 | 4.3 | 5.9 |  | 62.8(49.5,74.9) | 66.8(52.5,78.5) | 56.4(42.0,70.9) |
| Jiangxi | 361030 | Guangchang County | 4.0 | 3.5 | 4.6 |  | 62.4(50.0,76.2) | 54.7(42.9,67.3) | 76.2(57.8,90.0) |
| Jiangxi | 360921 | Fengxin County | 5.4 | 4.7 | 6.1 |  | 62.2(51.5,72.5) | 56.6(46.2,67.6) | 70.6(58.0,81.3) |
| Jiangxi | 361102 | xinzhou District | 2.3 | 1.6 | 3.0 |  | 61.6(46.2,76.3) | 58.5(43.2,75.2) | 66.1(47.2,84.7) |
| Jiangxi | 360722 | Xinfeng County | 5.5 | 4.8 | 6.2 |  | 61.5(52.2,70.9) | 63.9(54.0,72.3) | 59.8(49.2,69.6) |
| Jiangxi | 360124 | Jinxian County | 4.2 | 3.4 | 4.9 |  | 60.1(50.3,69.8) | 59.9(50.1,69.4) | 63.4(53.0,74.0) |
| Jiangxi | 360428 | Duchang County | 4.8 | 3.9 | 5.6 |  | 59.5(38.8,80.3) | 59.7(38.9,80.0) | 63.3(40.3,82.0) |
| Jiangxi | 360402 | Lushan District | 3.5 | 3.2 | 3.9 |  | 59.4(45.2,74.4) | 58.8(42.4,76.7) | 60.1(45.2,79.0) |
| Jiangxi | 360313 | Xiangdong District | 4.1 | 3.6 | 4.5 |  | 59.2(45.3,72.3) | 58.2(45.1,72.4) | 62.5(47.8,77.0) |
| Jiangxi | 361122 | Guangfeng County | 4.0 | 3.2 | 4.9 |  | 59.0(49.2,68.3) | 60.1(49.7,69.3) | 61.0(49.5,70.3) |
| Jiangxi | 361130 | Wuyuan Xia | 5.5 | 4.5 | 6.6 |  | 58.7(47.9,68.2) | 62.5(52.5,72.4) | 57.4(47.2,67.7) |
| Jiangxi | 360430 | Pengze County | 3.6 | 3.0 | 4.1 |  | 58.5(47.1,70.0) | 55.4(44.3,66.9) | 66.4(51.4,80.3) |
| Jiangxi | 360924 | Yifeng County | 4.9 | 3.9 | 5.9 |  | 58.2(46.4,69.5) | 61.7(50.1,75.0) | 54.2(42.4,67.1) |
| Jiangxi | 361123 | Yushan County | 4.8 | 3.9 | 5.6 |  | 57.2(47.3,66.5) | 58.2(48.5,68.6) | 57.2(46.9,67.1) |
| Jiangxi | 360103 | Xihu District | 2.5 | 2.0 | 3.0 |  | 56.7(39.7,74.7) | 49.0(33.9,68.5) | 63.3(41.2,84.6) |
| Jiangxi | 360782 | Nankang City | 4.4 | 3.9 | 4.9 |  | 56.4(46.2,66.1) | 58.3(49.2,68.0) | 56.0(45.4,66.1) |
| Jiangxi | 360829 | Anfu County | 5.0 | 4.4 | 5.6 |  | 56.1(45.9,66.2) | 56.8(45.5,69.5) | 56.4(44.7,67.6) |
| Jiangxi | 361125 | Hengfeng County | 5.1 | 3.8 | 6.3 |  | 55.8(42.9,67.9) | 63.7(48.0,77.2) | 44.6(32.1,58.4) |
| Jiangxi | 361026 | Yihuang County | 4.6 | 3.9 | 5.3 |  | 55.6(35.4,75.7) | 54.9(33.7,76.3) | 60.0(37.0,83.6) |
| Jiangxi | 361023 | Nanfeng County | 4.4 | 3.5 | 5.4 |  | 54.8(44.6,65.9) | 52.2(42.4,63.2) | 60.0(45.5,73.6) |
| Jiangxi | 360424 | Xiushui County | 3.8 | 3.0 | 4.6 |  | 54.1(44.7,64.5) | 54.4(44.6,64.5) | 57.3(46.4,67.8) |
| Jiangxi | 360826 | Taihe County | 3.5 | 3.1 | 3.9 |  | 54.1(42.7,65.2) | 48.5(38.4,59.4) | 63.8(49.9,78.4) |
| Jiangxi | 360321 | Lianhua County | 4.5 | 3.9 | 5.0 |  | 53.3(42.5,66.0) | 51.0(39.2,64.2) | 57.1(43.7,72.0) |
| Jiangxi | 360403 | Xunyang District | 3.3 | 2.6 | 4.0 |  | 53.1(38.9,71.1) | 58.0(40.9,76.9) | 47.2(34.7,65.3) |
| Jiangxi | 360111 | Qingshanhu District | 2.4 | 2.1 | 2.5 |  | 52.2(40.7,63.8) | 50.6(38.3,63.8) | 55.5(43.1,68.2) |
| Jiangxi | 360731 | Yudu County | 5.5 | 4.6 | 6.5 |  | 50.6(40.6,60.8) | 51.7(41.4,60.9) | 51.3(41.0,61.8) |
| Jiangxi | 361021 | Nancheng County | 4.2 | 3.5 | 4.8 |  | 50.3(38.5,60.8) | 50.0(39.3,61.4) | 55.2(41.9,67.6) |
| Jiangxi | 360429 | Hukou County | 3.8 | 3.2 | 4.3 |  | 49.8(39.1,61.0) | 50.2(39.1,63.2) | 52.3(40.1,64.7) |
| Jiangxi | 361124 | Yanshan County | 3.7 | 3.0 | 4.3 |  | 48.6(38.9,58.7) | 49.9(39.9,60.6) | 50.0(39.9,61.0) |
| Jiangxi | 361181 | Dexing City | 3.9 | 3.2 | 4.6 |  | 47.5(37.2,58.3) | 53.3(41.0,66.2) | 42.4(33.4,52.3) |
| Jiangxi | 360302 | Anyuan District | 2.7 | 2.3 | 3.1 |  | 46.8(35.3,59.0) | 50.3(37.4,67.2) | 44.6(34.1,57.4) |
| Jiangxi | 361128 | Poyang County | 4.1 | 3.5 | 4.7 |  | 46.2(37.9,54.8) | 46.2(37.4,55.5) | 49.7(40.0,59.0) |
| Jiangxi | 361126 | Yiyang County | 4.2 | 3.3 | 5.0 |  | 45.9(36.3,55.3) | 47.0(36.3,57.8) | 46.6(34.9,58.9) |
| Jiangxi | 360732 | Xingguo County | 5.1 | 4.3 | 5.9 |  | 45.8(36.2,56.5) | 46.0(36.9,55.4) | 46.5(36.4,57.2) |
| Jiangxi | 360828 | Wan'an County | 2.8 | 2.1 | 3.4 |  | 45.4(32.9,59.6) | 42.3(30.7,57.5) | 50.4(33.9,70.7) |
| Jiangxi | 360602 | Yuehu District | 1.8 | 1.2 | 2.3 |  | 42.5(27.8,63.2) | 49.9(30.5,73.5) | 33.5(21.7,54.7) |
| Jiangxi | 361028 | Zixi County | 3.3 | 2.6 | 4.0 |  | 41.1(30.5,54.5) | 46.4(34.0,62.3) | 39.4(29.7,49.9) |
| Jiangxi | 361127 | Yugan County | 4.1 | 3.6 | 4.6 |  | 41.1(32.0,50.4) | 38.7(30.5,47.4) | 46.4(36.3,57.3) |
| Jiangxi | 360881 | Jinggangshan City | 2.6 | 2.0 | 3.3 |  | 38.5(26.7,52.9) | 43.0(29.1,60.5) | 35.1(22.8,54.3) |
| Jiangxi | 360203 | Zhushan District | 1.8 | 1.3 | 2.2 |  | 28.5(13.3,58.9) | 30.4(14.3,62.0) | 27.4(12.7,62.9) |
| Jiangxi | 360521 | Fenyi County | 2.5 | 2.1 | 2.9 |  | 20.6(14.7,28.2) | 20.0(14.0,26.9) | 23.5(15.6,34.2) |
| Jiangxi | 360825 | Yongfeng County | 1.5 | 1.3 | 1.6 |  | 19.5(9.7,37.4) | 18.7(9.6,38.0) | 21.4(10.7,40.6) |
| Shaanxi | 610112 | Weiyang District | 5.1 | 4.2 | 5.9 |  | 92.2(86.1,96.3) | 91.4(84.2,96.1) | 91.8(83.7,96.6) |
| Shaanxi | 610502 | Linwei District | 6.0 | 5.4 | 6.7 |  | 91.2(84.9,95.8) | 89.2(80.7,94.3) | 93.0(85.8,97.1) |
| Shaanxi | 610113 | Yanta District | 4.8 | 3.6 | 5.8 |  | 89.0(82.2,94.4) | 89.6(81.1,95.0) | 85.2(74.9,92.6) |
| Shaanxi | 610111 | Baqiao District | 5.5 | 4.6 | 6.3 |  | 84.5(75.1,91.7) | 88.8(78.4,95.4) | 76.4(63.8,86.4) |
| Shaanxi | 610102 | Xincheng District | 5.6 | 4.8 | 6.3 |  | 83.5(68.7,93.6) | 84.1(69.2,94.6) | 80.0(60.4,93.4) |
| Shaanxi | 610924 | Ziyang County | 8.5 | 6.8 | 10.1 |  | 82.7(73.7,89.5) | 82.6(72.5,90.2) | 79.5(69.7,87.4) |
| Shaanxi | 610923 | Ningshan County | 7.1 | 6.3 | 7.7 |  | 81.6(63.4,93.3) | 75.6(54.8,92.0) | 84.2(65.1,96.7) |
| Shaanxi | 610902 | Hanbin District | 5.2 | 4.3 | 6.1 |  | 80.4(72.1,87.2) | 80.6(71.6,87.8) | 81.3(72.5,88.0) |
| Shaanxi | 610125 | Hu County | 6.2 | 5.5 | 6.8 |  | 79.0(69.7,86.9) | 77.8(67.6,86.7) | 80.9(70.5,89.0) |
| Shaanxi | 610581 | Hancheng City | 6.2 | 4.8 | 7.7 |  | 78.9(69.0,86.2) | 81.8(72.5,88.6) | 76.3(66.1,85.6) |
| Shaanxi | 610116 | Chang'an District | 5.3 | 4.7 | 5.9 |  | 78.8(68.3,86.5) | 75.2(63.3,84.7) | 82.0(72.3,90.2) |
| Shaanxi | 610115 | Lintong District | 6.0 | 5.2 | 6.7 |  | 78.5(69.7,85.8) | 79.7(69.6,87.5) | 78.0(68.0,86.0) |
| Shaanxi | 610925 | Lan'gao County | 8.5 | 6.8 | 9.9 |  | 78.1(67.9,86.0) | 79.4(68.9,88.7) | 74.2(63.8,83.2) |
| Shaanxi | 611024 | Shanyang County | 6.4 | 5.3 | 7.3 |  | 78.0(68.7,85.9) | 78.1(67.7,86.5) | 79.2(69.1,86.8) |
| Shaanxi | 610628 | Fu County | 6.3 | 5.7 | 6.9 |  | 77.6(66.2,85.3) | 75.2(64.1,84.6) | 81.9(71.5,89.3) |
| Shaanxi | 610327 | Long County | 7.1 | 6.7 | 7.4 |  | 76.3(65.4,84.4) | 72.7(60.4,82.2) | 79.8(69.9,87.6) |
| Shaanxi | 610602 | Baota District | 6.0 | 4.6 | 7.3 |  | 75.6(66.2,83.3) | 77.7(67.6,85.1) | 75.6(65.4,84.0) |
| Shaanxi | 611021 | Luonan County | 5.8 | 5.3 | 6.2 |  | 75.1(64.7,83.5) | 71.4(60.9,80.8) | 80.3(70.5,88.4) |
| Shaanxi | 610929 | Baihe County | 7.6 | 5.9 | 9.0 |  | 74.3(51.7,91.3) | 73.3(52.1,90.0) | 75.1(49.4,94.1) |
| Shaanxi | 610204 | Yaozhou District | 5.2 | 4.6 | 5.8 |  | 74.2(62.0,83.9) | 70.4(58.0,81.8) | 78.2(65.2,88.6) |
| Shaanxi | 611002 | Shangzhou District | 5.4 | 4.6 | 6.2 |  | 73.8(63.0,82.6) | 72.1(61.5,80.6) | 76.9(66.3,86.8) |
| Shaanxi | 611026 | Zhashui County | 6.1 | 5.1 | 7.1 |  | 73.8(62.0,84.5) | 78.9(63.5,91.0) | 66.1(54.3,75.9) |
| Shaanxi | 610103 | Beilin District | 5.8 | 5.2 | 6.4 |  | 73.1(62.0,83.2) | 76.8(63.8,86.6) | 66.3(53.7,78.7) |
| Shaanxi | 610104 | Lianhu District | 5.9 | 5.0 | 6.9 |  | 72.6(60.3,83.4) | 80.4(66.4,89.3) | 58.1(45.9,70.7) |
| Shaanxi | 610527 | Baishui County | 5.6 | 5.2 | 6.0 |  | 72.6(62.4,81.3) | 68.8(58.5,78.7) | 78.4(67.0,87.9) |
| Shaanxi | 610402 | Qindu District | 3.6 | 3.0 | 4.2 |  | 72.5(56.8,85.9) | 74.0(54.6,90.5) | 71.4(55.6,88.0) |
| Shaanxi | 610622 | Yanchuan County | 5.3 | 4.6 | 6.0 |  | 72.0(59.9,82.5) | 70.3(57.3,81.6) | 73.1(59.1,85.5) |
| Shaanxi | 610621 | Yanchang County | 6.0 | 5.5 | 6.5 |  | 71.9(60.8,81.6) | 69.2(58.5,80.0) | 78.1(67.6,87.2) |
| Shaanxi | 610730 | Foping County | 6.8 | 4.7 | 8.6 |  | 71.6(44.6,93.6) | 69.7(46.1,94.3) | 62.6(28.8,95.0) |
| Shaanxi | 610523 | Dali County | 6.9 | 6.1 | 7.7 |  | 70.8(61.0,78.7) | 72.7(63.0,81.0) | 69.7(60.0,78.4) |
| Shaanxi | 610928 | Xunyang County | 6.8 | 5.7 | 7.8 |  | 69.5(44.3,86.6) | 69.8(48.3,87.7) | 68.9(45.6,85.7) |
| Shaanxi | 610304 | Chencang District | 6.1 | 5.4 | 6.9 |  | 67.7(57.8,76.7) | 68.5(57.3,77.4) | 70.4(60.2,78.5) |
| Shaanxi | 610431 | Wugong County | 5.8 | 5.3 | 6.2 |  | 66.7(56.7,75.5) | 67.5(57.8,77.0) | 68.9(59.7,77.1) |
| Shaanxi | 610927 | Zhenping County | 9.0 | 6.9 | 10.9 |  | 66.7(55.0,76.8) | 66.0(53.5,76.4) | 63.9(50.2,75.6) |
| Shaanxi | 611025 | Zhen'an County | 6.3 | 5.2 | 7.4 |  | 66.6(54.4,75.7) | 60.4(49.8,70.9) | 73.5(59.7,85.3) |
| Shaanxi | 610526 | Pucheng County | 6.1 | 5.6 | 6.5 |  | 66.3(56.1,74.9) | 67.0(56.2,76.1) | 67.6(58.0,76.5) |
| Shaanxi | 610423 | Jingyang County | 5.6 | 5.0 | 6.2 |  | 65.8(55.5,75.2) | 68.9(56.6,79.7) | 62.5(51.7,72.6) |
| Shaanxi | 611023 | Shangnan County | 5.7 | 4.5 | 6.9 |  | 65.8(53.3,77.7) | 68.6(55.0,80.3) | 64.8(51.0,77.1) |
| Shaanxi | 610629 | Luochuan County | 6.4 | 5.0 | 7.5 |  | 65.7(40.4,84.0) | 68.5(44.6,87.9) | 60.0(37.0,79.6) |
| Shaanxi | 610632 | Huangling County | 6.0 | 5.6 | 6.4 |  | 65.7(53.1,78.3) | 58.2(45.1,71.3) | 74.0(56.9,87.1) |
| Shaanxi | 610522 | Tongguan County | 5.9 | 5.3 | 6.5 |  | 65.5(54.9,75.7) | 67.0(53.9,78.5) | 67.7(57.5,77.3) |
| Shaanxi | 610322 | Fengxiang County | 6.4 | 5.9 | 6.8 |  | 65.3(55.3,74.7) | 62.7(53.1,71.7) | 70.2(60.0,79.3) |
| Shaanxi | 610726 | Ningqiang County | 8.0 | 7.0 | 9.0 |  | 64.8(54.8,74.4) | 64.3(52.8,75.0) | 65.1(54.3,74.3) |
| Shaanxi | 610126 | Gaoling County | 4.7 | 4.0 | 5.4 |  | 64.7(53.5,76.8) | 65.1(52.5,77.5) | 64.6(51.6,77.2) |
| Shaanxi | 610582 | Huayin City | 6.3 | 5.3 | 7.3 |  | 64.4(52.9,75.1) | 66.1(53.7,76.6) | 63.1(51.2,74.7) |
| Shaanxi | 610525 | Chengcheng County | 5.7 | 5.0 | 6.3 |  | 63.8(53.4,73.2) | 62.1(52.3,71.6) | 68.3(57.0,80.3) |
| Shaanxi | 611022 | Danfeng County | 6.1 | 5.6 | 6.5 |  | 63.2(52.7,72.9) | 60.5(50.0,71.9) | 69.3(59.3,78.0) |
| Shaanxi | 610428 | Changwu County | 4.2 | 4.1 | 4.3 |  | 63.1(47.6,77.0) | 50.1(39.4,60.5) | 80.3(59.3,94.4) |
| Shaanxi | 610114 | Yanliang District | 5.0 | 4.4 | 5.5 |  | 62.1(49.0,75.3) | 60.4(45.7,75.9) | 64.8(48.2,80.4) |
| Shaanxi | 610328 | Qianyang County | 6.7 | 6.0 | 7.2 |  | 61.9(50.7,73.2) | 56.5(44.2,66.9) | 70.0(55.8,83.5) |
| Shaanxi | 610404 | Weicheng District | 3.3 | 2.6 | 4.0 |  | 61.5(44.6,80.9) | 67.0(48.1,86.4) | 56.8(40.0,78.1) |
| Shaanxi | 610521 | Hua County | 6.1 | 5.4 | 6.9 |  | 60.9(50.6,71.2) | 63.4(52.2,73.0) | 60.7(49.2,70.4) |
| Shaanxi | 610528 | Fuping County | 6.2 | 5.7 | 6.6 |  | 60.5(50.9,69.9) | 59.2(48.6,69.2) | 64.0(53.5,73.3) |
| Shaanxi | 610203 | Yintai District | 6.2 | 5.3 | 7.0 |  | 59.7(47.4,71.0) | 62.5(49.4,75.1) | 55.6(42.9,68.7) |
| Shaanxi | 610524 | Heyang County | 6.0 | 5.4 | 6.5 |  | 59.7(48.8,69.2) | 59.8(48.7,69.4) | 61.9(50.9,72.4) |
| Shaanxi | 610202 | Wangyi District | 6.5 | 5.7 | 7.2 |  | 59.6(45.6,73.0) | 66.0(50.6,82.7) | 49.4(38.2,60.2) |
| Shaanxi | 610921 | Hanyin County | 7.7 | 6.5 | 8.7 |  | 59.6(39.2,77.5) | 56.7(36.9,76.7) | 61.2(42.2,79.1) |
| Shaanxi | 610922 | Shiquan County | 6.5 | 4.9 | 7.9 |  | 59.5(46.4,72.7) | 63.0(49.3,77.5) | 50.9(37.3,66.2) |
| Shaanxi | 610122 | Lantian County | 5.5 | 5.1 | 5.9 |  | 59.0(48.4,68.9) | 55.5(44.9,66.7) | 64.3(53.0,74.3) |
| Shaanxi | 610826 | Suide County | 5.0 | 4.4 | 5.5 |  | 59.0(48.0,69.2) | 57.5(46.3,69.4) | 62.6(51.2,74.3) |
| Shaanxi | 610926 | Pingli County | 6.4 | 4.8 | 7.8 |  | 58.2(45.3,70.0) | 64.8(50.6,78.4) | 45.5(33.5,58.1) |
| Shaanxi | 610623 | Zichang County | 4.6 | 4.1 | 5.0 |  | 57.8(47.6,68.3) | 56.1(44.5,67.1) | 65.1(53.1,75.3) |
| Shaanxi | 610722 | Chenggu County | 7.8 | 6.4 | 9.2 |  | 57.8(46.5,67.9) | 57.4(45.9,67.6) | 57.6(47.3,68.3) |
| Shaanxi | 610222 | Yijun County | 6.2 | 5.2 | 7.0 |  | 57.6(46.0,68.7) | 56.7(44.6,68.4) | 61.3(48.6,72.8) |
| Shaanxi | 610331 | Taibai County | 5.9 | 5.8 | 6.0 |  | 57.4(43.3,74.4) | 55.4(37.3,78.1) | 63.7(50.0,76.3) |
| Shaanxi | 610124 | Zhouzhi County | 5.4 | 5.1 | 5.8 |  | 57.2(47.4,67.1) | 55.8(44.8,67.5) | 59.8(49.4,69.7) |
| Shaanxi | 610427 | Bin County | 4.7 | 4.5 | 4.9 |  | 57.0(46.6,68.4) | 52.7(41.3,63.8) | 65.7(54.3,76.5) |
| Shaanxi | 610624 | Ansai County | 4.4 | 4.0 | 4.7 |  | 56.9(45.9,66.8) | 56.5(44.7,68.6) | 62.2(51.0,71.9) |
| Shaanxi | 610724 | Xixiang County | 6.3 | 5.1 | 7.5 |  | 55.3(45.3,65.5) | 57.9(46.2,69.2) | 52.5(42,63.6.0) |
| Shaanxi | 610302 | Weibin District | 4.7 | 4.1 | 5.3 |  | 54.4(42.7,65.7) | 46.8(37.4,57.2) | 66.1(53.2,79.6) |
| Shaanxi | 610631 | Huanglong County | 5.3 | 4.6 | 5.9 |  | 54.0(36.1,76.1) | 49.9(32.6,75.8) | 57.3(35.0,85.6) |
| Shaanxi | 610727 | Lueyang County | 6.8 | 6.2 | 7.4 |  | 53.7(42.3,64.8) | 50.7(38.1,62.7) | 57.6(45.4,70.7) |
| Shaanxi | 610330 | Feng County | 6.0 | 5.3 | 6.6 |  | 52.0(39.7,66.4) | 52.2(37.4,68.2) | 52.4(38.1,67.8) |
| Shaanxi | 610326 | Mei County | 6.2 | 5.5 | 6.8 |  | 51.5(41.6,62.3) | 48.8(38.3,59.6) | 55.6(44.4,66.9) |
| Shaanxi | 610630 | Yichuan County | 4.1 | 3.4 | 4.9 |  | 51.2(38.7,65.4) | 53.2(40.7,67.4) | 53.3(38.9,69.4) |
| Shaanxi | 610323 | Qishan County | 6.2 | 5.9 | 6.6 |  | 50.6(30.5,71.4) | 47.1(28.6,68.7) | 56.2(37.2,75.6) |
| Shaanxi | 610721 | Nanzheng County | 7.0 | 5.7 | 8.3 |  | 49.9(38.5,61.3) | 52.0(39.7,63.7) | 45.8(35.0,57.8) |
| Shaanxi | 610329 | Linyou County | 6.0 | 5.9 | 6.0 |  | 48.5(36.5,60.3) | 44.7(31.3,61.6) | 54.9(43.6,67.5) |
| Shaanxi | 610729 | Liuba County | 6.7 | 5.1 | 8.1 |  | 47.9(33.6,66.6) | 53.8(36.1,75.8) | 38.7(24.7,53.1) |
| Shaanxi | 610821 | Shenmu County | 1.0 | 0.7 | 1.3 |  | 47.5(34.4,63.9) | 46.3(32.3,64.3) | 53.3(36.2,73.1) |
| Shaanxi | 610626 | Wuqi County | 3.3 | 2.9 | 3.8 |  | 46.9(35.3,59.4) | 48.5(34.5,63.9) | 49.3(39.2,60.4) |
| Shaanxi | 610723 | Yang County | 6.4 | 5.6 | 7.0 |  | 45.8(36.6,56.6) | 42.0(32.0,52.5) | 51.3(41.5,62.5) |
| Shaanxi | 610725 | Mian County | 5.7 | 4.7 | 6.6 |  | 45.7(34.6,58.4) | 50.2(37.6,62.8) | 40.4(30.1,52.8) |
| Shaanxi | 610303 | Jintai District | 5.1 | 4.5 | 5.7 |  | 45.5(35.7,56.0) | 47.5(36.6,59.2) | 46.4(37.0,58.0) |
| Shaanxi | 610702 | Hantai District | 4.1 | 3.4 | 4.8 |  | 44.3(33.3,57.1) | 42.7(31.0,56.1) | 46.9(34.0,61.3) |
| Shaanxi | 610403 | Yangling District | 1.7 | 1.5 | 1.8 |  | 43.8(29.8,61.8) | 46.7(32.4,73.3) | 46.4(35.7,57.6) |
| Shaanxi | 610728 | Zhenba County | 6.1 | 4.7 | 7.3 |  | 43.6(32.9,55.9) | 50.4(38.0,63.8) | 33.4(24.0,45.3) |
| Shaanxi | 610422 | Sanyuan County | 4.1 | 3.5 | 4.6 |  | 43.5(33.5,53.6) | 44.8(34.2,54.8) | 45.8(34.9,57.7) |
| Shaanxi | 610627 | Ganquan County | 4.3 | 3.4 | 5.0 |  | 42.8(32.8,53.9) | 44.7(33.0,56.6) | 45.3(33.6,57.2) |
| Shaanxi | 610429 | Xunyi County | 2.2 | 1.9 | 2.3 |  | 42.2(29.8,59.4) | 37.3(25.3,52.9) | 51.7(33.4,75.0) |
| Shaanxi | 610425 | Liquan County | 4.0 | 3.8 | 4.2 |  | 39.7(31.0,49.5) | 38.9(30.4,49.1) | 45.0(33.9,55.7) |
| Shaanxi | 610625 | Zhidan County | 2.5 | 2.1 | 2.8 |  | 37.9(27.6,51.1) | 42.0(29.2,60.4) | 36.5(27.8,46.0) |
| Shaanxi | 610324 | Fufeng County | 4.6 | 4.4 | 4.7 |  | 37.3(28.5,46.0) | 34.5(26.1,44.7) | 43.1(32.9,53.4) |
| Shaanxi | 610430 | Chunhua County | 2.7 | 2.2 | 3.2 |  | 37.0(27.6,47.9) | 39.6(28.6,54.4) | 37.6(28.6,47.4) |
| Shaanxi | 610824 | Jingbian County | 0.2 | 0.1 | 0.4 |  | 29.9(20.0,42.4) | 30.0(19.7,46.3) | 33.5(22.9,49.0) |
| Shaanxi | 610426 | Yongshou County | 2.3 | 2.2 | 2.4 |  | 27.7(20.2,36.2) | 27.0(19.5,35.2) | 33.9(25.8,43.0) |
| Shaanxi | 610822 | Fugu County | 0.4 | 0.2 | 0.5 |  | 27.2(17.9,41.0) | 30.5(18.3,50.2) | 27.5(19.8,36.6) |
| Shaanxi | 610424 | Qian County | 2.1 | 2.0 | 2.3 |  | 20.8(14.8,28.0) | 21.2(14.9,30.0) | 22.4(15.5,30.8) |
| Shaanxi | 610825 | Dingbian County | 0.5 | 0.3 | 0.6 |  | 20.4(13.2,31.0) | 23.5(14.5,39.0) | 17.5(11.5,24.7) |
| Shaanxi | 610481 | Xingping City | 1.7 | 1.5 | 1.9 |  | 17.7(12.2,24.9) | 20.3(14.0,28.6) | 16.5(11.9,23.1) |
| Shaanxi | 610823 | Hengshan County | 0.2 | 0.1 | 0.3 |  | 12.6(8.3,20.1) | 11.2(7.0,16.7) | 18.3(11.4,28.7) |
| Shaanxi | 610830 | Qingjian County | 0.4 | 0.3 | 0.5 |  | 11.5(6.7,22.5) | 9.5(6.2,14.5) | 16.8(7.7,42.4) |
| Shaanxi | 610831 | Zizhou County | 0.4 | 0.2 | 0.6 |  | 10.0(5.8,16.7) | 10.9(6.6,18.4) | 10.2(6.0,19.5) |
| Shaanxi | 610802 | Yuyang District | 0.5 | 0.3 | 0.8 |  | 9.1(6.3,13.3) | 9.2(6.3,13.5) | 10.5(7.3,15.3) |
| Shaanxi | 610829 | Wubu County | 0.3 | 0.1 | 0.5 |  | 9.1(5.9,13.6) | 9.5(5.9,14.4) | 10.2(7.0,14.8) |
| Shaanxi | 610828 | Jia County | 0.3 | 0.1 | 0.5 |  | 8.1(5.2,12.5) | 8.5(5.4,13.4) | 8.8(5.7,13.4) |
| Shaanxi | 610827 | Mizhi County | 0.2 | 0.1 | 0.3 |  | 7.9(4.9,12.8) | 8.7(5.2,15.8) | 7.6(5.0,11.0) |
| Gansu | 620102 | Chengguan District | 4.8 | 4.0 | 5.6 |  | 89.9(81.7,95.1) | 88.5(79.6,94.7) | 89.2(79.1,95.5) |
| Gansu | 620302 | Jinchuan District | 5.7 | 4.8 | 6.6 |  | 82.4(69.1,92.7) | 83.3(66.2,93.2) | 80.0(64.9,93.3) |
| Gansu | 620981 | Yumen City | 5.3 | 4.7 | 5.9 |  | 81.8(66.9,92.5) | 79.9(61.8,93.3) | 80.0(60.5,94.2) |
| Gansu | 620104 | Xigu District | 4.8 | 4.3 | 5.3 |  | 81.5(66.8,92.3) | 77.8(60.4,91.7) | 82.7(62.4,94.9) |
| Gansu | 620702 | Ganzhou District | 6.8 | 5.9 | 7.7 |  | 80.1(72.0,86.7) | 77.8(69.0,85.2) | 81.8(73.2,88.5) |
| Gansu | 620982 | Dunhuang City | 5.4 | 4.7 | 6.1 |  | 77.7(64.0,88.7) | 74.1(58.4,88.4) | 80.1(63.5,92.3) |
| Gansu | 620423 | Jingtai County | 6.2 | 5.5 | 6.9 |  | 76.8(64.8,85.5) | 75.1(63.0,85.3) | 75.6(61.1,87.0) |
| Gansu | 620103 | Qilihe District | 4.9 | 4.4 | 5.3 |  | 74.9(62.5,85.4) | 73.4(59.2,85.5) | 73.1(57.9,86.3) |
| Gansu | 620923 | Zizhixian | 4.4 | 4.8 | 4.1 |  | 74.5(38.5,98.8) | 42.5(25.9,61.6) | 92.4(48.9,99.9) |
| Gansu | 620622 | Gulang County | 5.4 | 5.1 | 5.7 |  | 71.6(61.3,79.5) | 70.5(59.1,80.1) | 73.3(62.2,82.1) |
| Gansu | 620402 | Baiyin District | 5.5 | 4.7 | 6.4 |  | 70.0(55.2,83.1) | 71.1(54.8,84.2) | 65.5(47.7,83.4) |
| Gansu | 620602 | Liangzhou District | 5.7 | 5.3 | 6.2 |  | 69.6(59.9,78.1) | 66.4(56.7,74.7) | 74.2(65.3,82.7) |
| Gansu | 620201 | Shixia District | 4.8 | 4.0 | 5.6 |  | 66.0(50.7,80.9) | 70.6(52.4,86.2) | 57.6(42.4,75.1) |
| Gansu | 621025 | Zhengning County | 6.0 | 5.5 | 6.5 |  | 66.0(54.4,76.5) | 64.3(51.6,75.6) | 69.3(59.0,80.1) |
| Gansu | 620105 | Anning District | 3.2 | 2.5 | 3.8 |  | 65.8(39.0,90.0) | 62.1(37.3,87.8) | 70.0(41.3,94.2) |
| Gansu | 620724 | Gaotai County | 6.8 | 5.9 | 7.7 |  | 63.8(52.5,75.2) | 60.9(47.9,73.6) | 68.8(54.7,80.6) |
| Gansu | 621024 | Heshui County | 6.1 | 5.4 | 6.8 |  | 61.1(49.2,71.3) | 57.8(45.2,69.5) | 67.7(53.0,80.1) |
| Gansu | 620321 | Yongchang County | 5.2 | 4.8 | 5.6 |  | 60.7(49.3,71.1) | 57.2(45.8,68.5) | 66.9(53.8,81.1) |
| Gansu | 623021 | Lintan County | 6.5 | 5.6 | 7.3 |  | 60.5(47.7,71.3) | 55.0(43.0,66.2) | 67.7(52.4,81.3) |
| Gansu | 621002 | Xifeng District | 4.2 | 3.7 | 4.7 |  | 59.9(48.8,70.2) | 55.7(45.0,66.3) | 69.8(57.3,80.6) |
| Gansu | 620921 | Jinta County | 5.8 | 4.9 | 6.7 |  | 59.8(47.3,72.1) | 60.3(45.9,74.8) | 59.1(44.8,73.7) |
| Gansu | 620503 | Maiji District | 5.5 | 4.8 | 6.2 |  | 59.7(50.0,68.5) | 57.6(48.0,68.1) | 65.2(55.6,74.6) |
| Gansu | 620621 | Minqin County | 6.6 | 6.3 | 6.9 |  | 58.9(47.0,69.4) | 56.2(42.9,69.7) | 62.6(50.6,73.8) |
| Gansu | 620403 | Pingchuan District | 3.7 | 3.2 | 4.2 |  | 57.8(44.1,72.6) | 49.6(37.7,63.8) | 71.3(51.0,88.7) |
| Gansu | 621102 | Anding District | 6.4 | 5.6 | 7.1 |  | 57.6(46.9,67.8) | 51.8(41.7,62.5) | 64.5(51.7,75.9) |
| Gansu | 620111 | Honggu District | 4.5 | 3.7 | 5.2 |  | 56.7(43.2,71.1) | 62.5(46.2,80.6) | 50.7(39.2,61.8) |
| Gansu | 621226 | Li County | 6.3 | 5.6 | 6.9 |  | 56.2(45.8,66.0) | 54.5(44.2,63.8) | 60.3(49.0,70.5) |
| Gansu | 620723 | Linze County | 6.2 | 5.0 | 7.3 |  | 56.0(45.3,66.8) | 58.6(45.8,70.2) | 54.7(42.9,66.7) |
| Gansu | 620822 | Lingtai County | 6.3 | 5.0 | 7.5 |  | 55.6(45.0,66.4) | 60.7(48.4,74.1) | 47.8(37.6,59.7) |
| Gansu | 621023 | Huachi County | 5.2 | 4.5 | 5.9 |  | 55.3(43.9,66.6) | 55.3(43.1,67.6) | 59.6(47.7,70.8) |
| Gansu | 621124 | Lintao County | 6.3 | 5.4 | 7.3 |  | 55.3(45.0,64.7) | 54.2(44.3,63.7) | 57.2(46.7,67.9) |
| Gansu | 620725 | Shandan County | 5.4 | 4.7 | 6.1 |  | 55.2(44.3,65.9) | 54.0(41.7,65.1) | 59.6(45.5,73.5) |
| Gansu | 620821 | Jingchuan County | 6.8 | 6.0 | 7.6 |  | 54.1(43.7,64.5) | 50.1(38.8,61.5) | 61.2(48.5,72.3) |
| Gansu | 621021 | Qingcheng County | 5.4 | 4.8 | 6.1 |  | 53.9(44.0,65.2) | 52.0(41.0,63.2) | 59.4(47.1,71.2) |
| Gansu | 620922 | Guazhou County | 4.1 | 3.1 | 4.9 |  | 53.8(30.3,78.8) | 55.3(32.1,82.4) | 46.1(24.4,74.9) |
| Gansu | 620824 | Huating County | 4.6 | 4.0 | 5.1 |  | 53.2(41.6,64.6) | 50.6(39.3,61.8) | 60.0(45.9,74.2) |
| Gansu | 620825 | Zhuanglang County | 6.0 | 4.9 | 7.2 |  | 52.8(42.8,61.6) | 52.9(43.5,62.6) | 56.7(46.3,66.9) |
| Gansu | 620421 | Jingyuan County | 4.5 | 4.2 | 4.8 |  | 52.7(42.2,62.6) | 50.8(40.0,62.3) | 56.5(45.1,68.2) |
| Gansu | 620722 | Minle County | 5.5 | 4.8 | 6.1 |  | 52.7(42.5,63.5) | 54.1(43.2,64.6) | 53.2(41.8,64.2) |
| Gansu | 622923 | Yongjing County | 5.4 | 4.7 | 6.1 |  | 52.7(40.0,64.5) | 47.9(35.2,62.0) | 57.5(42.4,72.9) |
| Gansu | 620524 | Wushan County | 5.3 | 4.7 | 5.9 |  | 52.2(41.9,62.4) | 48.5(37.6,58.6) | 60.0(49.1,69.4) |
| Gansu | 620823 | Chongxin County | 5.6 | 4.6 | 6.5 |  | 51.0(40.1,62.5) | 52.6(42.2,64.2) | 52.4(40.7,65.4) |
| Gansu | 623024 | Teewo County | 6.0 | 5.1 | 6.9 |  | 51.0(37.5,66.2) | 48.2(35.8,61.8) | 55.3(35.3,77.9) |
| Gansu | 621027 | Zhenyuan County | 6.1 | 5.5 | 6.7 |  | 50.8(41.1,60.8) | 47.8(38.4,58.1) | 56.4(45.9,66.1) |
| Gansu | 620721 | Zizhixian | 5.4 | 5.6 | 5.2 |  | 49.3(37.3,61.1) | 43.4(31.1,57.1) | 61.1(45.6,75.7) |
| Gansu | 621121 | Tongwei County | 4.8 | 4.0 | 5.6 |  | 49.3(38.2,61.6) | 46.4(35.2,58.1) | 54.0(39.7,68.6) |
| Gansu | 621227 | Hui County | 5.3 | 4.6 | 6.0 |  | 47.0(36.0,58.2) | 44.1(33.2,56.9) | 51.8(39.2,64.7) |
| Gansu | 621225 | Xihe County | 4.7 | 4.1 | 5.2 |  | 45.7(28.0,65.2) | 43.7(27.3,63.8) | 52.2(33.9,72.4) |
| Gansu | 621223 | Dangchang County | 4.7 | 3.9 | 5.5 |  | 45.2(35.7,56.0) | 45.3(35.4,55.7) | 46.2(34.8,59.0) |
| Gansu | 621026 | Ning County | 4.8 | 4.2 | 5.5 |  | 45.1(35.2,55.7) | 43.6(34.3,54.3) | 50.3(39.3,61.5) |
| Gansu | 621221 | Cheng County | 5.9 | 5.1 | 6.8 |  | 44.8(35.1,55.1) | 43.9(34.1,54.7) | 47.7(37.1,59.4) |
| Gansu | 620826 | jingning County | 5.2 | 4.7 | 5.8 |  | 44.4(34.3,54.2) | 40.7(31.9,51.3) | 49.9(38.2,61.3) |
| Gansu | 622922 | Kangle County | 4.4 | 3.8 | 4.9 |  | 43.0(34.0,53.2) | 43.4(33.0,56.2) | 43.5(32.7,55.7) |
| Gansu | 621022 | Huan County | 4.2 | 3.8 | 4.7 |  | 42.7(34.1,52.9) | 43.0(33.4,53.3) | 45.8(35.7,56.2) |
| Gansu | 620623 | Zizhixian | 5.3 | 4.6 | 5.9 |  | 42.3(32.3,54.0) | 42.5(32.1,54.4) | 43.0(31.8,56.5) |
| Gansu | 620802 | Kongtong District | 4.9 | 4.3 | 5.5 |  | 42.2(33.8,52.7) | 39.5(31.1,49.4) | 49.0(39.1,59.8) |
| Gansu | 620122 | Gaolan County | 4.8 | 4.3 | 5.3 |  | 41.5(31.1,54.1) | 39.1(27.8,52.5) | 47.1(33.4,63.6) |
| Gansu | 620123 | Yuzhong County | 4.6 | 4.2 | 5.0 |  | 39.7(30.3,49.1) | 38.1(28.0,49.4) | 43.6(33.5,54.3) |
| Gansu | 621123 | Weiyuan County | 4.3 | 3.8 | 4.8 |  | 37.2(28.1,46.6) | 36.3(26.9,46.9) | 39.4(29.0,51.7) |
| Gansu | 621228 | Liangdang County | 5.6 | 4.5 | 6.6 |  | 36.6(25.3,48.8) | 38.4(26.2,50.9) | 35.2(21.5,49.0) |
| Gansu | 620422 | Huining County | 4.1 | 3.6 | 4.6 |  | 36.5(28.1,45.9) | 35.6(27.2,44.6) | 41.5(31.9,52.5) |
| Gansu | 621202 | Wudu District | 3.4 | 2.8 | 3.9 |  | 35.1(26.5,44.0) | 35.0(26.8,45.3) | 38.5(28.6,48.9) |
| Gansu | 620121 | Yongdeng County | 4.1 | 3.6 | 4.6 |  | 34.9(26.7,44.5) | 34.6(26.3,43.4) | 37.9(27.3,47.9) |
| Gansu | 621122 | Longxi County | 3.9 | 3.3 | 4.4 |  | 34.4(26.2,43.3) | 31.8(24.6,40.4) | 40.7(30.6,52.5) |
| Gansu | 621224 | Kang County | 4.1 | 3.5 | 4.6 |  | 33.0(24.0,45.1) | 28.7(20.7,38.5) | 38.5(25.4,56.6) |
| Gansu | 623026 | Luqu County | 3.8 | 3.7 | 3.9 |  | 31.6(18.6,50.0) | 28.8(15.8,50.3) | 33.5(18.7,56.1) |
| Gansu | 620521 | Qingshui County | 3.0 | 2.4 | 3.7 |  | 31.5(23.6,41.1) | 30.3(22.9,39.4) | 37.3(27.7,48.2) |
| Gansu | 623027 | Xiahe County | 4.4 | 4.6 | 4.3 |  | 29.6(14.4,54.5) | 23.6(11.6,45.2) | 38.7(18.8,70.6) |
| Gansu | 623023 | Zhugqu County | 3.9 | 3.4 | 4.5 |  | 27.5(19.6,37.5) | 28.7(20.0,40.3) | 26.7(17.7,37.5) |
| Gansu | 620902 | Suzhou District | 2.3 | 1.8 | 2.7 |  | 23.9(17.3,32.1) | 24.9(18.1,33.1) | 26.6(19.2,37.0) |
| Gansu | 620502 | Qinzhou District | 2.3 | 1.8 | 2.7 |  | 23.3(16.7,30.3) | 24.0(17.6,32.1) | 24.6(18.0,32.4) |
| Gansu | 620924 | Zizhixian | 1.9 | 1.2 | 2.5 |  | 23.0(13.7,35.7) | 25.0(13.8,41.4) | 25.3(14.7,42.5) |
| Gansu | 623001 | Hezuo City | 3.3 | 3.0 | 3.5 |  | 21.8(11.9,39.2) | 21.5(11.6,38.5) | 24.3(12.8,45.1) |
| Gansu | 620522 | Qin'an County | 2.3 | 2.0 | 2.5 |  | 19.3(13.7,26.1) | 17.8(12.9,24.3) | 24.7(18.3,31.8) |
| Gansu | 622901 | Linxia City | 2.1 | 1.6 | 2.6 |  | 18.7(13.0,26.4) | 20.4(13.8,29.1) | 18.7(12.5,26.3) |
| Gansu | 621126 | Min County | 1.3 | 1.1 | 1.5 |  | 15.7(11.5,21.9) | 16.7(11.8,22.9) | 16.6(11.9,22.7) |
| Gansu | 620523 | Gan'gu County | 1.6 | 1.4 | 1.9 |  | 15.3(10.8,21.4) | 13.7(9.7,19.0) | 20.2(14.7,26.9) |
| Gansu | 623022 | Jonee County | 3.7 | 3.2 | 4.1 |  | 14.6(8.9,23.8) | 16.3(9.1,26.5) | 14.3(7.9,23.3) |
| Gansu | 620525 | Zizhixian | 1.7 | 1.4 | 2.0 |  | 14.5(8.1,26.0) | 13.4(7.5,22.3) | 18.4(9.9,32.4) |
| Gansu | 621125 | Zhang County | 2.3 | 2.0 | 2.6 |  | 13.5(9.5,18.2) | 13.4(9.8,18.3) | 15.7(11.4,21.6) |
| Gansu | 621222 | Wen County | 2.6 | 2.0 | 3.1 |  | 12.9(9.1,18.2) | 14.4(9.7,20.7) | 12.2(8.5,17.4) |
| Gansu | 622925 | Hezheng County | 1.6 | 1.3 | 1.9 |  | 12.4(8.2,17.8) | 12.1(8.2,18.3) | 13.8(8.9,20.5) |
| Gansu | 623025 | Maqu County | 2.3 | 2.2 | 2.4 |  | 11.9(8.1,17.9) | 10.2(6.8,15.5) | 16.2(9.9,27.2) |
| Gansu | 622921 | Linxia County | 1.2 | 1.0 | 1.5 |  | 10.5(7.0,15.5) | 10.4(7.0,15.5) | 11.9(7.7,18.1) |
| Gansu | 622927 | Dongxiangzu Salarzu | 0.8 | 0.6 | 1.0 |  | 10.0(6.8,14.6) | 9.5(6.5,13.9) | 12.7(8.3,19.6) |
| Gansu | 622924 | Guanghe County | 0.3 | 0.2 | 0.3 |  | 6.6(4.5,9.8) | 6.7(4.2,10.5) | 7.4(4.9,11.0) |
| Gansu | 622926 | Dongxiangzu Zizhixian | 0.3 | 0.2 | 0.3 |  | 5.4(2.8,9.7) | 4.8(2.4,9.5) | 6.6(3.4,12.5) |
| Hainan | 460108 | Meilan District | 3.4 | 2.9 | 4.0 |  | 83.0(71.8,91.4) | 80.4(67.7,89.7) | 86.8(76.0,94.9) |
| Hainan | 469006 | Wanning City | 5.7 | 4.4 | 7.1 |  | 72.4(62.9,80.0) | 78.3(69.4,85.1) | 68.3(57.3,77.1) |
| Hainan | 469002 | Qionghai City | 5.6 | 4.5 | 6.7 |  | 68.6(59.0,77.7) | 74.4(64.9,82.7) | 63.1(51.2,73.4) |
| Hainan | 460107 | Qiongshan District | 2.1 | 1.6 | 2.6 |  | 67.1(53.2,79.2) | 69.1(54.4,81.6) | 66.7(51.8,82.7) |
| Hainan | 460106 | Longhua District | 1.8 | 1.4 | 2.3 |  | 61.0(46.8,74.7) | 69.4(54.6,82.9) | 55.0(41.5,68.7) |
| Hainan | 460105 | Xiuying District | 1.8 | 1.4 | 2.3 |  | 59.0(44.6,74.6) | 67.5(50.3,83.9) | 51.7(37.5,67.7) |
| Hainan | 469001 | Wuzhishan City | 4.4 | 2.7 | 6.2 |  | 57.1(42.0,74.5) | 65.0(49.4,78.9) | 47.8(30.3,70.2) |
| Hainan | 469028 | Lingshui Lizu Zizhixian | 3.4 | 2.2 | 4.6 |  | 54.5(37.8,70.3) | 60.9(46.2,75.5) | 48.4(31.0,68.8) |
| Hainan | 469021 | Ding'an County | 4.4 | 3.3 | 5.5 |  | 51.4(41.0,62.5) | 62.6(51.0,73.9) | 42.2(32.1,53.4) |
| Hainan | 460201 | sanya shi | 2.5 | 1.8 | 3.2 |  | 45.2(33.4,59.3) | 51.7(39.2,66.1) | 44.5(32.0,58.1) |
| Hainan | 469026 | Zizhixian | 3.8 | 2.8 | 4.8 |  | 43.8(32.8,54.7) | 53.1(40.8,67.0) | 35.4(25.2,48.7) |
| Hainan | 469025 | Baisha Lizu Zizhixian | 4.2 | 2.8 | 5.6 |  | 41.7(32.0,52.1) | 51.3(40.3,62.1) | 33.6(23.7,47.2) |
| Hainan | 469022 | Tunchang County | 3.2 | 2.2 | 4.2 |  | 40.0(30.0,51.4) | 48.6(37.0,60.3) | 34.8(25.3,46.2) |
| Hainan | 469007 | Dongfang City | 2.6 | 2.0 | 3.3 |  | 39.5(30.0,49.5) | 45.0(35.1,56.2) | 39.4(29.7,50.9) |
| Hainan | 469030 | Zizhixian | 4.5 | 3.0 | 6.0 |  | 39.2(30.0,49.0) | 51.7(39.8,62.7) | 28.6(20.2,39.7) |
| Hainan | 469027 | Ledong Lizu Zizhixian | 2.8 | 2.0 | 3.7 |  | 38.2(27.4,51.7) | 49.8(36.8,63.5) | 32.2(21.5,45.0) |
| Hainan | 469029 | Zizhixian | 3.3 | 2.5 | 4.1 |  | 32.5(22.4,43.3) | 38.4(27.5,52.0) | 29.2(19.0,41.8) |
| Hainan | 469023 | Chengmai County | 2.7 | 2.1 | 3.3 |  | 31.7(23.0,42.5) | 35.3(25.5,47.7) | 30.8(21.9,44.1) |
| Hainan | 469024 | Lingao County | 1.8 | 1.3 | 2.3 |  | 24.5(17.4,32.5) | 27.0(20.0,34.6) | 25.6(18.5,34.4) |
| Hainan | 469003 | Danzhou City | 1.4 | 1.0 | 1.9 |  | 22.9(16.3,30.4) | 27.3(19.9,36.2) | 21.2(15.2,29.2) |
| Hainan | 469005 | Wenchang City | 1.7 | 1.4 | 2.1 |  | 22.2(15.9,30.8) | 29.3(20.0,41.9) | 18.2(12.8,25.4) |
| Guizhou | 520121 | Kaiyang County | 7.5 | 6.4 | 8.5 |  | 97.0(93.5,99.0) | 96.3(91.8,98.7) | 96.4(91.5,98.8) |
| Guizhou | 520102 | Nanming District | 4.0 | 3.5 | 4.5 |  | 95.3(89.1,98.4) | 94.4(87.2,98.0) | 94.8(87.9,98.4) |
| Guizhou | 520621 | Jiangkou County | 6.1 | 5.1 | 7.1 |  | 95.2(88.2,98.7) | 95.7(87.7,99.2) | 92.8(79.9,98.2) |
| Guizhou | 520381 | Chishui City | 9.1 | 7.9 | 10.1 |  | 93.6(84.7,98.0) | 90.2(78.7,96.4) | 95.9(87.9,99.2) |
| Guizhou | 520181 | Qingzhen City | 6.0 | 5.0 | 6.9 |  | 92.8(86.8,96.9) | 91.1(83.3,95.9) | 92.5(85.6,96.9) |
| Guizhou | 520402 | Xixiu District | 6.7 | 5.7 | 7.7 |  | 91.3(79.4,97.3) | 91.9(80.8,97.5) | 87.9(74.2,95.5) |
| Guizhou | 522327 | Ceheng County | 7.0 | 5.6 | 8.3 |  | 90.4(81.1,95.8) | 88.8(78.7,95.2) | 88.0(75.5,95.6) |
| Guizhou | 520330 | Xishui County | 7.4 | 6.4 | 8.3 |  | 89.2(82.5,94.1) | 88.4(80.9,94.1) | 89.3(82.2,94.3) |
| Guizhou | 520521 | Dafang County | 6.2 | 5.0 | 7.4 |  | 89.0(81.4,94.3) | 90.6(83.6,95.3) | 83.6(73.5,90.8) |
| Guizhou | 520122 | Xifeng County | 6.6 | 5.8 | 7.3 |  | 88.7(80.7,94.4) | 86.3(75.3,93.3) | 90.2(80.3,96.1) |
| Guizhou | 520111 | Huaxi District | 5.6 | 4.6 | 6.6 |  | 86.3(77.5,92.2) | 85.3(76.7,92.0) | 86.3(76.6,93.1) |
| Guizhou | 520502 | Bijie City | 4.7 | 4.0 | 5.3 |  | 84.9(75.4,91.8) | 86.1(76.6,93.0) | 79.6(69.4,87.7) |
| Guizhou | 520603 | Wanshan Tequ | 6.2 | 5.3 | 7.1 |  | 84.8(72.0,93.8) | 85.8(71.5,94.9) | 80.4(63.0,92.8) |
| Guizhou | 520622 | Zizhixian | 7.1 | 6.1 | 8.0 |  | 84.6(73.4,93.1) | 82.3(69.6,91.7) | 86.1(74.1,95.2) |
| Guizhou | 520203 | Luzhi Tequ | 7.3 | 6.0 | 8.5 |  | 84.3(75.6,90.3) | 85.9(78.7,91.9) | 80.5(70.8,88.1) |
| Guizhou | 522730 | Longli County | 8.4 | 6.8 | 9.9 |  | 84.3(75.4,91.0) | 85.9(77.1,92.9) | 80.4(68.6,89.0) |
| Guizhou | 522702 | Fuquan City | 7.0 | 5.7 | 8.2 |  | 84.0(75.5,90.2) | 85.5(76.2,92.6) | 78.9(66.9,87.8) |
| Guizhou | 520221 | Shuicheng County | 6.4 | 4.9 | 7.9 |  | 82.5(73.2,89.3) | 84.2(75.6,91.0) | 76.6(65.0,85.6) |
| Guizhou | 522632 | Rongjiang County | 7.1 | 6.2 | 7.9 |  | 78.6(61.4,90.0) | 78.8(62.4,90.4) | 77.1(60.0,89.5) |
| Guizhou | 522625 | Zhenyuan County | 6.3 | 5.6 | 7.1 |  | 78.1(66.5,86.9) | 78.5(66.4,87.8) | 77.3(64.7,87.2) |
| Guizhou | 520222 | Pan County | 5.7 | 4.8 | 6.6 |  | 77.5(65.0,86.8) | 78.5(65.7,89.3) | 74.1(61.8,84.3) |
| Guizhou | 520123 | Xiuwen County | 5.7 | 4.8 | 6.6 |  | 77.2(57.5,90.7) | 78.1(60.2,92.1) | 75.1(55.1,91.3) |
| Guizhou | 522623 | Shibing County | 7.4 | 6.8 | 8.0 |  | 77.1(66.3,86.0) | 73.8(61.5,84.6) | 81.1(68.0,90.4) |
| Guizhou | 520201 | Zhongshan District | 4.7 | 3.4 | 5.9 |  | 75.7(62.3,87.1) | 83.2(70.2,91.8) | 59.4(45.0,73.3) |
| Guizhou | 522726 | Dushan County | 7.1 | 6.2 | 8.0 |  | 75.5(65.3,84.1) | 76.3(64.5,85.4) | 73.8(62.3,83.2) |
| Guizhou | 520422 | Puding County | 5.4 | 4.6 | 6.1 |  | 73.1(61.8,82.8) | 74.4(62.2,85.1) | 69.6(56.7,80.3) |
| Guizhou | 520624 | Sinan County | 6.6 | 5.6 | 7.5 |  | 71.3(60.3,79.9) | 74.8(63.8,83.5) | 66.6(55.6,75.8) |
| Guizhou | 520113 | Baiyun District | 4.4 | 3.5 | 5.4 |  | 71.2(58.6,82.4) | 72.2(58.6,84.3) | 68.3(52.2,82.9) |
| Guizhou | 520328 | Meitan County | 6.0 | 5.4 | 6.5 |  | 70.5(59.4,80.7) | 69.3(57.4,79.5) | 72.1(60.3,81.7) |
| Guizhou | 520628 | Zizhixian | 4.2 | 3.4 | 5.0 |  | 70.5(46.7,88.8) | 75.1(51.8,93.0) | 60.9(39.9,82.8) |
| Guizhou | 520523 | Jinsha County | 6.4 | 5.5 | 7.3 |  | 70.0(53.8,83.4) | 71.5(55.0,84.5) | 68.7(52.5,83.5) |
| Guizhou | 520423 | Miaozu Zizhixian | 5.8 | 4.7 | 6.9 |  | 67.5(56.4,76.8) | 68.9(58.0,78.9) | 65.8(53.0,78.0) |
| Guizhou | 520303 | Huichuan District | 4.9 | 4.4 | 5.4 |  | 65.5(46.3,81.9) | 64.3(47.2,82.9) | 67.6(49.3,82.1) |
| Guizhou | 520602 | Tongren City | 5.4 | 4.1 | 6.5 |  | 64.5(52.4,74.9) | 73.8(61.6,84.2) | 50.2(39.5,62.8) |
| Guizhou | 522636 | Danzhai County | 6.1 | 4.8 | 7.3 |  | 62.5(50.1,75.3) | 63.8(51.2,75.3) | 61.4(44.1,77.5) |
| Guizhou | 520103 | Yunyan District | 2.5 | 2.1 | 2.9 |  | 61.6(47.4,74.0) | 63.3(49.9,76.1) | 58.4(45.3,71.6) |
| Guizhou | 520112 | Wudang District | 4.3 | 3.5 | 5.1 |  | 61.5(49.3,73.2) | 61.3(48.1,74.3) | 62.7(47.0,78.2) |
| Guizhou | 522629 | Jianhe County | 4.9 | 4.0 | 5.7 |  | 59.7(40.7,79.7) | 68.1(46.8,87.0) | 49.6(32.4,68.7) |
| Guizhou | 522324 | Qinglong County | 4.2 | 3.3 | 5.1 |  | 59.5(44.5,73.5) | 68.6(53.0,82.7) | 43.8(31.7,58.0) |
| Guizhou | 522301 | Xingyi City | 5.0 | 4.4 | 5.6 |  | 58.5(48.0,68.9) | 58.5(47.2,69.9) | 57.5(47.6,68.7) |
| Guizhou | 520302 | Honghuagang District | 4.1 | 3.5 | 4.6 |  | 54.9(38.1,74.2) | 54.7(37.1,73.3) | 56.6(38.7,73.8) |
| Guizhou | 522601 | Kaili City | 5.0 | 4.1 | 5.9 |  | 54.4(43.3,64.8) | 56.6(45.0,67.5) | 51.8(40.3,63.2) |
| Guizhou | 520329 | Yuqing County | 3.9 | 3.6 | 4.2 |  | 44.3(33.4,56.2) | 45.5(32.6,62.1) | 43.7(31.3,56.5) |
| Guizhou | 522634 | Leishan County | 3.8 | 3.1 | 4.5 |  | 41.6(24.9,62.4) | 39.4(25.7,59.3) | 44.1(25.1,70.0) |
| Guizhou | 522635 | Majiang County | 3.9 | 3.6 | 4.2 |  | 37.1(22.8,56.3) | 37.5(22.2,56.9) | 39.9(25.0,60.5) |
| Guizhou | 522622 | Huangping County | 3.8 | 3.1 | 4.4 |  | 33.4(24.5,43.7) | 36.9(26.5,49.2) | 31.0(21.8,41.6) |
| Guizhou | 520322 | Tongzi County | 3.0 | 2.5 | 3.4 |  | 29.8(21.9,39.4) | 32.7(24.0,43.0) | 28.8(21.2,38.2) |
| Guizhou | 522722 | Libo County | 3.2 | 2.7 | 3.6 |  | 27.1(18.1,38.8) | 29.8(19.4,44.3) | 24.5(16.0,37.2) |
| Guizhou | 522328 | Anlong County | 2.1 | 1.8 | 2.3 |  | 25.5(16.9,36.7) | 23.6(15.6,35.5) | 28.5(17.7,42.5) |
| Guizhou | 520323 | Suiyang County | 2.3 | 2.1 | 2.6 |  | 24.1(13.9,38.4) | 23.7(14.2,40.3) | 24.6(15.1,39.6) |
| Guizhou | 520522 | Qianxi County | 0.8 | 0.7 | 1.0 |  | 16.5(8.4,32.9) | 16.7(8.5,34.4) | 17.2(8.5,34.7) |
| Guizhou | 522701 | Duyun City | 2.3 | 2.1 | 2.4 |  | 16.3(11.6,22.4) | 15.5(10.6,22.1) | 18.6(12.5,26.1) |
| Guizhou | 520625 | Zizhixian | 1.1 | 0.7 | 1.4 |  | 15.5(10.6,22.7) | 16.3(11.3,23.7) | 16.5(10.7,25.2) |
| Guizhou | 520627 | Yanhe Tujiazu Zizhixian | 1.6 | 1.4 | 1.8 |  | 15.0(10.1,21.4) | 15.2(10.1,22.5) | 16.2(11.1,23.3) |
| Guizhou | 522624 | Sansui County | 0.7 | 0.6 | 0.8 |  | 11.0(6.8,19.0) | 9.8(6.2,16.7) | 13.4(7.6,25.3) |
| Guizhou | 520382 | Renhuai City | 1.1 | 0.8 | 1.3 |  | 10.4(7.0,15.6) | 12.3(8.1,18.7) | 9.2(6.1,13.5) |
| Guizhou | 522325 | Zhenfeng County | 1.3 | 1.0 | 1.5 |  | 10.1(6.7,15.1) | 11.8(7.4,18.4) | 9.1(5.8,13.3) |
| Guizhou | 520421 | Pingba County | 0.4 | 0.3 | 0.4 |  | 9.7(6.3,14.7) | 9.3(5.9,14.7) | 11.4(7.3,17.8) |
| Guizhou | 522628 | Jinping County | 0.2 | 0.2 | 0.2 |  | 9.6(4.9,19.4) | 10.3(5.0,23.2) | 9.8(5.1,18.0) |
| Guizhou | 522630 | Taijiang County | 0.4 | 0.3 | 0.4 |  | 9.1(5.4,14.9) | 7.8(4.9,12.9) | 11.7(6.5,24.1) |
| Guizhou | 520524 | Zhijin County | 0.1 | 0.1 | 0.1 |  | 9.0(5.9,14.0) | 9.3(5.9,14.3) | 9.6(6.3,14.9) |
| Guizhou | 520623 | Shiqian County | 0.2 | 0.2 | 0.2 |  | 8.9(5.7,14.0) | 8.0(5.2,12.6) | 11.8(7.4,19.5) |
| Guizhou | 520324 | Zheng'an County | 0.2 | 0.1 | 0.2 |  | 8.6(5.6,13.1) | 8.5(5.3,13.4) | 9.2(5.9,14.4) |
| Guizhou | 522732 | Sandu Shuizu Zizhixian | 0.2 | 0.1 | 0.3 |  | 8.6(5.6,13.5) | 9.9(6.1,16.1) | 8.4(5.4,12.6) |
| Guizhou | 520525 | Nayong County | 0.1 | 0.0 | 0.1 |  | 8.5(5.5,12.9) | 9.8(6.1,15.3) | 8.0(5.4,11.8) |
| Guizhou | 520626 | Dejiang County | 1.0 | 0.9 | 1.1 |  | 8.4(5.7,12.0) | 8.9(6.2,12.8) | 9.2(6.3,13.3) |
| Guizhou | 520424 | Miaozu Zizhixian | 0.1 | 0.1 | 0.2 |  | 8.1(5.1,12.7) | 8.8(5.2,14.6) | 7.9(5.0,12.7) |
| Guizhou | 520425 | Zizhixian | 0.1 | 0.1 | 0.2 |  | 8.0(5.0,12.2) | 8.3(5.3,13.0) | 8.7(5.8,13.6) |
| Guizhou | 522322 | Xingren County | 0.1 | 0.0 | 0.1 |  | 7.3(4.6,12.0) | 8.1(4.9,13.3) | 7.6(4.9,11.5) |
| Guizhou | 522626 | Cen'gong County | 0.2 | 0.1 | 0.3 |  | 7.2(3.8,15.6) | 8.2(4.1,16.5) | 6.8(3.5,12.7) |
| Guizhou | 520527 | Hezhang County | 0.1 | 0.0 | 0.1 |  | 7.1(4.5,11.2) | 7.2(4.5,11.1) | 7.8(5.0,12.0) |
| Guizhou | 522731 | Huishui County | 0.3 | 0.2 | 0.5 |  | 6.9(3.5,13.2) | 7.1(3.9,14.2) | 7.0(3.8,13.9) |
| Guizhou | 522631 | Liping County | 0.1 | 0.1 | 0.2 |  | 6.8(4.5,10.9) | 7.0(4.3,11.3) | 7.2(4.7,11.4) |
| Guizhou | 520326 | Miaozu Zizhixian | 0.1 | 0.1 | 0.1 |  | 6.7(4.4,10.3) | 6.9(4.4,10.4) | 7.3(4.9,11.6) |
| Guizhou | 522729 | Changshun County | 0.1 | 0.0 | 0.2 |  | 6.6(4.1,10.3) | 6.9(4.2,10.3) | 7.2(4.6,11.2) |
| Guizhou | 520327 | Fenggang County | 0.1 | 0.1 | 0.2 |  | 6.5(4.3,10.3) | 6.8(4.3,10.2) | 7.5(4.8,11.2) |
| Guizhou | 522627 | Tianzhu County | 0.2 | 0.1 | 0.3 |  | 6.5(4.2,10.2) | 7.1(4.5,11.9) | 6.3(4.1,9.7) |
| Guizhou | 520325 | Miaozu Zizhixian | 0.2 | 0.1 | 0.2 |  | 6.3(4.1,9.4) | 6.4(4.1,9.6) | 7.1(4.6,10.9) |
| Guizhou | 520526 | Miaozu Zizhixian | 0.1 | 0.0 | 0.2 |  | 6.3(3.9,9.8) | 7.0(4.2,11) | 6.5(4.1,9.9) |
| Guizhou | 522323 | Pu'an County | 0.1 | 0.1 | 0.1 |  | 6.0(3.9,9.3) | 6.4(4.1,10.3) | 6.4(4.2,9.4) |
| Guizhou | 520321 | Zunyi County | 0.1 | 0.1 | 0.2 |  | 5.8(3.7,9.0) | 5.9(3.8,9.1) | 6.3(4.0,9.9) |
| Guizhou | 522725 | Weng'an County | 0.1 | 0.1 | 0.1 |  | 5.5(3.5,8.5) | 5.8(3.5,9.6) | 5.8(3.8,8.7) |
| Guizhou | 522633 | Congjiang County | 0.1 | 0.1 | 0.1 |  | 5.3(2.7,9.9) | 5.4(2.9,10.5) | 5.8(3.1,10.3) |
| Guizhou | 522326 | Wangmo County | 0.0 | 0.0 | 0.1 |  | 5.0(3.2,8.0) | 5.3(3.4,7.9) | 5.5(3.5,8.3) |
| Guizhou | 522723 | Guiding County | 0.1 | 0.1 | 0.1 |  | 4.2(2.6,6.6) | 4.4(2.8,6.7) | 4.5(2.9,7.1) |
| Guizhou | 522728 | Luodian County | 0.1 | 0.0 | 0.1 |  | 4.2(2.8,6.4) | 4.6(2.9,7.3) | 4.3(2.8,6.7) |
| Guizhou | 522727 | Pingtang County | 0.1 | 0.1 | 0.1 |  | 3.8(2.3,6.2) | 4.1(2.5,6.5) | 4.1(2.6,6.3) |
| Xinjiang | 652925 | Xinhe County | 6.9 | 5.8 | 8.0 |  | 92.7(86.1,96.9) | 91.3(82.6,96.5) | 93.8(86.7,97.2) |
| Xinjiang | 653221 | Hotan County | 6.9 | 5.7 | 8.0 |  | 88.4(79.1,94.1) | 86.4(76.1,93.8) | 88.8(80.5,94.4) |
| Xinjiang | 652926 | Baicheng County | 5.8 | 4.8 | 6.8 |  | 83.6(70.9,91.4) | 81.0(68.5,90.4) | 86.7(76.3,93.5) |
| Xinjiang | 652301 | Changji City | 4.1 | 3.5 | 4.7 |  | 81.3(66.8,91.0) | 72.6(56.7,86.2) | 88.5(75.9,96.5) |
| Xinjiang | 653125 | Shache(Yarkant) County | 6.2 | 5.4 | 7.1 |  | 78.4(68.8,86.2) | 72.9(61.7,82.3) | 85.2(76.6,91.0) |
| Xinjiang | 652929 | Kalpin County | 4.6 | 3.9 | 5.4 |  | 77.5(59.4,91.1) | 73.8(54.4,88.6) | 83.0(65.1,94.1) |
| Xinjiang | 650203 | karamay District | 3.8 | 2.9 | 4.6 |  | 74.4(50.9,92.1) | 78.4(57.2,95.7) | 67.1(44.1,89.4) |
| Xinjiang | 653201 | Hotan City | 3.4 | 2.8 | 4.1 |  | 73.9(60.0,85.5) | 71.6(55.1,85.8) | 78.6(64.9,89.6) |
| Xinjiang | 652924 | Sayar County | 5.2 | 4.3 | 6.0 |  | 73.6(60.1,84.7) | 69.2(54.1,83.3) | 79.8(66.1,89.7) |
| Xinjiang | 650202 | Dushanzi District | 4.1 | 3.1 | 5.1 |  | 71.0(46.2,92.6) | 76.0(51.1,95.8) | 63.6(38.2,91.8) |
| Xinjiang | 652302 | Fukang City | 4.6 | 3.7 | 5.4 |  | 70.4(54.5,85.2) | 59.9(44.0,77.7) | 80.6(59.7,94.7) |
| Xinjiang | 650109 | Midong District | 2.9 | 2.5 | 3.3 |  | 67.1(50.7,82.8) | 72.2(51.1,89.7) | 60.8(45.6,77.0) |
| Xinjiang | 652927 | County | 4.8 | 4.0 | 5.6 |  | 66.0(52.5,77.4) | 61.8(46.4,75.4) | 73.7(61.8,83.8) |
| Xinjiang | 652123 | Toksun County | 5.1 | 4.0 | 6.3 |  | 65.8(45.8,84.9) | 71.6(51.1,90.2) | 62.0(43.4,79.8) |
| Xinjiang | 652922 | Wensu County | 3.6 | 2.9 | 4.3 |  | 64.8(51.3,78.2) | 64.1(48.9,78.6) | 67.9(54.9,80.8) |
| Xinjiang | 650106 | Toutunhe District | 4.3 | 3.7 | 4.9 |  | 64.6(47.1,80.6) | 67.1(47.5,86.8) | 58.8(40.7,79.4) |
| Xinjiang | 652928 | Awat County | 4.0 | 3.1 | 4.9 |  | 63.7(50.0,75.9) | 62.9(47.1,75.8) | 67.5(54.5,79.5) |
| Xinjiang | 650204 | Baijiantan District | 4.1 | 3.2 | 4.9 |  | 62.9(46.1,77.3) | 64.9(49.2,79.9) | 66.5(48.7,79.8) |
| Xinjiang | 654025 | Xinyuan County | 4.6 | 3.4 | 5.7 |  | 62.2(48.6,74.8) | 63.9(50.1,77.6) | 60.9(46.9,74.5) |
| Xinjiang | 653121 | Shufu County | 5.0 | 4.1 | 5.9 |  | 60.7(48.3,72.6) | 58.7(45.4,71.4) | 66.6(55.2,76.7) |
| Xinjiang | 653001 | Artux City | 4.6 | 3.9 | 5.3 |  | 60.1(47.1,73.5) | 54.4(40.5,67.0) | 68.7(54.6,80.6) |
| Xinjiang | 654324 | Habahe(Kaba) County | 4.7 | 3.8 | 5.6 |  | 59.1(39.2,80.6) | 57.5(36.4,80.7) | 63.3(41.6,84.8) |
| Xinjiang | 652101 | Turpan City | 4.1 | 3.2 | 5.1 |  | 55.5(44.3,66.7) | 60.5(48.0,72.4) | 55.0(42.9,67.5) |
| Xinjiang | 652825 | Qiemo(Qarqan) County | 4.3 | 3.4 | 5.0 |  | 55.1(40.9,70.8) | 55.7(38.6,74.5) | 57.3(43.4,71.9) |
| Xinjiang | 652801 | Korla City | 3.8 | 2.9 | 4.7 |  | 54.7(43.5,66.6) | 58.8(45.0,71.9) | 51.8(40.2,65.1) |
| Xinjiang | 653127 | Markit County | 4.4 | 3.6 | 5.3 |  | 54.1(41.3,67.1) | 53.9(39.5,68.0) | 58.1(45.0,71.6) |
| Xinjiang | 652826 | Yanqi Huizu Zizhixian | 3.6 | 2.6 | 4.5 |  | 53.8(38.4,69.3) | 62.0(43.7,80.0) | 42.8(30.2,59.9) |
| Xinjiang | 650104 | Xinshi District | 2.8 | 2.2 | 3.4 |  | 53.6(38.7,70.2) | 58.4(43.0,75.7) | 49.5(35.7,64.3) |
| Xinjiang | 652201 | Hami(Kumul) City | 3.9 | 3.3 | 4.4 |  | 52.7(39.4,67.0) | 47.7(34.8,61.8) | 61.2(45.6,77.2) |
| Xinjiang | 652327 | Jimsar County | 5.0 | 3.7 | 6.1 |  | 52.4(38.9,66.8) | 57.1(41.5,73.5) | 47.2(34.1,65.0) |
| Xinjiang | 654224 | Toli County | 3.5 | 2.6 | 4.5 |  | 52.0(37.0,68.6) | 57.2(39.8,76.7) | 48.8(35.6,64.2) |
| Xinjiang | 653226 | Yutian(Keriya) County | 3.8 | 0.8 | 6.7 |  | 51.7(39.0,64.3) | 69.3(57.1,80.0) | 29.4(20.1,41.9) |
| Xinjiang | 654226 | Zizhixian | 3.3 | 2.5 | 4.1 |  | 50.1(33.8,71.0) | 46.8(31.9,70.0) | 57.8(36.1,83.6) |
| Xinjiang | 654002 | Yining City | 4.2 | 3.3 | 5.0 |  | 49.8(32.6,69.4) | 51.6(33.9,69.4) | 52.0(34.0,71.5) |
| Xinjiang | 654325 | Qinghe(Qinggil) County | 3.4 | 3.1 | 3.7 |  | 49.2(34.0,67.1) | 45.9(30.0,65.7) | 58.5(41.9,75.9) |
| Xinjiang | 652122 | Shanshan(Piqan) County | 4.0 | 3.6 | 4.4 |  | 47.4(30.4,66.0) | 44.3(28.7,63.6) | 57.9(39.2,75.3) |
| Xinjiang | 652822 | Luntai(Buuguur) County | 3.4 | 2.6 | 4.1 |  | 46.2(32.6,60.0) | 44.7(30.9,60.5) | 52.7(37.7,68.5) |
| Xinjiang | 652722 | Jinghe County | 3.2 | 2.5 | 3.9 |  | 45.6(31.6,61.7) | 41.5(27.5,58.8) | 54.1(36.5,74.7) |
| Xinjiang | 654201 | Tacheng City | 4.4 | 3.5 | 5.3 |  | 45.2(32.9,59.4) | 46.7(33.9,64.5) | 43.7(30.7,60.5) |
| Xinjiang | 650102 | Tianshan District | 3.8 | 3.0 | 4.7 |  | 44.4(32.9,57.3) | 47.7(35.0,61.3) | 44.1(33.2,57.5) |
| Xinjiang | 654003 | Kuytun City | 2.5 | 1.9 | 3.1 |  | 44.2(24.2,73.8) | 47.3(24.5,78.6) | 38.8(20.5,72.9) |
| Xinjiang | 654321 | Burqin County | 3.3 | 2.2 | 4.4 |  | 43.6(28.9,63.1) | 43.0(28.4,63.7) | 46.7(29.0,73.1) |
| Xinjiang | 654326 | Jeminay County | 4.1 | 3.2 | 5.0 |  | 43.6(29.3,63.6) | 51.1(30.5,75.3) | 37.3(24.6,52.6) |
| Xinjiang | 653126 | Yecheng County | 2.9 | 2.4 | 3.5 |  | 43.3(31.9,54.8) | 42.4(30.4,56.0) | 51.8(40.0,64.8) |
| Xinjiang | 654028 | Nilka County | 4.4 | 3.5 | 5.3 |  | 42.4(32.1,54.9) | 41.6(30.1,53.6) | 48.1(36.4,60.9) |
| Xinjiang | 650105 | Shuimogou District | 2.6 | 2.2 | 3.0 |  | 42.3(29.6,57.3) | 40.7(27.8,56.4) | 49.2(33.9,66.9) |
| Xinjiang | 652901 | Aksu City | 2.6 | 1.9 | 3.4 |  | 39.4(28.3,51.2) | 38.5(28.9,50.2) | 45.3(33.2,57.7) |
| Xinjiang | 654221 | Emin County | 3.0 | 2.5 | 3.5 |  | 39.2(26.7,56.0) | 33.0(21.0,48.2) | 48.9(33.6,71.2) |
| Xinjiang | 650103 | Saybag District | 2.8 | 2.2 | 3.4 |  | 37.4(22.2,60.2) | 41.4(24.2,65.4) | 35.9(21.1,57.6) |
| Xinjiang | 653022 | Akto County | 3.8 | 3.2 | 4.4 |  | 37.1(20.3,59.1) | 30.8(16.8,50.5) | 48.6(29.1,70.4) |
| Xinjiang | 652923 | Kuqa County | 3.9 | 3.2 | 4.7 |  | 36.9(22.7,55.9) | 35.7(20.4,54.3) | 41.3(25.4,60.2) |
| Xinjiang | 652328 | Mori Kazak Zizhixian | 3.9 | 3.0 | 4.9 |  | 36.8(27.1,48.0) | 39.7(28.2,52.2) | 38.6(27.3,51.1) |
| Xinjiang | 650107 | Dabancheng District | 3.0 | 3.0 | 3.1 |  | 35.7(19.8,62.5) | 26.7(15.4,42.5) | 53.1(27.4,92.1) |
| Xinjiang | 652223 | County | 3.3 | 3.1 | 3.6 |  | 35.0(24.3,48.6) | 31.8(20.4,47.2) | 47.0(30.8,64.6) |
| Xinjiang | 652325 | Qitai County | 3.6 | 3.3 | 3.9 |  | 34.0(24.7,47.0) | 31.5(21.5,44.7) | 40.9(28.9,54.0) |
| Xinjiang | 653130 | Bachu County | 3.0 | 2.3 | 3.6 |  | 33.9(24.9,45.4) | 31.3(22.5,41.7) | 43.6(33.1,56.0) |
| Xinjiang | 652701 | Bole City | 2.4 | 1.9 | 3.0 |  | 32.7(23.4,45.6) | 35.0(24.1,49.0) | 34.1(25.0,47.2) |
| Xinjiang | 652222 | Barkol Kazak Zizhixian | 3.1 | 2.6 | 3.5 |  | 32.1(20.4,47.8) | 38.3(22.9,63.0) | 27.6(18.9,38.2) |
| Xinjiang | 650121 | Urumqi County | 2.7 | 2.3 | 3.0 |  | 31.5(20.9,46.0) | 29.0(18.4,47.2) | 37.5(25.2,56.7) |
| Xinjiang | 654323 | County | 3.2 | 2.6 | 3.8 |  | 30.4(16.8,49.8) | 31.2(16.6,58.2) | 30.8(17.9,49.0) |
| Xinjiang | 653124 | Zepu(Poskam) County | 3.4 | 3.0 | 3.7 |  | 29.5(20.9,39.8) | 23.8(17.0,33.4) | 43.8(32.6,56.2) |
| Xinjiang | 652823 | Yuli(Lopnur) County | 2.7 | 1.9 | 3.5 |  | 27.3(14.8,47.2) | 28.5(15.4,50.4) | 28.1(14.4,52.1) |
| Xinjiang | 653024 | Wuqia(Ulugqat) County | 3.3 | 2.6 | 4.0 |  | 27.0(14.6,43.5) | 26.7(15.1,44.7) | 31.5(17.6,51.8) |
| Xinjiang | 652323 | Hutubi County | 2.3 | 1.9 | 2.6 |  | 26.9(14.9,50.0) | 26.8(14.1,52.0) | 27.2(16.2,45.0) |
| Xinjiang | 652824 | County | 2.1 | 2.0 | 2.3 |  | 24.9(14.7,38.8) | 21.9(11.9,36.5) | 35.5(19.8,53.8) |
| Xinjiang | 654301 | Altay City | 3.0 | 2.2 | 3.9 |  | 24.0(12.9,42.1) | 23.6(12.6,41.5) | 26.1(13.3,52.9) |
| Xinjiang | 654225 | Yumin County | 3.6 | 2.6 | 4.4 |  | 23.6(12.9,40.8) | 23.6(12.5,43.1) | 25.9(13.5,47.4) |
| Xinjiang | 654223 | Shawan County | 2.0 | 1.7 | 2.2 |  | 21.6(12.0,40.3) | 17.8(9.2,32.2) | 29.7(15.1,57.2) |
| Xinjiang | 652829 | Bohu(Bagrax) County | 2.0 | 1.6 | 2.4 |  | 21.5(10.9,43.1) | 24.2(11.1,55.9) | 21.6(12.3,35.1) |
| Xinjiang | 654202 | Usu City | 2.1 | 1.5 | 2.7 |  | 21.4(14.8,30.6) | 22.1(15.3,30.9) | 23.2(16.0,35.0) |
| Xinjiang | 652324 | Manas County | 2.6 | 2.0 | 3.1 |  | 21.3(14.3,31.9) | 24.8(15.5,38.8) | 18.6(12.9,25.9) |
| Xinjiang | 653131 | Zizhixian | 2.5 | 1.5 | 3.4 |  | 20.6(12.1,32.3) | 24.5(13.8,41.2) | 17.6(10.3,29.5) |
| Xinjiang | 652828 | Hoxud County | 1.3 | 1.0 | 1.7 |  | 20.5(12.2,36.5) | 18.3(10.9,34.8) | 26.6(15.0,51.2) |
| Xinjiang | 654023 | Huocheng County | 2.3 | 1.9 | 2.6 |  | 19.2(13.0,26.7) | 17.3(11.8,25.1) | 24.0(16.9,34.0) |
| Xinjiang | 653224 | Lop County | 1.3 | 0.9 | 1.8 |  | 19.1(12.9,27.5) | 17.2(11.6,25.9) | 26.1(17.7,37.4) |
| Xinjiang | 653227 | Minfeng(Niya) County | 1.8 | 1.4 | 2.1 |  | 18.5(10.8,32.4) | 17.6(8.8,34.0) | 25.4(13.7,41.4) |
| Xinjiang | 652723 | Wenquan County | 2.4 | 1.5 | 3.2 |  | 17.5(11.2,27.2) | 20.9(12.2,36.2) | 16.2(10.7,23.9) |
| Xinjiang | 654322 | Fuyun County | 1.4 | 1.0 | 1.8 |  | 17.5(12.0,25.1) | 17.1(11.4,25.0) | 22.3(14.8,31.9) |
| Xinjiang | 654027 | Tekes County | 2.0 | 1.5 | 2.4 |  | 17.2(11.8,24.2) | 16.5(10.7,24.2) | 22.2(16.1,31.4) |
| Xinjiang | 653123 | Yengisar County | 1.0 | 0.5 | 1.4 |  | 16.9(11.3,24.2) | 16(10.2,24.5) | 23.0(15.3,32.5) |
| Xinjiang | 653101 | Kashi(Kaxgar) City | 1.7 | 1.1 | 2.4 |  | 16.8(9.2,28.4) | 16.8(9.0,28.8) | 20.1(11.2,35.6) |
| Xinjiang | 654024 | County | 2.0 | 1.5 | 2.5 |  | 16.3(8.9,27.2) | 15.2(8.2,26.4) | 21.4(12.3,36.2) |
| Xinjiang | 650205 | Orku District | 1.2 | 0.4 | 2.0 |  | 16.2(8.7,29.0) | 21.8(10.7,38.6) | 12.7(6.7,23.2) |
| Xinjiang | 659001 | Shihezi City | 0.5 | 0.4 | 0.6 |  | 15.9(9.9,26.2) | 20.7(11.6,37.1) | 13.6(9.2,19.7) |
| Xinjiang | 652827 | Hejing County | 1.5 | 1.3 | 1.6 |  | 15.1(9.7,22.8) | 13.0(8.0,20.7) | 21.4(13.0,33.2) |
| Xinjiang | 653225 | Qira County | 1.7 | 1.1 | 2.3 |  | 15.0(9.9,22.4) | 15.0(9.4,23.4) | 18.3(12.1,26.0) |
| Xinjiang | 653023 | Akqi County | 2.3 | 1.7 | 2.9 |  | 13.3(7.1,23.8) | 13.4(6.9,26.7) | 15.7(8.4,27.0) |
| Xinjiang | 659004 | Wujiaqu Shi | 0.7 | 0.5 | 0.9 |  | 13.3(9.1,19.6) | 14.5(9.4,22.0) | 14.5(9.4,21.6) |
| Xinjiang | 653129 | Jiashi County | 0.5 | 0.3 | 0.6 |  | 12.3(8.2,18.3) | 10.8(6.9,16.9) | 18(12.1,26.0) |
| Xinjiang | 659002 | Elaer Shi | 0.1 | 0.1 | 0.2 |  | 9.6(6.3,14.5) | 9.6(6.2,14.3) | 12.1(8.0,17.4) |
| Xinjiang | 653223 | Pishan(Guma) County | 0.9 | 0.5 | 1.3 |  | 9.4(5.9,14.4) | 8.6(5.5,13.4) | 12.4(8.0,18.6) |
| Xinjiang | 659003 | Tumushuke Shi | 0.1 | 0.1 | 0.1 |  | 8.7(5.7,13.6) | 8.8(5.7,13.3) | 10.7(7.0,15.7) |
| Xinjiang | 654026 | Zhaosu County | 0.3 | 0.2 | 0.4 |  | 8.2(5.4,12.4) | 7.4(4.6,11.4) | 12.3(8.0,17.6) |
| Xinjiang | 654021 | Yining County | 0.5 | 0.2 | 0.7 |  | 8.1(4.7,13.5) | 7.2(4.0,12.2) | 11.6(6.6,19.4) |
| Xinjiang | 653222 | Moyu(Karakax) County | 1.1 | 0.7 | 1.5 |  | 7.7(4.3,13.5) | 7.0(3.8,13.0) | 10.5(5.8,19.3) |
| Xinjiang | 654022 | Qapqal Xibe Zizhixian | 0.4 | 0.3 | 0.5 |  | 6.7(4.4,10.2) | 6.1(4.0,9.4) | 9.4(6.4,13.7) |
| Xinjiang | 653128 | Yopurga County | 0.6 | 0.4 | 0.9 |  | 6.1(3.9,9.4) | 5.7(3.7,9.0) | 7.9(5.1,12.2) |
| Xinjiang | 653122 | Shule County | 0.5 | 0.3 | 0.7 |  | 3.5(2.2,5.4) | 3.0(1.9,4.8) | 5.2(3.3,7.9) |
| Tibet | 542323 | Gyangzee County | 5.8 | 5.3 | 6.3 |  | 92.8(78.6,98.7) | 93.1(72.4,99.2) | 91.0(75.1,98.5) |
| Tibet | 542301 | Xigazee City | 3.4 | 2.8 | 3.9 |  | 91.8(69.3,98.9) | 85.0(61.2,98.4) | 94.7(75.7,99.7) |
| Tibet | 540127 | Maizhokunggar County | 5.1 | 4.5 | 5.8 |  | 75.6(56.8,90.7) | 83.4(60.2,95.9) | 58.6(41.7,76.6) |
| Tibet | 540102 | Chengguan District | 1.9 | 1.4 | 2.4 |  | 74.7(43.0,95.6) | 74.8(41.1,96.8) | 73.0(40.4,96.8) |
| Tibet | 542622 | Gongbo'gyamda County | 3.9 | 3.4 | 4.5 |  | 73.0(39.7,95.6) | 75.7(37.0,97.7) | 65.9(35.5,93.2) |
| Tibet | 542421 | Nagqu County | 3.0 | 2.4 | 3.5 |  | 72.5(45.4,92.6) | 80.1(50.8,96.7) | 54.3(34.0,78.4) |
| Tibet | 542623 | Mainling County | 5.2 | 4.4 | 6.0 |  | 68.5(43.0,93.0) | 61.3(33.2,89.0) | 76.5(45.3,96.8) |
| Tibet | 542225 | Qonggyai County | 6.4 | 5.5 | 7.2 |  | 63.1(41.8,85.9) | 67.6(40.0,92.0) | 61.5(38.7,78.6) |
| Tibet | 542627 | Nang County | 4.2 | 3.5 | 4.9 |  | 54.5(25.4,89.3) | 61.7(25.1,96.6) | 49.2(22.3,75.7) |
| Tibet | 542132 | Lhorong County | 2.9 | 2.2 | 3.7 |  | 49.7(22.1,83.3) | 48.5(22.6,82.8) | 50.9(24.8,85.6) |
| Tibet | 542621 | Nyingchi County | 3.3 | 2.4 | 4.1 |  | 42.5(21.1,72.7) | 38.2(19.4,60.6) | 53.6(25.2,87.6) |
| Tibet | 542327 | Ngamring County | 4.6 | 4.0 | 5.3 |  | 41.2(22.1,65.8) | 37.1(20.0,60.8) | 48.9(26.7,75.3) |
| Tibet | 542330 | Rinbung County | 4.6 | 4.4 | 4.8 |  | 40.5(23.4,61.8) | 36.2(19.6,62.9) | 47.6(28.0,72.8) |
| Tibet | 542221 | Needong County | 4.0 | 3.8 | 4.3 |  | 40.0(19.7,74.7) | 35.8(16.7,69.4) | 48.9(25.8,78.9) |
| Tibet | 542625 | Bomi(Bowo) County | 2.9 | 2.4 | 3.4 |  | 36.9(19.7,65.4) | 32.9(16.6,65.4) | 43.1(21.3,74.1) |
| Tibet | 542624 | Metog County | 3.4 | 4.1 | 2.8 |  | 35.4(14.9,70.0) | 23.1(8.8,62.5) | 54.3(23.8,87.5) |
| Tibet | 542224 | Sangri County | 4.0 | 3.4 | 4.6 |  | 30.9(18.9,47.9) | 31.0(17.0,51.4) | 35.5(19.5,56.9) |
| Tibet | 542626 | Zayuu County | 2.2 | 1.7 | 2.7 |  | 30.6(15.8,58.9) | 32.5(15.9,66.1) | 31.4(16.0,64.3) |
| Tibet | 542222 | Chanang County | 3.0 | 2.7 | 3.4 |  | 28.8(14.8,55.7) | 26.3(12.7,54.7) | 33.1(16.1,65.3) |
| Tibet | 542228 | Lhozhag County | 5.1 | 4.8 | 5.5 |  | 28.2(16.1,44.1) | 25.0(12.3,43.7) | 36.8(19.4,57.6) |
| Tibet | 542231 | Lhuunzee County | 3.4 | 3.0 | 3.8 |  | 25.9(15.8,42.3) | 24.9(13.2,43.8) | 32.7(17.3,57.9) |
| Tibet | 540126 | Dagzee County | 1.8 | 1.8 | 1.9 |  | 25.4(9.1,64.2) | 22.8(8.3,67.9) | 31.5(12.0,73.9) |
| Tibet | 540125 | Doilungdeeqeen County | 2.4 | 2.4 | 2.3 |  | 19.4(9.1,42.2) | 15.9(7.2,35.4) | 28.5(13.0,57.3) |
| Tibet | 542232 | Cona County | 4.7 | 4.2 | 5.1 |  | 19.3(9.7,32.5) | 19.0(9.0,36.1) | 22.3(10.8,43.2) |
| Tibet | 542128 | Zogang County | 1.9 | 1.5 | 2.4 |  | 18.7(10.0,33.5) | 20.1(10.9,37.5) | 19.3(11.1,34.1) |
| Tibet | 542322 | Namling County | 1.7 | 1.7 | 1.7 |  | 16.4(6.0,47.0) | 11.9(4.4,32.0) | 26.4(9.3,63.2) |
| Tibet | 540123 | Nyeemo County | 2.8 | 2.6 | 3.1 |  | 16.1(9.1,27.0) | 16.4(8.6,31.6) | 18.3(9.6,30.6) |
| Tibet | 542226 | Qusum County | 3.0 | 3.1 | 2.9 |  | 15.4(8.5,27.0) | 11.9(5.9,23.6) | 24.2(12.0,45.0) |
| Tibet | 542233 | Nagarzee County | 2.3 | 2.3 | 2.3 |  | 14.9(8.1,25.9) | 12.3(6.1,23.3) | 21.1(11.1,38.0) |
| Tibet | 540122 | Damxung County | 2.1 | 1.9 | 2.3 |  | 14.0(8.6,21.6) | 12.1(7.1,20.0) | 19.7(11.1,33.5) |
| Tibet | 542331 | Kangmar County | 2.4 | 1.0 | 3.8 |  | 13.2(7.4,24.0) | 21.4(10.8,38.8) | 8.3(4.5,14.9) |
| Tibet | 542338 | Gamba County | 2.2 | 2.3 | 2.1 |  | 12.5(3.7,41.1) | 10.1(2.6,44.0) | 19.0(6.4,55.1) |
| Tibet | 540124 | Quuxuu County | 1.3 | 1.1 | 1.6 |  | 12.0(7.2,18.9) | 12.4(7.5,20.6) | 14.2(8.5,23.2) |
| Tibet | 542425 | Amdo County | 0.5 | 0.4 | 0.6 |  | 10.8(5.1,27.9) | 10.9(4.8,30.3) | 12.8(6.5,31.5) |
| Tibet | 542229 | Gyaca County | 1.3 | 1.1 | 1.5 |  | 9.4(4.4,19.8) | 10.0(4.5,22.1) | 12.0(4.9,25.4) |
| Tibet | 542423 | Biru County | 0.4 | 0.4 | 0.5 |  | 9.2(4.4,19.6) | 8.5(4.0,21.9) | 10.4(5.2,22.2) |
| Tibet | 542124 | Riwoqee County | 0.3 | 0.2 | 0.5 |  | 9.1(5.1,15.9) | 8.9(4.6,15.8) | 12.7(6.5,22.3) |
| Tibet | 542329 | Bainang County | 0.4 | 0.4 | 0.3 |  | 8.5(4.9,14.1) | 7.3(4.3,11.8) | 13.2(7.9,21.0) |
| Tibet | 542429 | Baqeen County | 0.2 | 0.1 | 0.3 |  | 8.5(3.9,20.9) | 8.9(3.8,25.5) | 9.4(4.8,17.5) |
| Tibet | 542125 | Deengqeen County | 0.1 | 0.1 | 0.1 |  | 7.8(3.4,16.0) | 6.9(3.1,14.9) | 11.2(5.2,24.3) |
| Tibet | 542523 | Gar County | 0.4 | 0.2 | 0.5 |  | 7.7(4.3,13.1) | 7.4(4.1,13.6) | 10.1(5.7,17.2) |
| Tibet | 542227 | Comai County | 2.2 | 1.3 | 3.2 |  | 7.6(4.4,14.0) | 10.1(5.2,19.9) | 6.4(3.3,12.9) |
| Tibet | 542126 | Chagyab County | 0.7 | 0.5 | 0.9 |  | 7.1(4.0,13.3) | 7.3(3.8,14.9) | 7.7(4.0,14.2) |
| Tibet | 542121 | Qamdo County | 0.3 | 0.2 | 0.3 |  | 7.0(3.2,16.0) | 6.8(2.8,15.4) | 9.5(4.3,19.9) |
| Tibet | 542223 | Gonggar County | 1.3 | 1.2 | 1.5 |  | 6.9(4.2,11.1) | 6.5(3.9,11.0) | 8.9(5.2,15.2) |
| Tibet | 542328 | Xaitongmoin County | 0.0 | 0.0 | 0.1 |  | 6.9(3.9,12.6) | 7.1(4.0,14.2) | 7.9(4.9,12.5) |
| Tibet | 542422 | Jiali(Lhari) County | 0.2 | 0.2 | 0.2 |  | 6.9(2.5,18.8) | 6.4(2.1,25.8) | 8.8(3.6,20.6) |
| Tibet | 542424 | Nyainrong County | 0.3 | 0.1 | 0.5 |  | 6.1(3.0,12.6) | 5.7(2.9,13.5) | 8.3(4.1,18.3) |
| Tibet | 542133 | Banbar County | 0.1 | 0.1 | 0.1 |  | 5.9(2.7,13.1) | 5.0(2.2,10.0) | 9.4(4.0,22.3) |
| Tibet | 542430 | Nyima County | 0.3 | 0.2 | 0.3 |  | 5.9(2.4,16.2) | 6.3(2.2,25.0) | 7.2(3.0,16.6) |
| Tibet | 540121 | Lhuunzhub County | 0.2 | 0.1 | 0.3 |  | 5.8(3.4,9.9) | 6.2(3.5,10.2) | 6.5(3.9,10.9) |
| Tibet | 542427 | Sog County | 0.2 | 0.2 | 0.1 |  | 5.7(2.9,10.7) | 4.9(2.6,8.6) | 9.0(4.7,20.1) |
| Tibet | 542336 | Nyalam County | 1.1 | 1.0 | 1.2 |  | 5.6(2.9,11.0) | 5.4(2.5,12.0) | 6.6(3.4,12.0) |
| Tibet | 542324 | Tingri County | 0.6 | 0.6 | 0.5 |  | 5.4(2.1,12.8) | 4.4(2.0,9.9) | 8.1(3.5,21.5) |
| Tibet | 542428 | Bangoin County | 0.3 | 0.2 | 0.4 |  | 5.4(2.3,12.0) | 5.6(2.4,12.1) | 7.0(2.9,14.9) |
| Tibet | 542431 | Shuanghu County | 0.3 | 0.2 | 0.3 |  | 5.4(2.2,12.0) | 4.8(2.1,11.3) | 7.5(3.1,17.4) |
| Tibet | 542337 | Saga County | 0.8 | 1.1 | 0.6 |  | 5.2(1.7,15.4) | 3.1(1.2,7.3) | 10.2(3.1,37.2) |
| Tibet | 542332 | Dinggyee County | 0.5 | 0.6 | 0.5 |  | 4.9(2.1,10.7) | 4.2(1.8,9.4) | 7.2(3.0,16.1) |
| Tibet | 542326 | Lhazee County | 0.1 | 0.1 | 0.1 |  | 4.8(2.1,10.8) | 4.0(1.8,8.9) | 7.4(3.0,18.8) |
| Tibet | 542122 | Jomda County | 0.1 | 0.1 | 0.2 |  | 4.7(2.4,9.5) | 4.4(2.2,9.0) | 6.0(2.9,12.3) |
| Tibet | 542325 | Sa'gya County | 0.1 | 0.1 | 0.1 |  | 4.3(2.5,7.3) | 3.4(2.0,5.7) | 6.4(3.6,12.4) |
| Tibet | 542129 | Mangkam County | 0.5 | 0.2 | 0.7 |  | 4.0(1.7,9.8) | 4.1(1.6,9.5) | 4.1(1.6,10.4) |
| Tibet | 542335 | Gyirong County | 0.5 | 0.6 | 0.4 |  | 3.9(2.1,7.1) | 3.1(1.6,5.9) | 6.1(3.0,12.1) |
| Tibet | 542123 | Konjo County | 0.1 | 0.1 | 0.1 |  | 3.7(1.9,7.3) | 3.5(1.8,7.3) | 4.6(2.5,8.4) |
| Tibet | 542426 | Xainza County | 0.2 | 0.1 | 0.2 |  | 3.7(1.5,8.3) | 3.2(1.4,8.0) | 5.2(2.3,12.2) |
| Tibet | 542522 | Zanda County | 0.0 | 0.0 | 0.0 |  | 3.7(2.0,6.6) | 3.3(1.8,5.8) | 5.3(2.9,9.3) |
| Tibet | 542127 | Baxoi County | 0.3 | 0.2 | 0.4 |  | 3.3(2.0,5.6) | 3.2(1.8,5.3) | 4.5(2.6,7.1) |
| Tibet | 542334 | Yadong(Chomo) County | 0.2 | 0.1 | 0.3 |  | 3.3(1.4,7.0) | 3.1(1.4,6.6) | 4.5(1.9,9.5) |
| Tibet | 542525 | Gee'gyai County | 0.1 | 0.0 | 0.2 |  | 3.2(1.6,6.1) | 2.7(1.3,5.4) | 4.9(2.5,8.6) |
| Tibet | 542527 | Coqeen County | 0.1 | 0.0 | 0.2 |  | 3.1(1.4,7.4) | 3.2(1.4,8.6) | 3.3(1.7,6.1) |
| Tibet | 542526 | Geerzee County | 0.1 | 0.2 | 0.0 |  | 2.9(1.5,5.1) | 2.4(1.3,4.3) | 4.4(2.4,7.6) |
| Tibet | 542333 | Zhongba County | 0.0 | 0.0 | 0.0 |  | 2.7(1.3,6.0) | 2.5(1.1,5.7) | 3.9(1.7,9.0) |
| Tibet | 542524 | Rutog County | 0.0 | 0.0 | 0.0 |  | 2.6(1.4,4.7) | 2.3(1.3,4.3) | 3.9(2.2,7.2) |
| Tibet | 542521 | Burang County | 0.0 | 0.0 | 0.0 |  | 2.4(1.0,5.0) | 2.2(0.9,5.1) | 2.8(1.1,6.1) |
